# Palladium/N-heterocyclic carbene catalysed regio and diastereoselective reaction of ketones with allyl reagents via inner-sphere mechanism

**DOI:** 10.1038/ncomms11806

**Published:** 2016-06-10

**Authors:** Da-Chang Bai, Fei-Le Yu, Wan-Ying Wang, Di Chen, Hao Li, Qing-Rong Liu, Chang-Hua Ding, Bo Chen, Xue-Long Hou

**Affiliations:** 1 State Key Laboratory of Organometallic Chemistry, Shanghai Institute of Organic Chemistry, Chinese Academy of Sciences, 345 Lingling Road, Shanghai 200032, China; 2 Department of Chemistry and Chemical Biology, Baker Laboratory, Cornell University, Ithaca, New York 14853, USA; 3 Shanghai–Hong Kong Joint Laboratory in Chemical Synthesis, Shanghai Institute of Organic Chemistry, Chinese Academy of Sciences, 345 Lingling Road, Shanghai 200032, China

## Abstract

The palladium-catalysed allylic substitution reaction is one of the most important reactions in transition-metal catalysis and has been well-studied in the past decades. Most of the reactions proceed through an outer-sphere mechanism, affording linear products when monosubstituted allyl reagents are used. Here, we report an efficient Palladium-catalysed protocol for reactions of β-substituted ketones with monosubstituted allyl substrates, simply by using N-heterocyclic carbene as ligand, leading to branched products with up to three contiguous stereocentres in a (*syn, anti*)-mode with excellent regio and diastereoselectivities. The scope of the protocol in organic synthesis has been examined preliminarily. Mechanistic studies by both experiments and density functional theory (DFT) calculations reveal that the reaction proceeds via an inner-sphere mechanism—nucleophilic attack of enolate oxygen on Palladium followed by C–C bond-forming [3,3']-reductive elimination.

## Abstract (web summary)

Palladium catalyzed allylic substitution reactions typically proceed via an outer sphere mechanism, yielding predominately linear products. Here, the authors report an inner sphere process for the allylic substitution of ketone enolates, giving branched products with up to three contiguous stereocentres.

## Graphical abstract

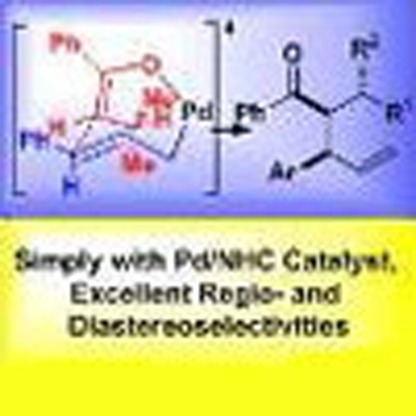

The palladium-catalysed allylic substitution reaction of allyl reagents with nucleophiles has become one of the most important carbon–carbon and carbon-hetero-atom bond-forming processes^1^,^2^,^3^,^4^. In most cases, the reaction proceeds through an outer-sphere mechanism, in which the nucleophile attacks the allyl carbon of π-allyl-Pd complex, and affords linear products when monosubstituted allyl reagents are used^1^,^2^,^3^,^4^,^5^,^6^,^7^,^8^,^9^,^10^,^11^,^12^. Less commonly, the reaction can follow an inner-sphere mechanism; in this case, the nucleophilic attack is targeted at Pd, forming an intermediate at first, followed by reductive elimination^13^,^14^,^15^,^16^,^17^,^18^,^19^,^20^. Recently, several studies on Pd-catalysed allylic substitution/coupling reactions via inner-sphere mechanism have been reported^21^,^22^,^23^,^24^,^25^,^26^,^27^,^28^,^29^,^30^,^31^,^32^,^33^. The key C–C bond-forming step was proposed to occur via [3,3']-reductive elimination in an allyl-Pd-allyl or allyl-Pd-nucleophile intermediate. For example, Stoltz^27^ achieved the construction of a chiral quaternary carbon centre at the α-position of cyclic ketones using unsubstituted allyl carbonates in an intramolecular decarboxylative process (Fig. 1a)^25^,^26^,^27^. Morken realized excellent regio- and enantio-selectivities in the cross-coupling of monosubstituted allyl reagents with allyl boronates under Pd catalysis (Fig. 1b)^28^,^29^,^30^,^31^,^32^. These results reveal the unique character of the inner-sphere mechanism; in particular, branched products can be formed as major products when monosubstituted allyl reagents are used^21^,^28^. Pd-catalysed allylic substitution via an inner-sphere mechanism also found applications in organic synthesis^26^,^32^. However, so far only limited reaction modes following an inner-sphere process have been explored, that is, coupling of diallyl-Pd-species^21^,^28^,^29^,^30^,^31^ and an intramolecular decarboxylative process, with narrow substrate scope for the latter (Fig. 1a)^27^,^34^. The development of more reaction modes with wider substrate scope remains a great challenge.

Recently, we developed a regio- and chemo-tunable Pd-catalysed allylic alkylation with nitrogen nucleophiles, yielding branched allylated products with excellent regioselectivity; mechanistic investigations including DFT calculations support an inner-sphere mechanism for the reaction (Fig. 1c)^33^. In further investigations, we realized (with an oxygen nucleophile) Pd-catalysed allylic alkylation of monosubstituted allyl reagents by acyclic ketones. Acyclic compounds having up to three contiguous stereocentres can be obtained with excellent regio and diastereoselectivities (Fig. 1d). The key to high selectivity is the use of an *N*-heterocyclic carbene (NHC) as ligand. In this paper, we would like to report this new type of Pd/NHC-catalysed allylic alkylation (Fig. 1d) as well as mechanistic studies on the reaction by both experiments and DFT calculations. The scope of the protocol in organic synthesis is also examined preliminarily.

## Results

### Reaction design

To test whether oxygen nucleophiles are also compatible, as nitrogen nucleophiles are, in the Pd/PPh_3_-catalysed allylic substitution to give branched products via an inner-sphere mechanism^33^, we began our investigations with the reaction of propiophenone **1a** with cinnamyl *tert*-butyl carbonate **2a**, using [Pd(*η*^3^-C_3_H_5_)Cl]_2_ and PPh_3_ as catalyst and *t*-BuOK or lithium diisopropylamide as base. Unfortunately, the linear product was obtained as the major component in both cases, with branched to linear ratios (B/L) of 6/94 and 15/85, respectively, despite high yields (95% and 98%). Considering the similar coordination chemistry of NHC and phosphine ligands but different stereochemistry of reactions using the two types of ligands^35^, we envisioned that using NHC ligand, instead of PPh_3_, in the current reaction might lead to predominantly branched product. Indeed, with imidazolium salts IPr·HCl (**L1**), the precursor to corresponding NHC ligand on deprotonation, branched product **3a** was generated in 95% yield with a B/L ratio of 96/4 in the reaction of **1a** with **2a** (entry 1, Table 1). Since NHC has a better performance than PPh_3_, leading to highly selective formation of branched product in the reaction, only NHC ligands were used in the subsequent reaction condition screening.

The diastereomeric ratio (*dr*) for the reaction of **1a** and **2a** in the presence of an NHC ligand, determined by the ratio of *syn*-**3**/*anti*-**3**, was modest, 20/80 (entry 1, Table 1). To increase the diastereoselectivity of this reaction, ketones of substituted **1a** were tested. Reaction of butyrophenone **1b** did not show any increase in diastereoselectivity (entry 2, Table 1). Surprisingly, when β-methyl butyrophenone **1c** was use as the substrate, the diastereoselectivity was reversed, and the *syn*-isomer became the major product with a *syn/anti* ratio of 85/15 (entry 3 versus entry 1, Table 1).

Next, we examined the effect of the structure of the NHC ligand on the diastereoselectivity of this reaction. Using an NHC ligand with a less sterically bulky aryl substituent, derived from **L2**, or 1,3-di-*tert*-butyl NHC, derived from **L3**, led to predominant linear product in low yield (entries 4 and 5, Table 1). A lower yield was obtained if the reaction was run at lower temperature (entry 6 versus entry 3, Table 1). Using an NHC ligand derived from S-IPr·HCl (**L4**), a dihydrogenated form of IPr·HCl (**L1**), high yields with excellent regio and diastereoselectivities were achieved (entry 7, Table 1; see Supplementary Table 5 for details). These results show that the structure of the NHC ligand is critical in controlling the regio and diastereoselectivities of the reaction.

In addition to ketones, some other carbonyl compounds were also examined in our catalyst system. Reaction of butyryl trimethylsilane **1ba** led to **3ba** in 72% yield with excellent regio and diastereoselectivities (entry 8, Table 1). Similar yield and B/L ratio, yet much lower dr were observed in the reaction of β-methyl substituted butyryl trimethylsilane **1ca** (entry 9, Table 1). No desired product was observed when *N*-acyl pyrrole **1bb** (entry 10, Table 1) or ethyl acetate (not shown in the table) was used as substrate.

We also investigated preliminarily the enantioselectivity of reactions of **1b** and **1c** using chiral NHCs. So far, only poor enantioselectivity with lower regio and diastereoselectivities has been achieved (see Supplementary Tables 7 and 8 for details). Realizing highly asymmetric induction in this type of reaction is the task of further investigations.

### Using β-substituted ketones to construct three stereocentres

The structural motif of three contiguous stereocentres in an acyclic system can be found in a wide range of natural products, pharmaceuticals and synthetic building blocks^36^,^37^,^38^. In view of above results and following investigations on the mechanism (*vide infra*, Mechanistic investigations), we reasoned that highly selective formation of three contiguous stereocentres in this reaction might be possible if we introduce a chiral centre at the β position of **1c** by changing one methyl at the β position to other groups. Following this thought, reactions of ketones **1** with different β-substituents were carried out. While using ketones **1d** and **1e** with, respectively, ^*n*^hexyl and ethyl at the β position led to very low diastereoselectivities (entries 1 and 2, Table 2), using β-phenyl substituted ketone **1f** results in 98% branched product (*syn*, *anti*)-**3f** with 86/14 *dr* (entry 3, Table 2; see Supplementary Table 6 for details). These results seemed to indicate that unsaturated groups at the β position are necessary to achieve high *dr*, presumably due to some favourable interaction between the unsaturated group at the β position of ketone and the phenyl on allyl^39^,^40^,^41^. It could be deduced that high stereoselectivity might also be obtained if other unsaturated groups were installed at the β position of ketone. Indeed, higher stereoselectivity was observed in reactions of β-alkenyl ketone **1g** (ref. 42) and **1h**, and β-alkynyl ketone **1i** (ref. 43), with *dr* of 77/23, 86/14 and 93/7, respectively (entries 4–6 versus entry 1, Table 2). Note, also the high yields and regioselectivity for **3g** (92%, B/L=93/7) and **3i** (99%, B/L=95/5), and somewhat lower yield and regioselectivity of reaction of *Z*-type β-alkenyl ketone **1h**, compared with those of reaction of *E*-type β-alkenyl ketone **1g**.

### Substrate scope

With the optimized reaction conditions, the compatibility of both ketone substrates and allyl reagents in the reaction was studied. As depicted in Table 3, the reaction is efficient for a wide range of β-substituted ketones, **1f–1l**, affording the branched products **3f–3l** bearing three contiguous stereocentres in nearly quantitative yields, with excellent regio and diastereoselectivities. Specifically, not only β-phenyl ketones (**1f** and **1j**), but also β-alkynyl ketones with a variety of terminal substituents on alkynyl (**1i**, **1k** and **1l**) are suitable substrates for this reaction. Notably, ketones with sterically bulky substituents also react smoothly to produce the corresponding allylated product **3y** in high yield with high diastereoselectivity, despite the somewhat lower B/L ratio of 85/15. Regarding the allyl reagents, we first examined mono-aryl substituted allyl reagents with both electron-withdrawing and donating substituents. With β-triisopropylsilylacetylenyl ketone **1l** as nucleophile, allylated product **3l**–**3v** were obtained in excellent yields (> 90%) with excellent regio and diastereoselectivities (B/L>90/10 and *dr*>93/7). Using β-methyl butyrophenone **1c** as nucleophile, equally good yields and selectivities were obtained for **3ca**–**3ce**. We also found that furanyl- and naphthyl-substituted allyl reagents are compatible in the reaction, delivering **3w** and **3x**. Note that the structures of one enantiomer of each of (±)-*syn*-**3c** and (±)-(*syn*, *anti*)-**3f** have been determined by X-ray crystallography and are shown in Table 3.

### Applications

**Figure 2:**
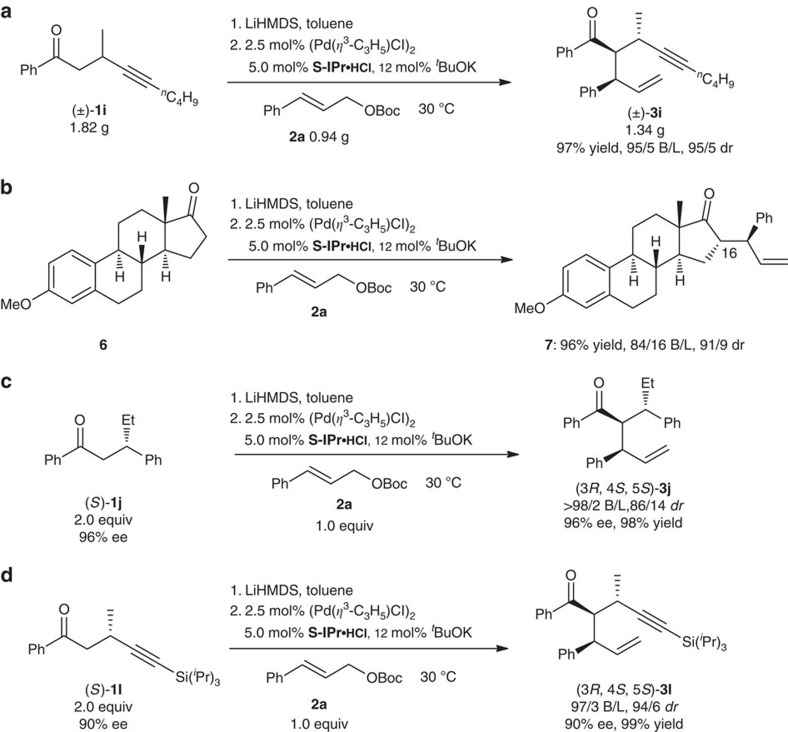
Applications of the titled reaction. (**a**) Reaction on a gram-scale. (**b**) Allylation of estrone 3-methyl ether at the C16 position. (**c**) and (**d**) Reactions with chirality transfer from optically active substrates without loss of optical activity.

To demonstrate the usefulness of this protocol, gram-scale reactions were carried out. Under optimal conditions, reaction of 1,6-enyne **1i** can be scaled up, affording 1.34 g of product **3i**, with almost no decrease in yield and selectivities (Fig. 2a). Our protocol can be used to allylate the C16 position of estrone 3-methyl ether **6**, providing product **7** in 96% yield with 84/16 B/L and 91/9 *dr* (Fig. 2b). Since optically active β-substituted ketones are easily available^44^,^45^,^46^,^47^,^48^,^49^, the capacity of this reaction for chirality transfer from optically active substrates was explored (Fig. 2cd). Products (3*R*, 4*S*, 5*S*)-**3j** and (3*R*, 4*S*, 5*S*)-**3l** were obtained in excellent yields with excellent regio and diastereoselectivities without loss of optical activity when optical active ketones (*S*)-**1j** (ref. 47) and (*S*)-**1l** (ref. 48) were used.

**Figure 3:**
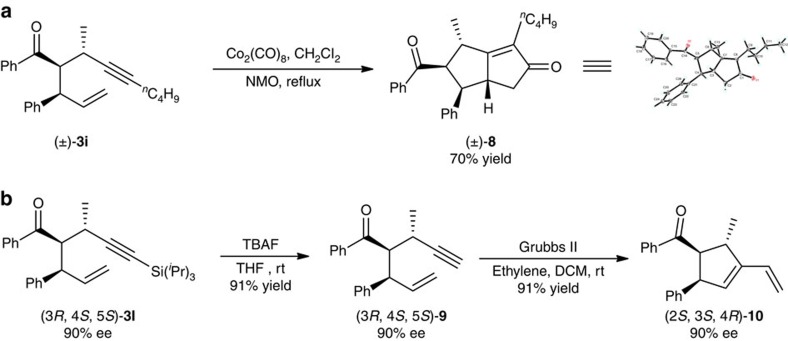
Transformation of reaction products. (**a**) Pauson–Khand reaction with **3i**. (**b**) Cyclization reaction of **3l** without loss of optical activity.

1,6-Enynes are important and versatile synthetic intermediates^50^,^51^. Thus, further transformations of the 1,6-enyne product **3i**, generated by the titled Pd/NHC-catalysed allylic substitution, were studied. Subjecting **3i** to Pauson–Khand reaction conditions leads to bicyclo[3.3.0]octane **8**, which has been found to be a core skeleton in many natural products^52^,^53^, in 70% yield with excellent stereoselectivity (Fig. 3a). Also, desilylation of **3i** with tetra-*n*-butylammonium fluoride, followed by intramolecular cyclization in the presence of ethylene and Grubbs II catalyst, yields tetra-substituted cyclopentene (2*S*, 3*S*, 4*R*)-**10** with three contiguous chiral centres in 83% yield without loss of optical activity (Fig. 3b). The 1-vinyl cyclopentene framework in **10** is a useful building block in the synthesis of many complex molecules^54^,^55^,^56^,^57^.

### Mechanistic investigations

**Figure 4:**
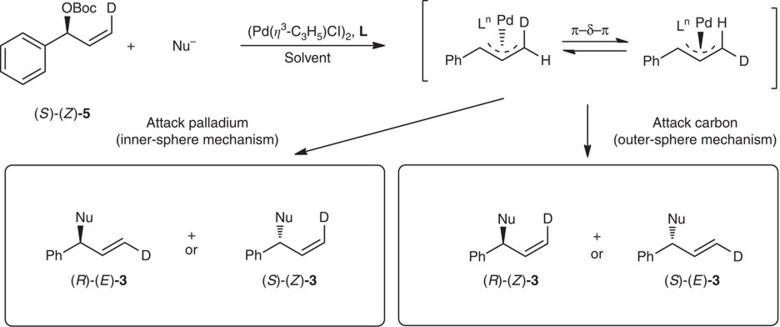
Stereochemistry of Pd-catalysed reaction of nucleophile with (*S*)-(*Z*)-5 via outer- and inner-sphere attack mechanisms. (*R*)-(*E*)-**3** and/or (*S*)-(*Z*)-**3** will be obtained by nucleophile attacks on palladium via an inner-sphere mechanism, whereas (*R*)-(*E*)-**3** and/or (*S*)-(*Z*)-**3** will be afforded if the nucleophile attacks on carbon via an outer-sphere mechanism.

To shed some light on the reaction mechanism, deuterium-labelled and cyclic allyl reagents were used as probes. According to the known stereochemistry Pd-catalysed allylic substitution^1^,^2^,^3^, (*S*)-(*Z*)-**3** and (*R*)-(*E*)-**3** will be formed if the reaction of deuterium-labelled, optically active, allyl ester (*S*)-(*Z*)-**5** proceeds via an inner-sphere mechanism, while (*R*)-(*Z*)-**3** and (*S*)-(*E*)-**3** will be afforded when the outer-sphere mechanism is at work (Fig. 4).

**Figure 5:**
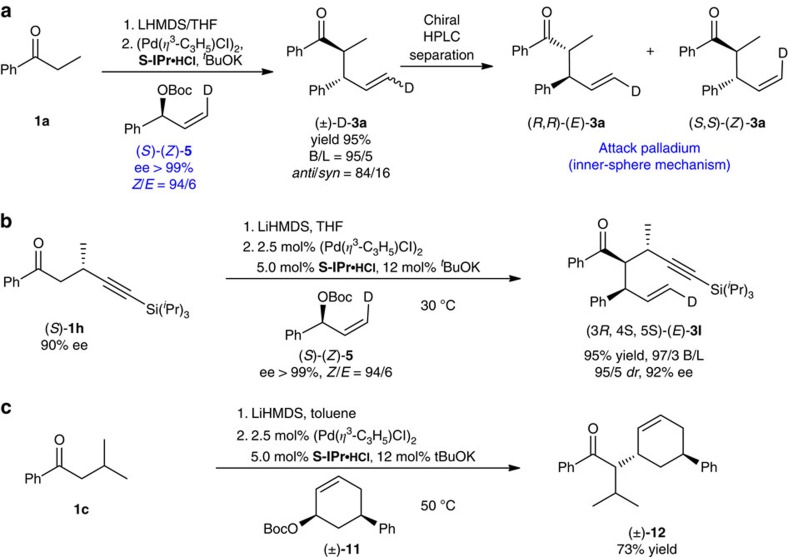
Mechanistic investigations. (**a**) (*R, R*)-(*E*)-**3a** And (*S, S*)-(*Z*)-**3a** were afforded by the reaction of (*S*)-(*Z*)-**5** with ketone **1a**, indicating that the reaction proceeds via the inner-sphere mechanism. (**b**) (3*R*, 4*S*, 5*S*)-(*E*)-**3i** was the only product in the reaction of (*S*)-(*Z*)-**5** supports an inner-sphere mechanism. (**c**) Inversion of the stereocentre at the allyl position of cyclohexene **11** in the reaction confirms also the inner-sphere mechanism.

When (*S*)-(*Z*)-**5** was subjected to reaction with ketone **1a** (Fig. 5a)^33^, only (*R, R*)-(*E*)-**3a** and (*S, S*)-(*Z*)-**3a** were obtained after chiral HPLC separation, which clearly suggests that the reaction proceeds via an inner-sphere mechanism. Similarly, the reaction of (*S*)-(*Z*)-**5** with β-substituted ketones (*S*)-**1h** yielding (3*R*, 4*S*, 5*S*)-(*E*)-**3i** (Fig. 5b), also supports that the reaction proceeds via an inner-sphere mechanism. In addition, the fact that the reaction of *cis*-disubstituted cyclohexene **11** afforded *trans*-product **12** in 73% isolated yield as a single diastereomer (determined by ^1^H NMR spectroscopy), again, confirms the inner-sphere mechanism (Fig. 5c)^7^,^10^.

**Figure 6:**
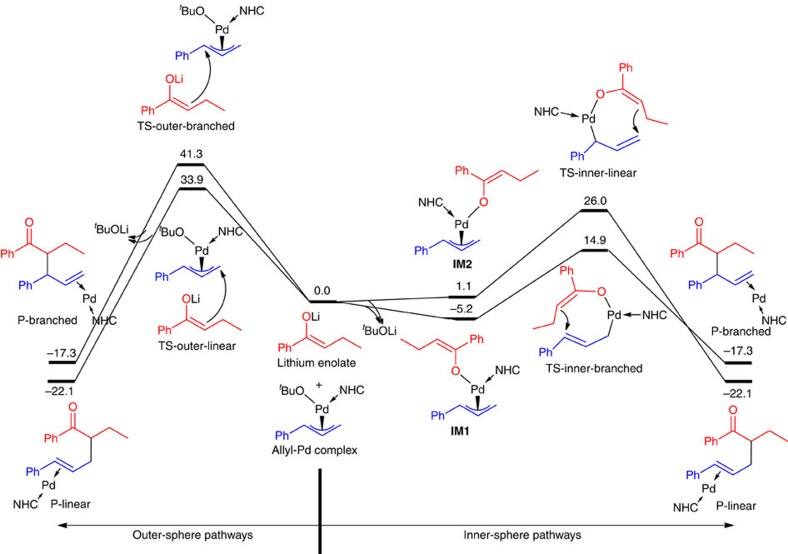
DFT calculations of the free-energy surface of plausible inner-sphere and outer-sphere pathways of reaction of ketone **1b**. Free energies in solvent Δ*G*_sol_ (in kcal mol^−1^) are computed at ωB97XD/def2-TZVP/SMD//ωB97XD/SDDAll level of theory. (More detailed calculations on E-enolate and η^1^-allyl-Pd intermediates can be found in Supplementary Figures 69 and 71). These calculations suggest that the inner-sphere pathway leading to branched product is the most favourable pathway, in agreement with experiment.

To further understand the reaction mechanism, DFT calculations were carried out on both inner-sphere and outer-sphere pathways (Fig. 6). The energy reference is set as separated reactants (that is, lithium enolate and allyl-Pd complex). In outer-sphere pathways, the transition states leading to linear product (TS-outer-linear) and branched product (TS-outer-branched) were calculated to be, respectively, 33.9 and 41.3 kcal mol^−1^ above the separated reactants. These very high-reaction barriers suggest that the outer-sphere mechanism is unlikely to be operative. In addition, if an outer-sphere pathway were followed, linear product would be predicted to be formed exclusively, which contradicts experimental observations. In inner-sphere pathways, lithium enolate first reacts, exothermically, with allyl-Pd complex to yield allyl-Pd enolate (**IM1** or **IM2**) and ^*t*^BuOLi; then, [3,3'] reductive elimination takes place in the allyl-Pd enolate. The transition state for the branched product (TS-inner-branched) was computed to be 14.9 kcal mol^−1^ above separated reactants, much lower in energy than TS-inner-linear and the two transition states in outer-sphere pathways. These calculations suggest that the branched product is the kinetically favourable one and should be formed as the major product in experiment through an inner-sphere mechanism. We note here previous theoretical work on the inner- and outer-sphere mechanisms of allylic alkylation of lactones^58^.

**Figure 7:**
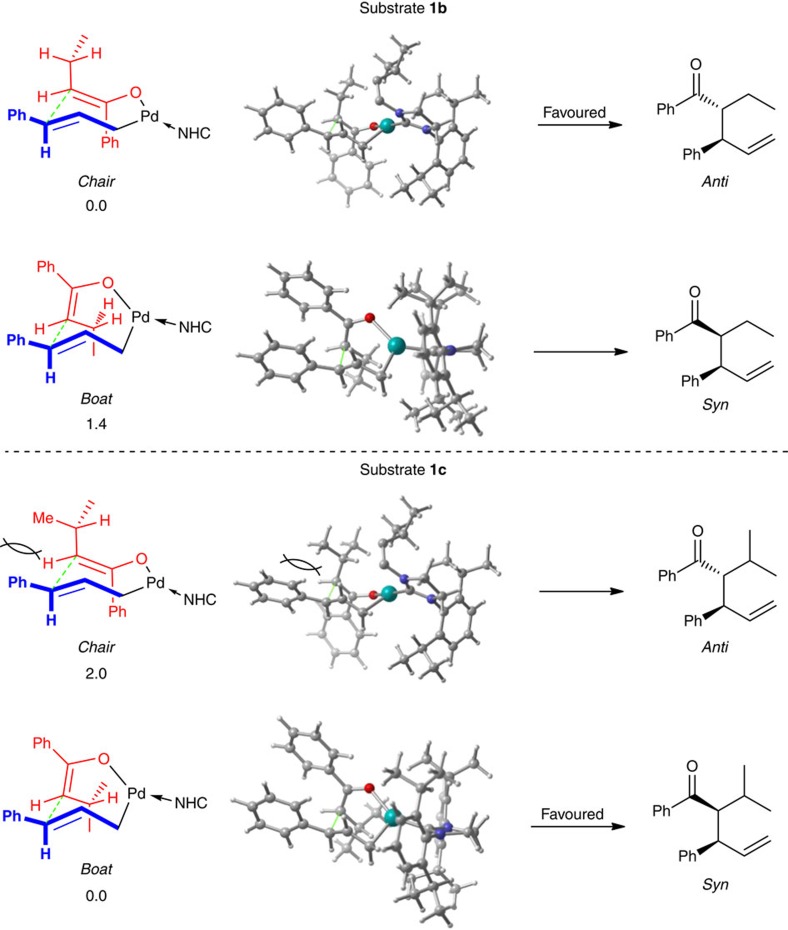
DFT calculated two major conformations of TS-inner-branched for reactions of ketone **1b** and **1c**. For substrate **1b**, the chair transition state leading to anti product is favoured, whereas for substrate **1c**, the boat transition state leading to syn product is favoured. Detailed analysis and calculations on possible conformations can be found in Supplementary Figures 70–73. Numbers at the bottom of each structure are relative free energies ΔΔ*G*_sol_ (in kcal mol^−1^), computed at ωB97XD/def2-TZVP/SMD//ωB97XD/SDDAll level of theory. In the pictures in the central column, some atoms of the bulky substituents on NHC are omitted for clarity.

To explain the stereochemistry of the reaction, possible conformations of the favourable transition state (TS-inner-branched) of the inner-sphere pathway were explored by DFT calculations (Fig. 7). We find that for the reaction of ketone **1b**, the seven-membered ring transition state with a chair conformation is favoured over the boat transition state by 1.4 kcal mol^−1^. The favourable chair transition state leads to anti product, in agreement with experiment. On the other hand, the boat transition state was predicted to be lower in energy than the chair transition state by 2.0 kcal mol^−1^ for the reaction of ketone **1c**, suggesting that syn product should be the major one. The reversal of diastereoselectivity, predicted by calculations and observed in experiments, on going from reaction of **1b** to **1c**, is probably associated with 1,3-diaxial interaction in the chair transition state. The steric encumbrance in the chair transition state for **1c**, arising from the phenyl on the allyl and one methyl at the β position of the enolate, might destabilize the chair transition state, making it higher in energy than the boat transitions state. However, such, presumably unfavourable, 1,3-diaxial interaction is absent in the chair transition state for **1b**.

In summary, a novel, simple and effective Pd/NHC-catalysed protocol has been developed to produce acyclic α-allylated ketones bearing three contiguous stereocentres with excellent regio and diastereoselectivities, starting with readily available ketones and allyl reagents. This reaction features facile yet highly efficient Pd catalysis, the use of commercially available NHC ligands and wide substrate scope. It was found that substituents on the NHC and β-substituents on ketones have a critical impact on the stereochemistry of the reaction. The synthetic applications of the methodology have also been examined preliminarily. The products from current protocol are likely to be useful in the synthesis of more complex molecules. Mechanistic investigations using stereo-probing allyl reagents and DFT calculations suggest that the reaction proceeds via an inner-sphere mechanism. DFT calculations also showed that the diastereoselectivity of this reaction is highly dependent on the β-substituent on ketone.

## Methods

### Materials

For ^1^H and ^13^C NMR spectra of the compounds in this article, see Supplementary Figs 1–66. For X-ray analysis data of **3c**, **3f**, **7** and **8**, see Supplementary Tables 1–4. Details of DFT calculations see Supplementary Figs 67–73 and Supplementary Information Computational methods. For coordinates and energies of calculated structures, see Supplementary Data 1.

### General

Commercially available reagents were used without further purification. Solvents were purified before use according to the standard methods. Unless otherwise noted, all reactions were carried out under an atmosphere of argon and flame-dried glassware with standard vacuum-line techniques. NMR spectra are recorded at room temperature on 400 MHz Varian-400, 400M Agilent-400 or 300 MHz Bruker AM-300 NMR spectrometers. The chemical shifts for ^1^H NMR are reported in p.p.m. from tetramethylsilane with the solvent resonance as the internal standard (7.26 p.p.m. for CHCl_3_). Data are reported as follows: chemical shift, multiplicity (s=singlet, d=doublet, t=triplet, q=quartet, sept=septet, bs=broad singlet, m=multiplet), coupling constants (Hz) and integration. Chemical shifts are reported in p.p.m. from tetramethylsilane with the solvent resonance as the internal standard (CDCl_3_: 77.15 p.p.m.). Infra-red spectra were measured in cm^−1^. HRMS were carried out on the Finnigan MAT 8430 spectrometer. Thin-layer chromatography was performed on pre-coated glassback plates and visualized with ultraviolet light at 254 nm. Flash column chromatography was performed on silica gel.

### General procedure for the palladium-catalysed allylic alkylation

A dry Schlenk tube was flame-dried and flushed with Argon. Ketone **1** (0.4 mmol) and toluene (2.0 ml) were added into the dry Schlenk tube. LiHMDS(1.0 M in THF, 0.4 ml, 0.4 mmol) were added at 0 °C and stirred at room temperature for 30 min. In a separated flask, [Pd(*η*^3^-C_3_H_5_)Cl]_2_ (1.9 mg, 0.005 mmol), S-IPr·HCl (4.2 mg, 0.005 mmol) and toluene (1.0 ml) were mixed, followed by addition of *t*-BuOK (1.0 M in THF, 25 μl, 0.025 mmol) at 0 °C. The resulting mixture was stirred at room temperature for 30 min, then added to the ketone solution. The allylic substrate **2** (0.2 mmol) and toluene (1.0 ml) was then added and the mixture was stirred at corresponding temperature. After the reaction was complete, the reaction mixture was quenched by H_2_O (0.5 ml). The solution was dried (anhydrous Na_2_SO_4_) and then filtered through a 0.5-inch plug of silica gel (eluting with EtOAc) to remove the solid. The crude reaction mixture was concentrated under reduced pressure. CDCl_3_ (0.7–0.8 ml) was added to dissolve the crude reaction mixture, and mesitylene (23 μl) was added as an internal standard. The regio and diastereoselectivity was then determined by ^1^H NMR spectroscopy. After this analysis, the crude reaction mixture was purified by flash column silica gel chromatography (eluting with petroleum ether/toluene 1/1 or petroleum ether/ethyl acetate 10/1) to afford the product **3**. For additional procedures see Supplementary Methods.

### Data availability

The authors declare that the data supporting the findings of this study are available within the article (and Supplementary Information files), and also are available from the corresponding author on request.

## Additional information

**Accession codes**: The X-ray crystallographic coordinates for the structures reported in this article have been deposited at the Cambridge Crystallographic Data Centre (CCDC), under deposition number CCDC 1457162 (for **3c**), 1457163 (for **3f**), 1457165 (for **7**) and 1457166 (for **8**). These data can be obtained free of charge from the Cambridge Crystallographic Data Centre via http://www.ccdc.cam.ac.uk/data_request/cif.

**How to cite this article:** Bai, D.-C. *et al*. Palladium/N-heterocyclic carbene catalysed regio and diastereoselective reaction of ketones with allyl reagents via inner-sphere mechanism. *Nat. Commun.* 7:11806 doi: 10.1038/ncomms11806 (2016).

## Supplementary Material

Supplementary Information Supplementary Figures 1-73, Supplementary Tables 1-8, Supplementary Methods and Supplementary References

Supplementary Data 1 Energies and coordinates of calculated structures

## Figures and Tables

**Figure 1:**
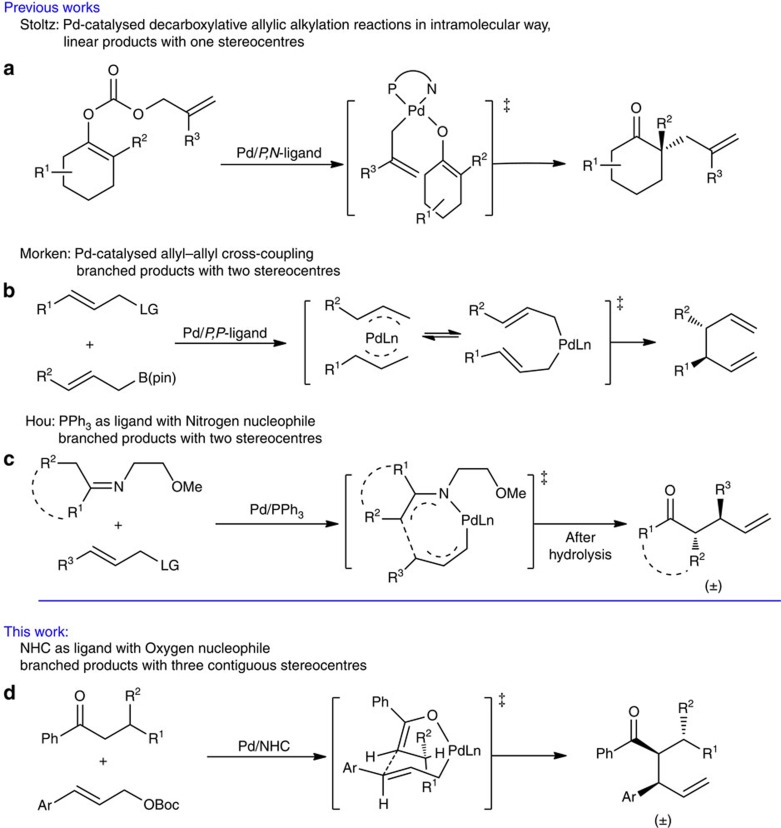
Examples of Pd-catalysed allylic alkylation following an inner-sphere mechanism. (**a**) Pd-Catalysed intramolecular decarboxylative allylic alkylation. (**b**) Pd-catalysed allyl–allyl cross-coupling reaction, branched products with two stereocentres were given. (**c**) Pd-catalysed allylic alkylation with nitrogen as nucleophile affording branched products with two stereocentres. (**d**) Pd-catalysed allylic alkylation with oxygen as nucleophile affording branched products with three contiguous stereocentres.

**Table 1:** Influences of reaction parametes on Pd-catalysed reaction of ketone **1a**–**c** with allyl substrate **2a** *.

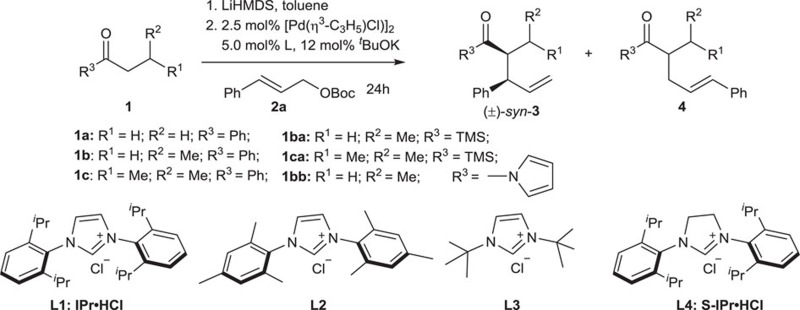						
**Entries**	**1**	**L**	**T (^o^C)**	**3/4**†	***dr***†	**Yield (%)**‡
1	**1a**	**L1**	50	96/4	20/80	95 (**3a**)
2	**1b**	**L1**	50	95/5	30/70	98 (**3b**)
3	**1c**	**L1**	50	95/5	85/15	83 (**3c**)
4	**1c**	**L2**	50	15/85	—	32 (**3c**)
5	**1c**	**L3**	50	<5/95	—	33 (**4c**)
6	**1c**	**L1**	RT	92/8	92/8	50 (**3c**)
7	**1c**	**L4**	RT	99/1	92/8	98 (**3c**)
8	**1ba**	**L4**	30	91/9	7/93	72 (**3ba**)
9§	**1ca**	**L4**	30	91/9	70/30	70 (**3ca**)
10	**1bb**	**L4**	30	—	—	NR

NR, no reaction; RT, room temperature; T, temperature.

^*^Reaction conditions: **1**/LiHMDS/**2a**/[Pd(*η*^3^-C_3_H_5_)Cl]_2_/ligand=200/200/100/2.5/5; 0.1 M of ketone **1**.

^†^Determined by ^1^H NMR, *dr* is the ratio of *syn*-**3**/*anti*-**3**.

^‡^Isolated yield.

^§^The configuration for major component of **3ca** is not determined.

**Table 2:** Reaction of ketone **1d**–**I** with allyl substrate **2a** *.

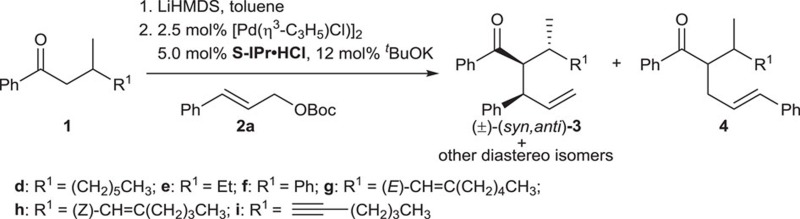				
**Entries**	**1**	**3/4**†	***dr***†	**Yield (%)**‡
1	**1d**	95/5	57/43	56 (**3d**)
2	**1e**	98/2	60/40	98 (**3e**)
3	**1f**	95/5	86/14	98 (**3f**)
4	**1g**	93/7	77/23	92 (**3g**)
5	**1h**	88/12	86/14	56 (**3h**)
6	**1i**	95/5	93/7	99 (**3i**)

^*^Reaction conditions: **1**/LiHMDS/**2a**/[Pd(*η*^3^-C_3_H_5_)Cl]_2_/S-IPr·HCl=200/200/100/2.5/5; 0.1 M of ketone **1**; 30 °C, 24 h.

^†^Determined by ^1^H NMR, *dr* is the ratio of (±)-(*syn,anti*)-**3**/other diastereo isomers.

^‡^Isolated yield.

**Table 3:** Substrate scope of the reaction*.

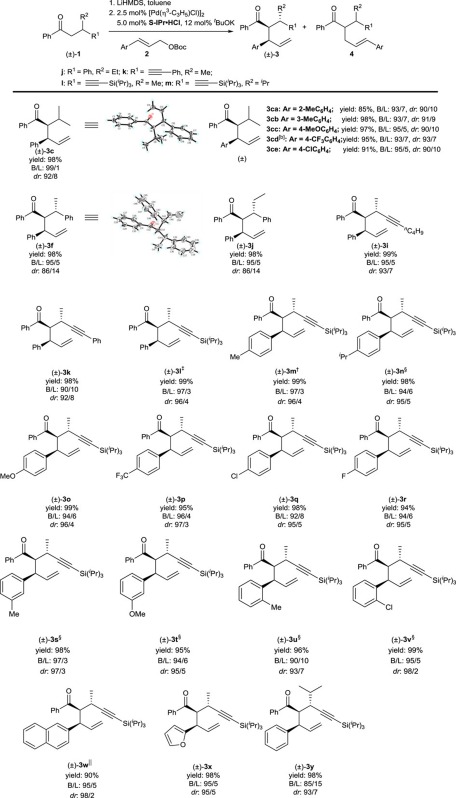

*Reaction conditions:**1**/LiHMDS/**2**/[Pd(*η*^3^-C_3_H_5_)Cl]_2_/S-IPr·HCl=200/200/100/2.5/5; 0.1 M of ketone **1**; T=30^o^C; B/L and *dr* was determined by ^1^H NMR, *dr* is the ratio of (±)-(*syn,anti*)-**3**/other diastereoisomers; Isolated yield. †T=50 ^o^C. ‡Solvent=THF. §OBoc of **2** was replaced with OP(OEt)_2_. ||The yield was determined by ^1^H NMR.
