# Supplementary material for: Palladium/N-heterocyclic carbene catalysed regio and diastereoselective reaction of ketones with allyl reagents via inner-sphere mechanism
Source: Nat Commun. 2016 Jun 10;7:11806. doi: 10.1038/ncomms11806 (PMC4906412; doi:10.1038/ncomms11806)
Supplement: Supplementary Information — Supplementary Figures 1-73, Supplementary Tables 1-8, Supplementary Methods and Supplementary References [file ncomms11806-s1.pdf]

Supplementary Figures

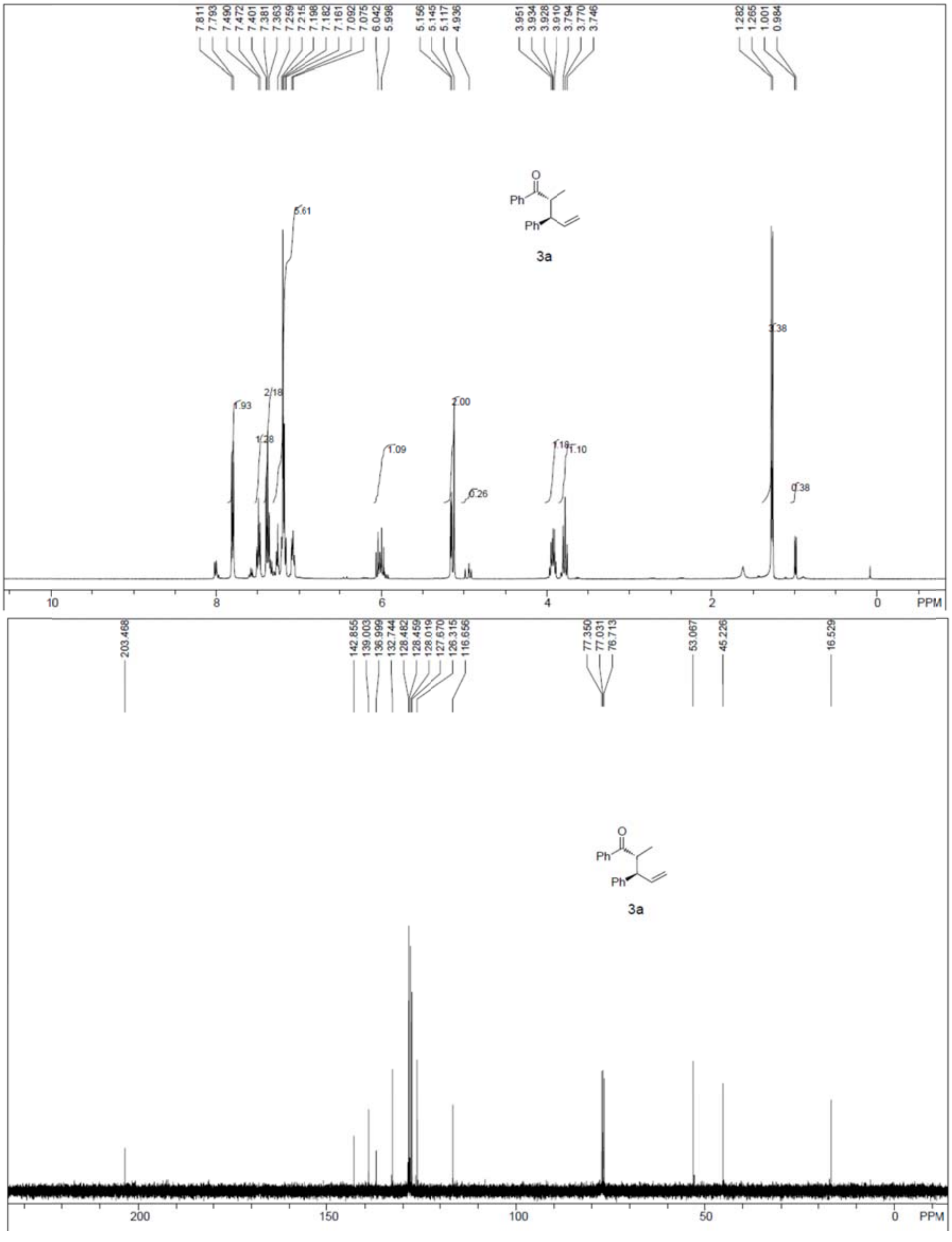

Supplementary Figure 1. ^{1}H and ^{13}C NMR spectrum for 3a.

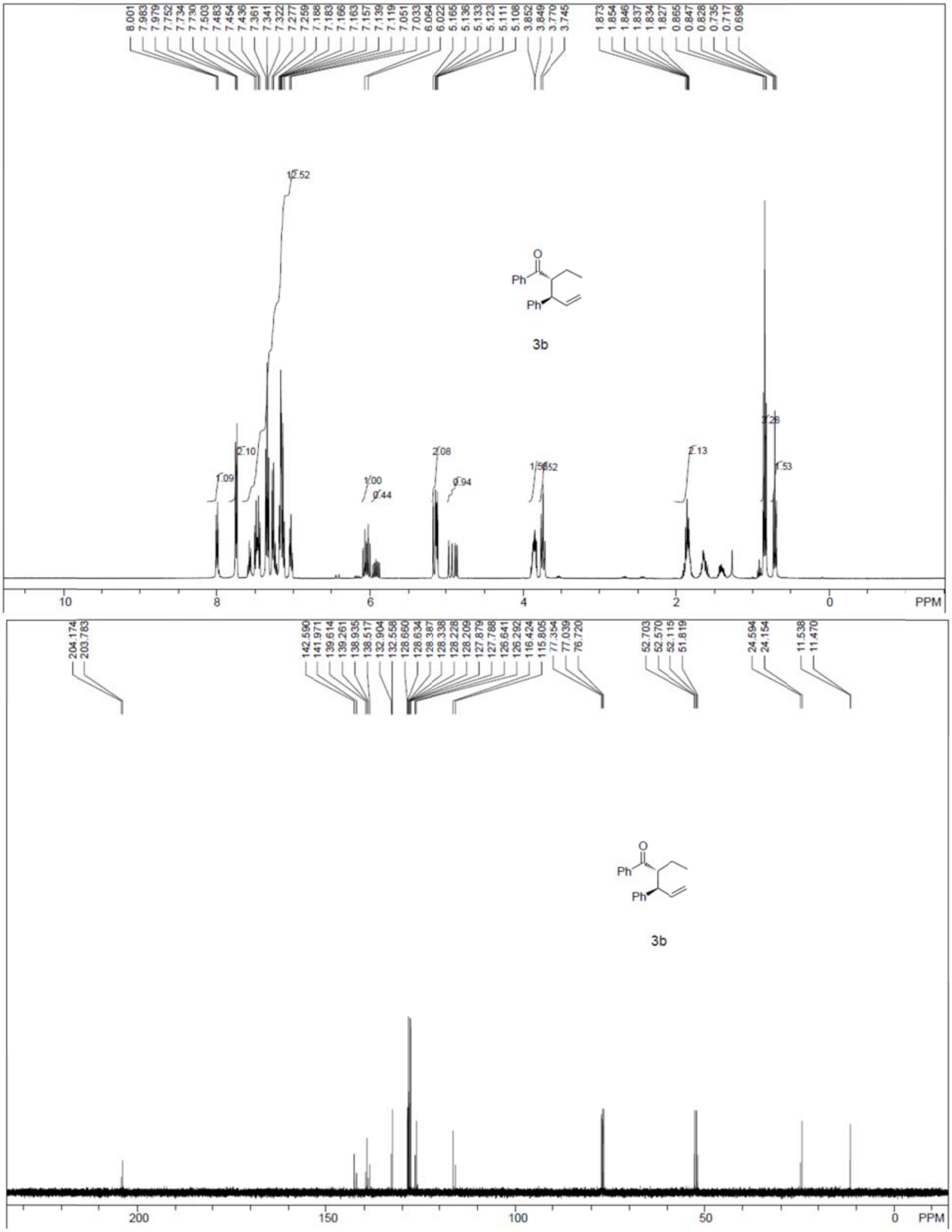

Supplementary Figure 2. ^{1}H and ^{13}C NMR spectrum for 3b.

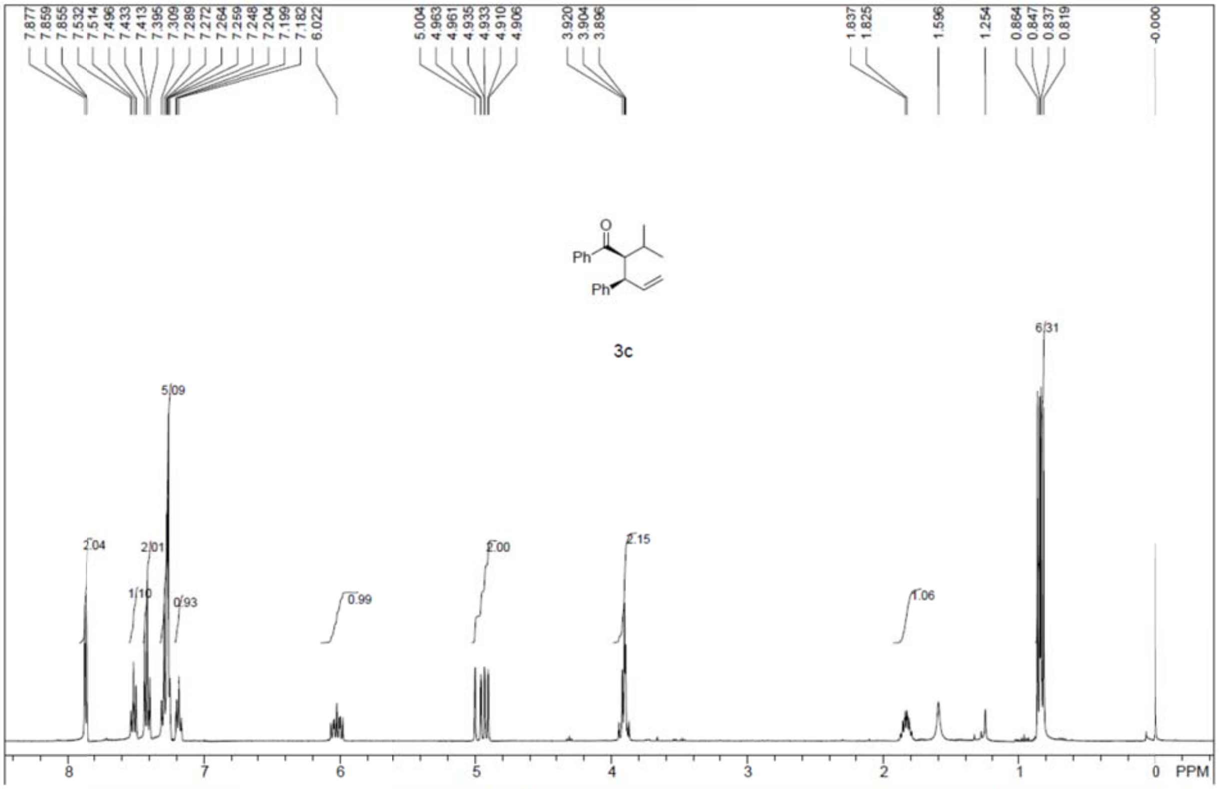

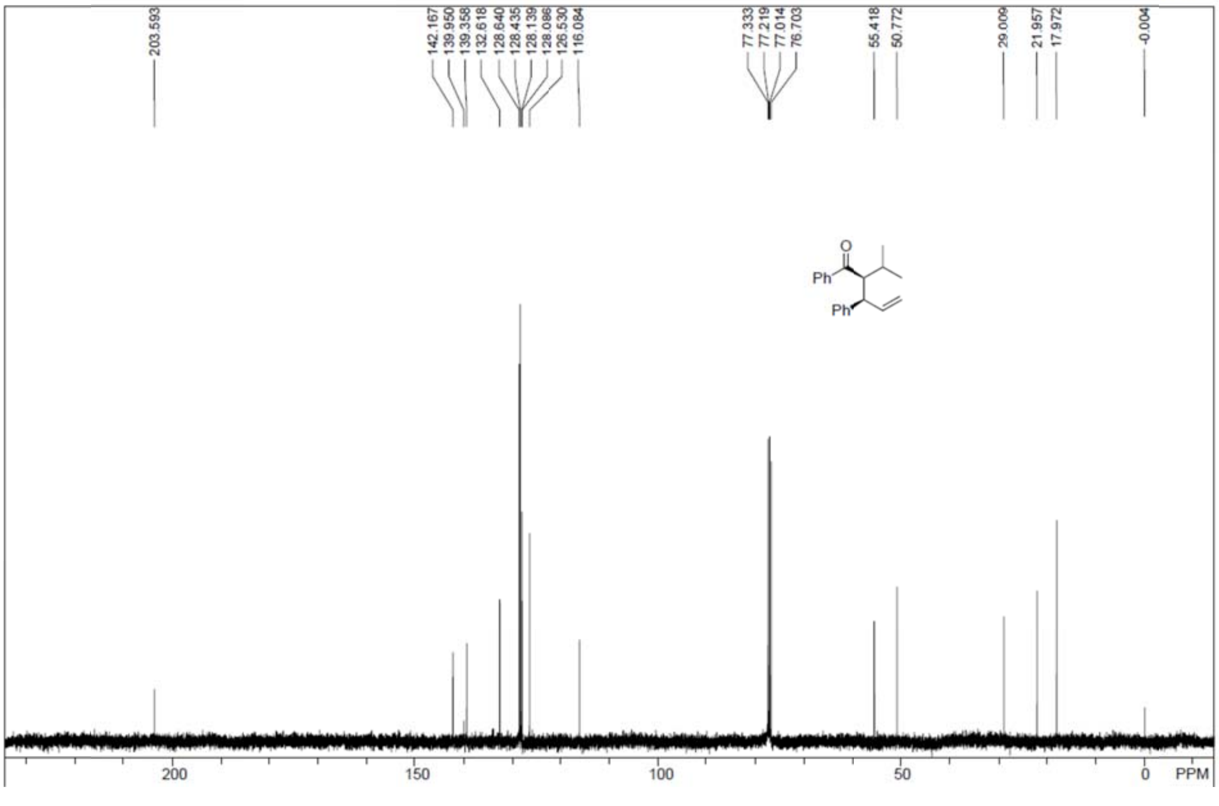

Supplementary Figure 3. ^{1}H and ^{13}C NMR spectrum for 3c.

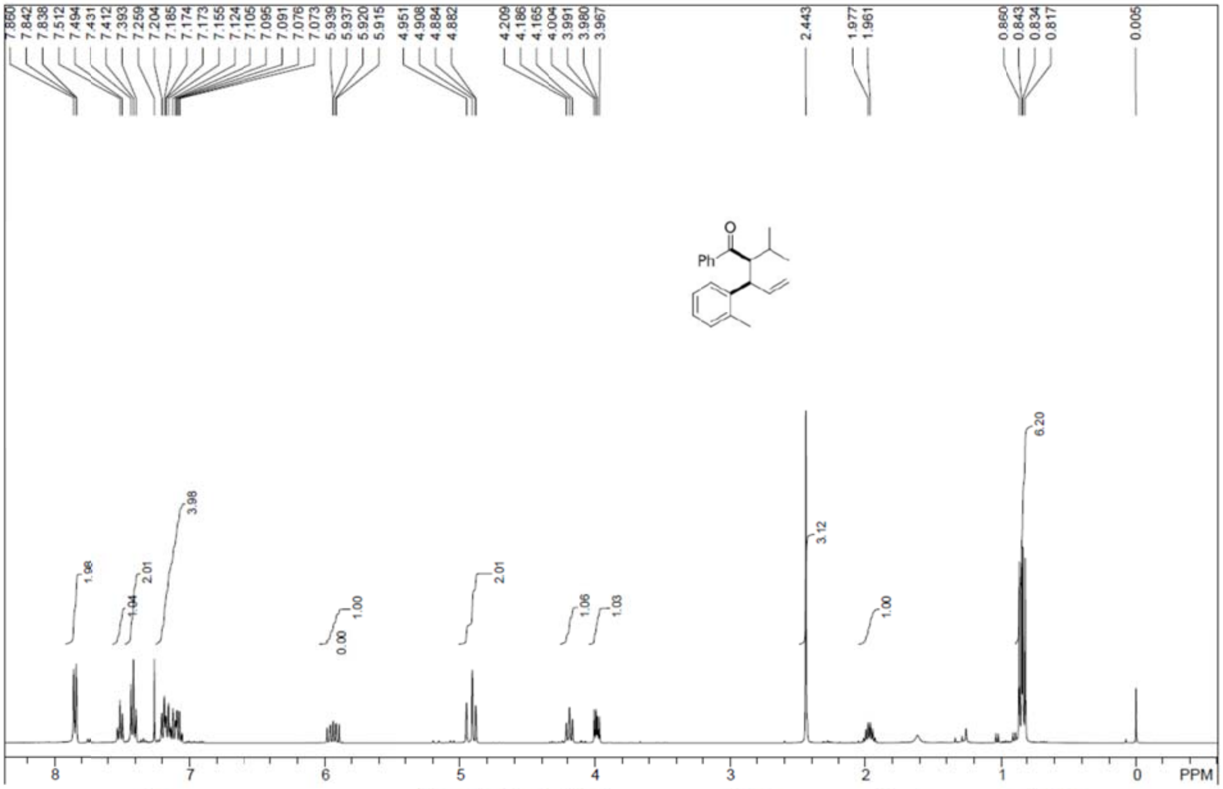

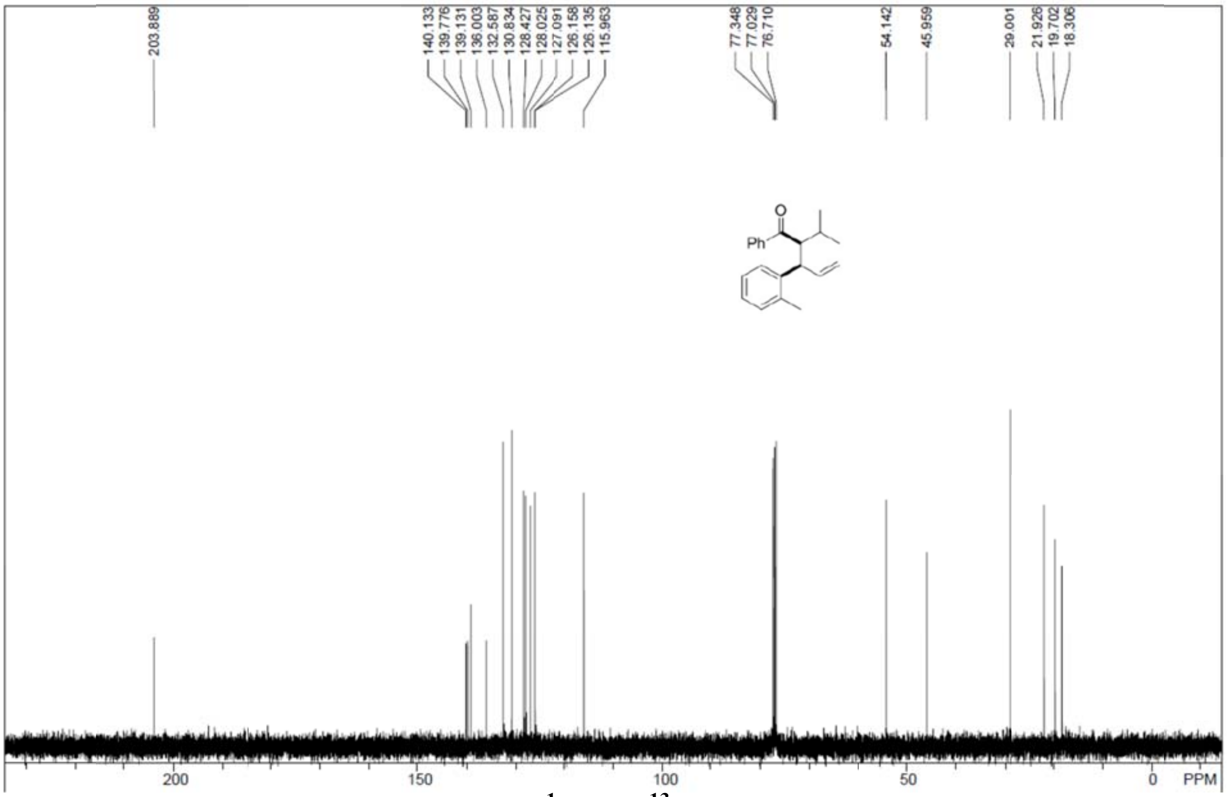

Supplementary Figure 4. ^{1}H and ^{13}C NMR spectrum for 3ca.

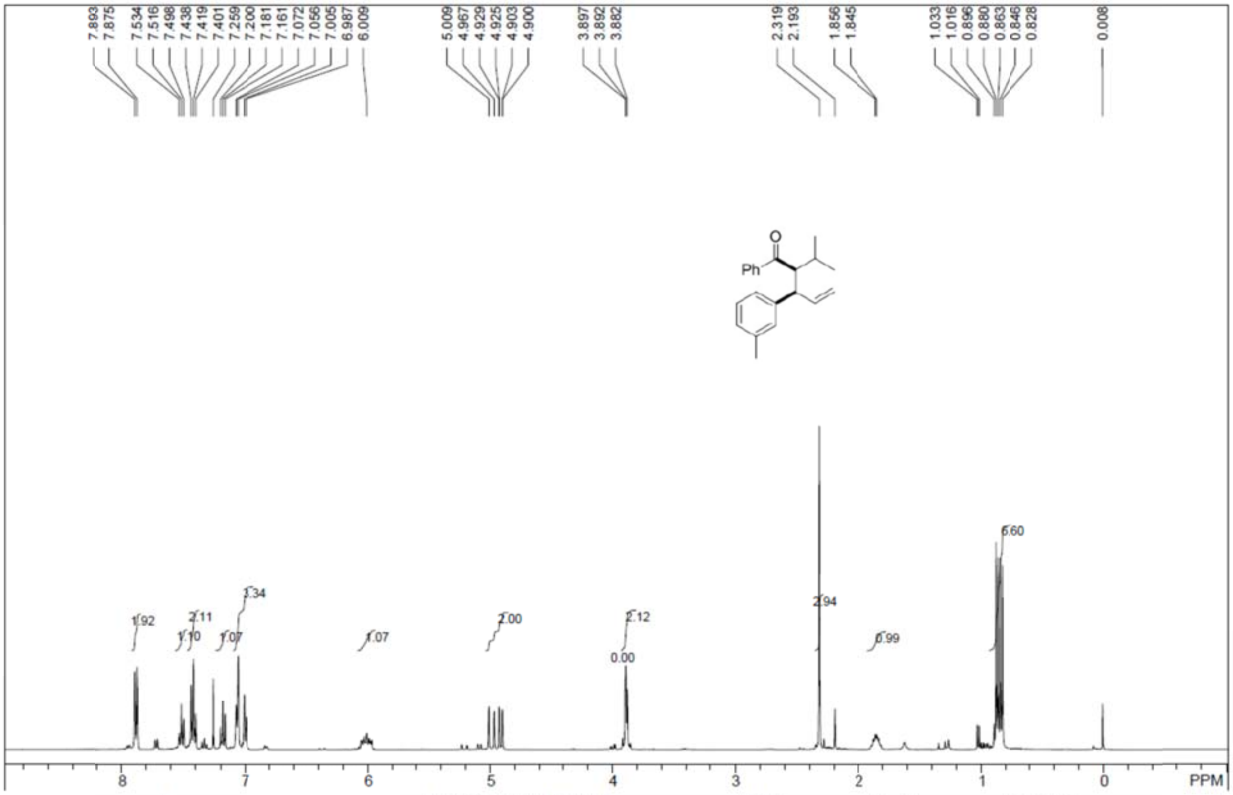

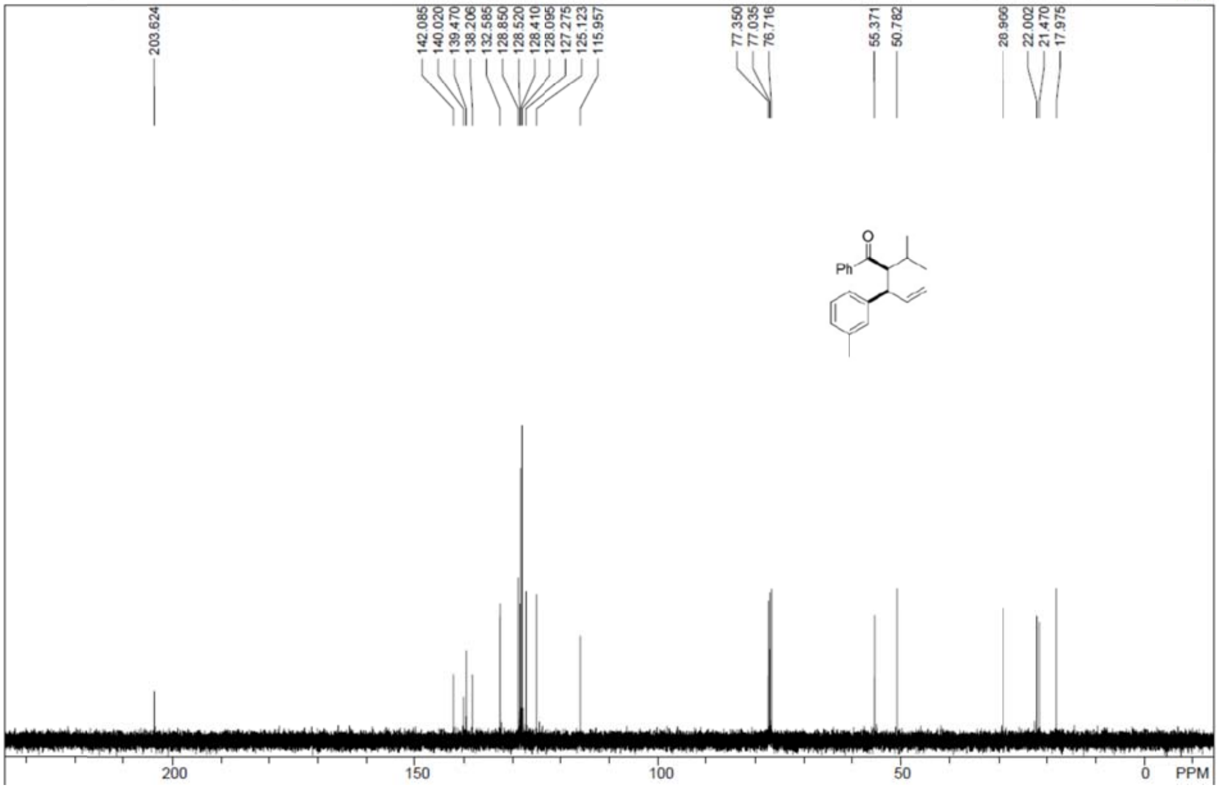

Supplementary Figure 5. ^{1}H and ^{13}C NMR spectrum for 3cb.

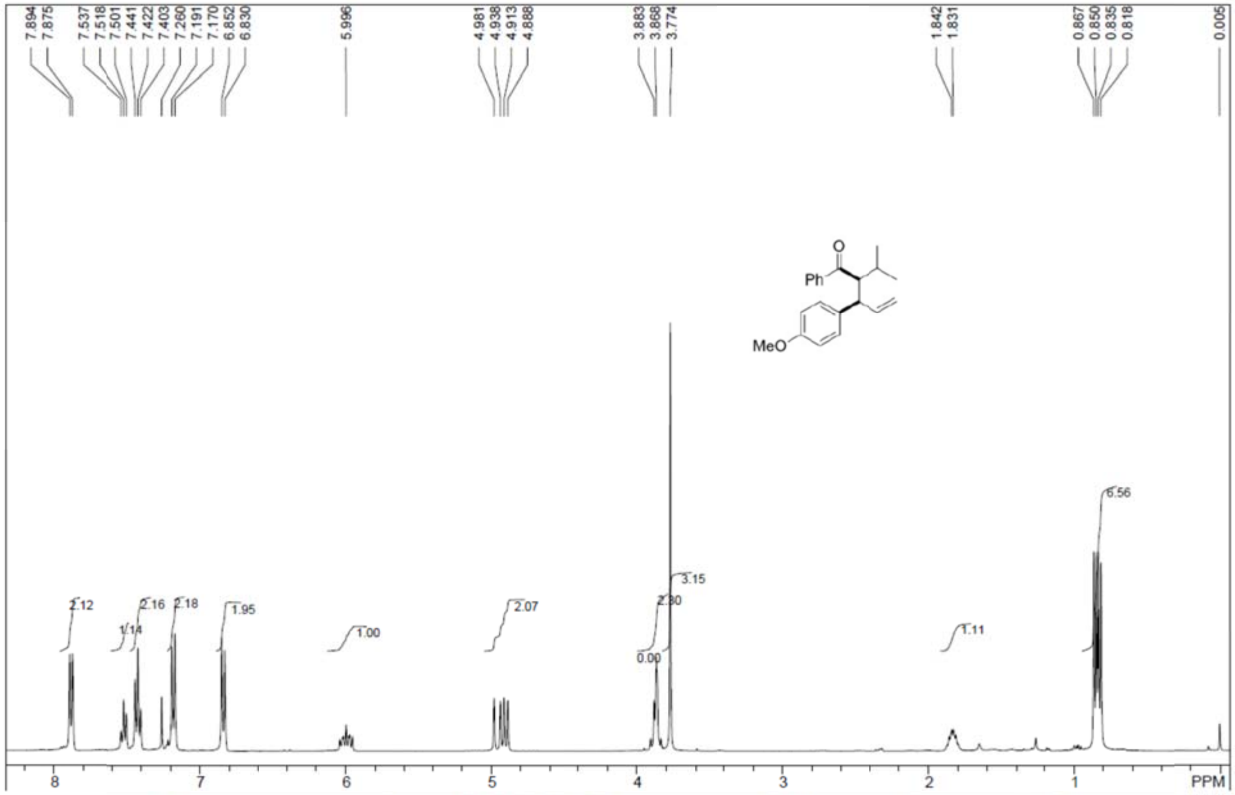

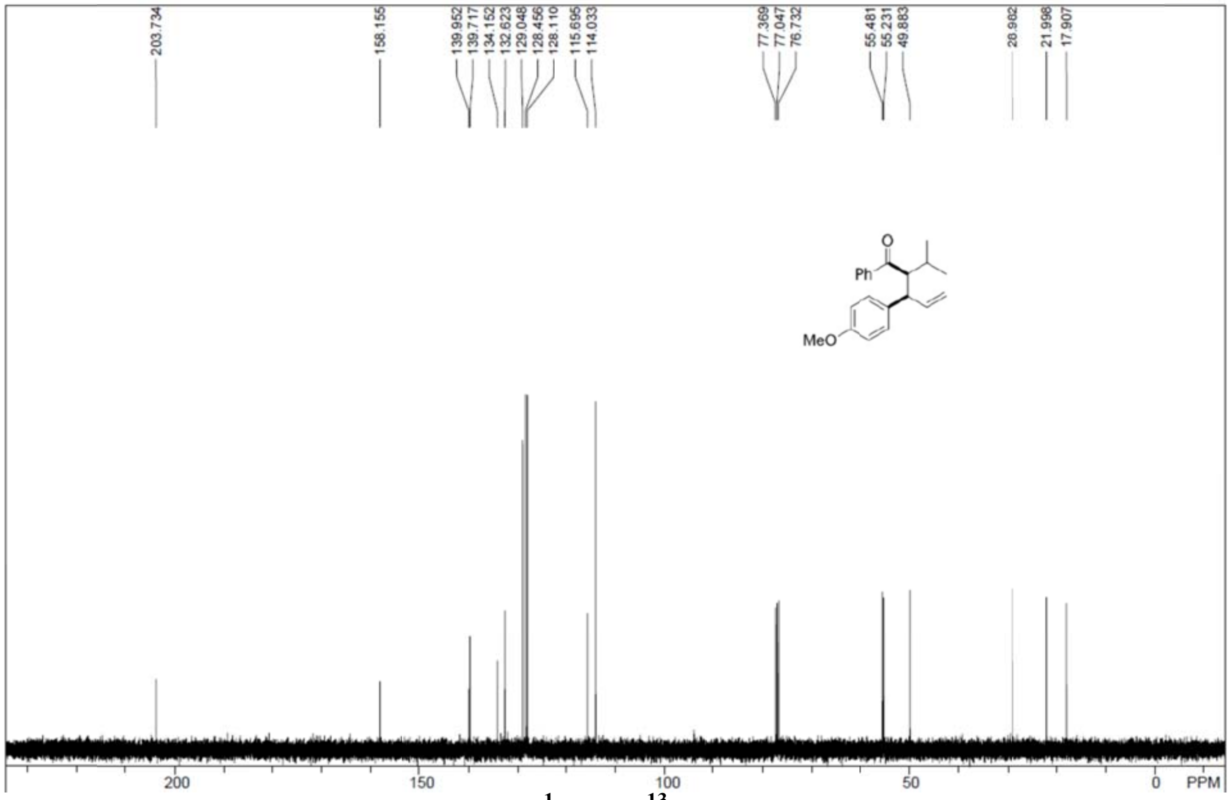

Supplementary Figure 6. ^{1}H and ^{13}C NMR spectrum for 3cc.

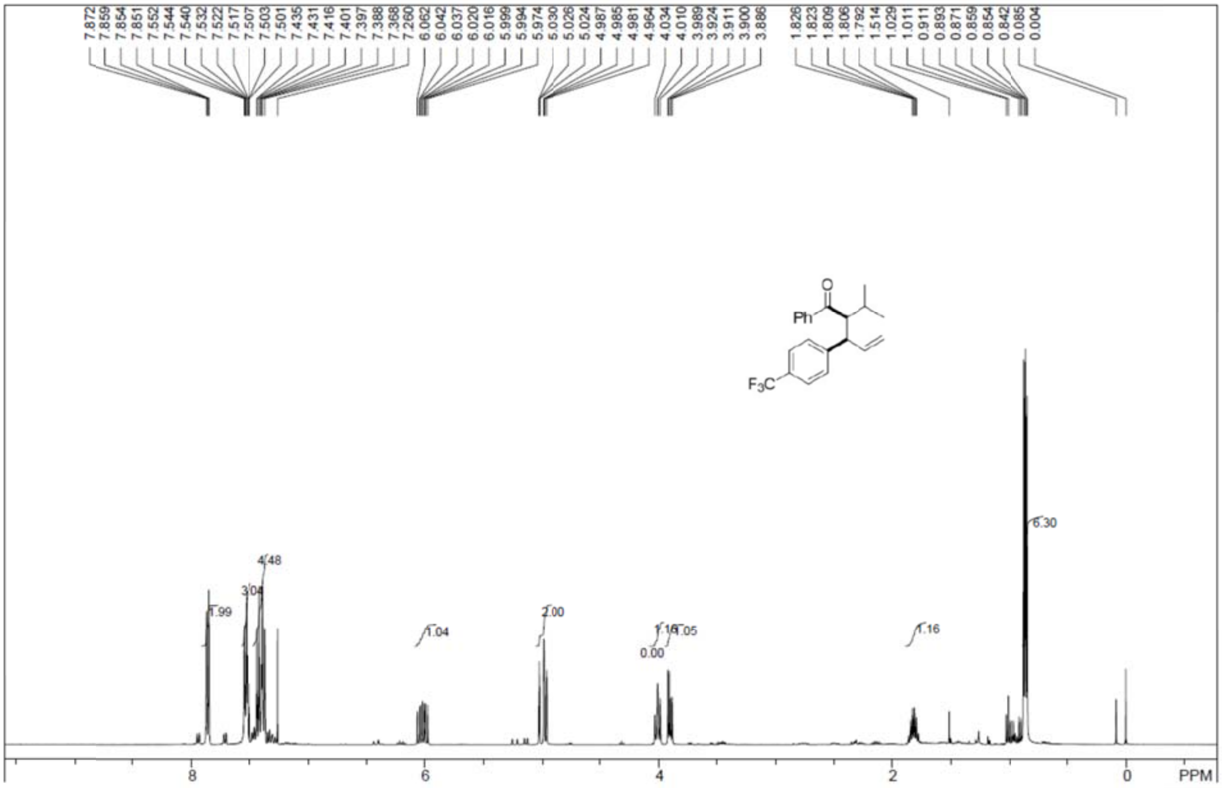

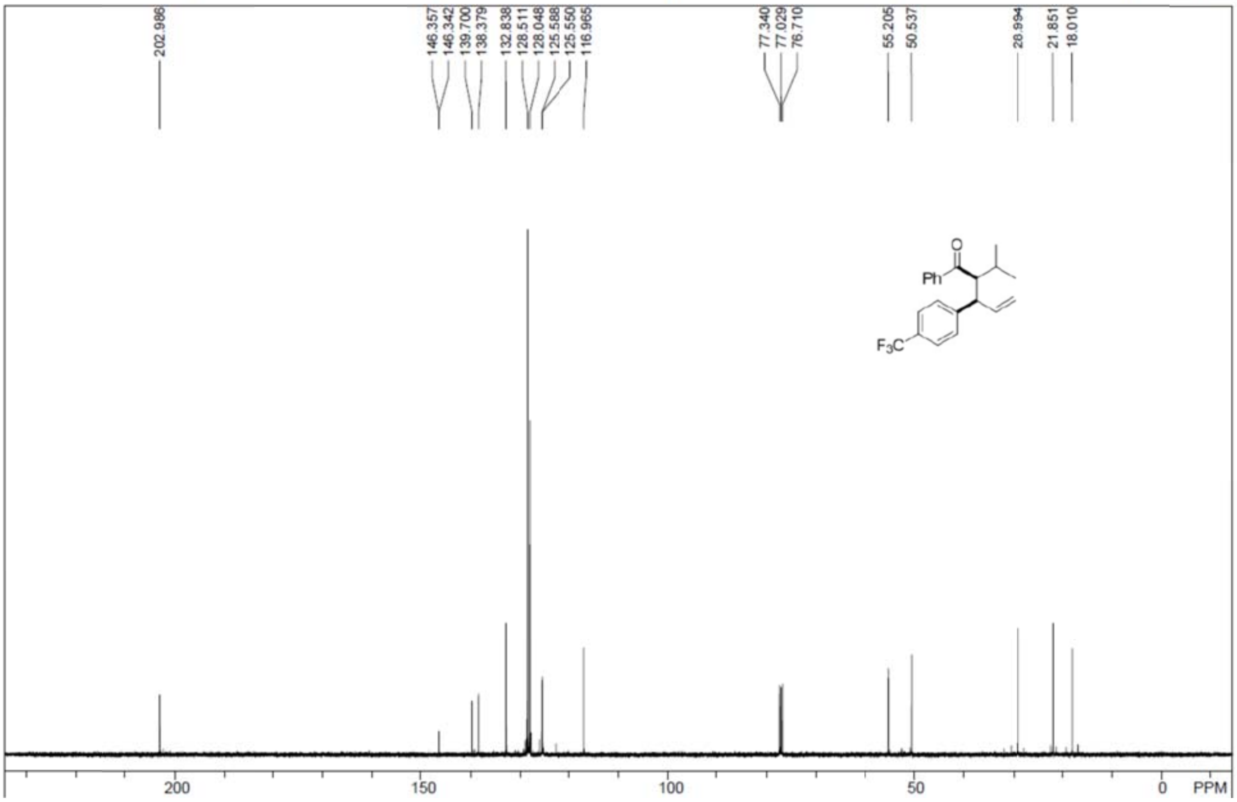

Supplementary Figure 7. ^{1}H and ^{13}C NMR spectrum for 3cd.

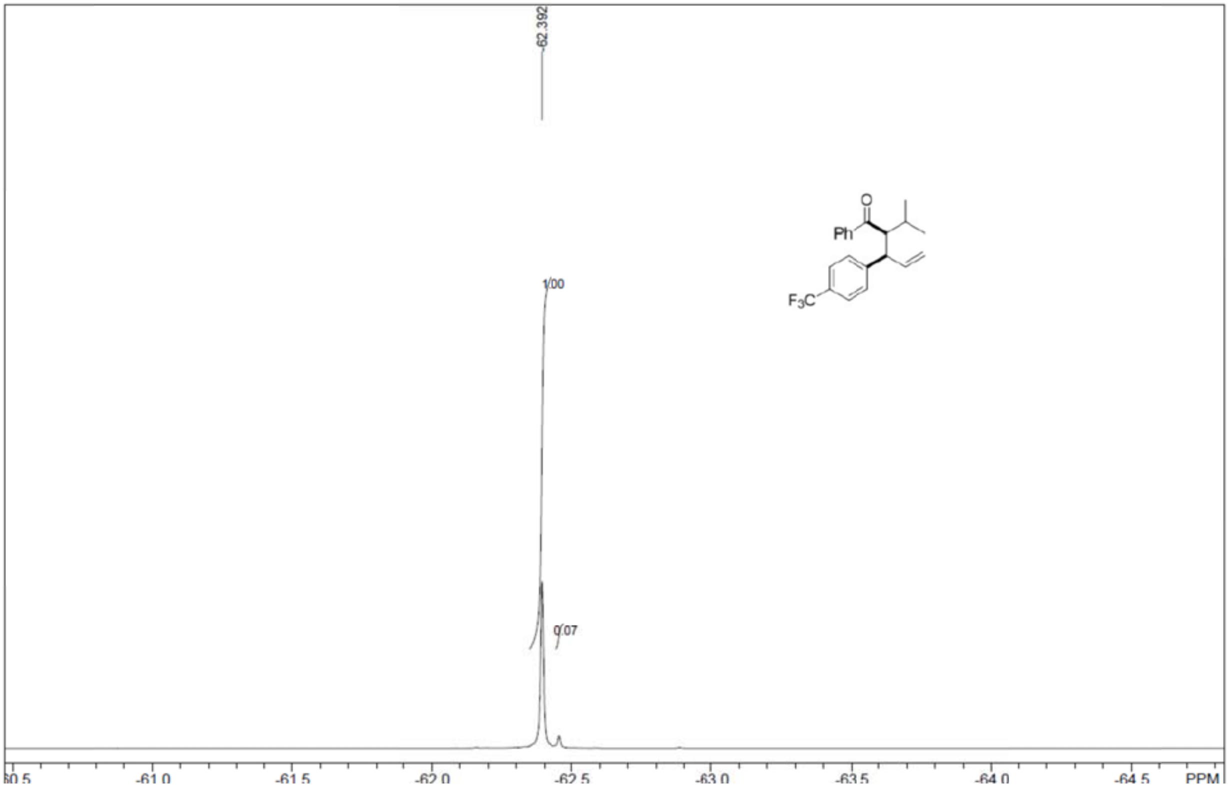

Supplementary Figure 8. ^{19}F NMR spectrum for 3cd.

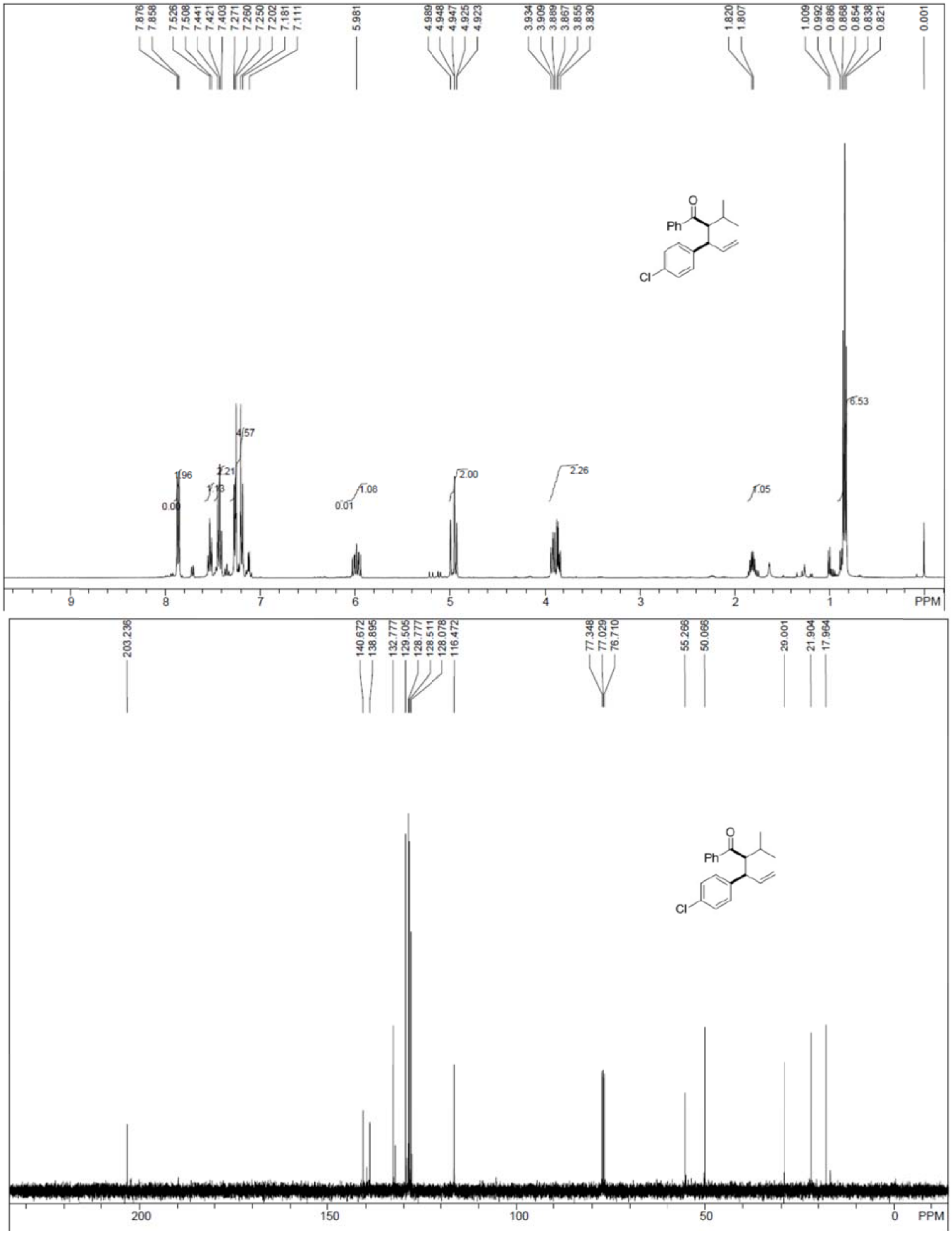

Supplementary Figure 9. ^{1}H and ^{13}C NMR spectrum for 3ce.

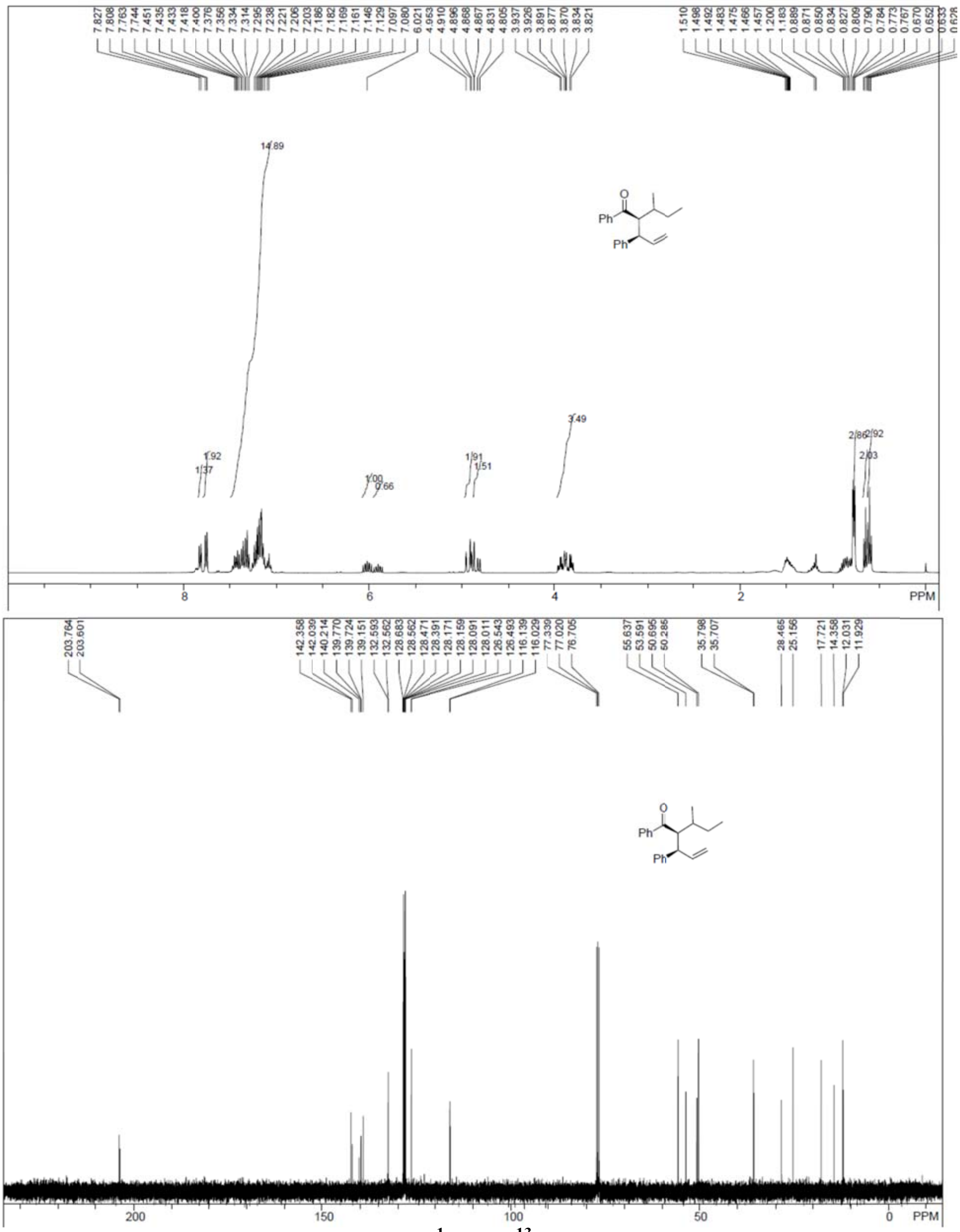

Supplementary Figure 10. ^{1}H and ^{13}C NMR spectrum for 3e.

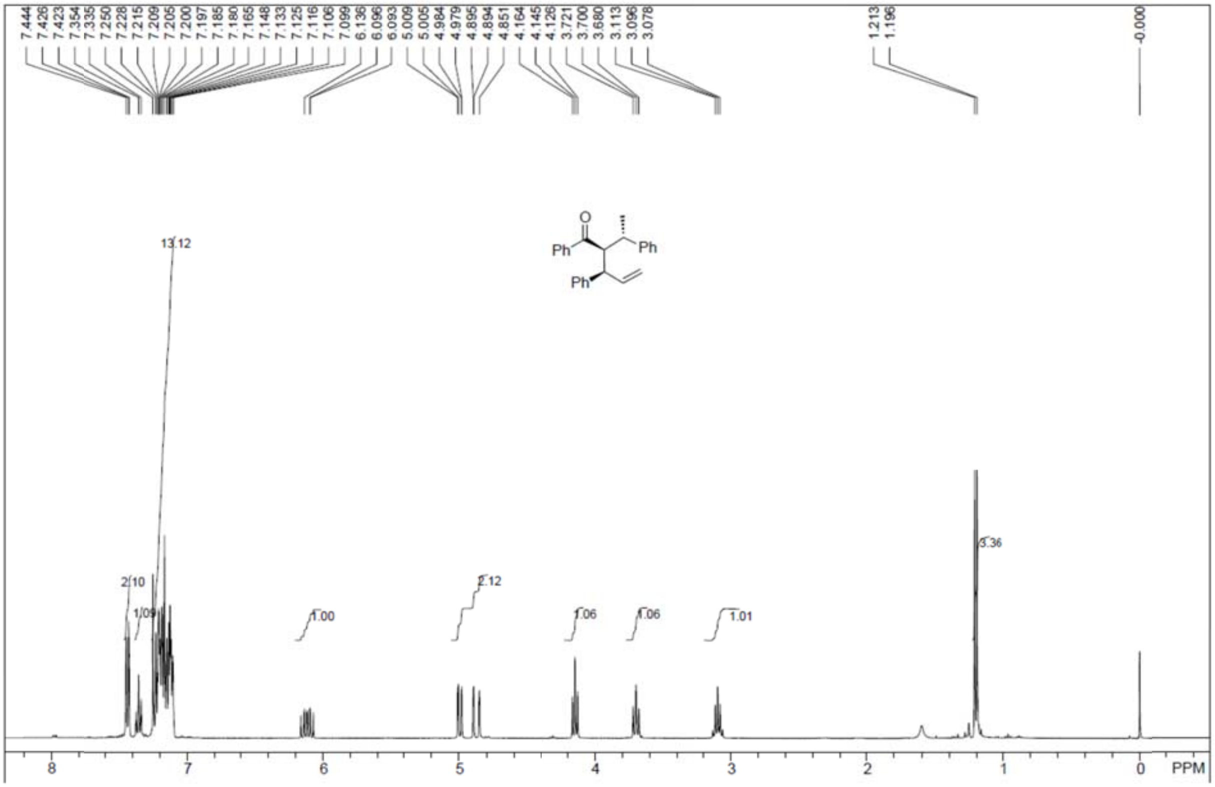

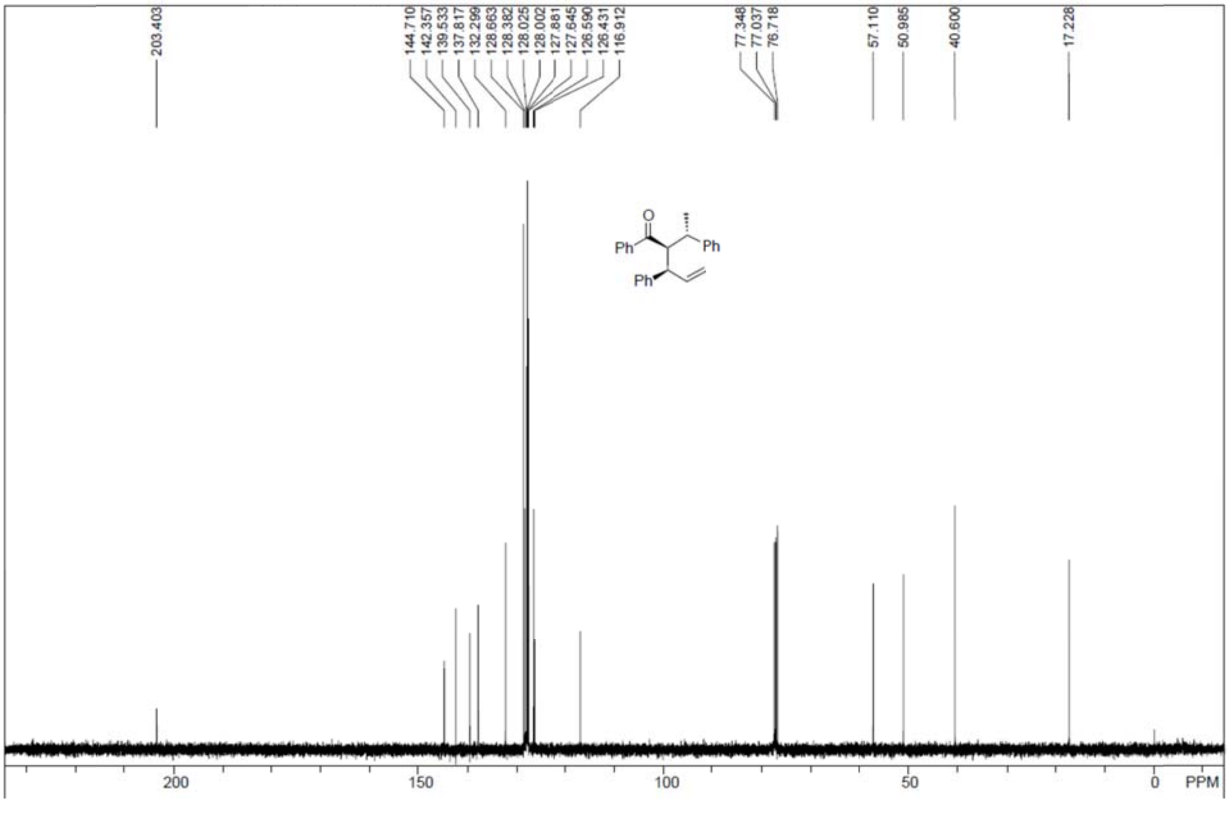

Supplementary Figure 11. ^{1}H and ^{13}C NMR spectrum for 3f

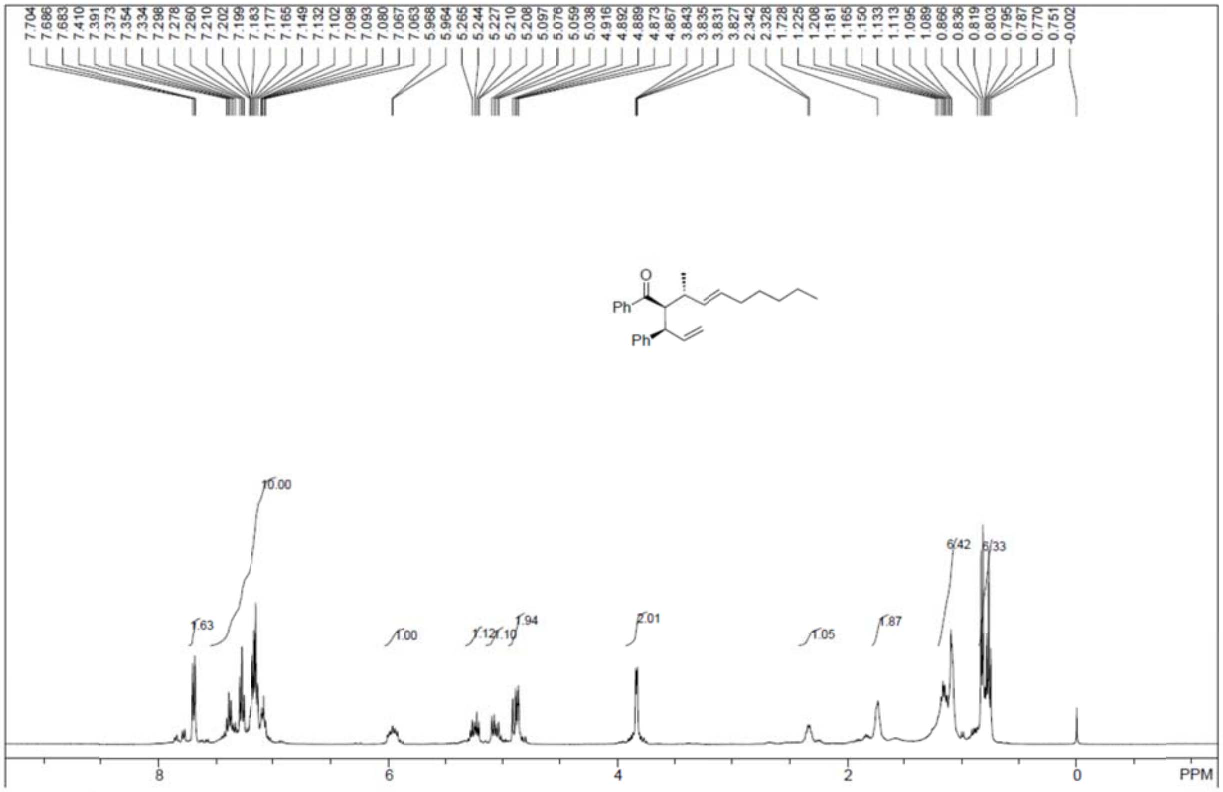

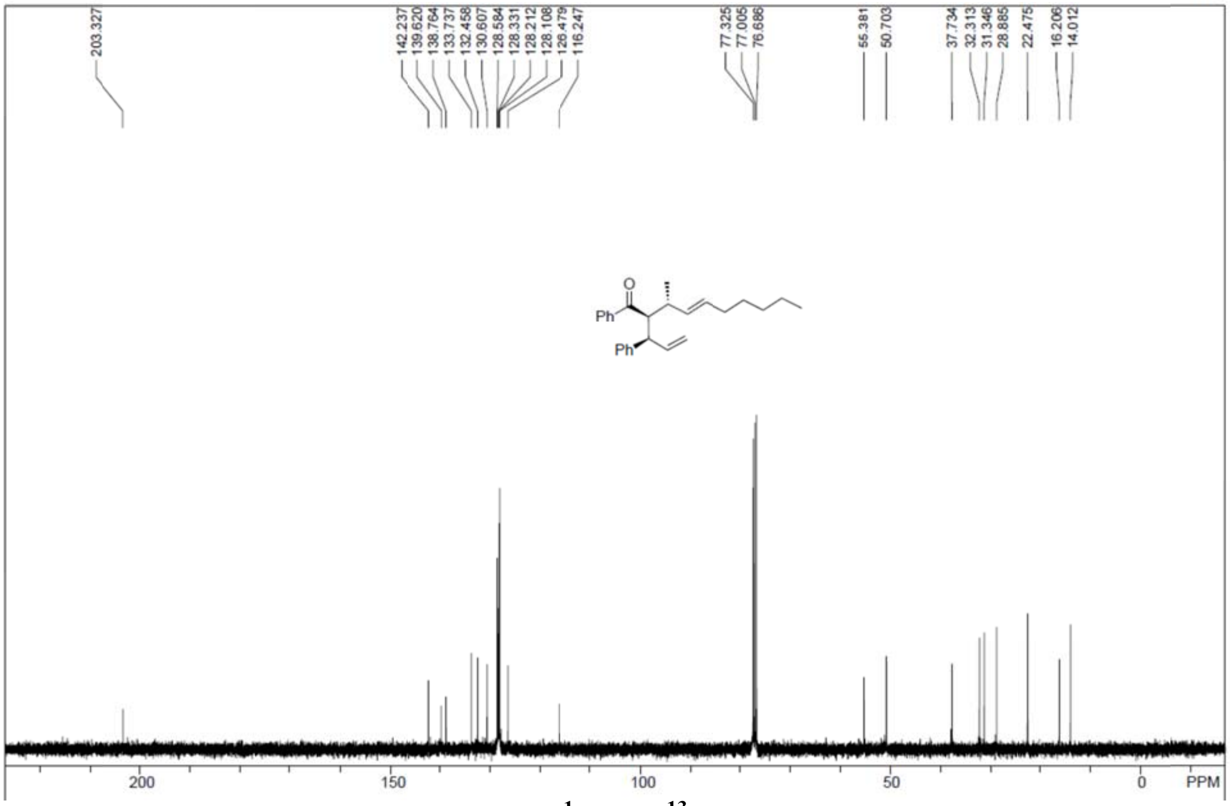

Supplementary Figure 12. ^{1}H and ^{13}C NMR spectrum for 3g.

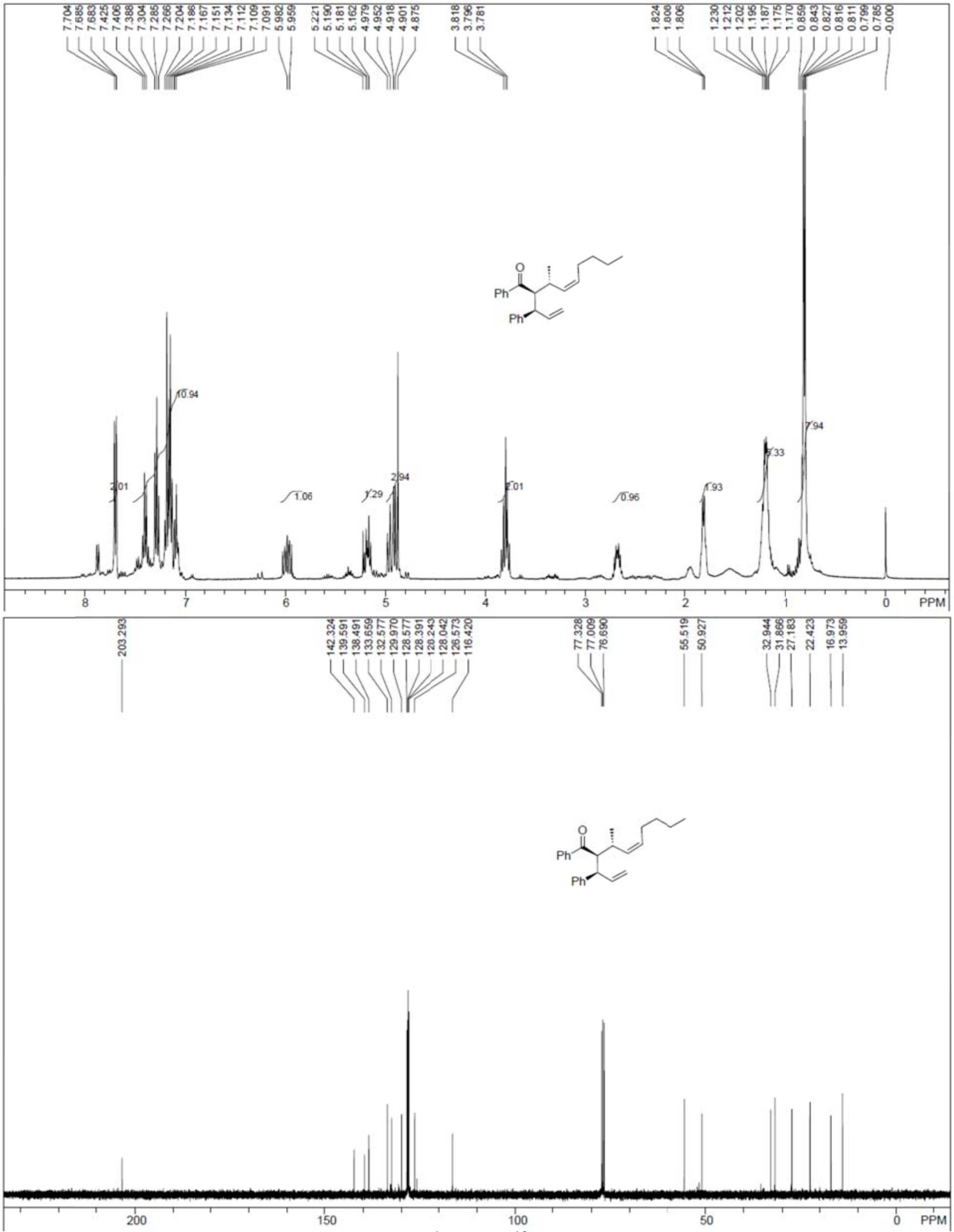

Supplementary Figure 13. ^{1}H and ^{13}C NMR spectrum for 3h.

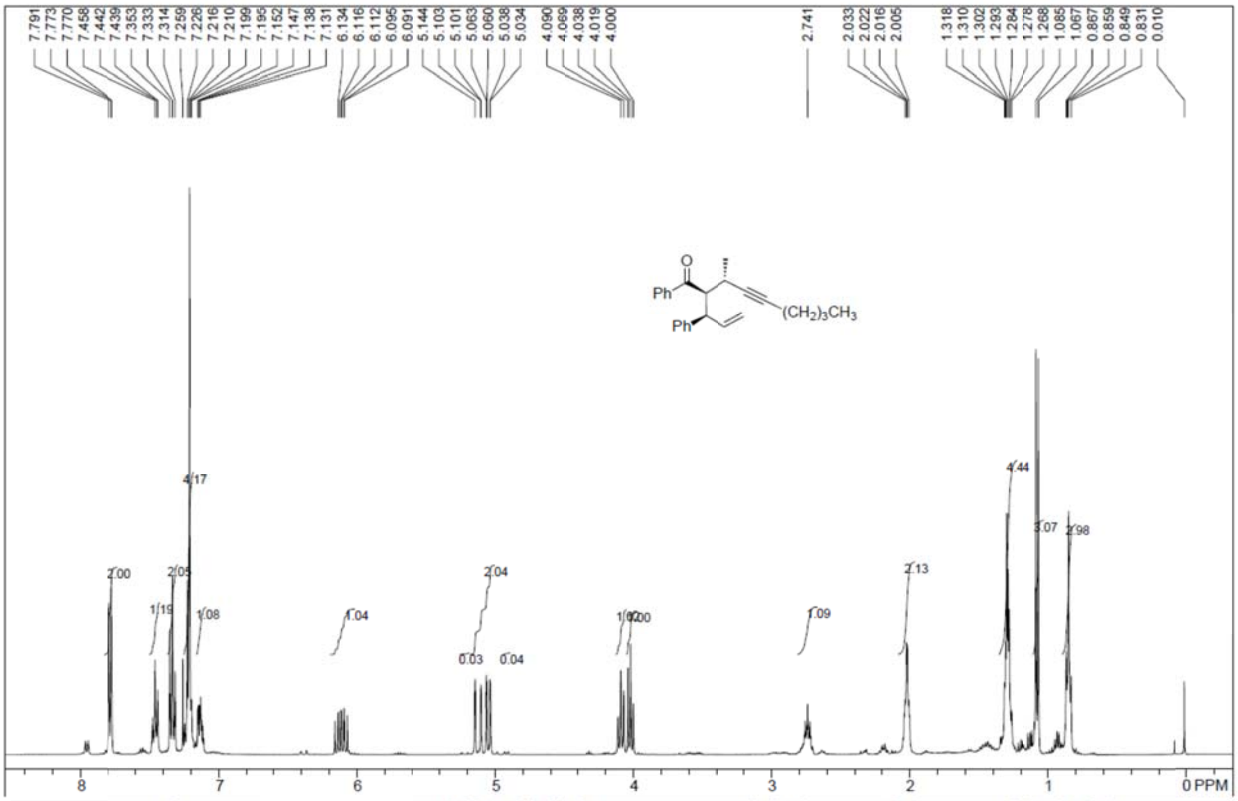

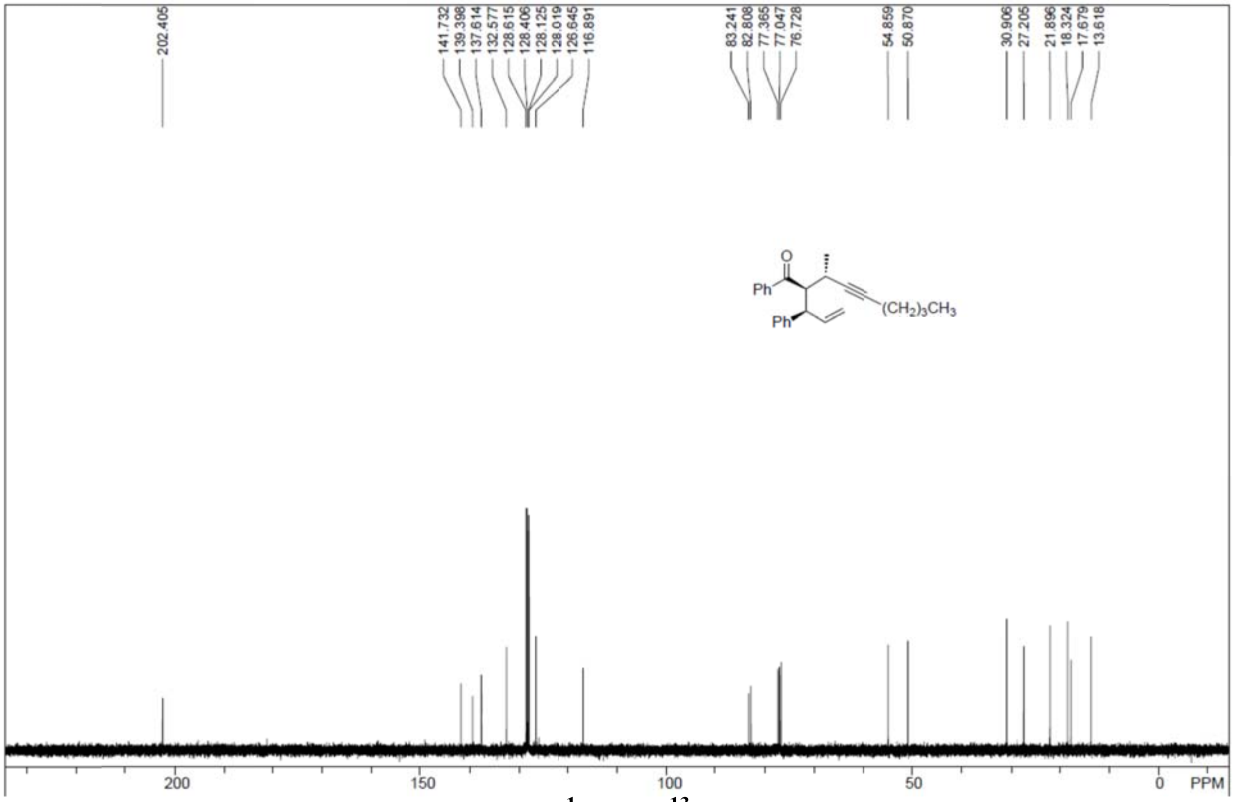

Supplementary Figure 14. ^{1}H and ^{13}C NMR spectrum for 3i.

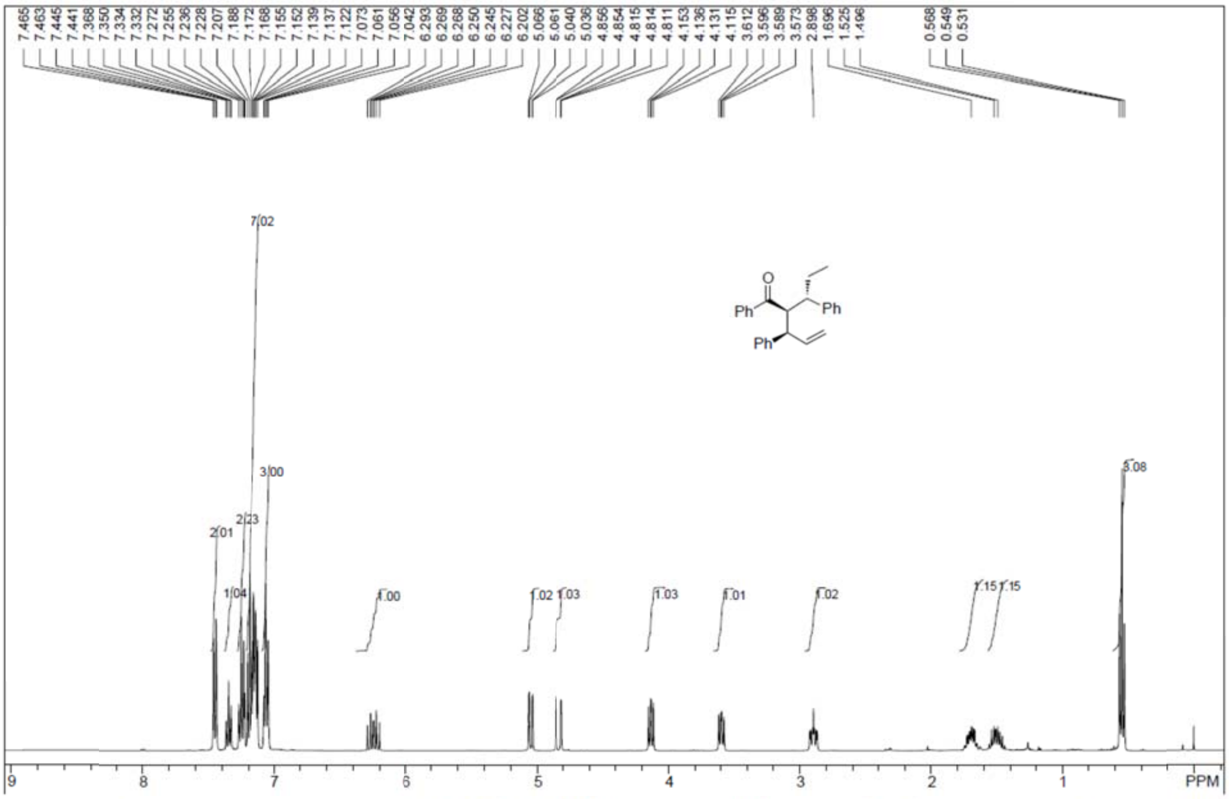

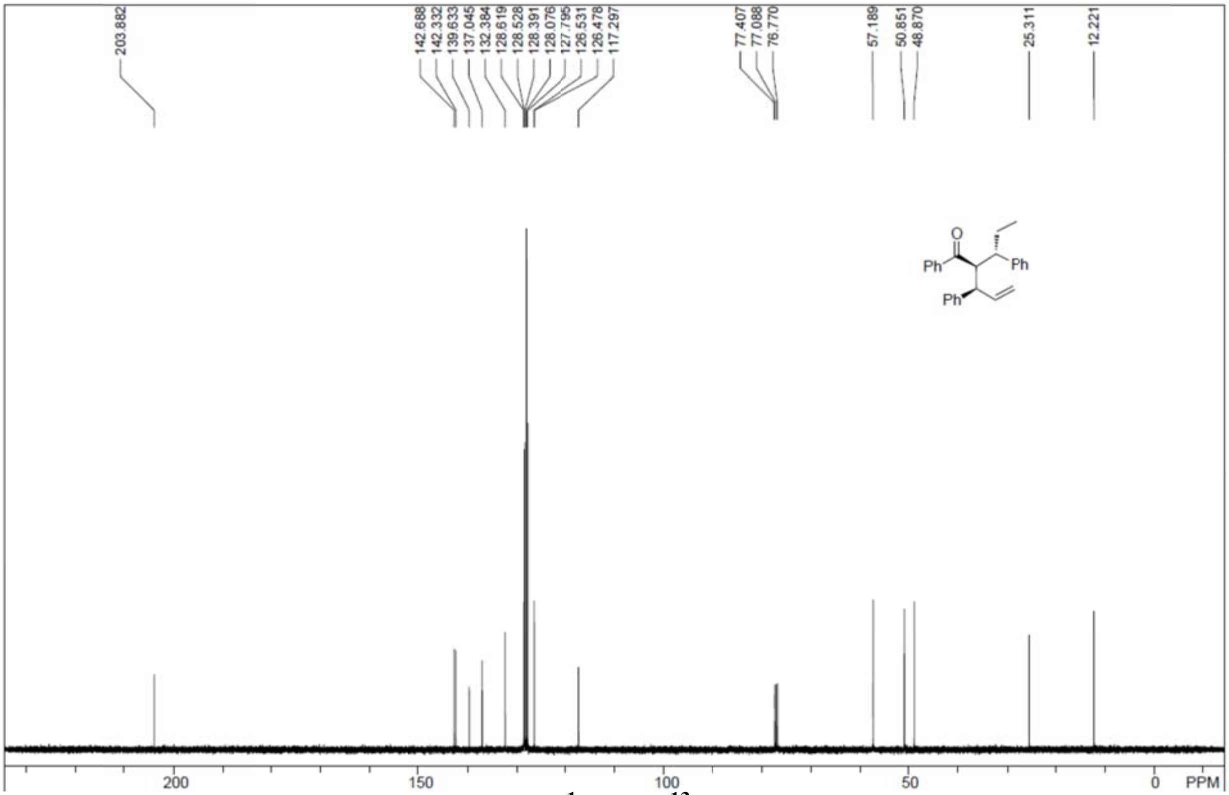

Supplementary Figure 15. ^{1}H and ^{13}C NMR spectrum for 3j.

==

Acq. Operator : [redacted]

Sample Operator : [redacted]

Acq. Instrument : SFC   Location : Vial 12

Injection Date : 1/15/2015 3:09:26 PM

Inj Volume : 5.000 µl

Acq. Method : C:\CHEM32\1\METHODS\AGILENT_SFC6.M

Last changed : 1/15/2015 2:03:31 PM by [redacted]

(modified after loading)

Analysis Method : C:\CHEM32\1\METHODS\AGILENT_SFC6.M

Last changed : 1/16/2015 9:17:39 AM by [redacted]

(modified after loading)

Additional Info : Peak(s) manually integrated

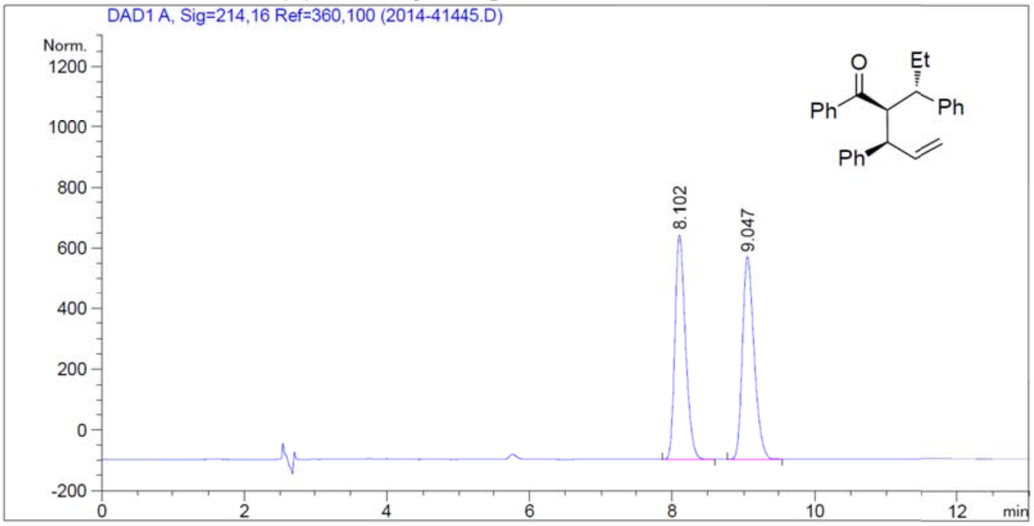

==

Area Percent Report

==

Sorted By : Signal

Multiplier : 1.0000

Dilution : 1.0000

Do not use Multiplier & Dilution Factor with ISTDs

Signal 1: DAD1 A, Sig=214,16 Ref=360,100

Peak #	RetTime [min]	Type	Width [min]	Area [mAU*s]	Height [mAU]	Area %
1	8.102	BB	0.1530	5398.75391	533.79285	49.9239
2	9.047	BB	0.1726	5415.20703	480.24991	50.0761

Totals : 1.08140e4 1014.04276

Supplementary Figure 16. HPLC spectrum for (*rac*)-3j.

Acq. Operator : [redacted]
Sample Operator : [redacted]
Acq. Instrument : SFC Location : Vial 14
Injection Date : 1/15/2015 2:48:31 PM
Inj Volume : 5.000 µl
Acq. Method : C:\CHEM32\1\METHODS\AGILENT_SFC6.M
Last changed : 1/15/2015 2:03:31 PM by [redacted]
(modified after loading)
Analysis Method : C:\CHEM32\1\METHODS\AGILENT_SFC6.M
Last changed : 1/16/2015 9:16:28 AM by [redacted]
(modified after loading)
Additional Info : Peak(s) manually integrated

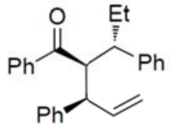

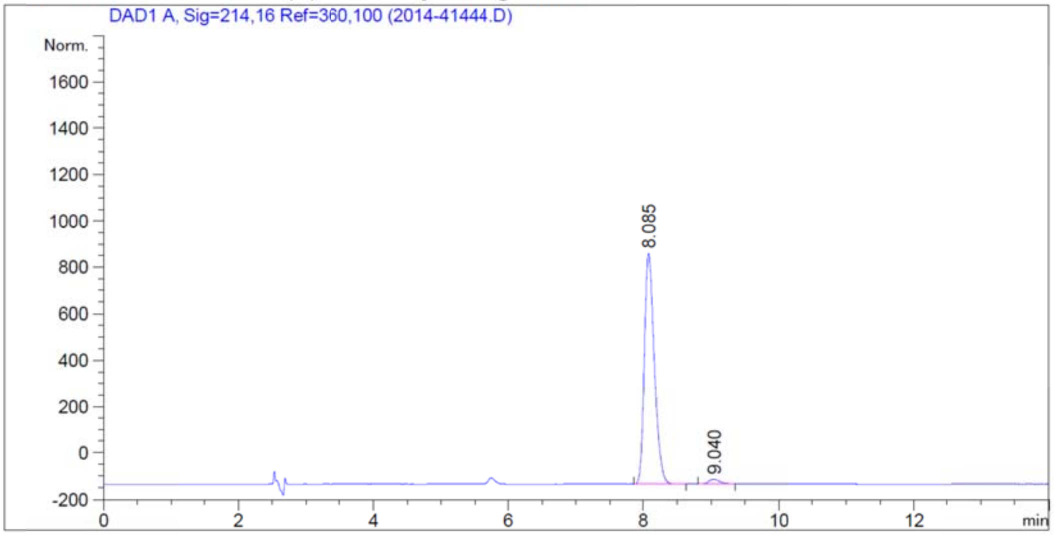

Area Percent Report

Sorted By : Signal
Multiplier : 1.0000
Dilution : 1.0000
Do not use Multiplier & Dilution Factor with ISTDs

Signal 1: DAD1 A, Sig=214,16 Ref=360,100

Peak #	RetTime [min]	Type	Width [min]	Area [mAU*s]	Height [mAU]	Area %
1	8.085	BB	0.1550	7258.21777	711.75519	97.9040
2	9.040	BB	0.1686	155.38846	14.21236	2.0960

Totals : 7413.60623 725.96755

Supplementary Figure 17. HPLC spectrum for *(3R, 4S, 5S)*-3j.

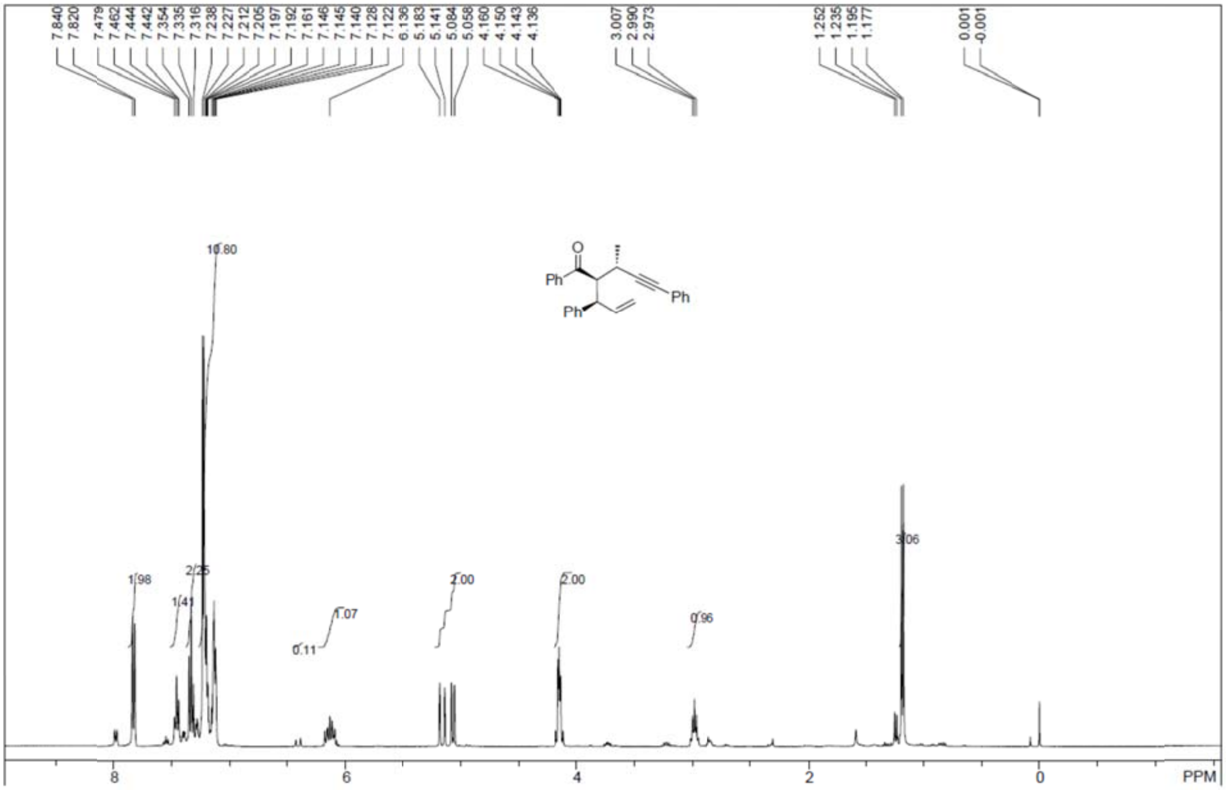

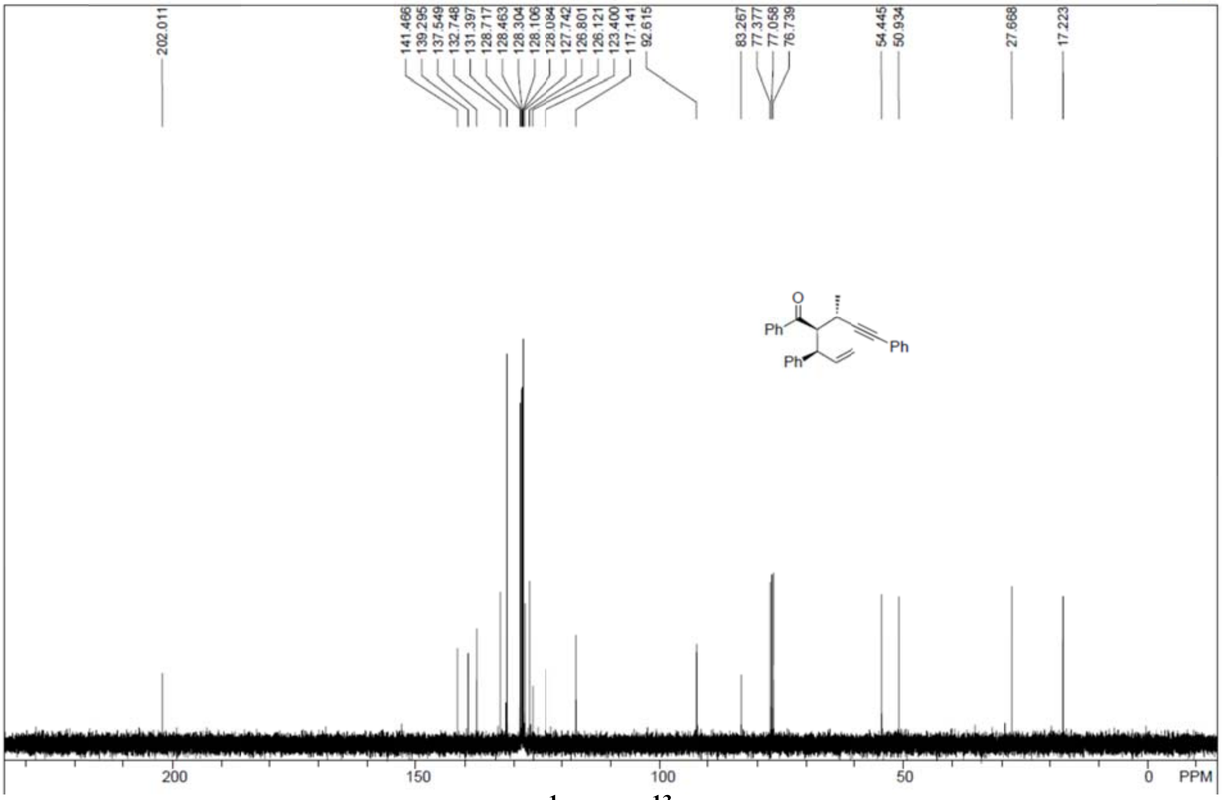

Supplementary Figure 18. ^{1}H and ^{13}C NMR spectrum for 3k.

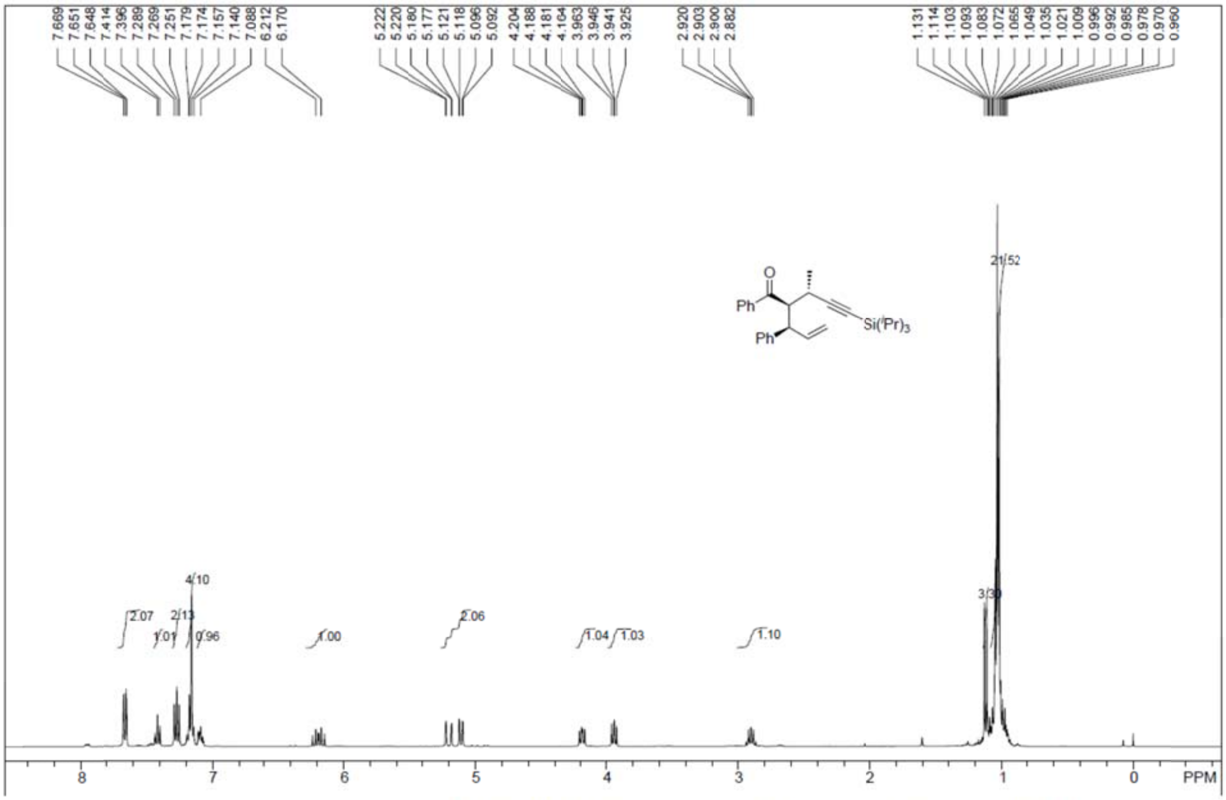

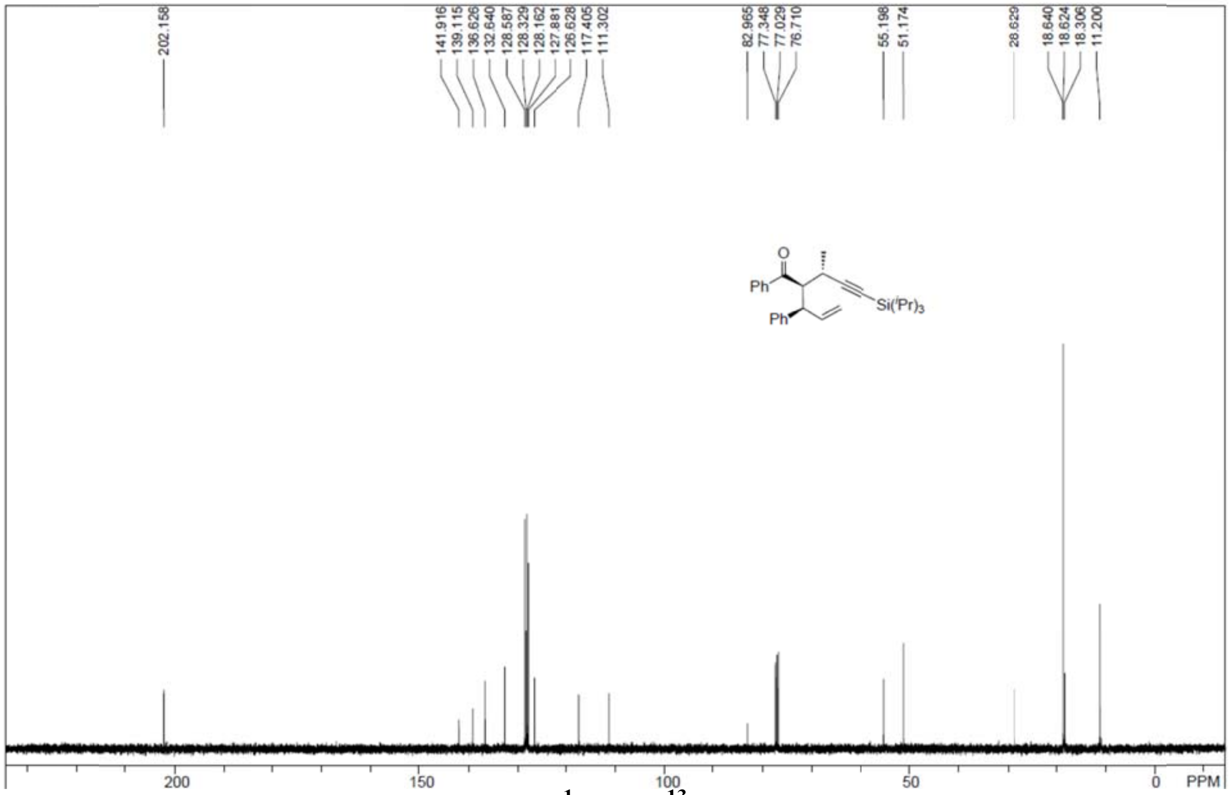

Supplementary Figure 19. ^{1}H and ^{13}C NMR spectrum for 3l.

==

Acq. Operator : [redacted]
Sample Operator : [redacted]
Acq. Instrument : SFC Location : Vial 12
Injection Date : 1/23/2015 9:50:50 AM
Inj Volume : 5.000 µl
Acq. Method : C:\CHEM32\1\METHODS\AGILENT_SFC6.M
Last changed : 1/23/2015 9:27:42 AM by [redacted]
(modified after loading)
Analysis Method : C:\CHEM32\1\METHODS\AGILENT_SFC6.M
Last changed : 1/23/2015 3:36:50 PM by [redacted]
(modified after loading)
Additional Info : Peak(s) manually integrated

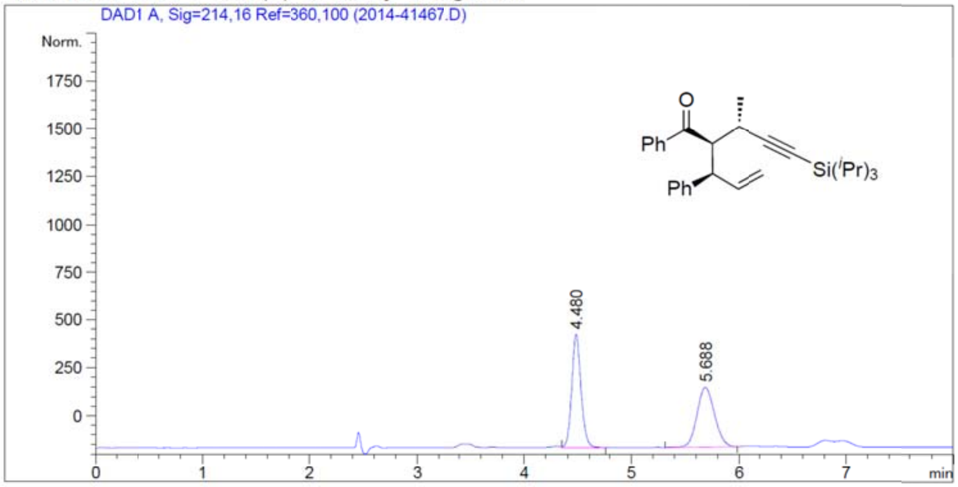

==

Area Percent Report

==

Sorted By : Signal
Multiplier : 1.0000
Dilution : 1.0000
Do not use Multiplier & Dilution Factor with ISTDs

Signal 1: DAD1 A, Sig=214,16 Ref=360,100

Peak #	RetTime [min]	Type	Width [min]	Area [mAU*s]	Height [mAU]	Area %
1	4.480	VB	0.0891	1389.57458	234.58810	49.8166
2	5.688	BV	0.1756	1399.80823	124.09475	50.1834

Totals : 2789.38281 358.68285

==

Supplementary Figure 20. HPLC spectrum for (*rac*)-3l.

```
======================================================================
Acq. Operator   : [redacted]
Sample Operator : [redacted]
Acq. Instrument : SFC                          Location : Vial 17
Injection Date  : 1/23/2015 10:24:55 AM
                                             Inj Volume : 5.000 µl
Acq. Method     : C:\CHEM32\1\METHODS\AGILENT_SFC6.M
Last changed    : 1/23/2015 9:27:42 AM by [redacted]
                  (modified after loading)
Analysis Method : C:\CHEM32\1\METHODS\AGILENT_SFC6.M
Last changed    : 1/23/2015 3:42:54 PM by [redacted]
                  (modified after loading)
Additional Info : Peak(s) manually integrated
```

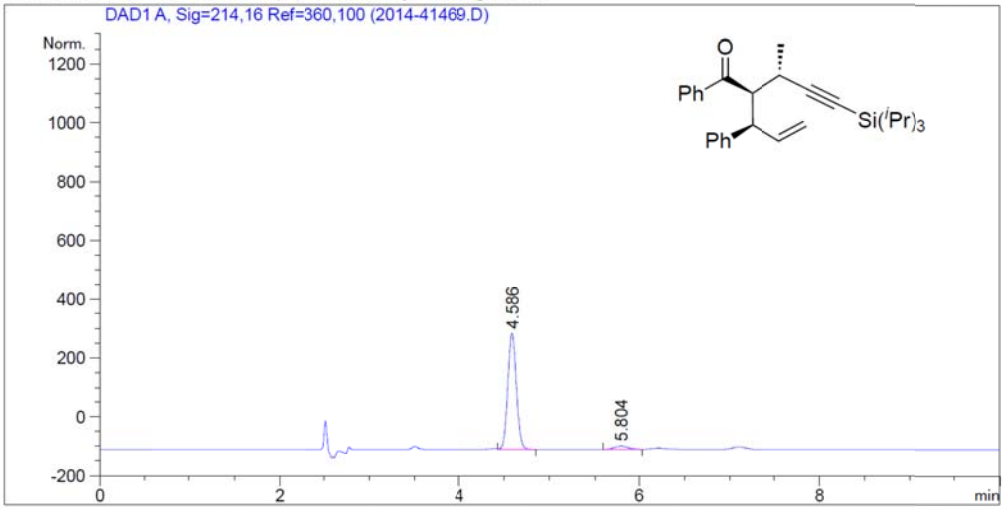


```
======================================================================
                         Area Percent Report
======================================================================

Sorted By             :      Signal
Multiplier            :      1.0000
Dilution              :      1.0000
Do not use Multiplier & Dilution Factor with ISTDs


Signal 1: DAD1 A, Sig=214,16 Ref=360,100

Peak RetTime Type  Width     Area      Height     Area
  #   [min]        [min]   [mAU*s]     [mAU]        %
----|-------|----|-------|----------|----------|--------|
   1   4.586 VB    0.1024 1011.38470  154.49608  95.3492
   2   5.804 BB    0.1614   49.33219    4.48283   4.6508

Totals :                  1060.71689  158.97891
```

Supplementary Figure 21. HPLC spectrum for (*3R,4S,5S*)-3l.

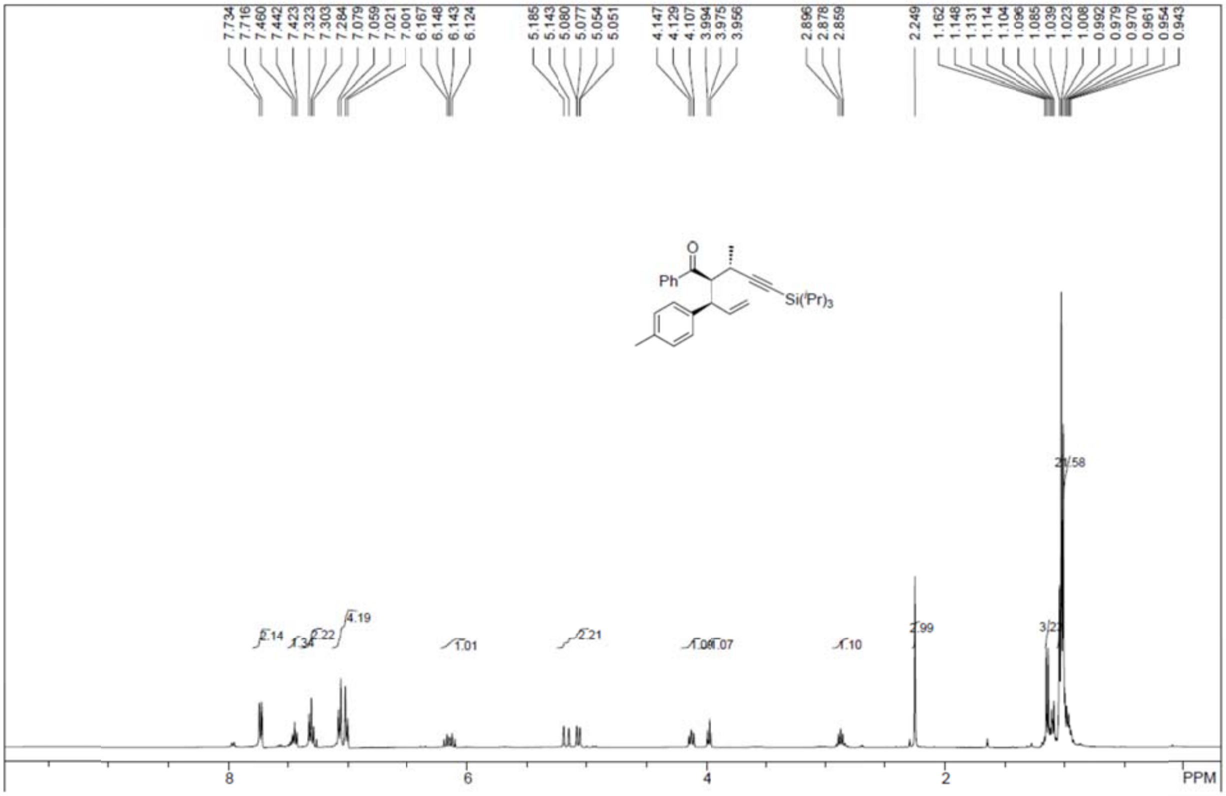

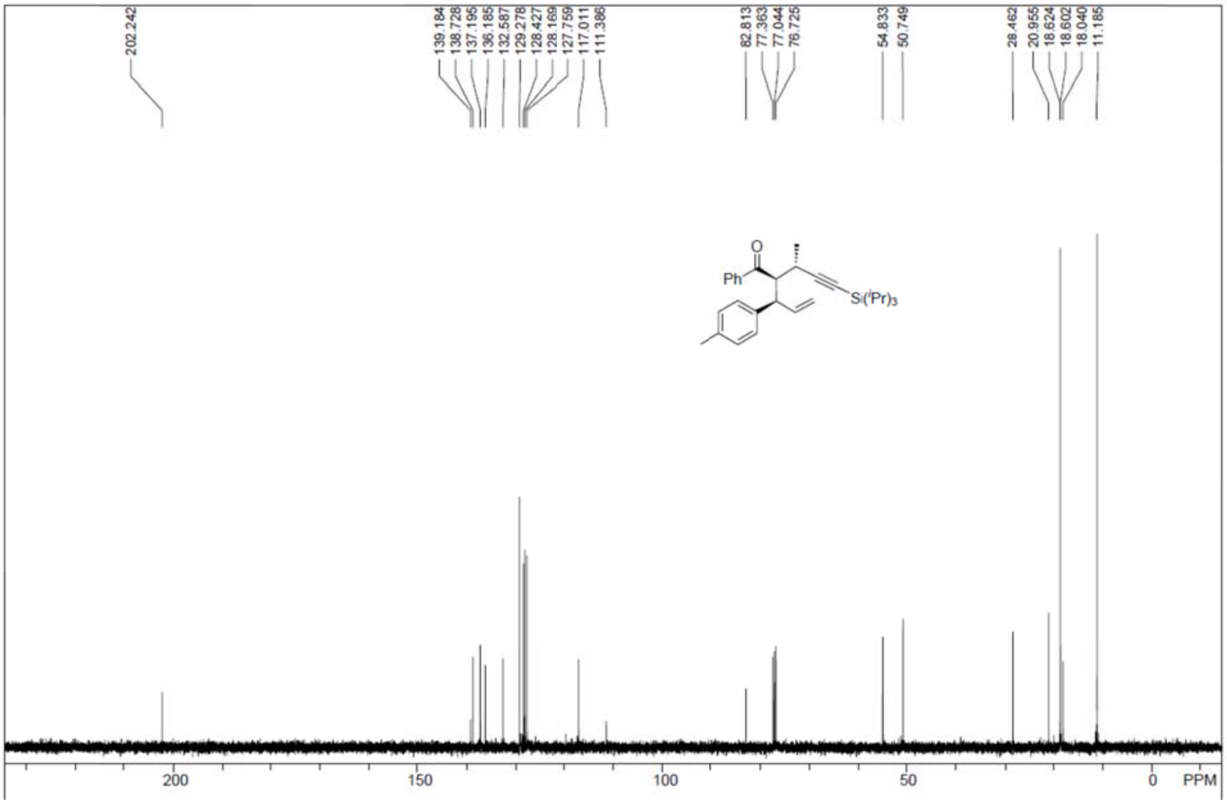

Supplementary Figure 22. ^{1}H and ^{13}C NMR spectrum for 3m.

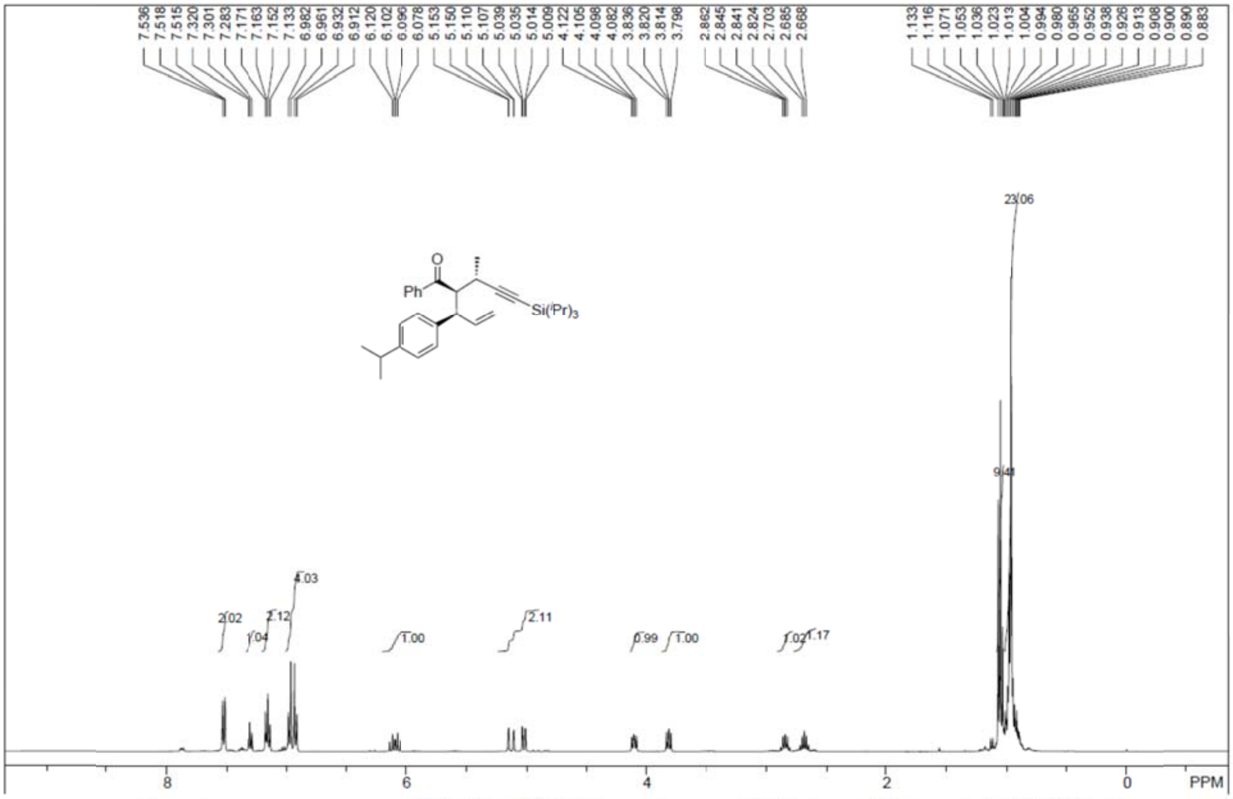

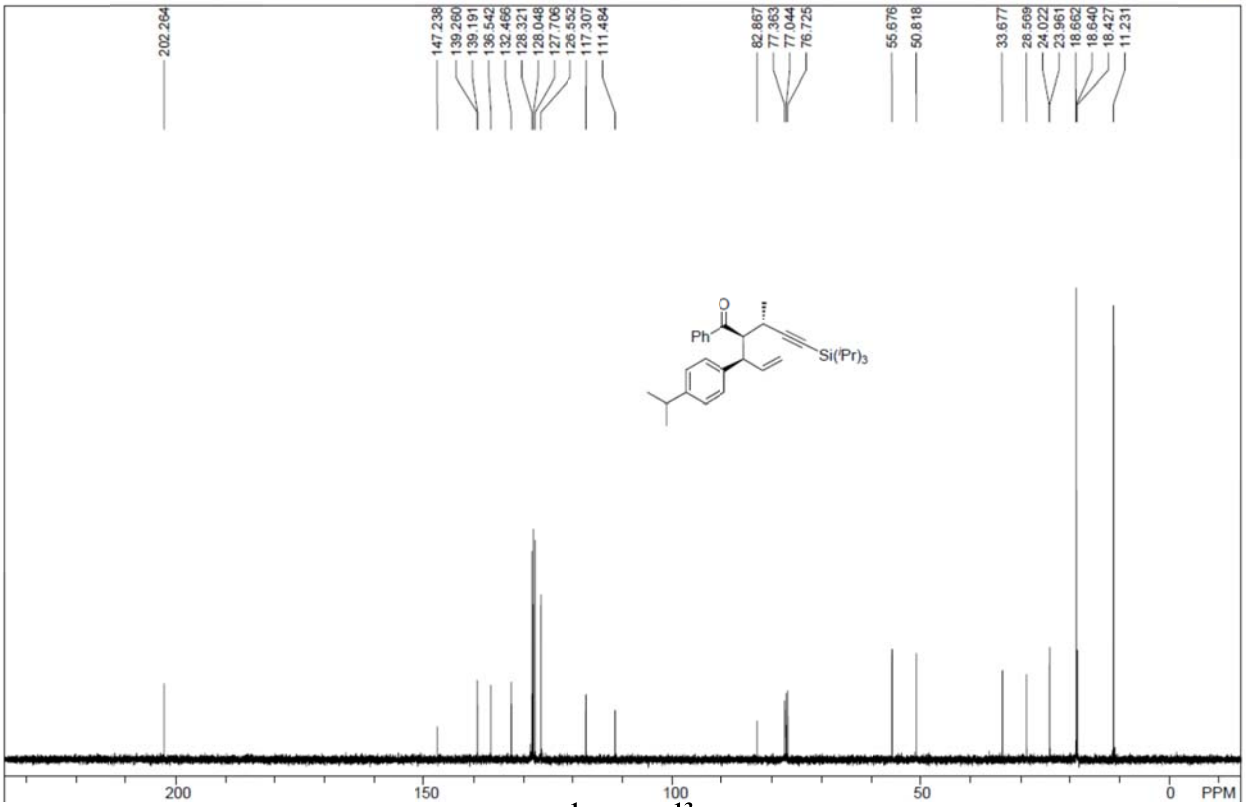

Supplementary Figure 23. ^{1}H and ^{13}C NMR spectrum for 3n.

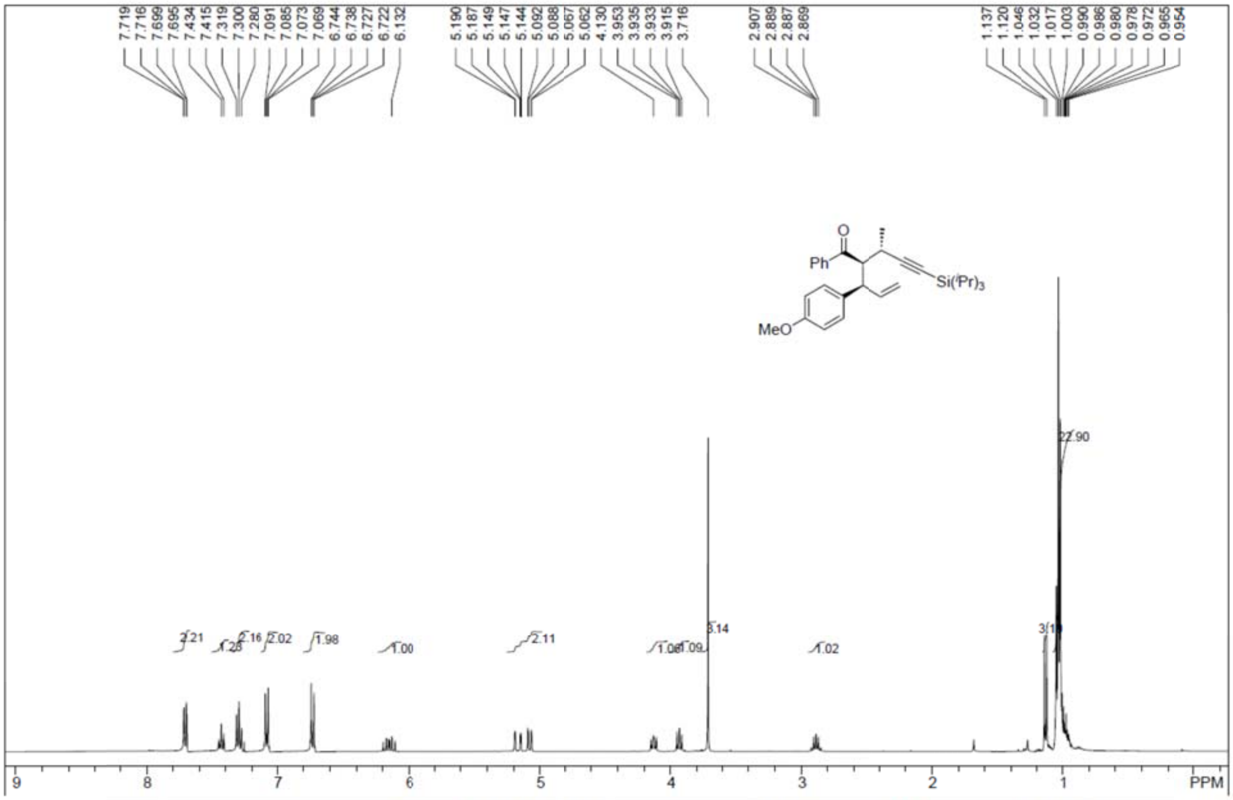

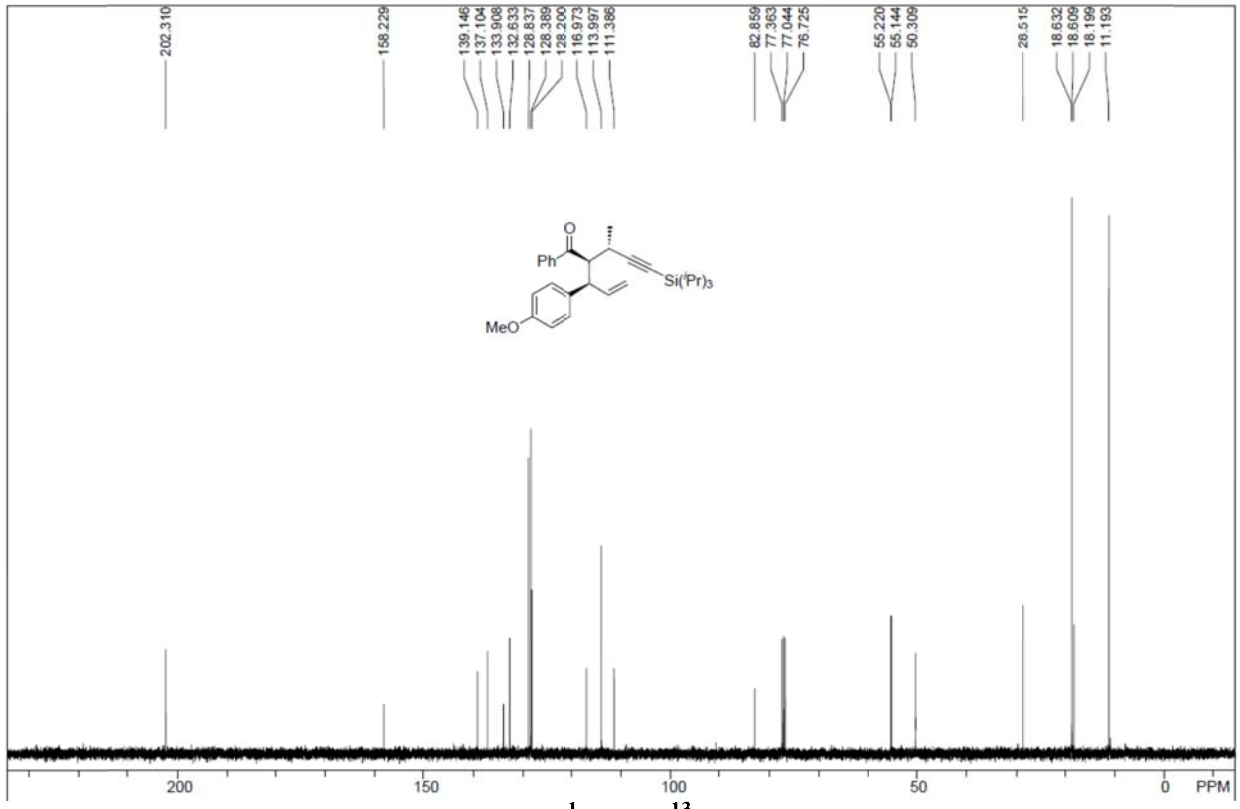

Supplementary Figure 24. ^{1}H and ^{13}C NMR spectrum for 3o.

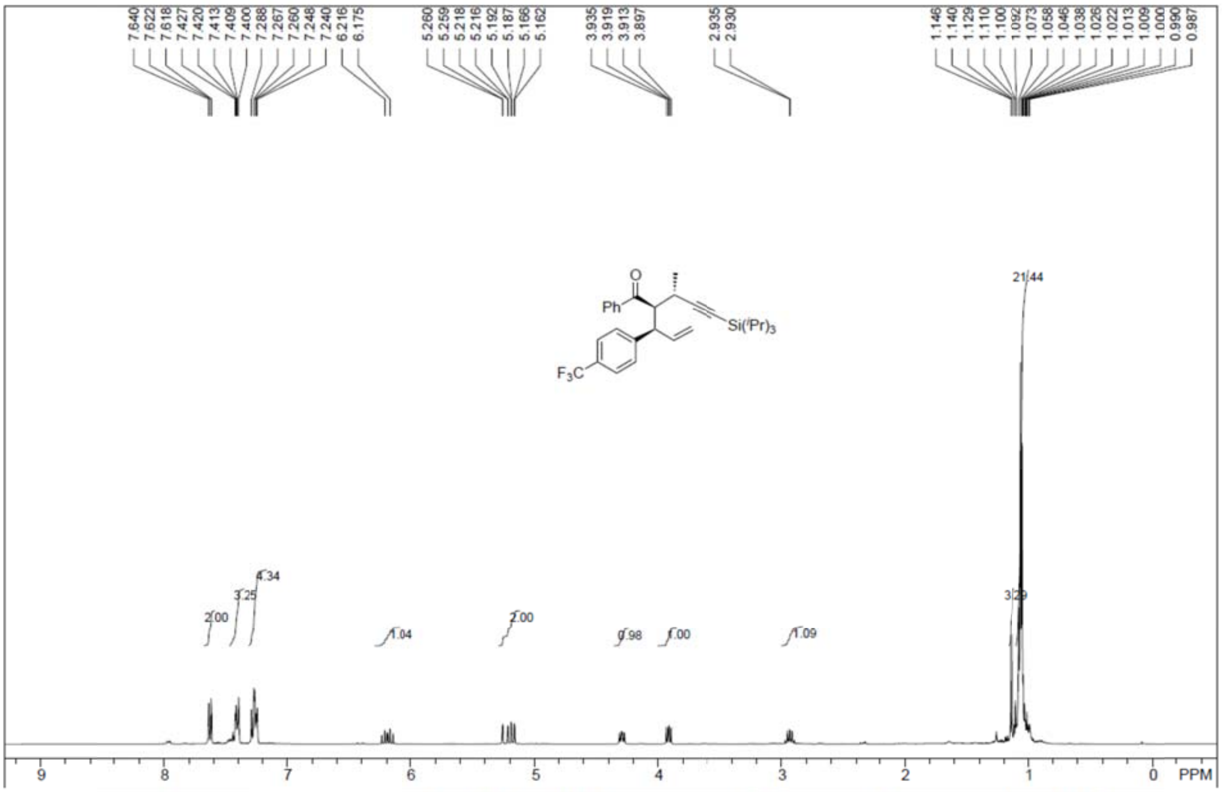

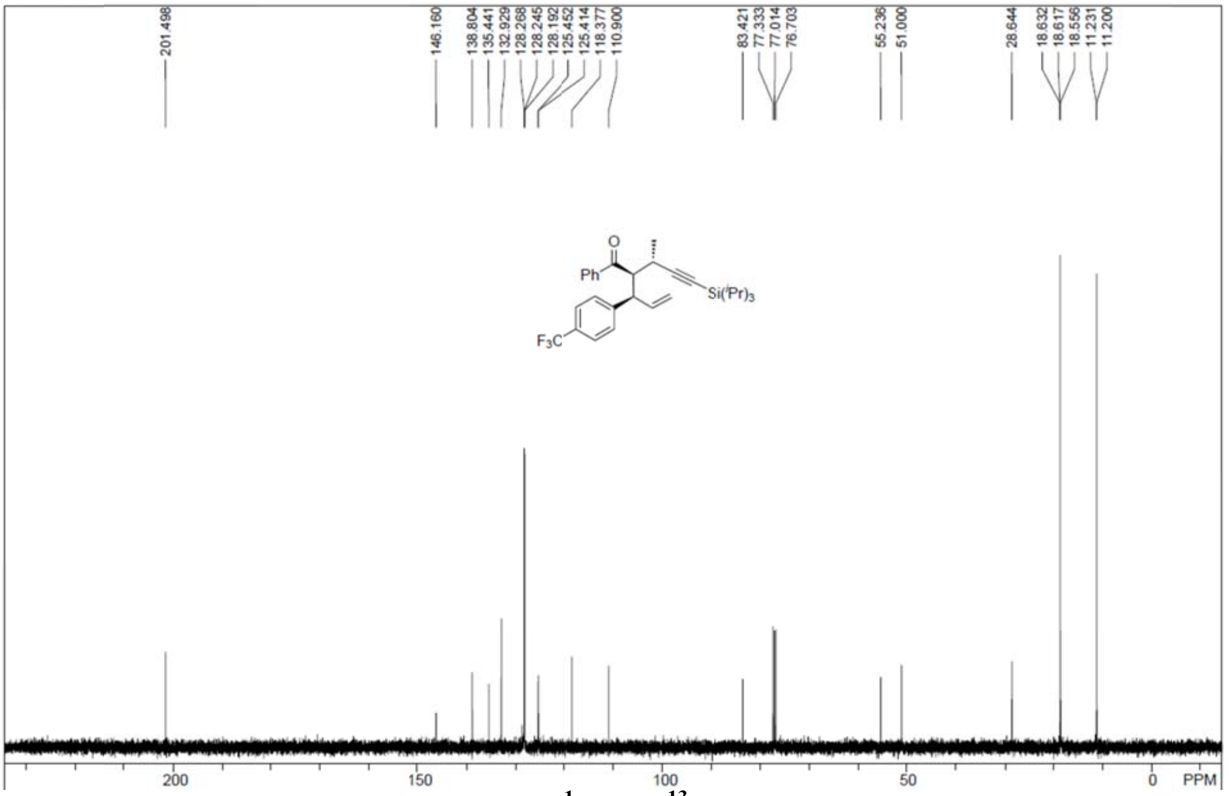

Supplementary Figure 25. ^{1}H and ^{13}C NMR spectrum for 3p.

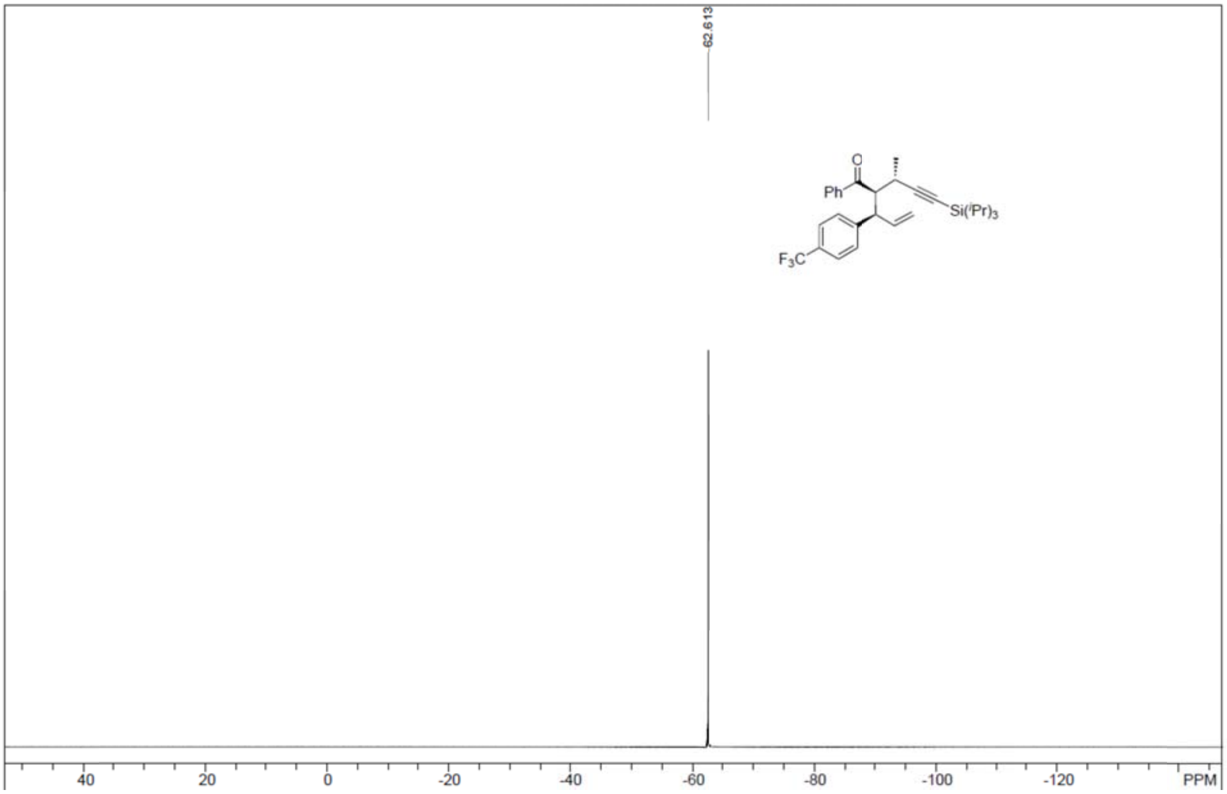

Supplementary Figure 26. ^{19}F NMR spectrum for 3p.

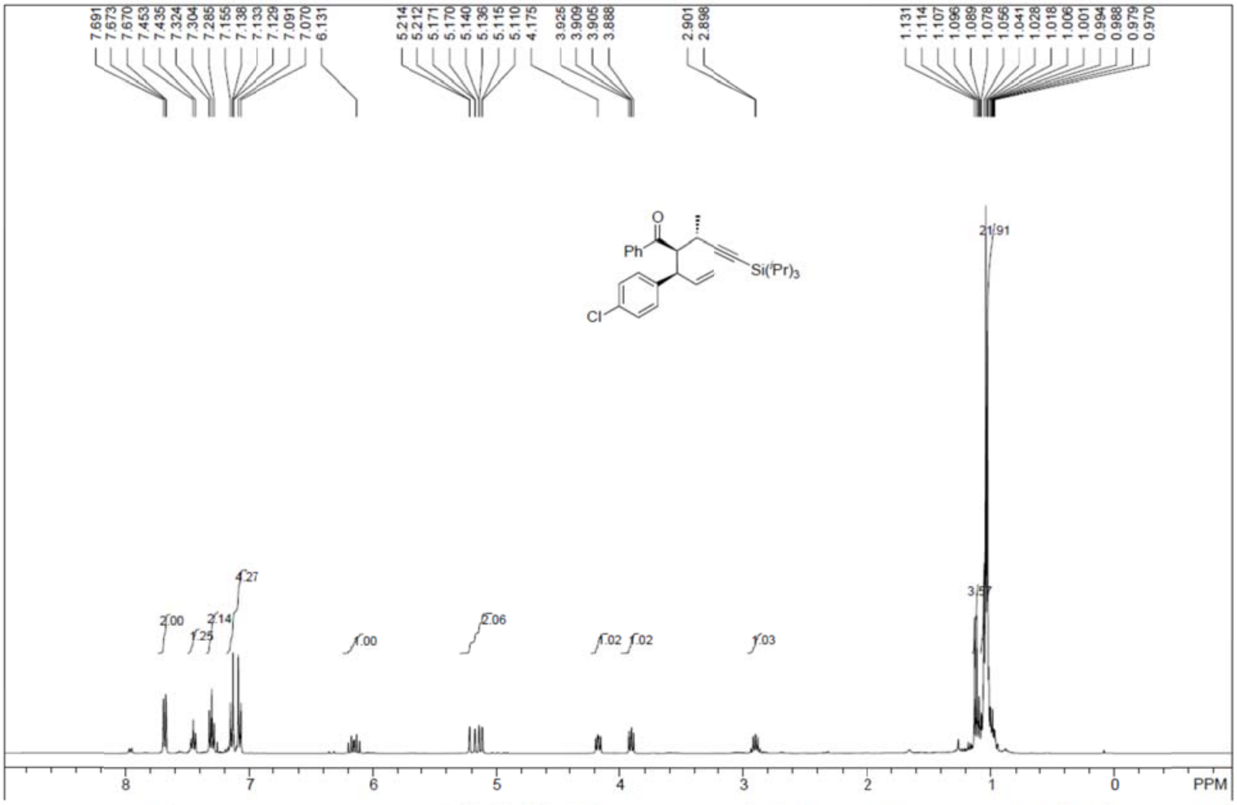

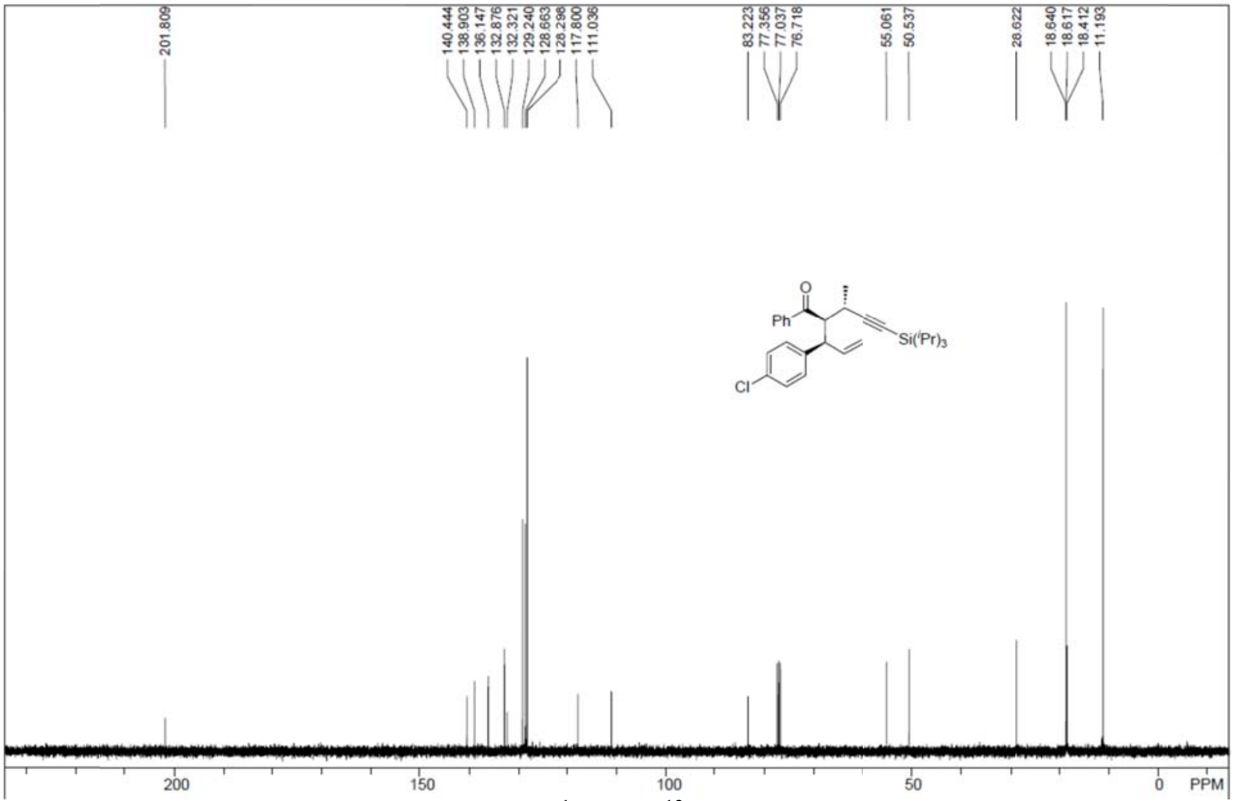

Supplementary Figure 27. ^{1}H and ^{13}C NMR spectrum for 3q.

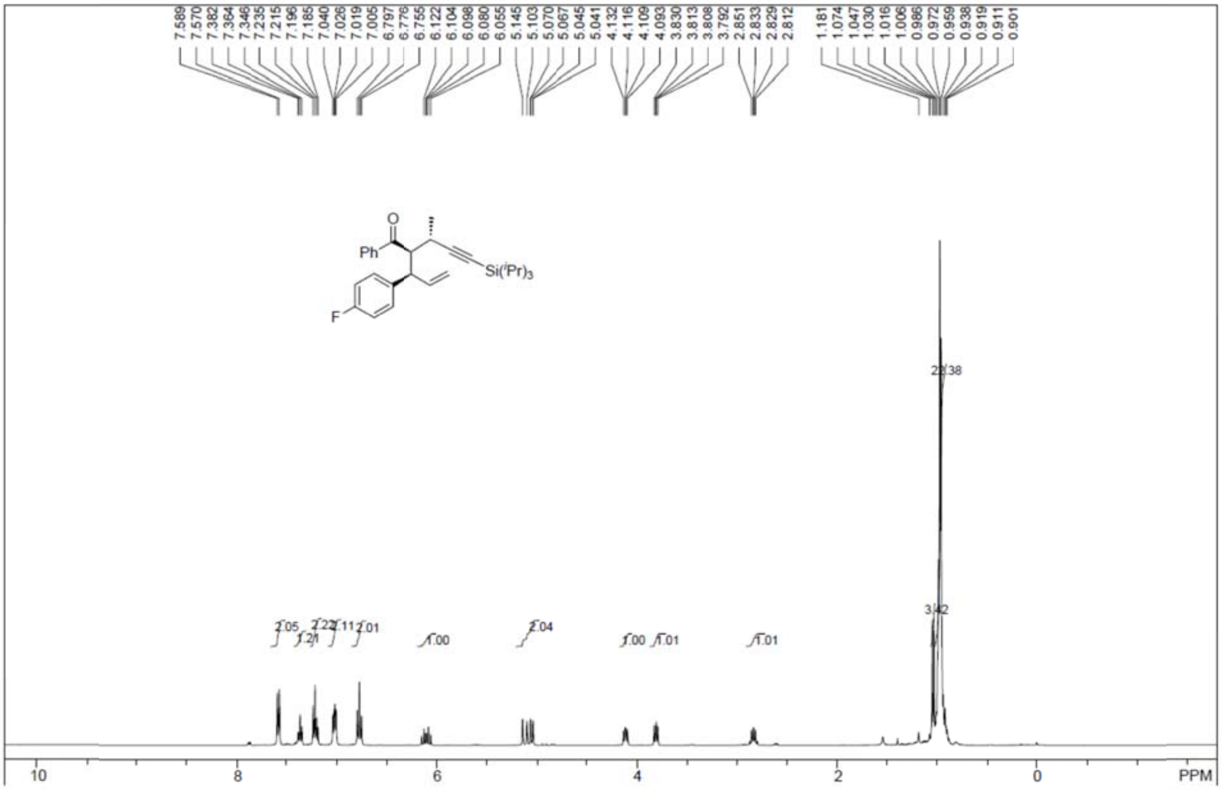

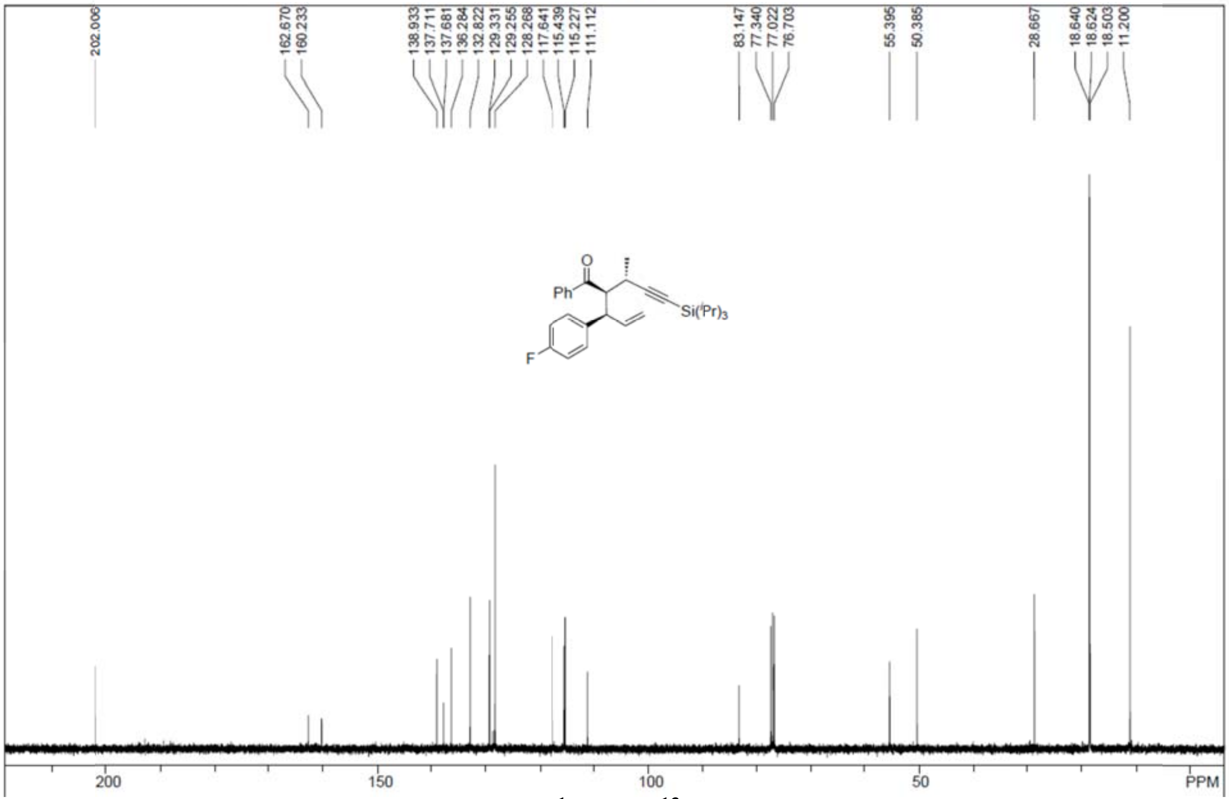

Supplementary Figure 28. ^{1}H and ^{13}C NMR spectrum for 3r.

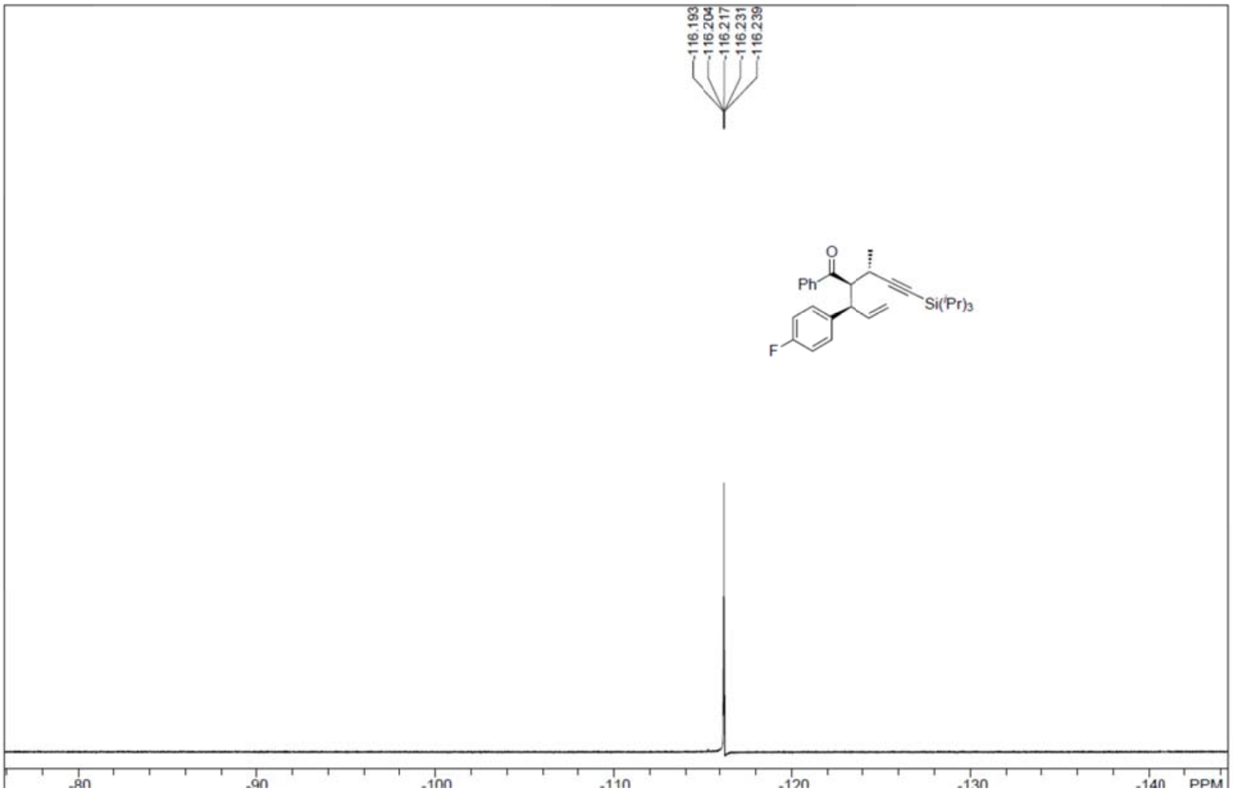

Supplementary Figure 29. ^{19}F spectrum for 3r.

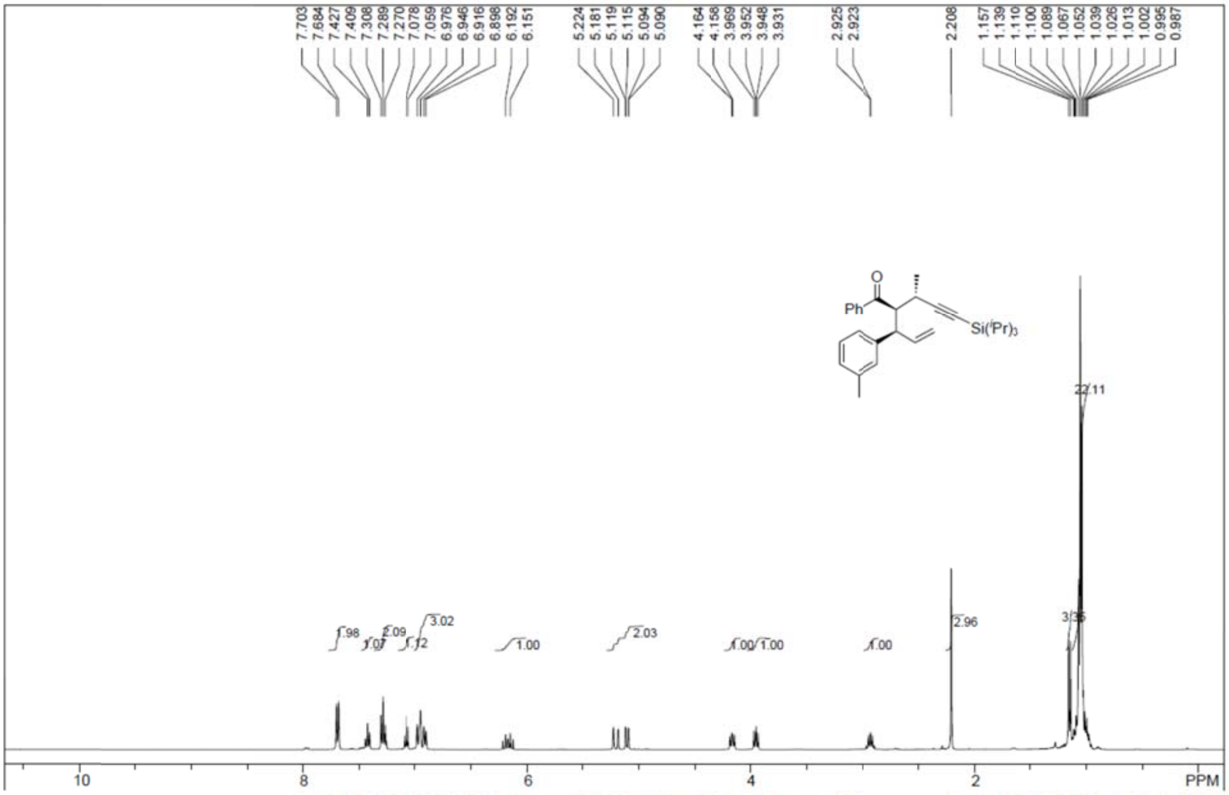

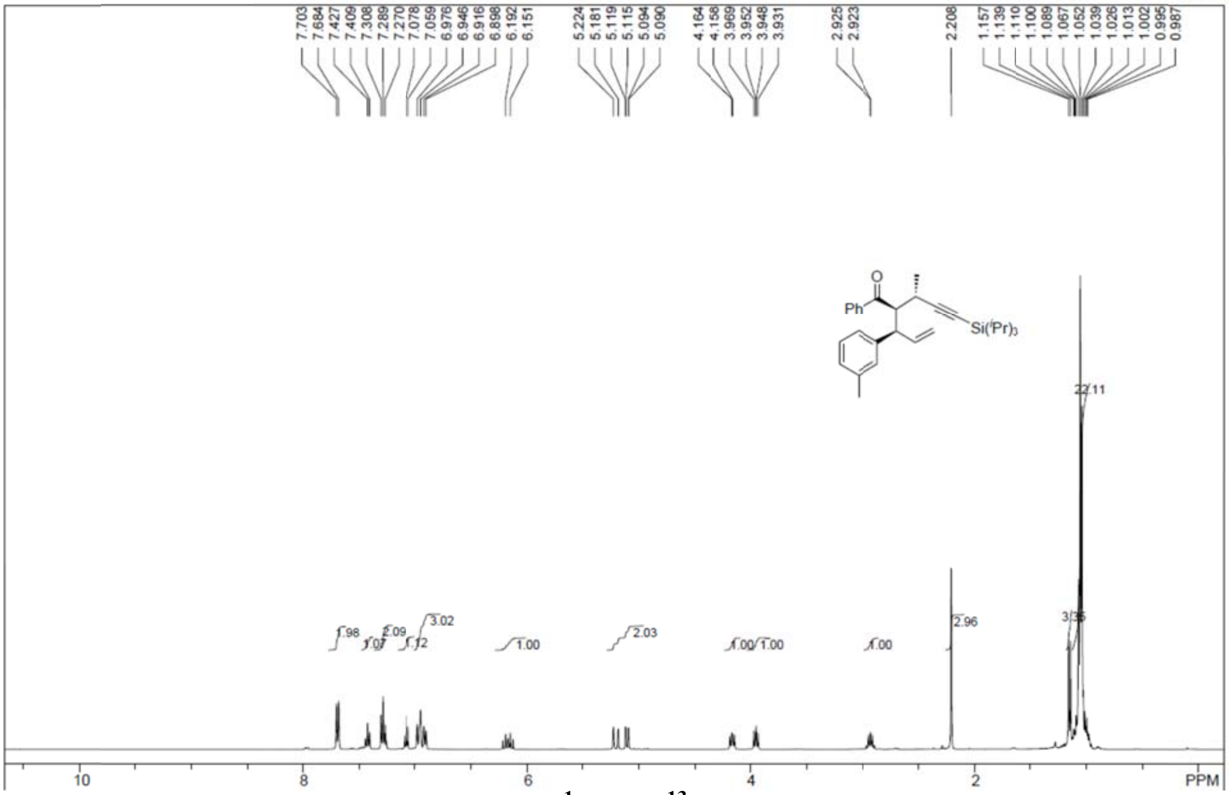

Supplementary Figure 30. ^{1}H and ^{13}C NMR spectrum for 3s.

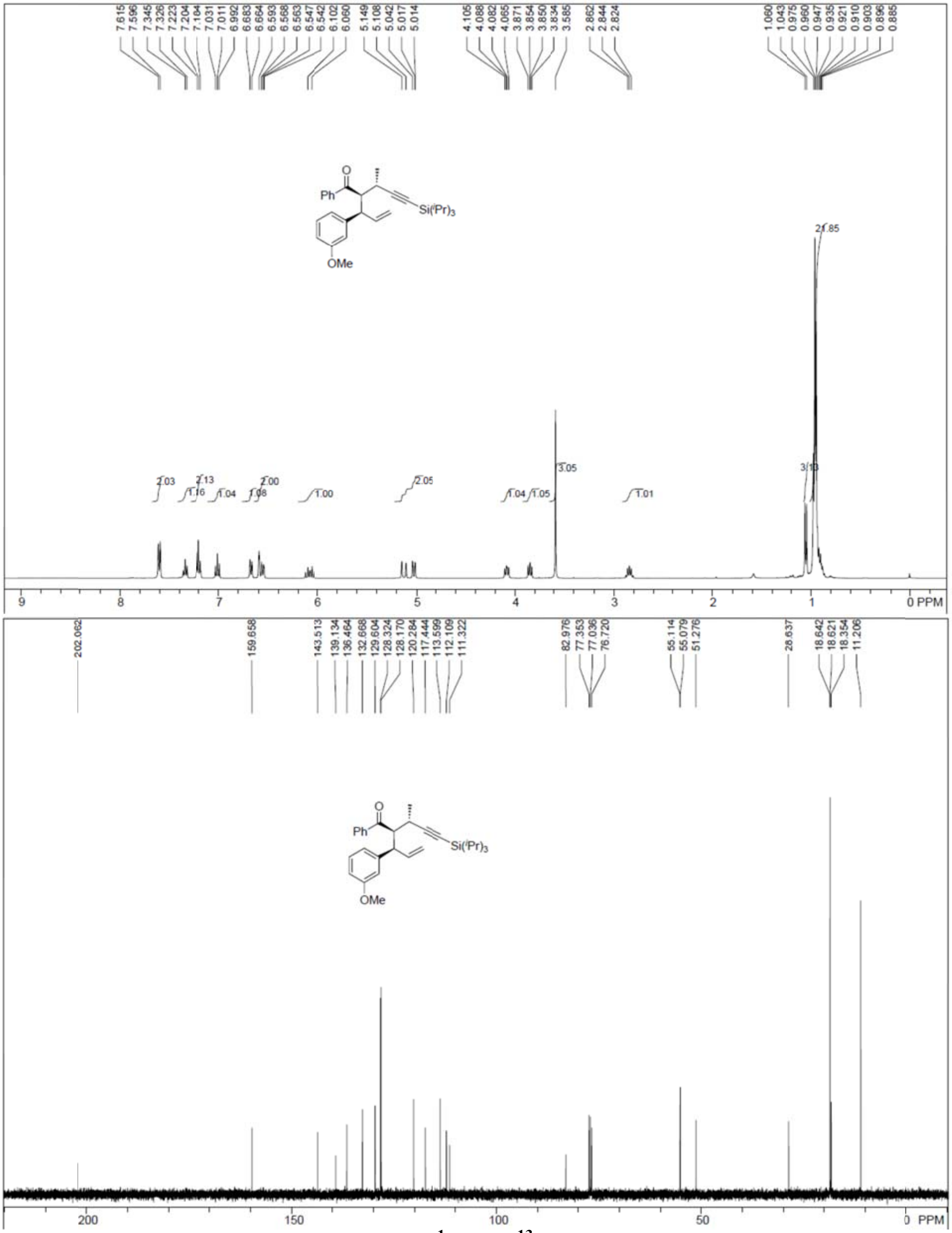

Supplementary Figure 31. ^{1}H and ^{13}C NMR spectrum for 3t.

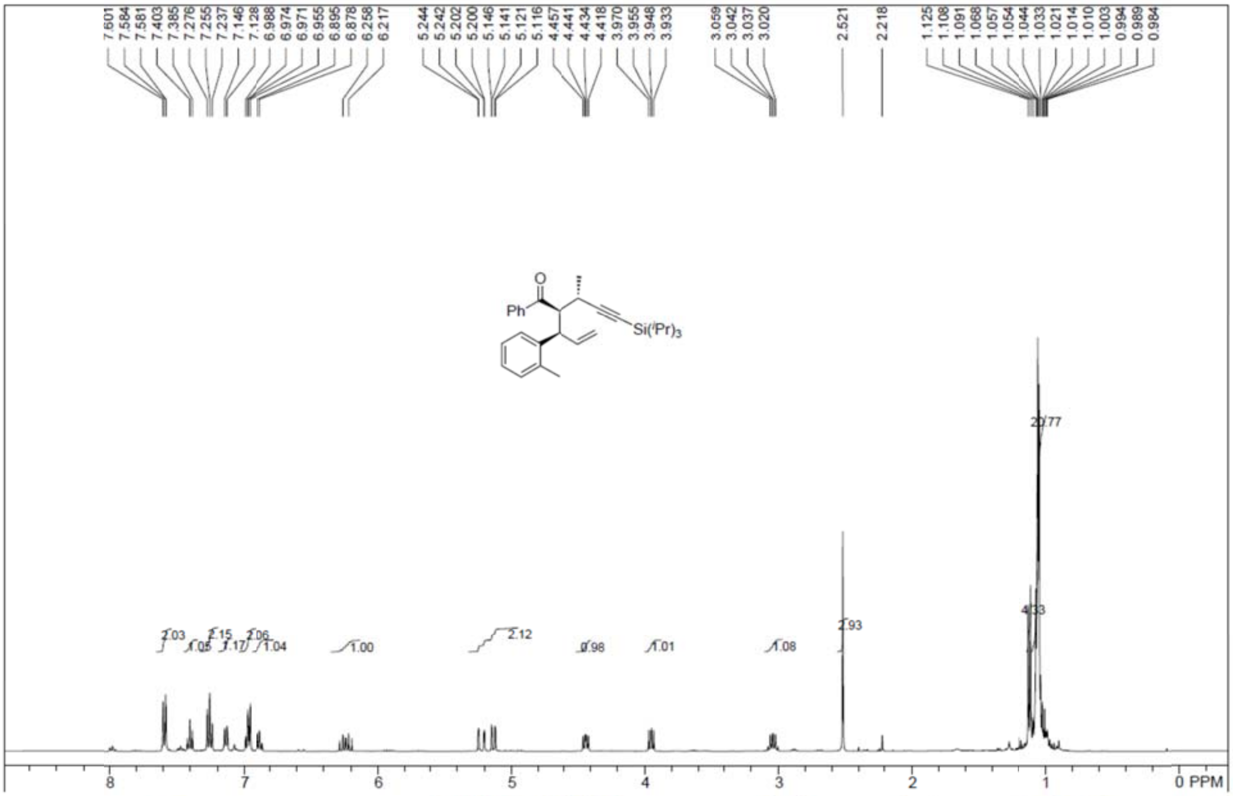

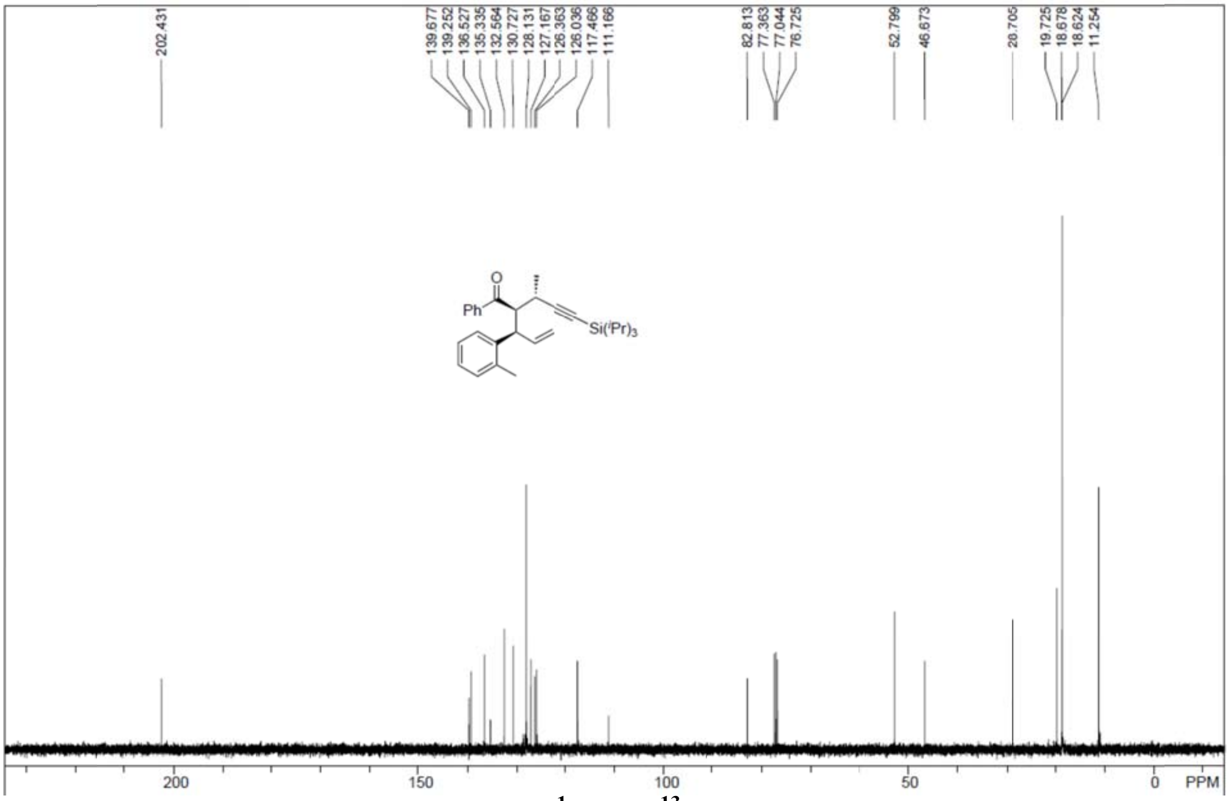

Supplementary Figure 32. ^{1}H and ^{13}C NMR spectrum for 3u.

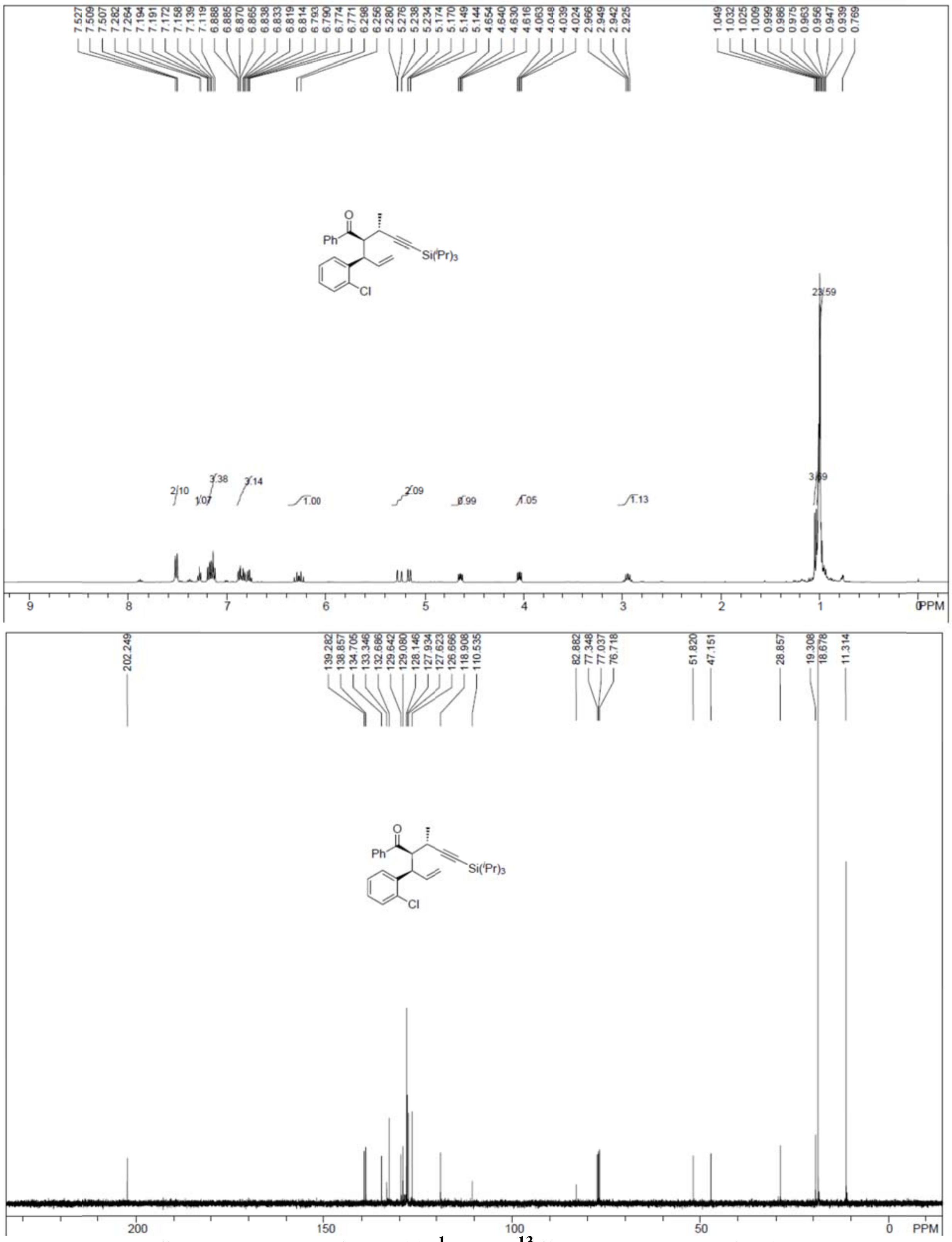

Supplementary Figure 33. ^{1}H and ^{13}C NMR spectrum for 3v.

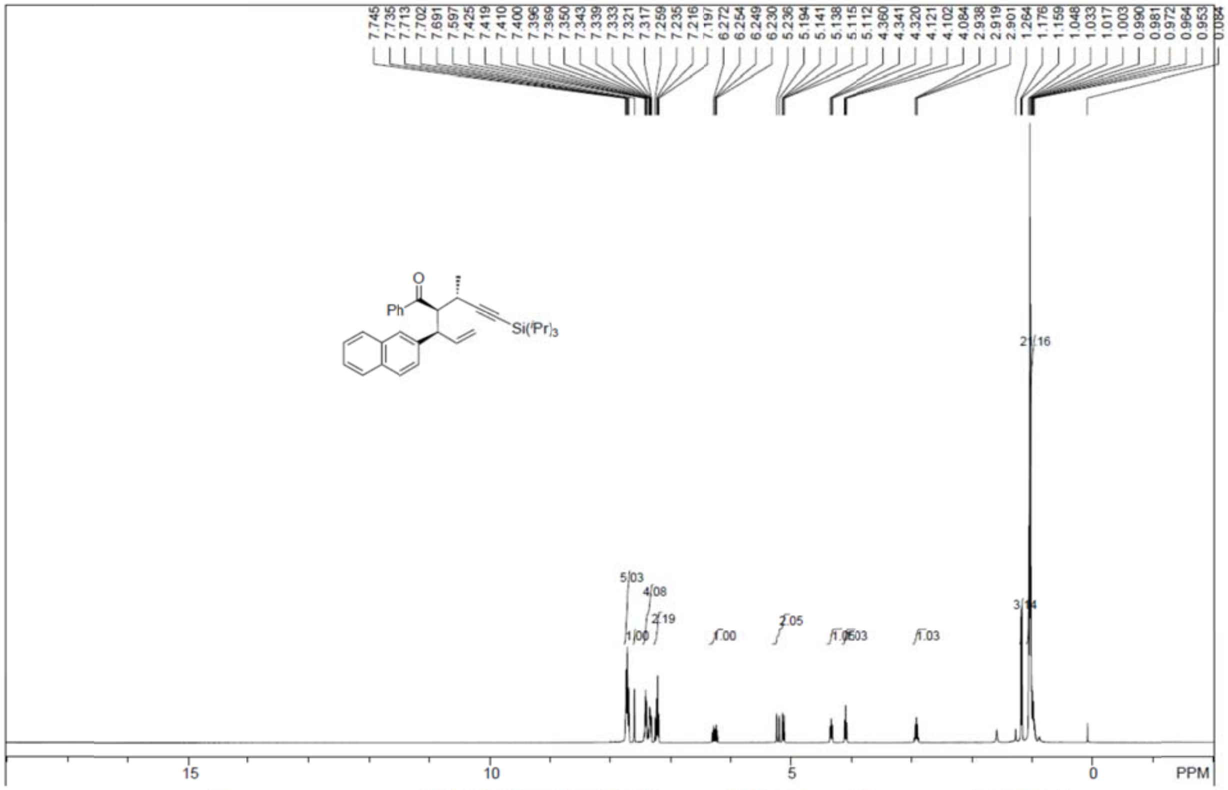

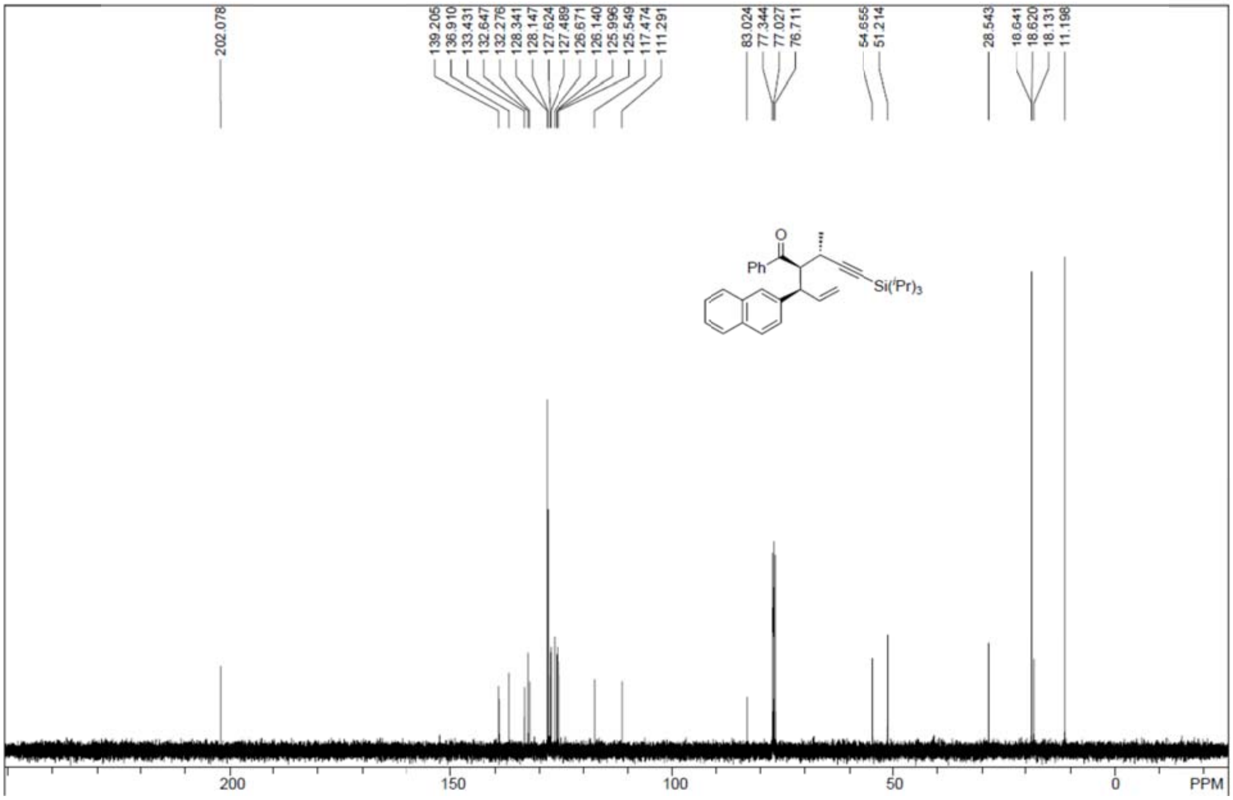

Supplementary Figure 34. ^{1}H and ^{13}C NMR spectrum for 3w.

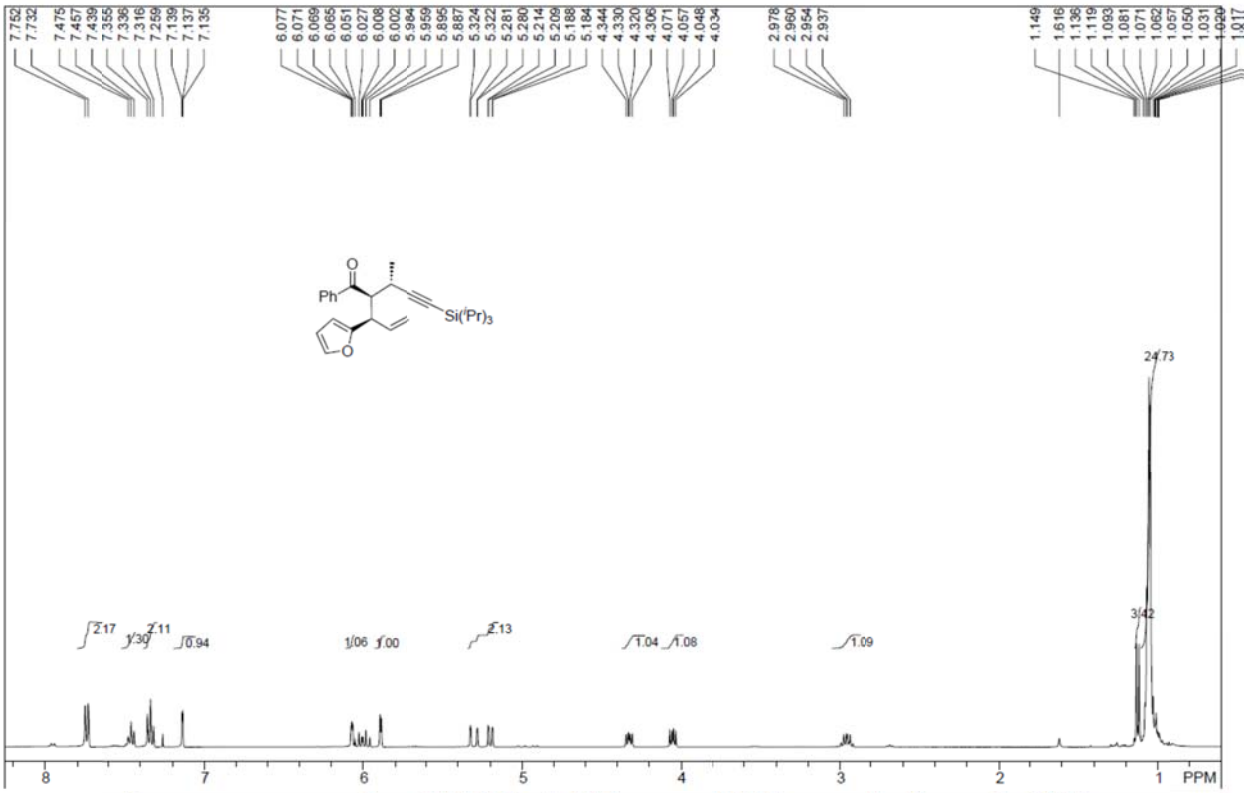

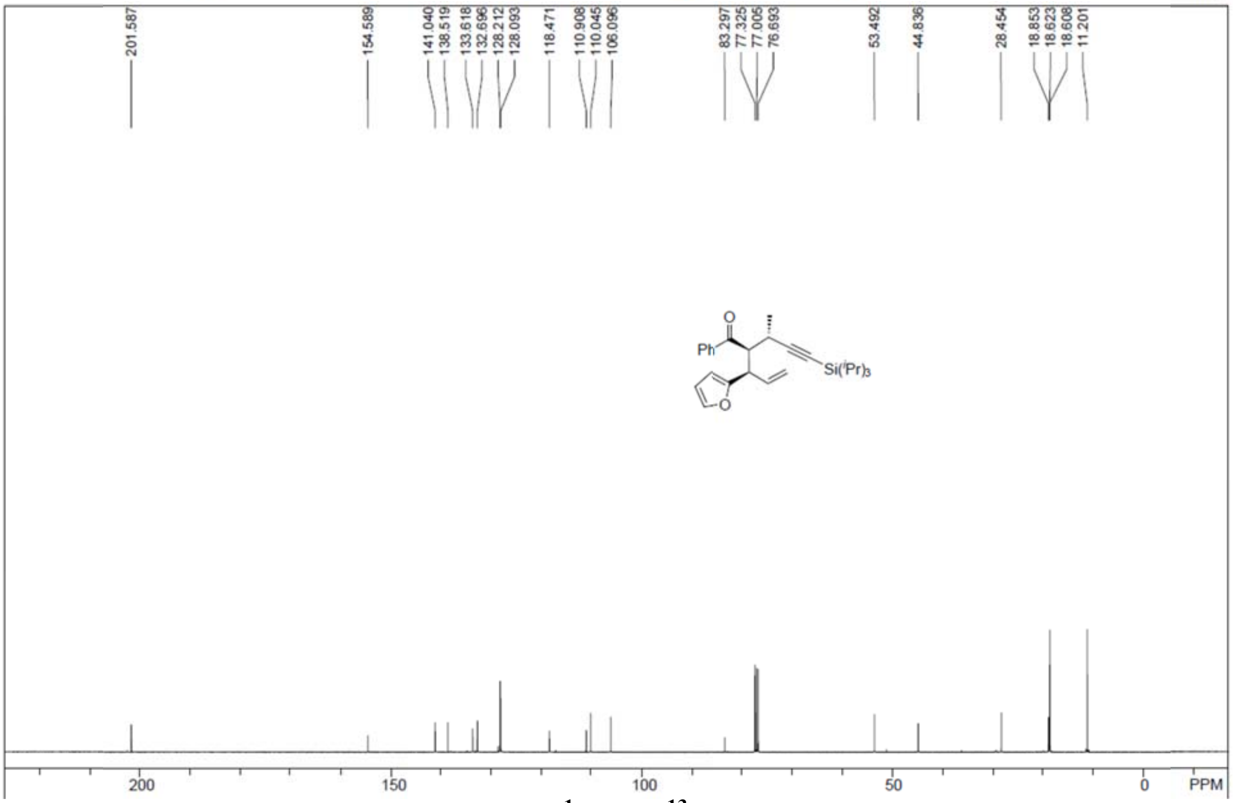

Supplementary Figure 35. ^{1}H and ^{13}C NMR spectrum for 3x.

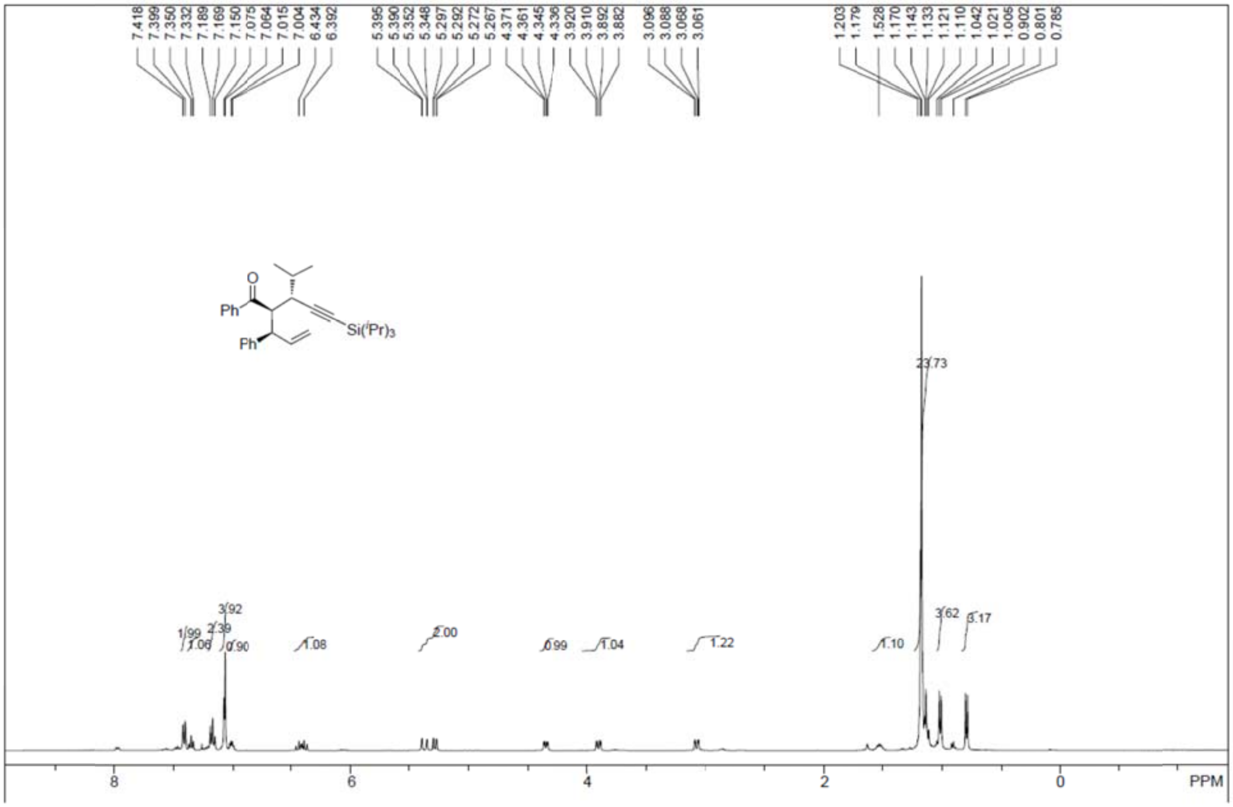

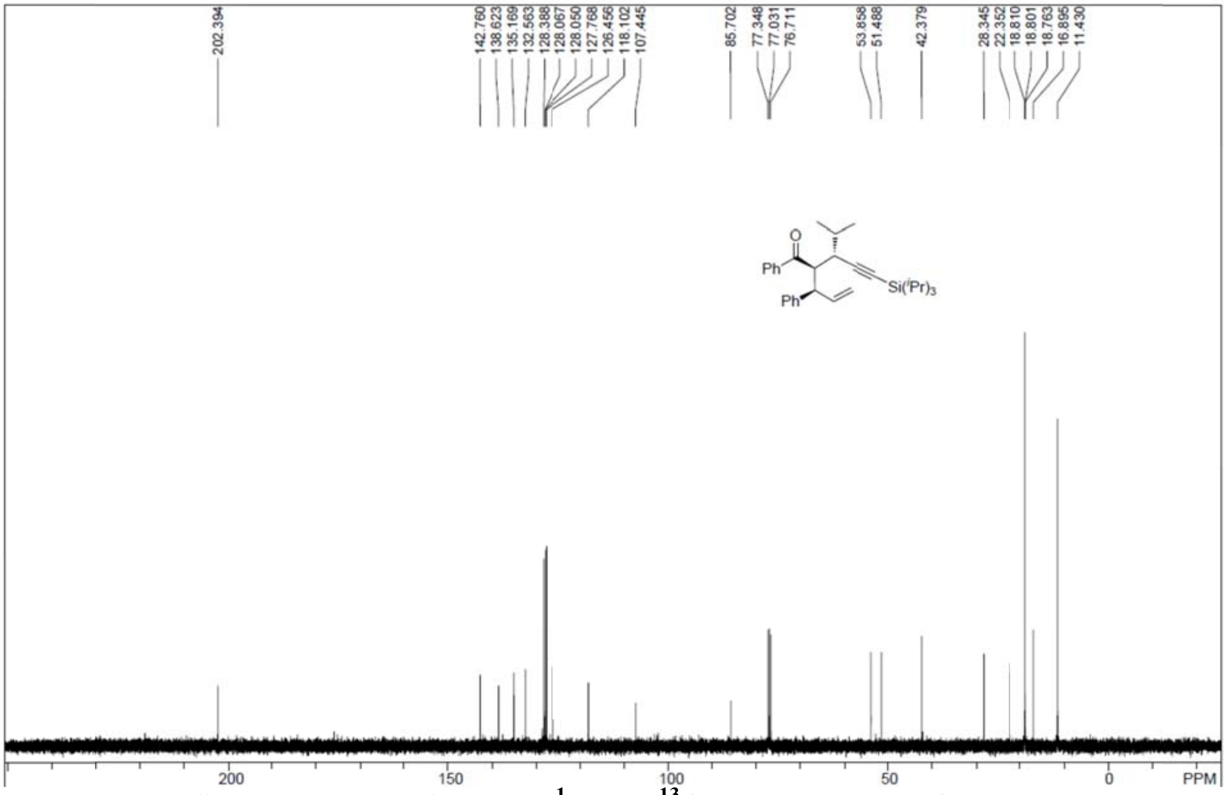

Supplementary Figure 36. ^{1}H and ^{13}C NMR spectrum for 3y.

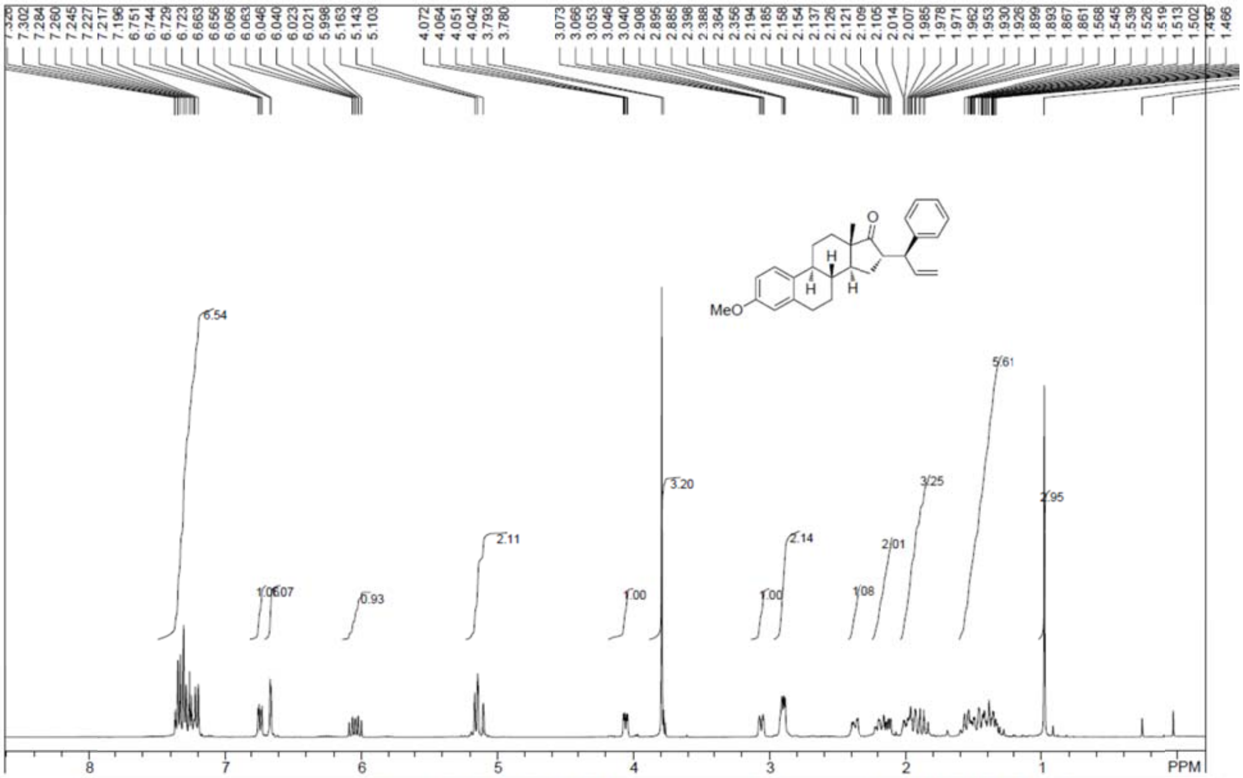

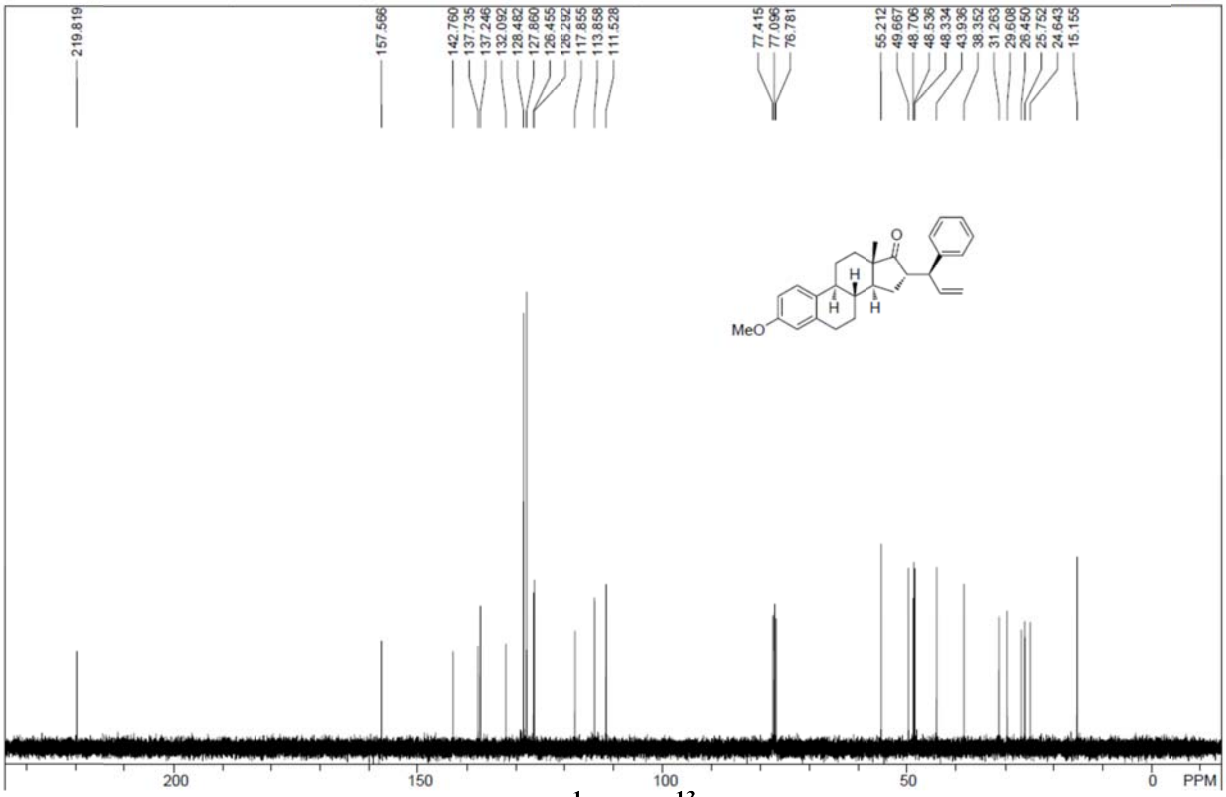

Supplementary Figure 37. ^{1}H and ^{13}C NMR spectrum for 7.

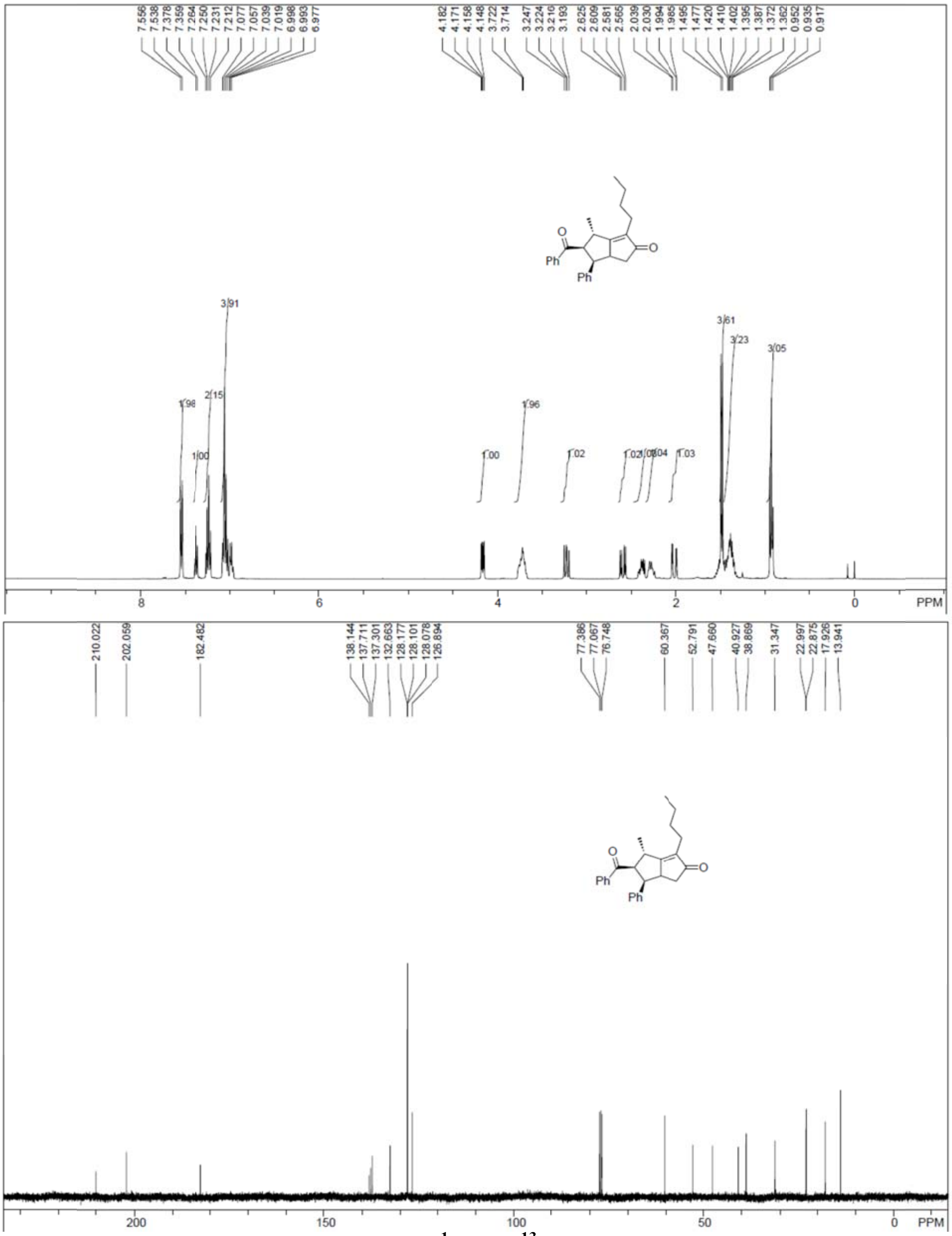

Supplementary Figure 38. ^{1}H and ^{13}C NMR spectrum for 8.

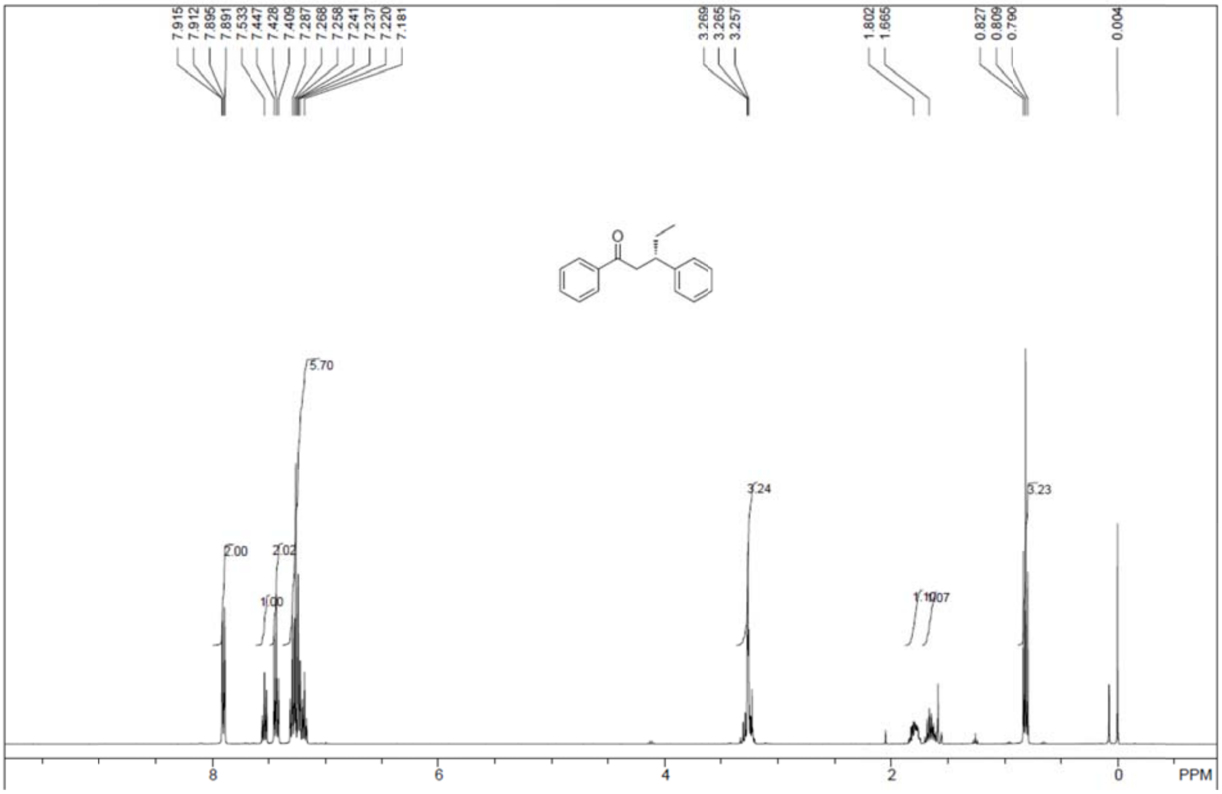

Supplementary Figure 39. ^{1}H NMR spectrum for (*S*)-1j.

==== Shimadzu LCsolution Analysis Report ====

C:\LabSolutions\Data\Project1\Project1\BDC\26\bdc-24-25--rac.lcd

Acquired by : Admin
Sample Name : bdc-24-25--rac
method :AD-H, 95/5,1.0, 214
Injection Volume : 1 uL
Data File Name : bdc-24-25--rac.lcd
Method File Name : 123.lcm
Report File Name : Default.lcr
Data Acquired : 2015-4-23 11:57:20
Data Processed : 2015-4-23 12:05:48

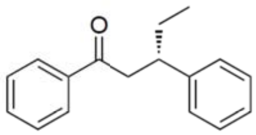

<Chromatogram>

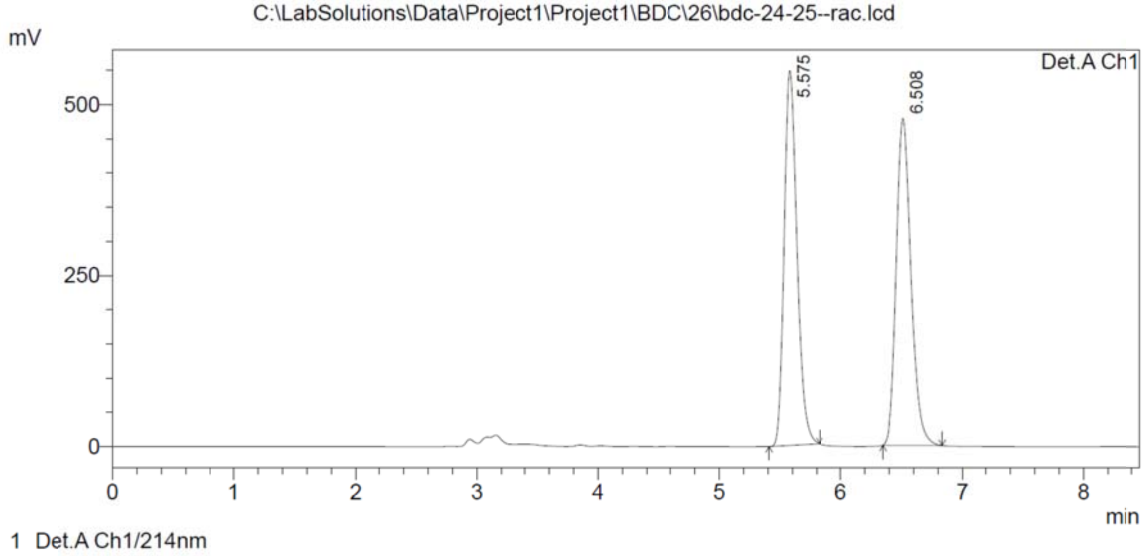

PeakTable

Detector A Ch1 214nm

Peak#	Ret. Time	Area	Height	Area %
1	5.575	4009603	548039	49.897
2	6.508	4026098	478011	50.103
Total		8035702	1026050	100.000

Supplementary Figure 40. HPLC spectrum for *rac*-1j.

==== Shimadzu LCsolution Analysis Report ====

C:\LabSolutions\Data\Project1\Project1\BDC\26\bdc-24-25--.lcd

Acquired by	: Admin
Sample Name	: bdc-24-25--
method	:AD-H, 95/5,1.0, 214
Injection Volume	: 1 uL
Data File Name	: bdc-24-25--.lcd
Method File Name	: 123.lcm
Report File Name	: Default.lcr
Data Acquired	: 2015-4-23 11:46:59
Data Processed	: 2015-4-23 11:56:15

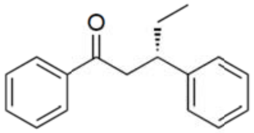

<Chromatogram>

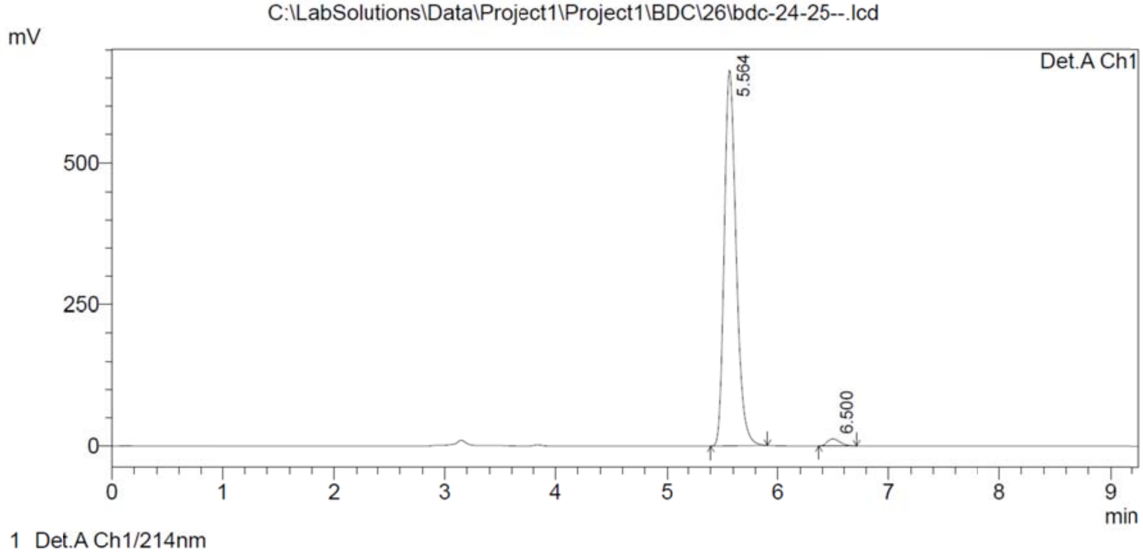

PeakTable

Detector A Ch1 214nm

Peak#	Ret. Time	Area	Height	Area %
1	5.564	4910943	663860	97.975
2	6.500	101480	12610	2.025
Total		5012423	676470	100.000

Supplementary Figure 41. HPLC spectrum for *(S)*-1j.

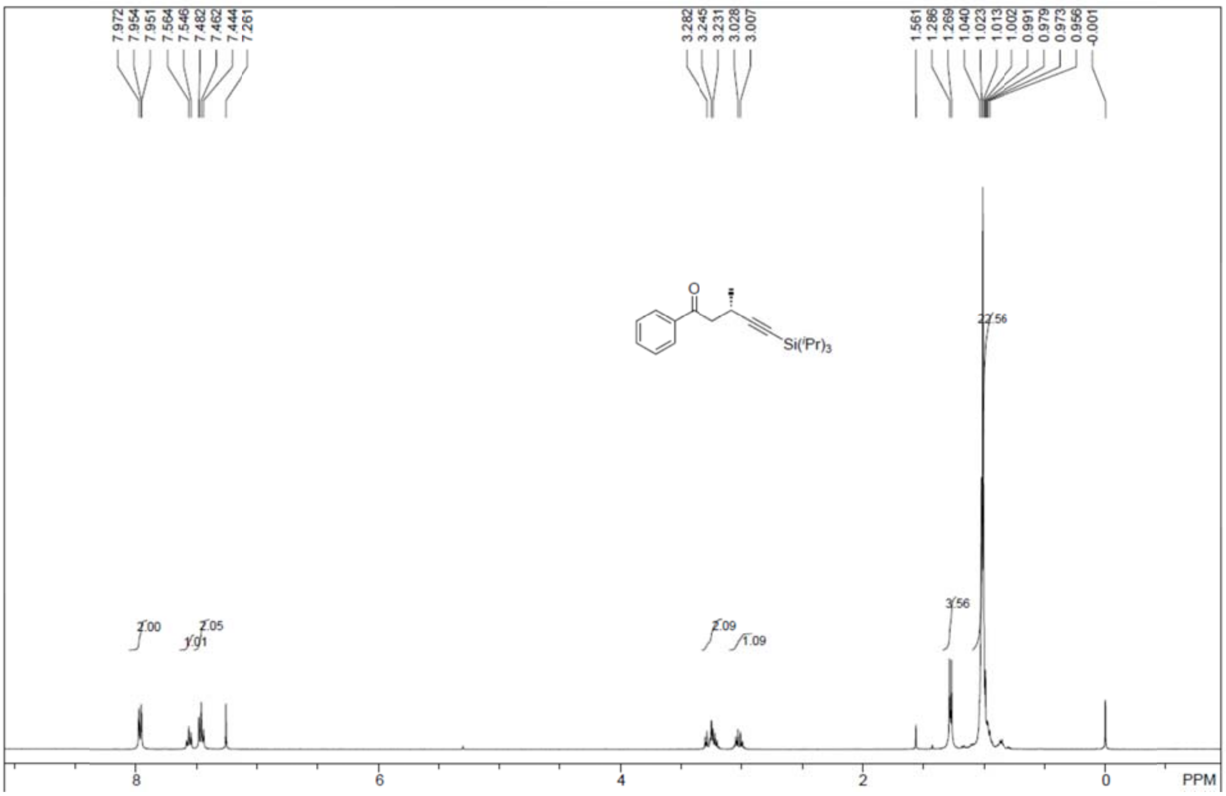

Supplementary Figure 42. ^{1}H NMR spectrum for (*S*)-1l.

==
Acq. Operator : [redacted]
Sample Operator : [redacted]
Acq. Instrument : SFC Location : Vial 74
Injection Date : 4/24/2015 3:58:09 PM
Inj Volume : 5.000 µl
Acq. Method : C:\CHEM32\1\METHODS\AGILENT_SFC6.M
Last changed : 4/24/2015 3:10:21 PM by [redacted]
(modified after loading)
Analysis Method : C:\CHEM32\1\METHODS\AGILENT_SFC6.M
Last changed : 4/27/2015 9:57:16 AM by [redacted]
(modified after loading)
Additional Info : Peak(s) manually integrated

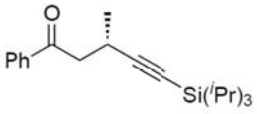

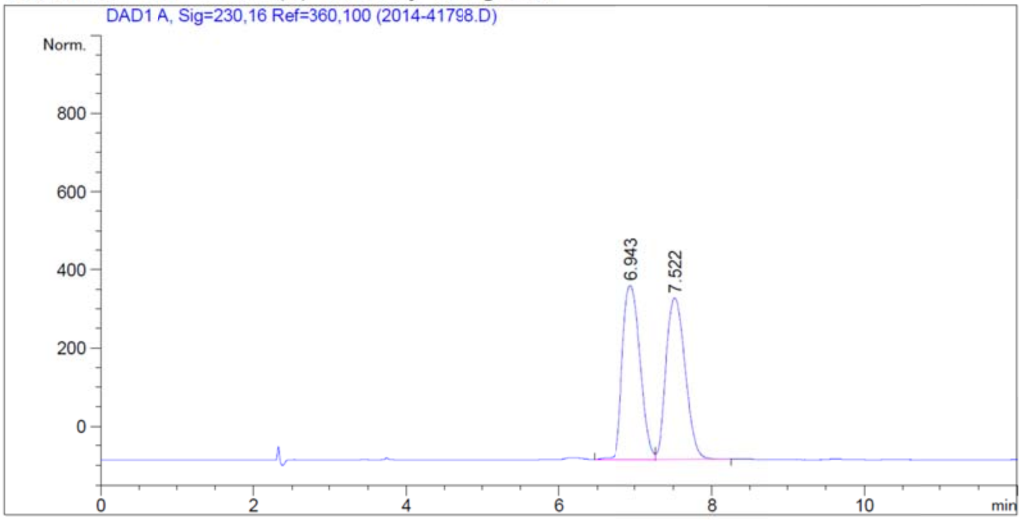

==
Area Percent Report
==

Sorted By : Signal
Multiplier : 1.0000
Dilution : 1.0000
Do not use Multiplier & Dilution Factor with ISTDs

Signal 1: DAD1 A, Sig=230,16 Ref=360,100

Peak #	RetTime [min]	Type	Width [min]	Area [mAU*s]	Height [mAU]	Area %
1	6.943	BV	0.2601	4276.85596	266.66382	49.9585
2	7.522	VB	0.2809	4283.96484	247.54297	50.0415
Totals :				8560.82080	514.20679	

Supplementary Figure 43. HPLC spectrum for *rac*-1l.

```
=====================================================================
Acq. Operator   : [redacted]
Sample Operator : [redacted]
Acq. Instrument : SFC                        Location : Vial 75
Injection Date  : 4/24/2015 3:33:05 PM
                                           Inj Volume : 5.000 µl
Acq. Method     : C:\CHEM32\1\METHODS\AGILENT_SFC6.M
Last changed    : 4/24/2015 3:10:21 PM by [redacted]
                  (modified after loading)
Analysis Method : C:\CHEM32\1\METHODS\AGILENT_SFC6.M
Last changed    : 4/27/2015 9:58:43 AM by [redacted]
                  (modified after loading)
Additional Info : Peak(s) manually integrated
```

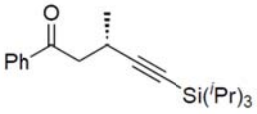

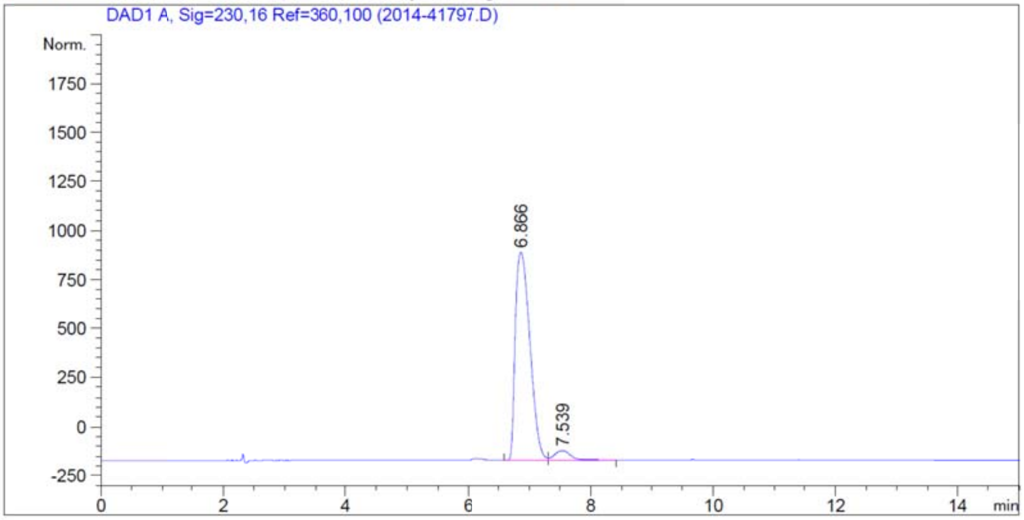


```
=====================================================================
                         Area Percent Report
=====================================================================

Sorted By             :      Signal
Multiplier            :      1.0000
Dilution              :      1.0000
Do not use Multiplier & Dilution Factor with ISTDs


Signal 1: DAD1 A, Sig=230,16 Ref=360,100
```

Peak #	RetTime [min]	Type	Width [min]	Area [mAU*s]	Height [mAU]	Area %
1	6.866	BV	0.2637	1.16703e4	714.10529	95.0300
2	7.539	VB	0.2943	610.35144	32.32950	4.9700

Totals : 1.22806e4 746.43479

Supplementary Figure 44. HPLC spectrum for *(S)*-1l.

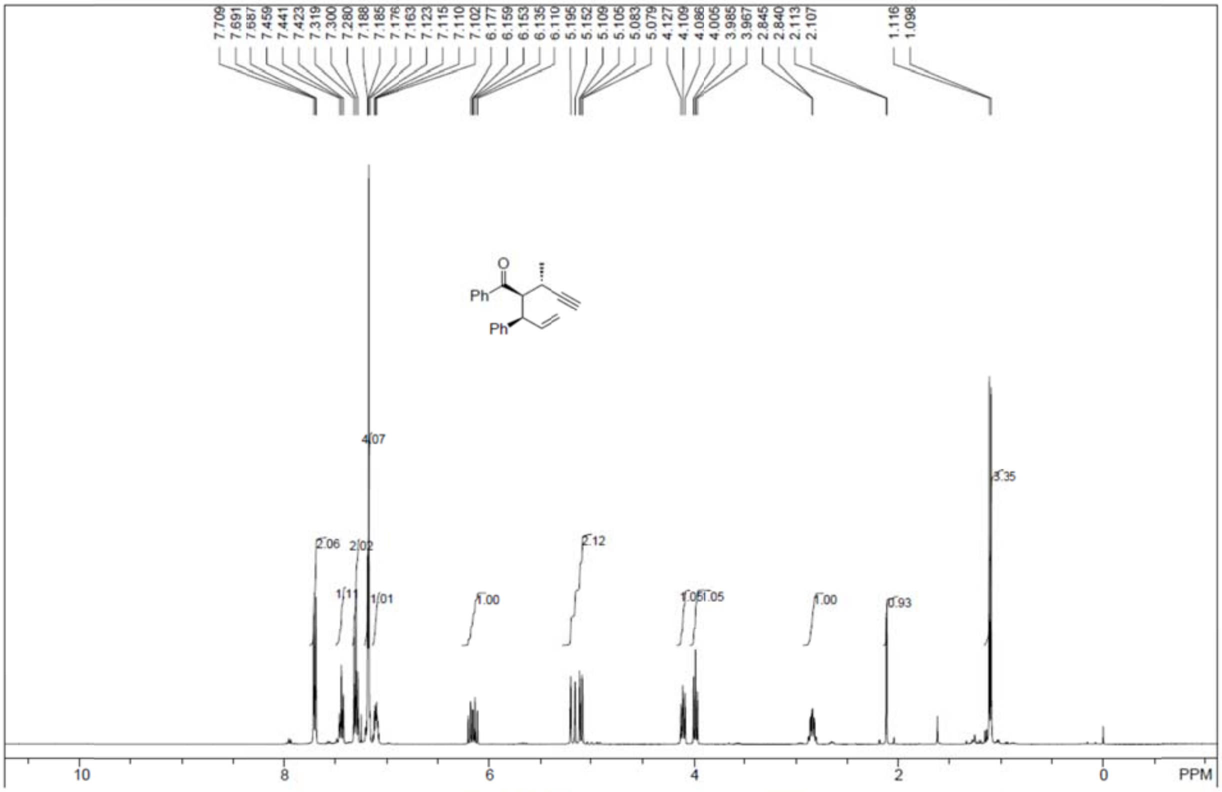

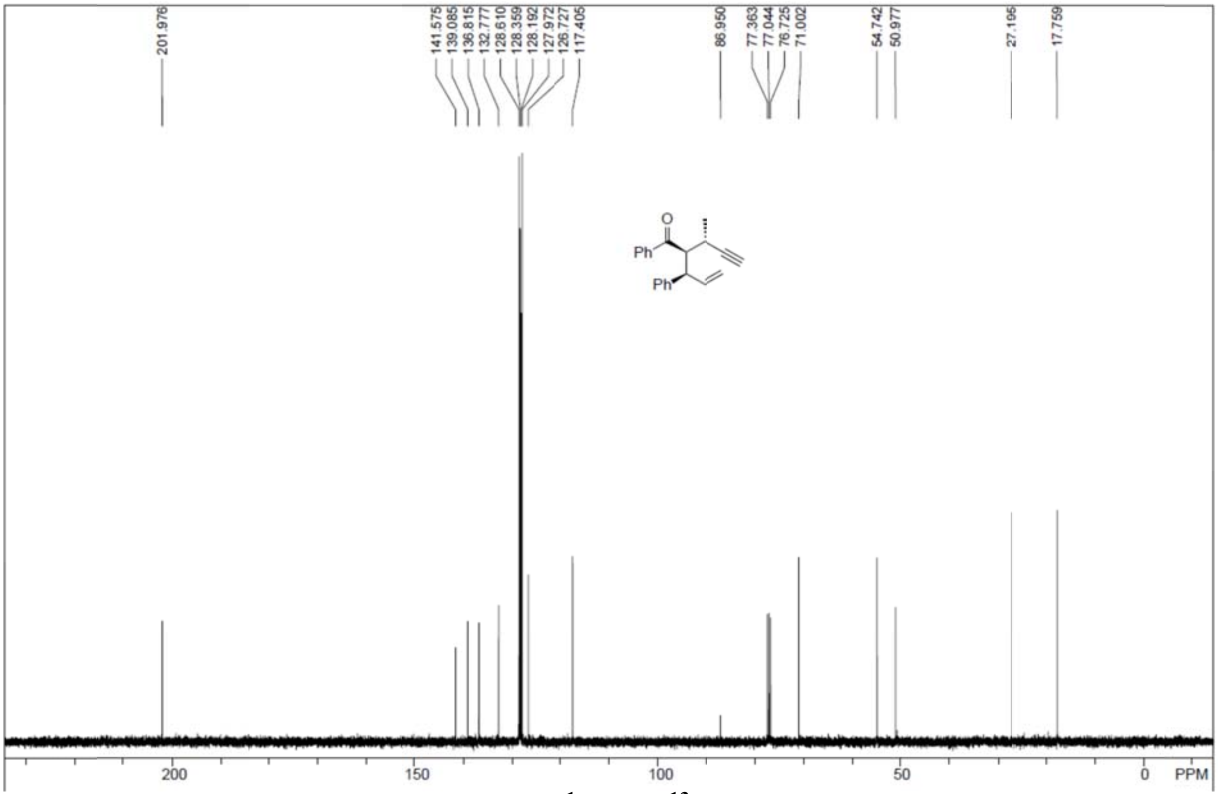

Supplementary Figure 45. ^{1}H **and** ^{13}C **NMR spectrum for 9.**

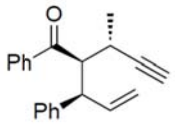

Acq. Operator : [redacted]
Sample Operator : [redacted]
Acq. Instrument : SFC Location : Vial 11
Injection Date : 1/23/2015 9:37:48 AM
Inj Volume : 5.000 µl
Acq. Method : C:\CHEM32\1\METHODS\AGILENT_SFC6.M
Last changed : 1/23/2015 9:27:42 AM by [redacted]
(modified after loading)
Analysis Method : C:\CHEM32\1\METHODS\AGILENT_SFC6.M
Last changed : 1/23/2015 3:35:00 PM by [redacted]
(modified after loading)
Additional Info : Peak(s) manually integrated

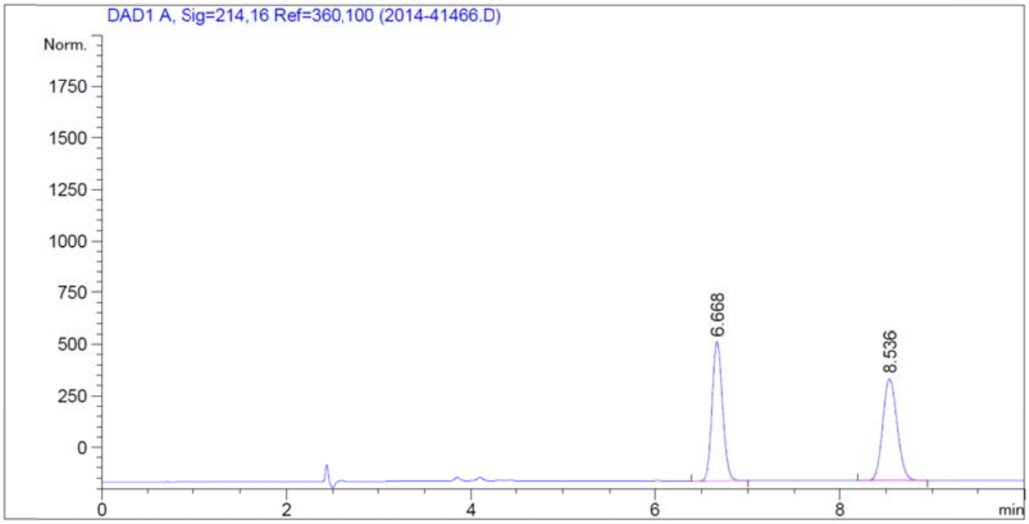

Area Percent Report

Sorted By : Signal
Multiplier : 1.0000
Dilution : 1.0000
Do not use Multiplier & Dilution Factor with ISTDs

Signal 1: DAD1 A, Sig=214,16 Ref=360,100

Peak #	RetTime [min]	Type	Width [min]	Area [mAU*s]	Height [mAU]	Area %
1	6.668	BB	0.1222	2539.32983	324.84845	49.7984
2	8.536	BB	0.1673	2559.88770	238.39680	50.2016

Totals : 5099.21753 563.24525

Supplementary Figure 46. HPLC spectrum for *rac*-9.

==

Acq. Operator : [redacted]
Sample Operator : [redacted]
Acq. Instrument : SFC Location : Vial 16
Injection Date : 1/23/2015 10:03:10 AM
Inj Volume : 5.000 µl
Acq. Method : C:\CHEM32\1\METHODS\AGILENT_SFC6.M
Last changed : 1/23/2015 9:27:42 AM by [redacted]
(modified after loading)
Analysis Method : C:\CHEM32\1\METHODS\AGILENT_SFC6.M
Last changed : 1/23/2015 3:39:51 PM by [redacted]
(modified after loading)
Additional Info : Peak(s) manually integrated

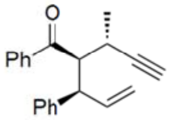

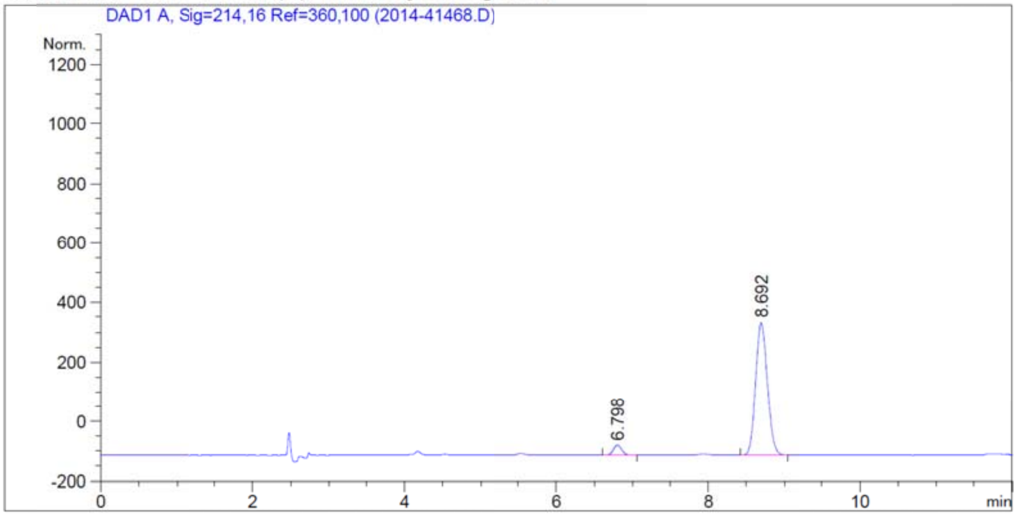

==
Area Percent Report
==

Sorted By : Signal
Multiplier : 1.0000
Dilution : 1.0000
Do not use Multiplier & Dilution Factor with ISTDs

Signal 1: DAD1 A, Sig=214,16 Ref=360,100

Peak #	RetTime [min]	Type	Width [min]	Area [mAU*s]	Height [mAU]	Area %
1	6.798	BB	0.1194	120.47047	15.54183	5.2092
2	8.692	BB	0.1678	2192.16797	204.92938	94.7908

Totals : 2312.63844 220.47121

Supplementary Figure 47. HPLC spectrum for 9.

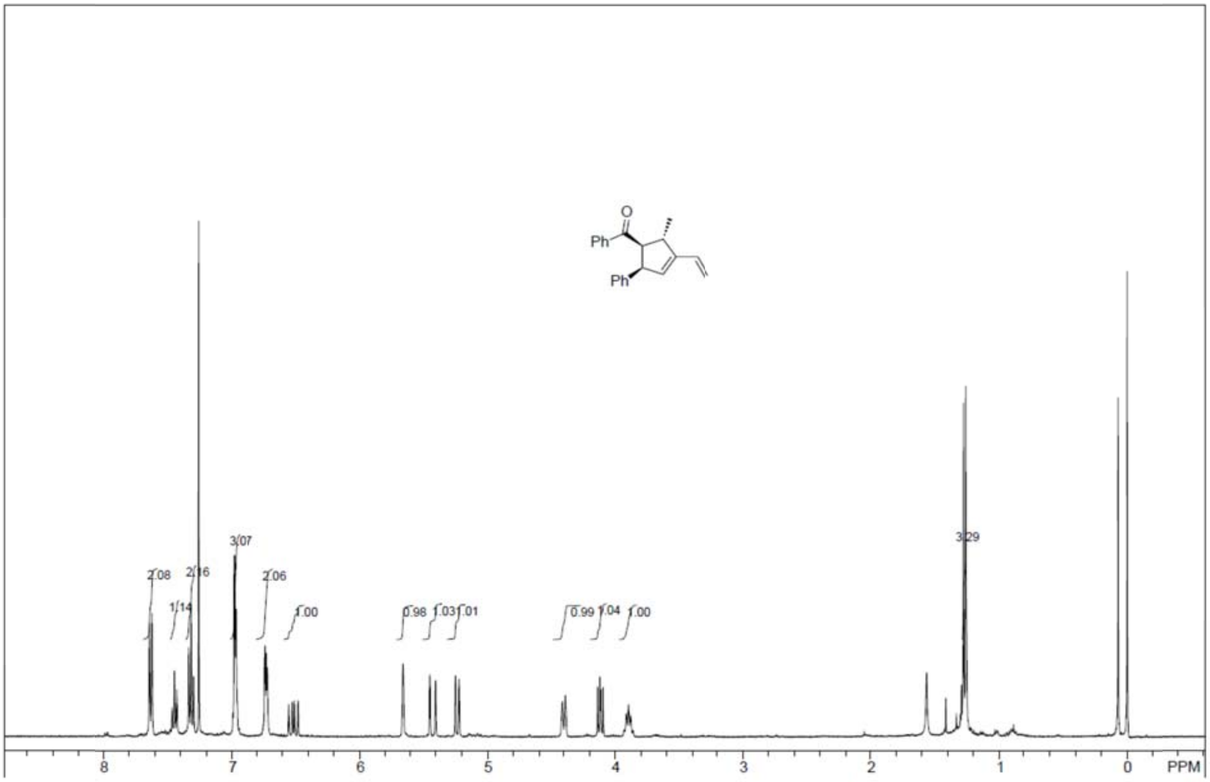

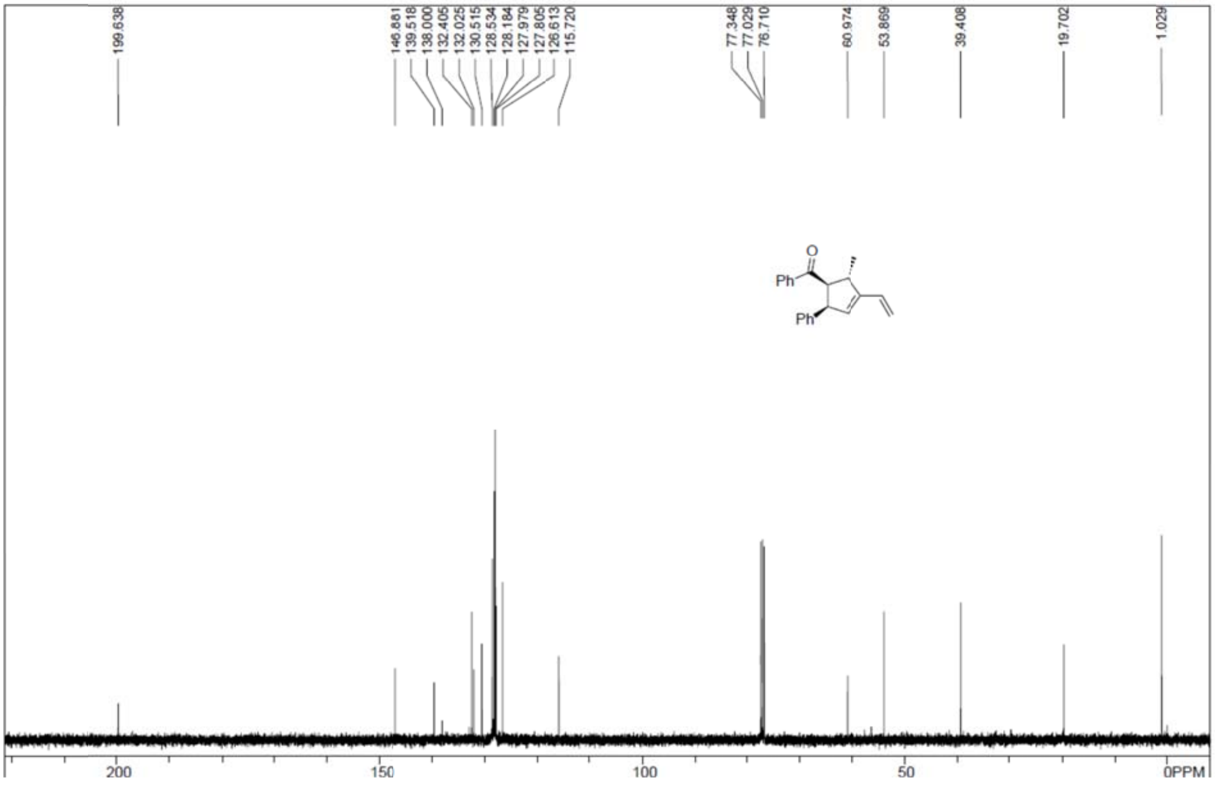

Supplementary Figure 48. ^{1}H and ^{13}C NMR spectrum for 10.

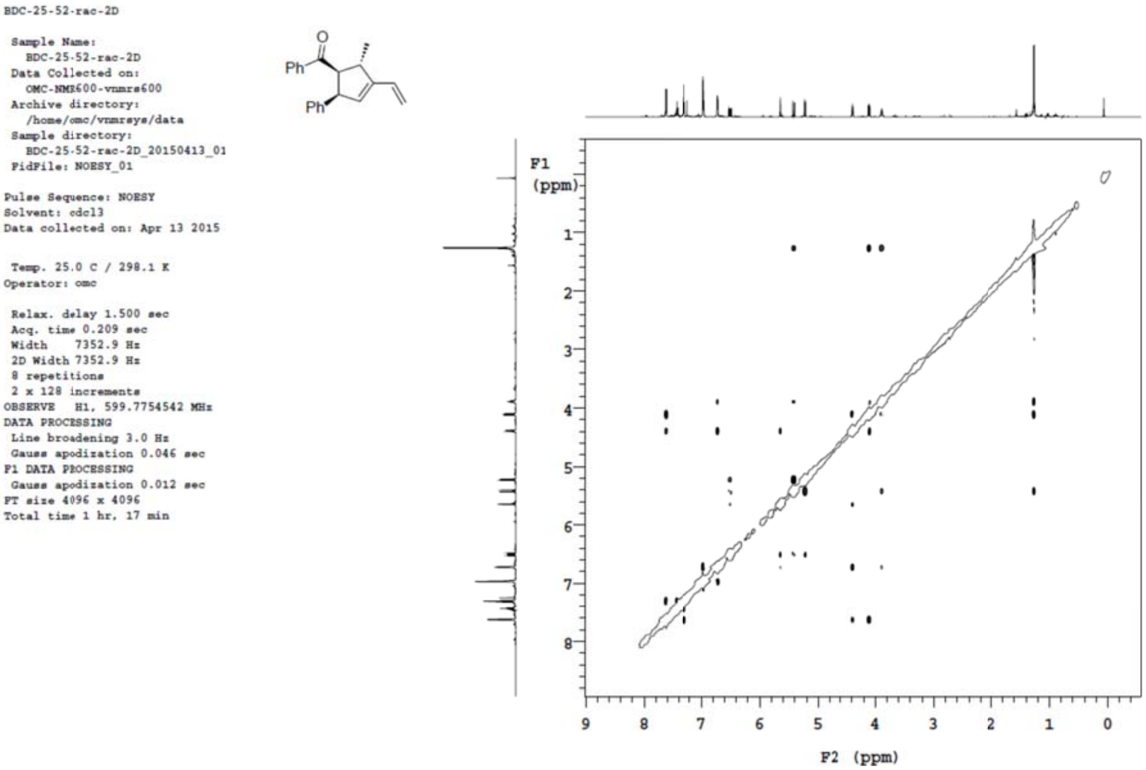

Supplementary Figure 49. NOESY spectrum for 10.

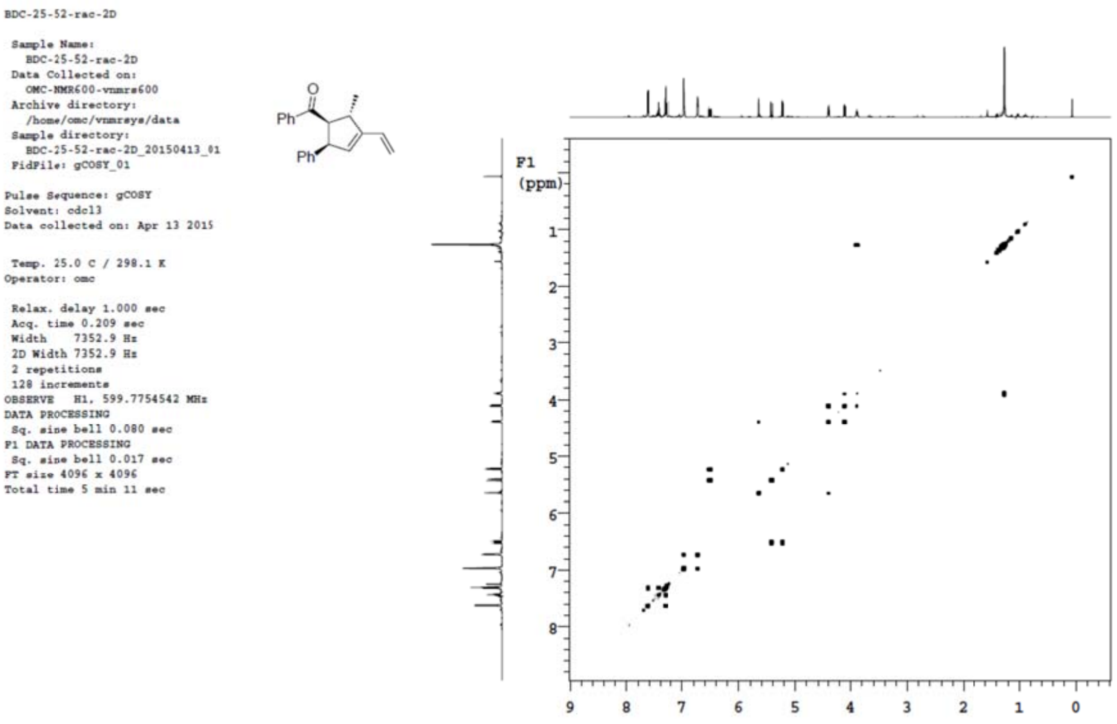

Supplementary Figure 50. gCOSY spectrum for 10.

==

Acq. Operator : [redacted]

Sample Operator : [redacted]

Acq. Instrument : SFC   Location : Vial 53

Injection Date : 4/16/2015 4:06:46 PM

Inj Volume : 5.000 µl

Acq. Method : C:\CHEM32\1\METHODS\AGILENT_SFC6.M

Last changed : 4/16/2015 3:44:43 PM by [redacted]

(modified after loading)

Analysis Method : C:\CHEM32\1\METHODS\AGILENT_SFC6.M

Last changed : 4/16/2015 4:20:22 PM by [redacted]

(modified after loading)

Additional Info : Peak(s) manually integrated

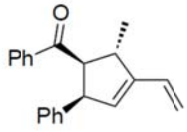

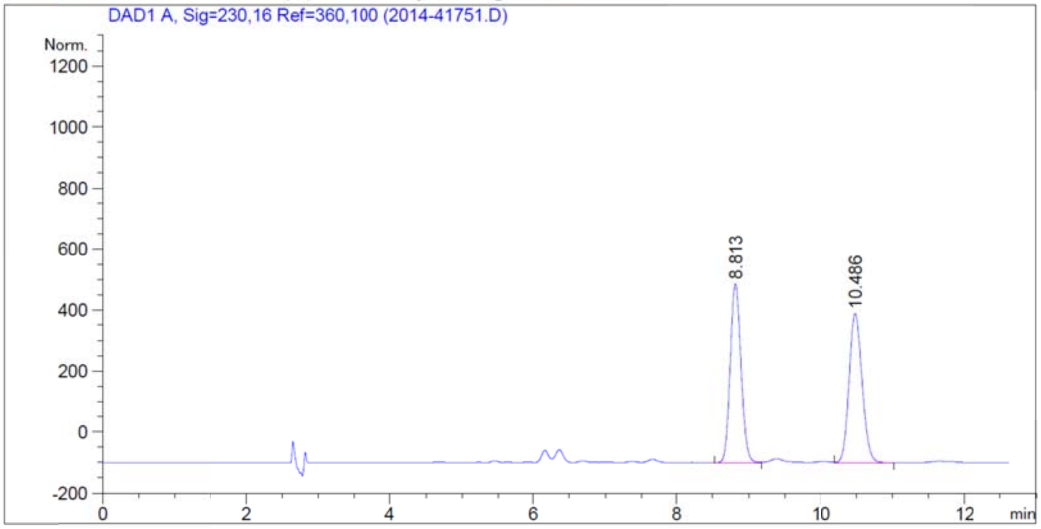

==

Area Percent Report

==

Sorted By : Signal

Multiplier : 1.0000

Dilution : 1.0000

Do not use Multiplier & Dilution Factor with ISTDs

Signal 1: DAD1 A, Sig=230,16 Ref=360,100

Peak #	RetTime [min]	Type	Width [min]	Area [mAU*s]	Height [mAU]	Area %
1	8.813	BV	0.1608	3785.69312	365.49261	49.8242
2	10.486	VB	0.1939	3812.40625	304.91330	50.1758

Totals : 7598.09937 670.40591

==

Supplementary Figure 51. HPLC spectrum for *rac*-10.

Acq. Operator : [redacted]
Sample Operator : [redacted]
Acq. Instrument : SFC   Location : Vial 81
Injection Date : 4/16/2015 3:50:10 PM
                                         Inj Volume : 5.000 µl
Acq. Method : C:\CHEM32\1\METHODS\AGILENT_SFC6.M
Last changed : 4/16/2015 3:44:43 PM by [redacted]
(modified after loading)
Analysis Method : C:\CHEM32\1\METHODS\AGILENT_SFC6.M
Last changed : 4/16/2015 4:16:03 PM by [redacted]
(modified after loading)
Additional Info : Peak(s) manually integrated

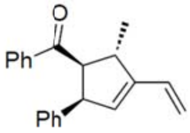

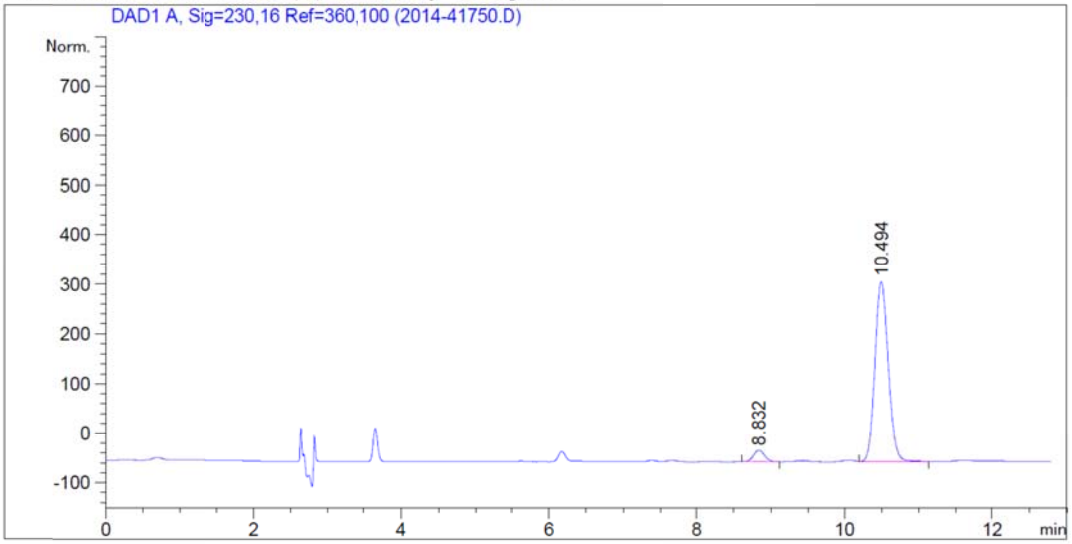

Area Percent Report

Sorted By : Signal
Multiplier : 1.0000
Dilution : 1.0000
Do not use Multiplier & Dilution Factor with ISTDs

Signal 1: DAD1 A, Sig=230,16 Ref=360,100

Peak #	RetTime [min]	Type	Width [min]	Area [mAU*s]	Height [mAU]	Area %
1	8.832	BB	0.1601	153.88518	15.07950	5.0652
2	10.494	VB	0.1929	2884.20679	232.18825	94.9348

Totals : 3038.09196 247.26775

Supplementary Figure 52. HPLC spectrum for 10.

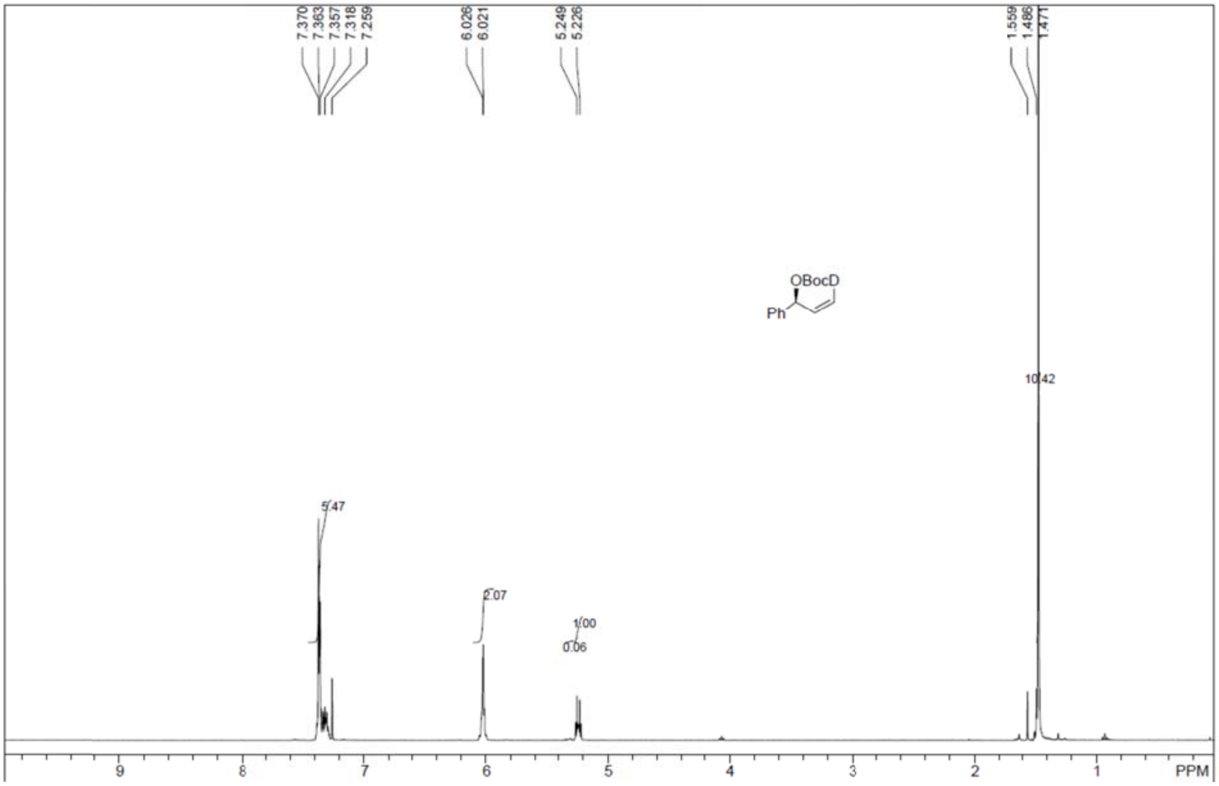

Supplementary Figure 53. ^{1}H NMR spectrum for (*S*)-(*Z*)-5.

==== Shimadzu LCsolution Analysis Report ====

C:\LabSolutions\Data\Project1\Project1\BDC\26\bdc-23-26-23s-RAC-.lcd

Acquired by	: Admin
Sample Name	: bdc-23-26-23s-RAC-
method	:OJ-H, 95/5,0.7, 214
Injection Volume	: 1 uL
Data File Name	: bdc-23-26-23s-RAC-.lcd
Method File Name	: 123.lcm
Report File Name	: Default.lcr
Data Acquired	: 2015-4-23 12:48:41
Data Processed	: 2015-4-23 13:00:59

<Chromatogram>

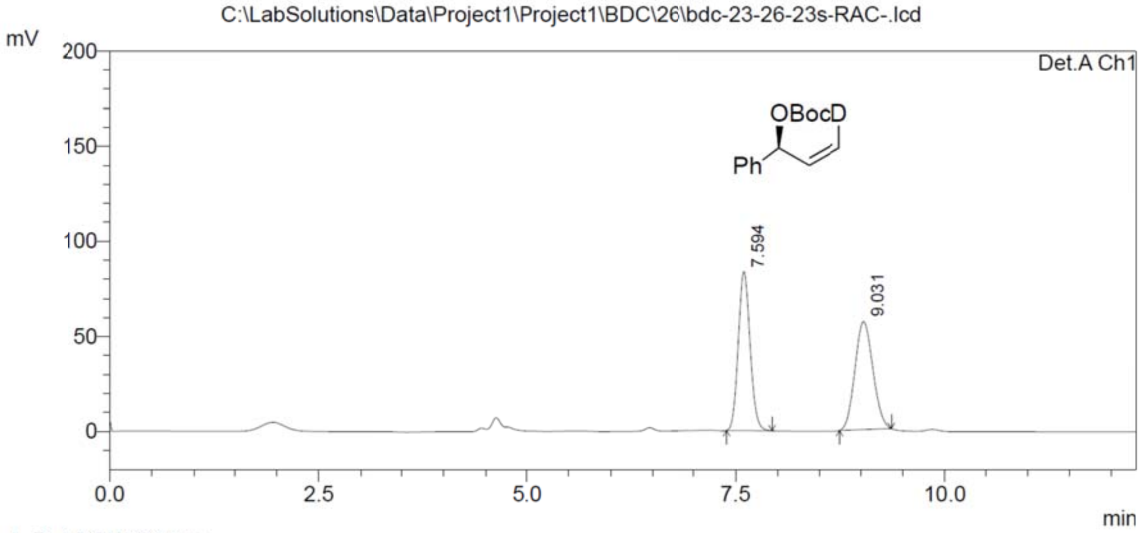

1 Det.A Ch1/214nm

PeakTable

Detector A Ch1 214nm

Peak#	Ret. Time	Area	Height	Area %
1	7.594	819833	83681	49.144
2	9.031	848387	57004	50.856
Total		1668220	140685	100.000

Supplementary Figure 54. HPLC spectrum for *rac*-5.

==== Shimadzu LCsolution Analysis Report ====

C:\LabSolutions\Data\Project1\Project1\BDC\26\bdc-23-26-23s-.lcd

Acquired by	: Admin
Sample Name	: bdc-23-26-23s-
method	:OJ-H, 95/5,0.7, 214
Injection Volume	: 1 uL
Data File Name	: bdc-23-26-23s-.lcd
Method File Name	: 123.lcm
Report File Name	: Default.lcr
Data Acquired	: 2015-4-23 13:02:03
Data Processed	: 2015-4-23 13:18:21

<Chromatogram>

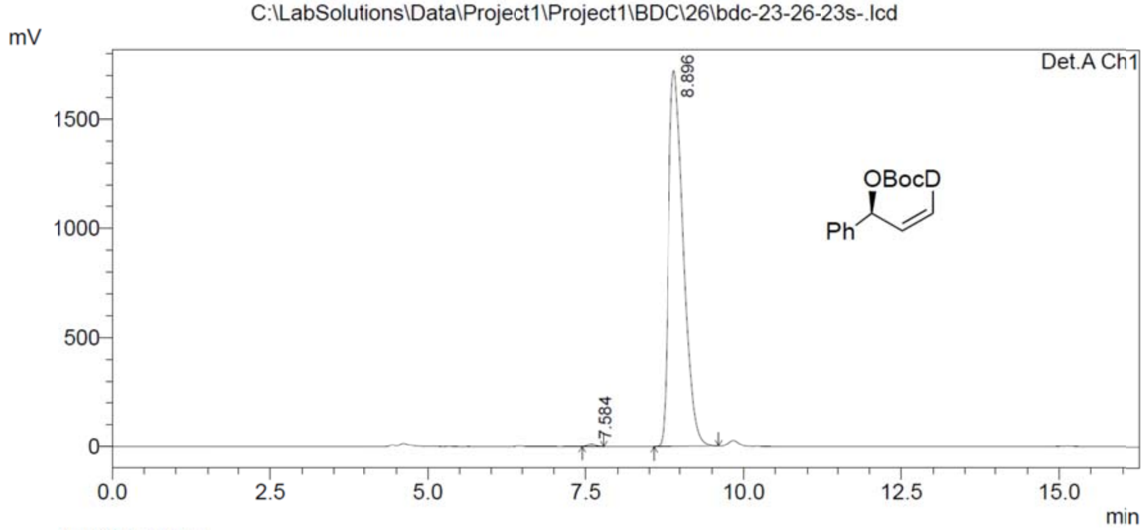

1 Det.A Ch1/214nm

PeakTable

Detector A Ch1 214nm

Peak#	Ret. Time	Area	Height	Area %
1	7.584	98562	10977	0.344
2	8.896	28532434	1721455	99.656
Total		28630996	1732432	100.000

Supplementary Figure 55. HPLC spectrum for (*S*)-(*Z*)-5.

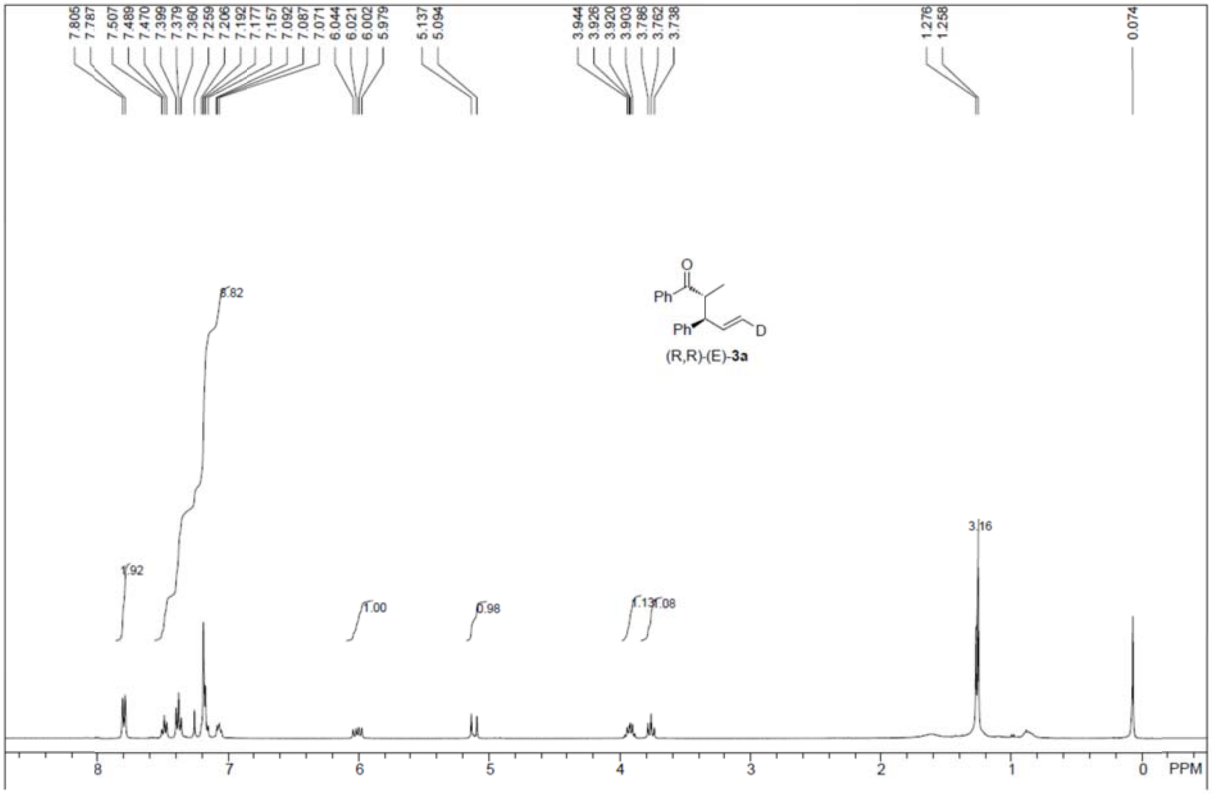

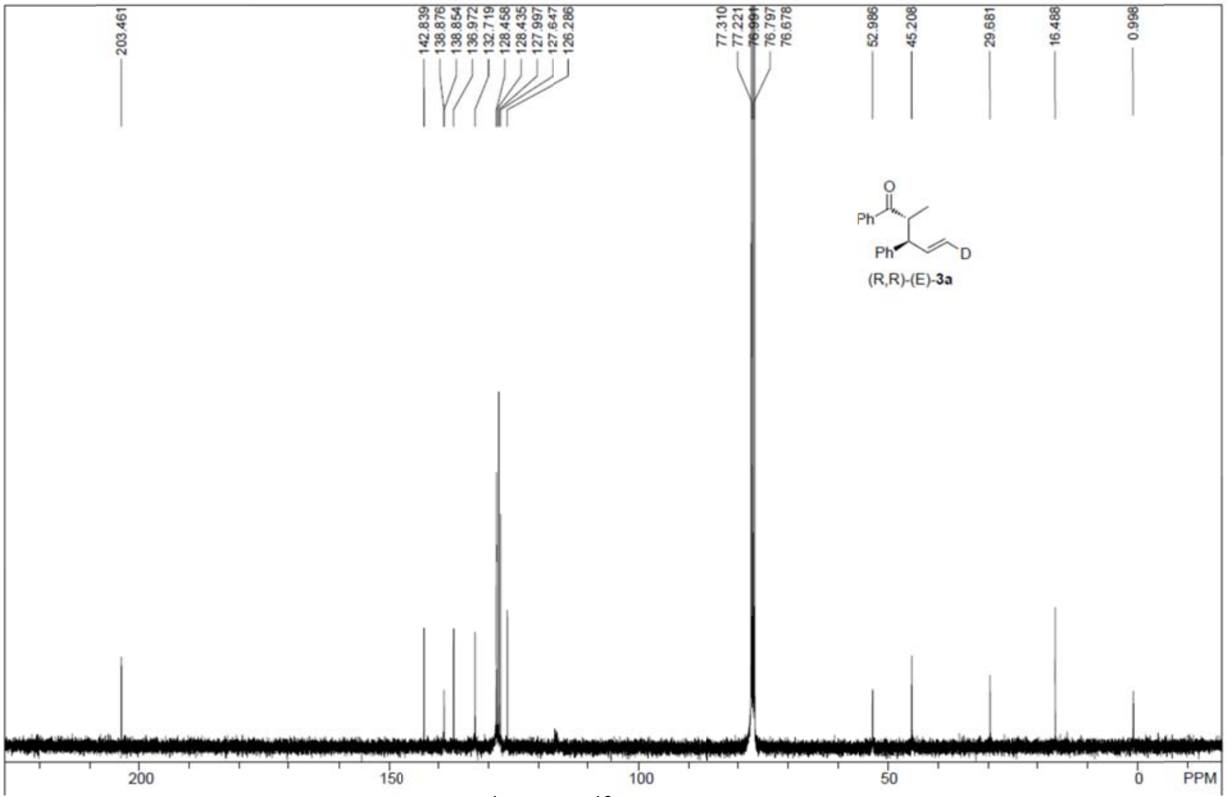

Supplementary Figure 56. ^{1}H and ^{13}C NMR spectrum for (*R,R*)-(*E*)-3a.

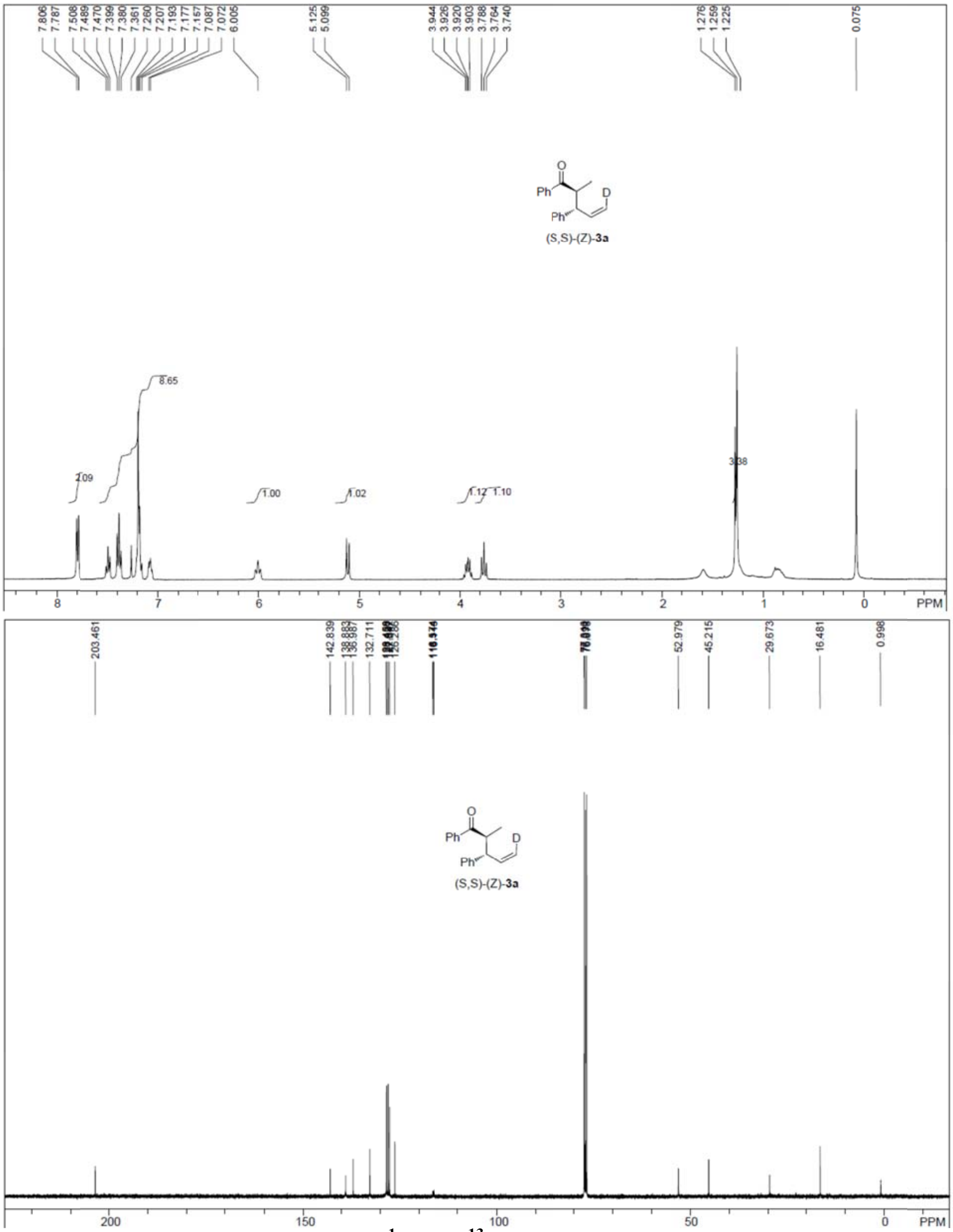

Supplementary Figure 57. ^{1}H and ^{13}C NMR spectrum for (*S,S*)-(*Z*)-3a.

==== Shimadzu LCsolution Analysis Report ====

D:\LabSolutions\Data\bdc\26\bdc-26-99d-rac.lcd

Acquired by	: Admin
Sample Name	: bdc-26-99d-rac
method	:oj-H,90/10,0.7,214
Injection Volume	: 1500 uL
Data File Name	: bdc-26-99d-rac.lcd
Method File Name	: 1234.lcm
Report File Name	: Default.lcr
Data Acquired	: 2014-6-8 20:28:52
Data Processed	: 2014-6-8 21:17:48

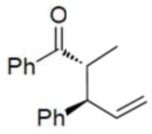

<Chromatogram>

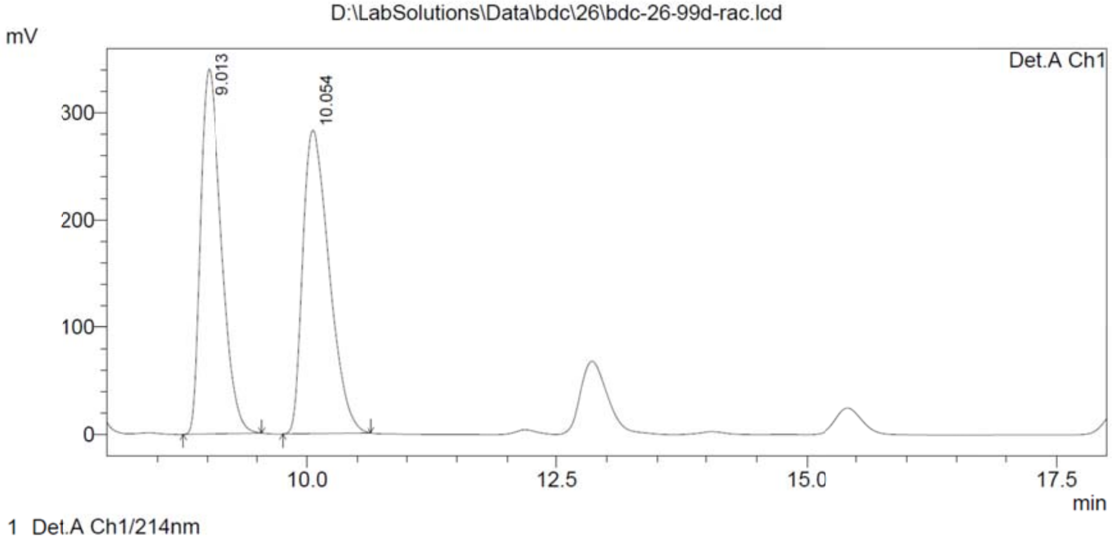

PeakTable

Detector A Ch1 214nm

Peak#	Ret. Time	Area	Height	Area %
1	9.013	5056260	340225	49.508
2	10.054	5156726	283013	50.492
Total		10212986	623238	100.000

Supplementary Figure 58. HPLC spectrum for *rac*-3a.

==== Shimadzu LCsolution Analysis Report ====

D:\LabSolutions\Data\bdc\26\bdc-26-99d-1-hplc.lcd

Acquired by	: Admin
Sample Name	: bdc-26-99d-1
method	:oj-H,90/10,0.7,214
Injection Volume	: 1500 uL
Data File Name	: bdc-26-99d-1-hplc.lcd
Method File Name	: 1234.lcm
Report File Name	: Default.lcr
Data Acquired	: 2014-6-8 19:35:36
Data Processed	: 2014-6-8 19:57:59

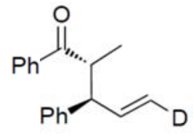

(R,R)-(E)-**3a**

<Chromatogram>

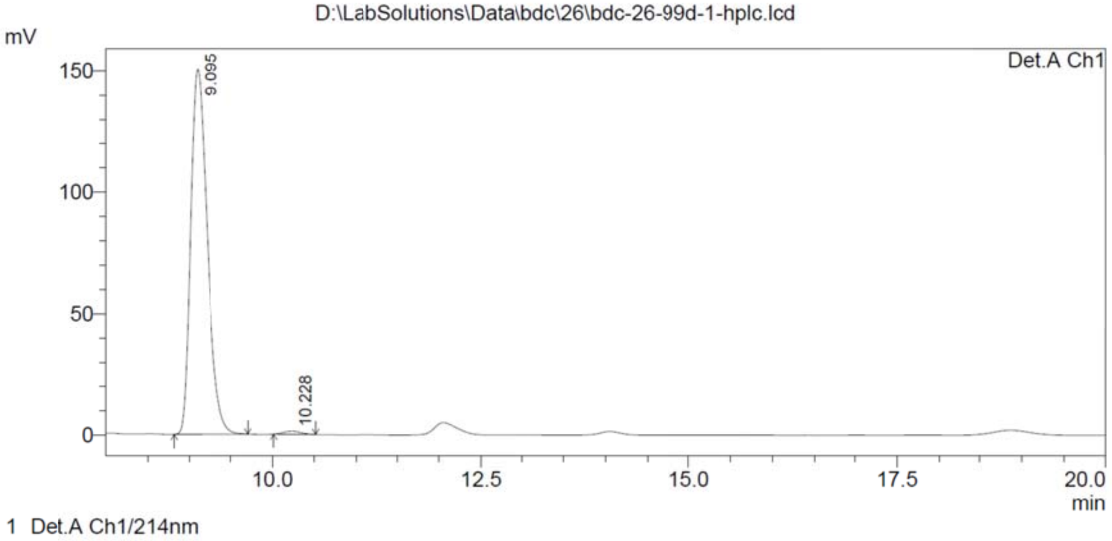

PeakTable

Detector A Ch1 214nm

Peak#	Ret. Time	Area	Height	Area %
1	9.095	2113957	150329	99.119
2	10.228	18786	1308	0.881
Total		2132743	151637	100.000

Supplementary Figure 59. HPLC spectrum for (*R*,*R*)-(*E*)-3a.

==== Shimadzu LCsolution Analysis Report ====

D:\LabSolutions\Data\bdc\26\bdc-26-99d-2-hplc.lcd

Acquired by	: Admin
Sample Name	: bdc-26-99d-2-hplc
method	:oj-H,90/10,0.7,214
Injection Volume	: 1500 uL
Data File Name	: bdc-26-99d-2-hplc.lcd
Method File Name	: 1234.lcm
Report File Name	: Default.lcr
Data Acquired	: 2014-6-8 20:00:38
Data Processed	: 2014-6-8 20:25:50

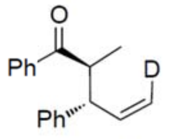

(S,S)-(Z)-**3a**

<Chromatogram>

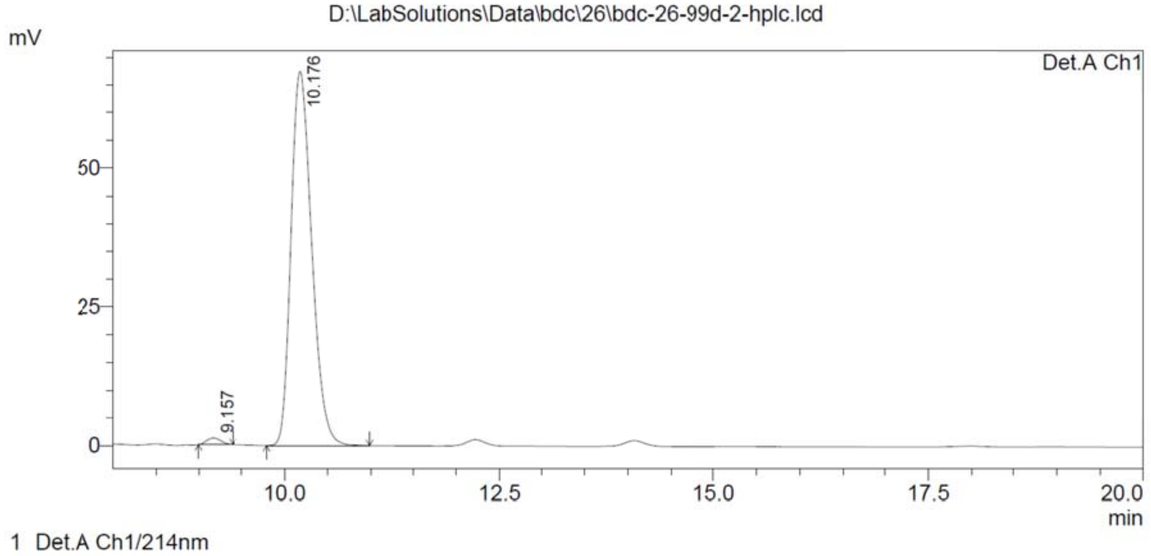

PeakTable

Detector A Ch1 214nm

Peak#	Ret. Time	Area	Height	Area %
1	9.157	13722	1192	1.206
2	10.176	1124023	67464	98.794
Total		1137745	68656	100.000

Supplementary Figure 60. HPLC spectrum for (*S,S*)-(*Z*)-3a.

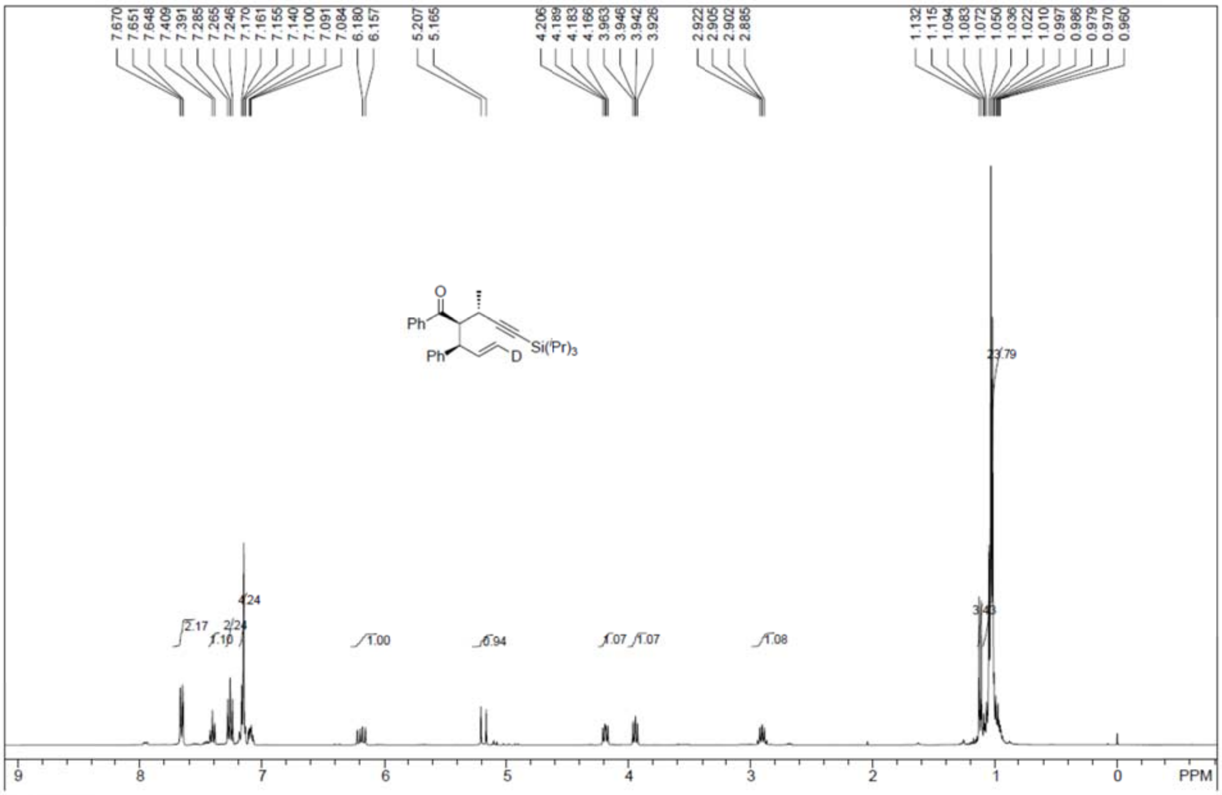

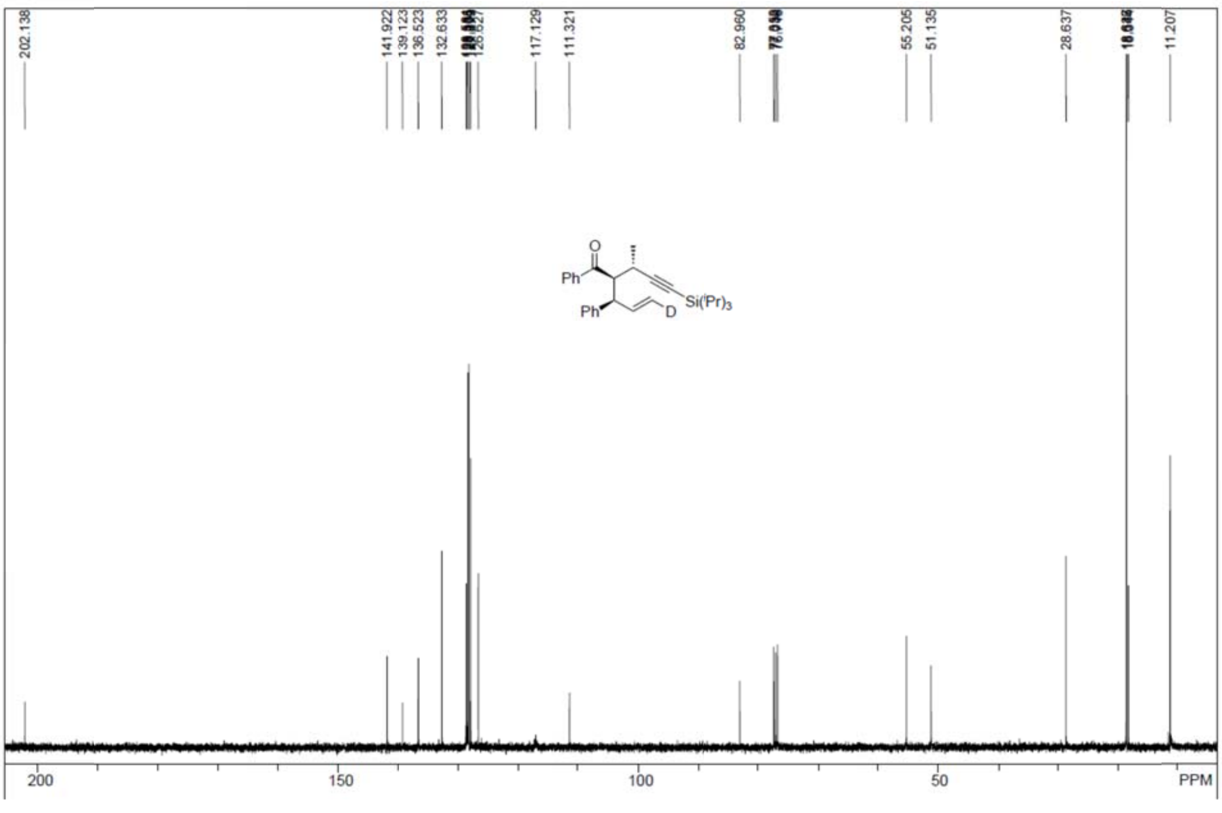

Supplementary Figure 61. ^{1}H and ^{13}C NMR spectrum for (*3R,4S,5S*)-(*E*)-3l.

==

Acq. Operator : [redacted]

Sample Operator : [redacted]

Acq. Instrument : SFC Location : Vial 72

Injection Date : 4/24/2015 11:11:14 AM

Inj Volume : 5.000 µl

Acq. Method : C:\CHEM32\1\METHODS\AGILENT_SFC6.M

Last changed : 4/24/2015 10:37:15 AM by [redacted]

(modified after loading)

Analysis Method : C:\CHEM32\1\METHODS\AGILENT_SFC6.M

Last changed : 4/28/2015 2:28:48 PM by [redacted]

(modified after loading)

Additional Info : Peak(s) manually integrated

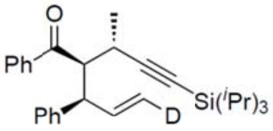

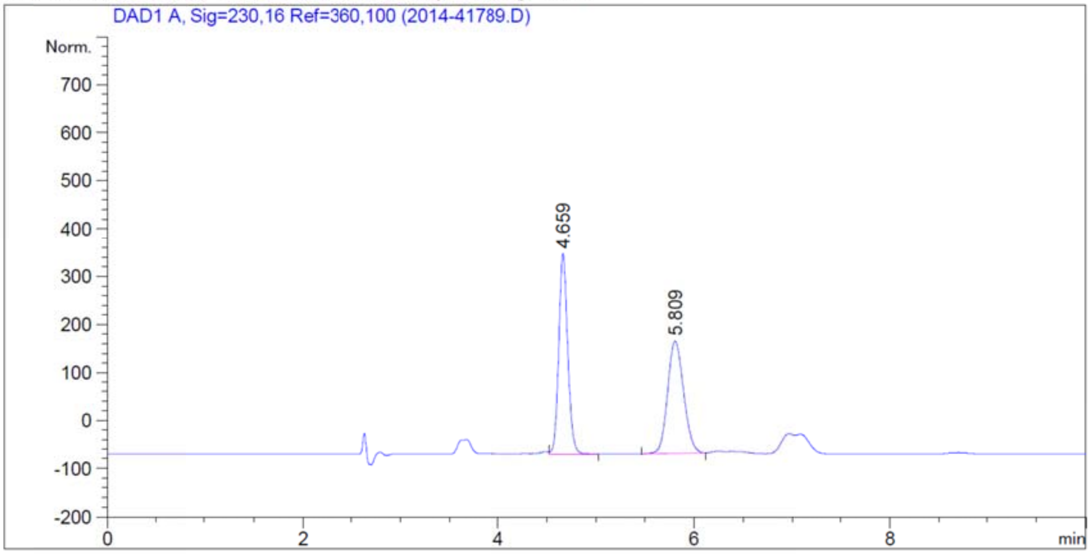

==

Area Percent Report

==

Sorted By : Signal

Multiplier : 1.0000

Dilution : 1.0000

Do not use Multiplier & Dilution Factor with ISTDs

Signal 1: DAD1 A, Sig=230,16 Ref=360,100

Peak #	RetTime [min]	Type	Width [min]	Area [mAU*s]	Height [mAU]	Area %
1	4.659	VB	0.0956	1697.59399	269.25052	50.0299
2	5.809	BB	0.1750	1695.56311	151.01114	49.9701
Totals :				3393.15710	420.26166	

Supplementary Figure 62. HPLC spectrum for *rac*-(*E*)-3l.

===

Acq. Operator : [redacted]
Sample Operator : [redacted]
Acq. Instrument : SFC Location : Vial 73
Injection Date : 4/24/2015 10:57:13 AM
Inj Volume : 5.000 µl
Acq. Method : C:\CHEM32\1\METHODS\AGILENT_SFC6.M
Last changed : 4/24/2015 10:37:15 AM by [redacted]
(modified after loading)
Analysis Method : C:\CHEM32\1\METHODS\AGILENT_SFC6.M
Last changed : 4/28/2015 2:30:48 PM by [redacted]
(modified after loading)
Additional Info : Peak(s) manually integrated

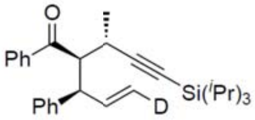

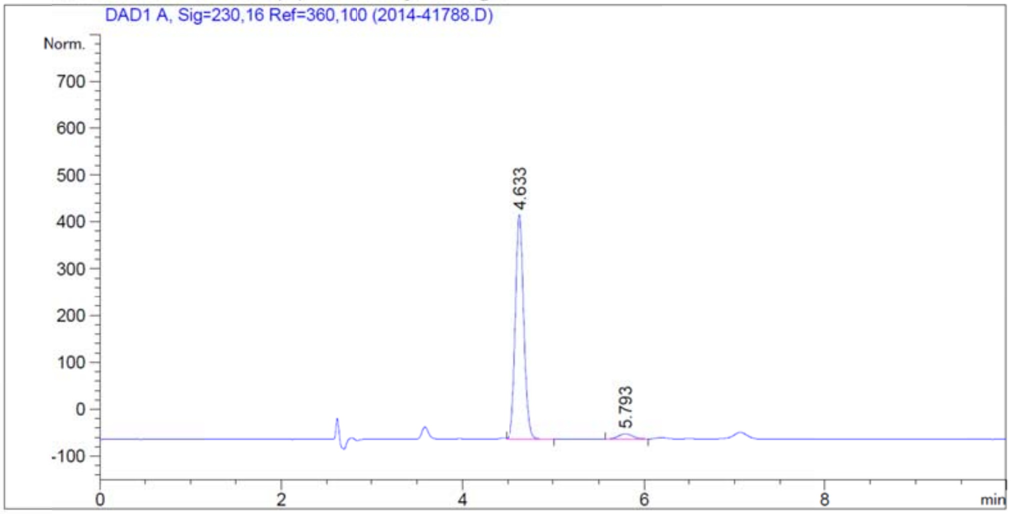

===
Area Percent Report
===

Sorted By : Signal
Multiplier : 1.0000
Dilution : 1.0000
Do not use Multiplier & Dilution Factor with ISTDs

Signal 1: DAD1 A, Sig=230,16 Ref=360,100

Peak #	RetTime [min]	Type	Width [min]	Area [mAU*s]	Height [mAU]	Area %
1	4.633	VB	0.0994	2226.16846	344.66232	96.1309
2	5.793	BB	0.1727	89.59998	8.05881	3.8691

Totals : 2315.76843 352.72113

Supplementary Figure 63. HPLC spectrum for (*3R*,*4S*,*5S*)-(*E*)-3l.

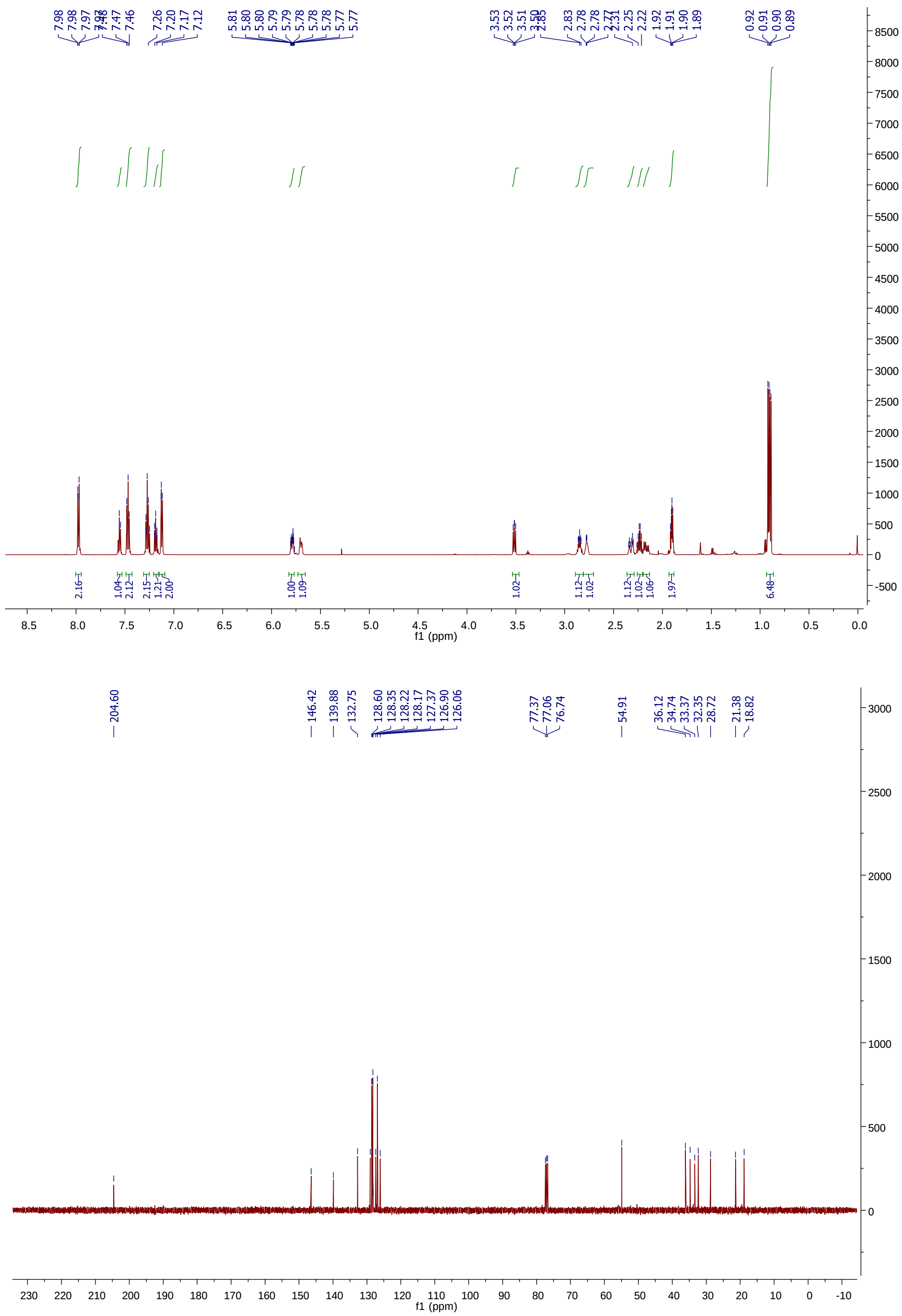

Supplementary Figure 64. ^{1}H and ^{13}C NMR spectrum for 12.

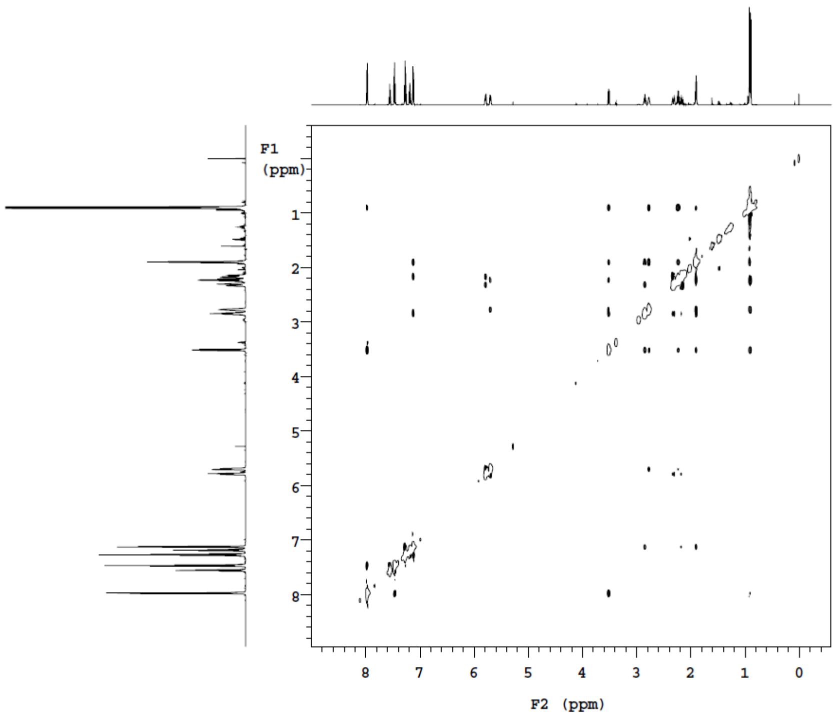

Supplementary Figure 65. NOESY spectrum for 12.

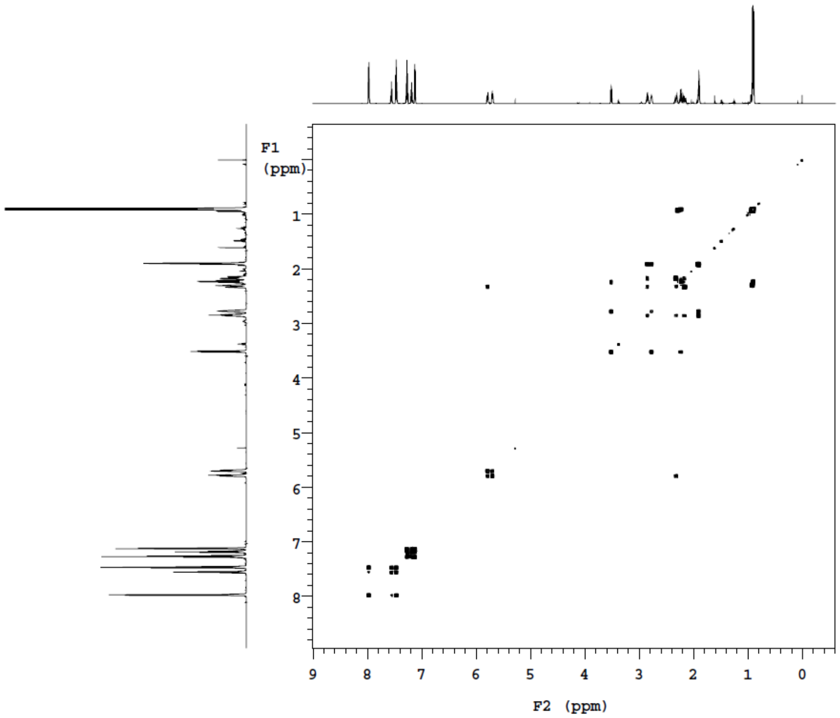

Supplementary Figure 66. gCOSY spectrum for 12.

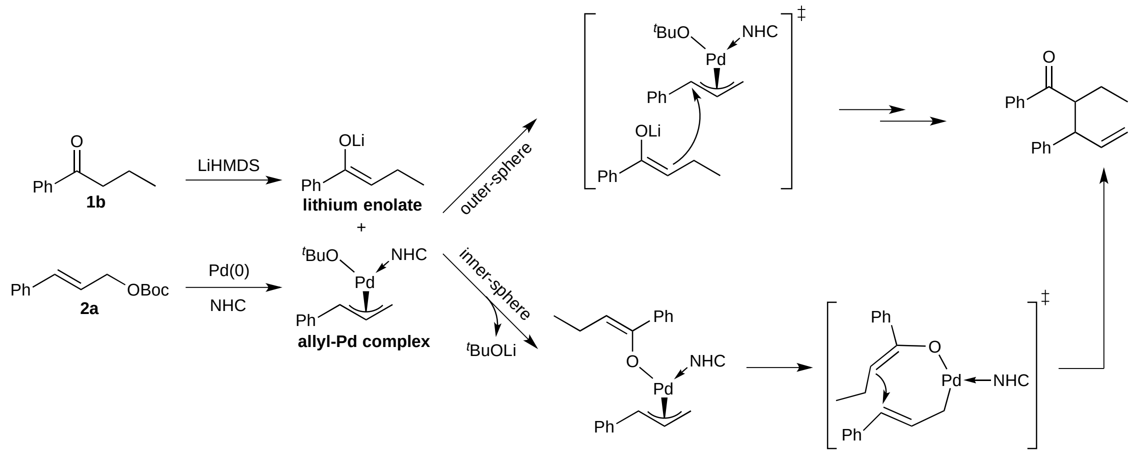

Supplementary Figure 67. *The proposed process of reaction of **1b** with **2a** in the presence of base, Pd and ligand, leading to the branched product.*

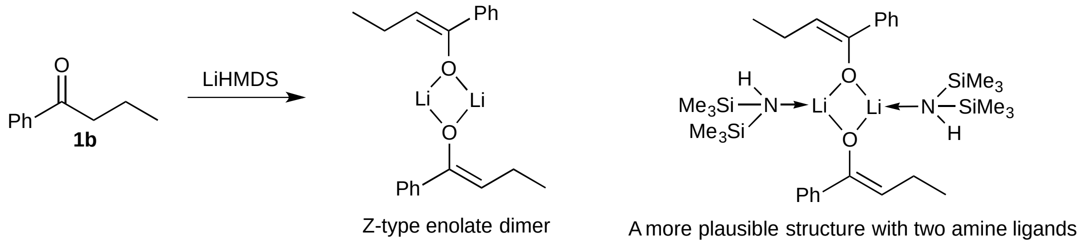

Supplementary Figure 68. *Proposed dimer structure of the lithium enolated, generated from **1b** by LiHMDS.*

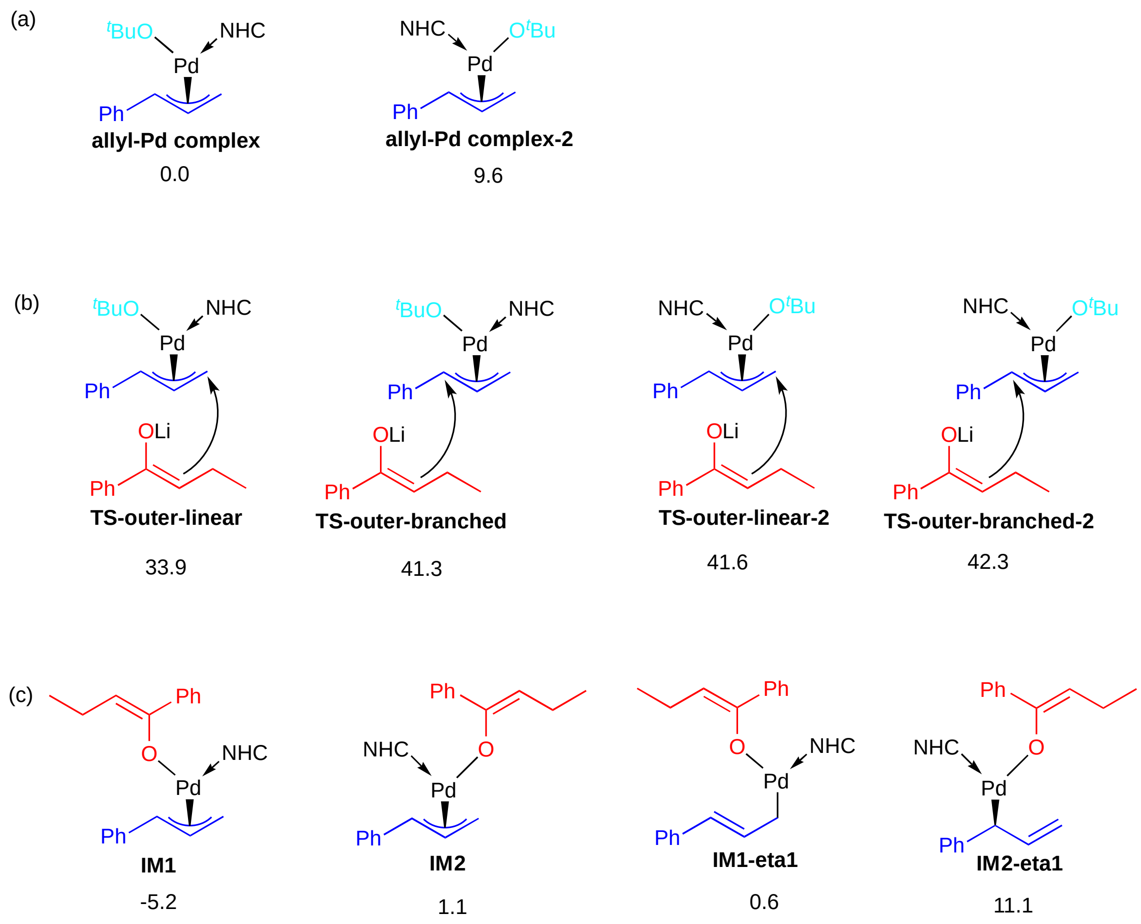

Supplementary Figure 69. *Calculated relative free energies (in kcal/mol) of (a) Two configurations of the* ***allyl-Pd complex****; (b) four transition states of the outer-sphere pathways (linear and branched for each of* ***allyl-Pd complex-1*** *and* ***allyl-Pd complex-2****); (c) four* η^1*-allyl-Pd complex intermediates in the inner-sphere pathways.*

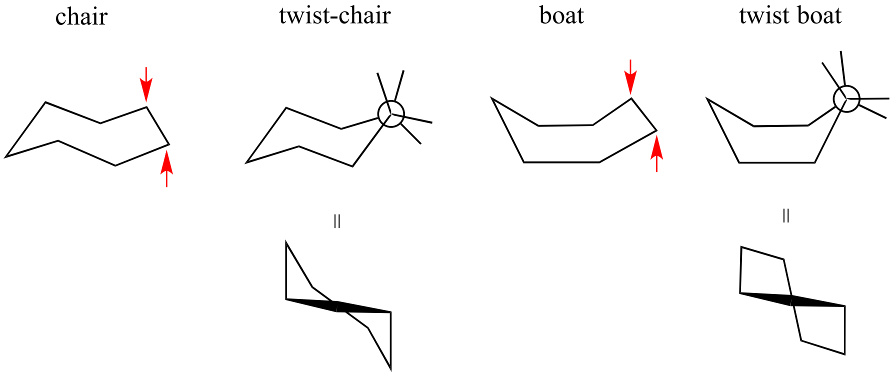

Supplementary Figure 70. *Conformations of cycloheptane.*

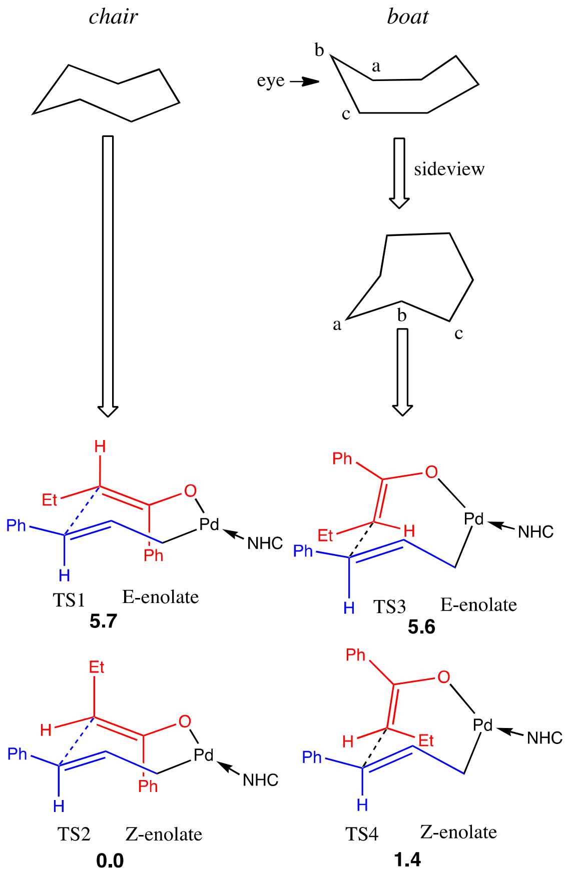

Supplementary Figure 71. *Construction of transition state structures (TS-inner-branched) for substrate* ***1b*** *from cycloheptane. The number at the bottom of each transition state structure show its energy (ΔG_{sol} in kcal/mol) relative to* ***TS2****.*

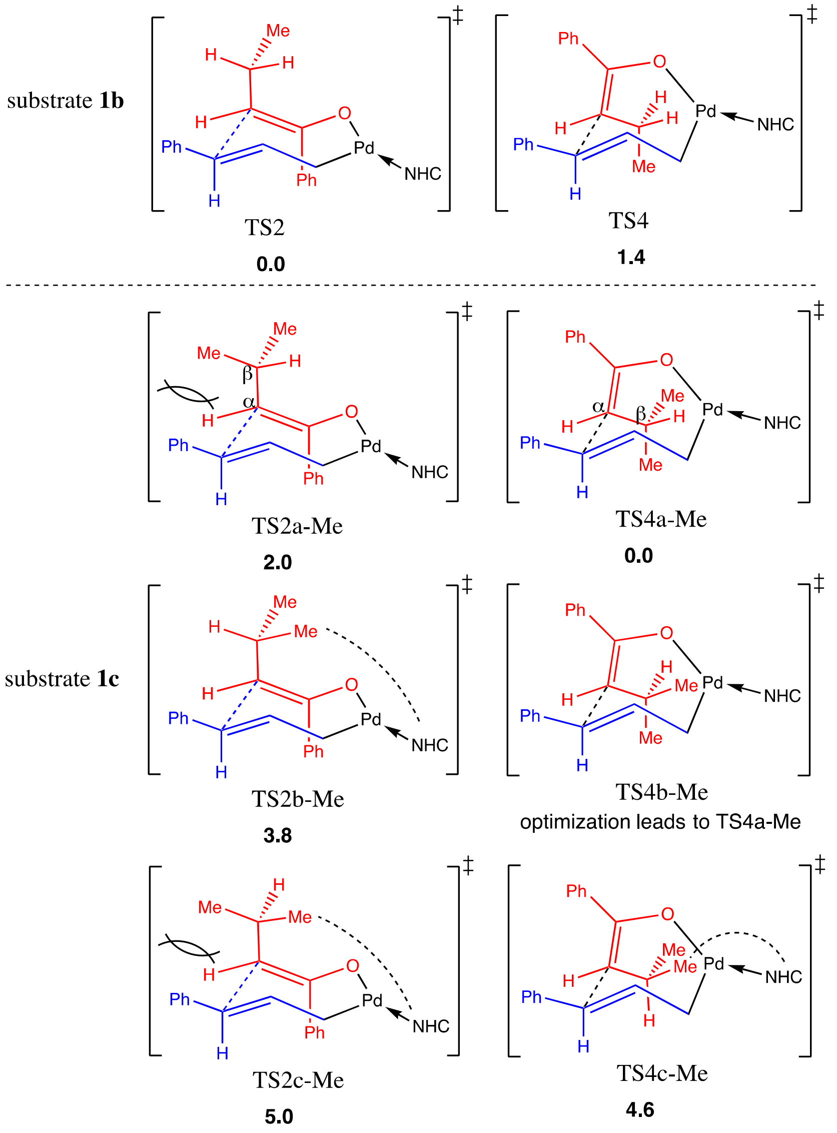

Supplementary Figure 72. *Calculated energies of transition state structures for* ***1b*** *and* ***1c****. The number at the bottom of each transition state structure show its energy* ΔG_{sol} *(in kcal/mol).*

substrate **1k**

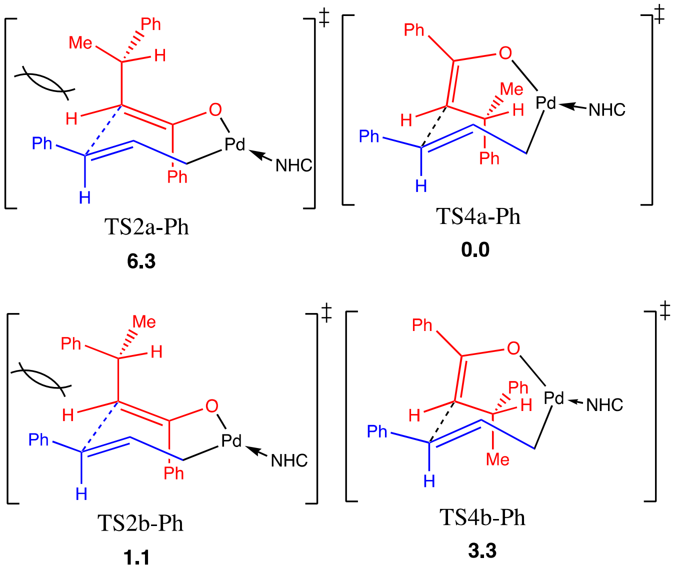

Supplementary Figure 73. *Calculated energies of transition state structures for* ***1k****. The number at the bottom of each transition state structure show its energy (ΔG$_{sol}$ in kcal/mol)*

Supplementary Tables

Supplementary Table 1. ORTEP Diagram of 3c.

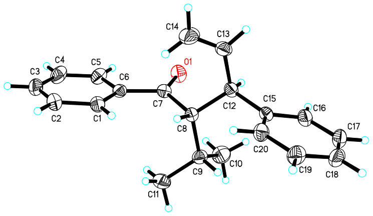

Identification code	**3c**	
Empirical formula	C21 H24 O	
Formula weight	292.40	
Temperature	293(2) K	
Wavelength	0.71073 Å	
Crystal system	Orthorhombic	
Space group	P b c a	
Unit cell dimensions	a = 7.3682(5) Å	α= 90°.
	b = 11.4357(8) Å	β= 90°.
	c = 41.662(3) Å	γ = 90°.
Volume	3510.5(4) $Å^3$	
Z	8	
Density (calculated)	1.106 Mg/m^3	
Absorption coefficient	0.066 mm^{-1}	
F(000)	1264	
Crystal size	0.188 x 0.145 x 0.113 mm^3	
Theta range for data collection	1.955 to 25.498°.	
Index ranges	-8<=h<=7, -13<=k<=13, -42<=l<=50	
Reflections collected	19148	
Independent reflections	3262 [R(int) = 0.0729]	
Completeness to theta = 25.242°	100.0 %	
Absorption correction	Semi-empirical from equivalents	
Max. and min. transmission	0.7457 and 0.6260	
Refinement method	Full-matrix least-squares on F^2	
Data / restraints / parameters	3262 / 28 / 223	
Goodness-of-fit on F^2	1.021	
Final R indices [I>2sigma(I)]	R1 = 0.0633, wR2 = 0.1666	
R indices (all data)	R1 = 0.1250, wR2 = 0.1965	
Extinction coefficient	n/a	
Largest diff. peak and hole	0.170 and -0.175 e.$Å^{-3}$	

Supplementary Table 2. ORTEP Diagram of 3f.

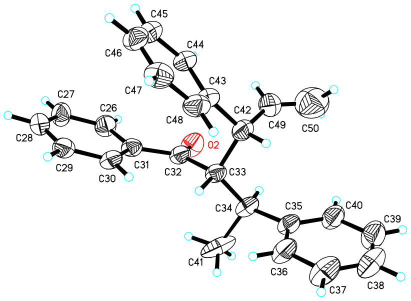

Identification code	**3f**	
Empirical formula	C25 H24 O	
Formula weight	340.44	
Temperature	293(2) K	
Wavelength	0.71073 Å	
Crystal system	Triclinic	
Space group	P -1	
Unit cell dimensions	a = 8.0107(13) Å	α= 90.066(4)°.
	b = 11.3987(18) Å	β= 97.728(4)°.
	c = 21.671(3) Å	γ = 91.878(4)°.
Volume	1959.8(5) $Å^3$	
Z	4	
Density (calculated)	1.154 Mg/m^3	
Absorption coefficient	0.068 mm^{-1}	
F(000)	728	
Crystal size	0.211 x 0.156 x 0.121 mm^3	
Theta range for data collection	1.897 to 25.050°.	
Index ranges	-9<=h<=9, -13<=k<=13, -23<=l<=25	
Reflections collected	11038	
Independent reflections	6946 [R(int) = 0.0389]	
Completeness to theta = 25.242°	97.9 %	
Absorption correction	Semi-empirical from equivalents	
Max. and min. transmission	0.7457 and 0.5494	
Refinement method	Full-matrix least-squares on F^2	
Data / restraints / parameters	6946 / 62 / 502	
Goodness-of-fit on F^2	1.039	
Final R indices [I>2sigma(I)]	R1 = 0.0838, wR2 = 0.2398	
R indices (all data)	R1 = 0.1705, wR2 = 0.2985	
Extinction coefficient	n/a	
Largest diff. peak and hole	0.666 and -0.428 e.$Å^{-3}$	

Supplementary Table 3. ORTEP Diagram of 7.

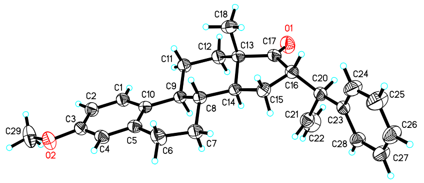

Identification code	**7**	
Empirical formula	C28 H32 O2	
Formula weight	400.53	
Temperature	293(2) K	
Wavelength	0.71073 Å	
Crystal system	Orthorhombic	
Space group	P 21 21 21	
Unit cell dimensions	a = 6.6085(6) Å	α= 90°.
	b = 10.5975(10) Å	β= 90°.
	c = 31.771(3) Å	γ = 90°.
Volume	2225.0(4) Å^3	
Z	4	
Density (calculated)	1.196 Mg/m^3	
Absorption coefficient	0.073 mm^{-1}	
F(000)	864	
Crystal size	0.211 x 0.165 x 0.123 mm^3	
Theta range for data collection	2.026 to 25.999°.	
Index ranges	-8<=h<=8, -13<=k<=12, -39<=l<=34	
Reflections collected	13581	
Independent reflections	4357 [R(int) = 0.0372]	
Completeness to theta = 25.242°	100.0 %	
Absorption correction	Semi-empirical from equivalents	
Max. and min. transmission	0.7457 and 0.6397	
Refinement method	Full-matrix least-squares on F^2	
Data / restraints / parameters	4357 / 0 / 274	
Goodness-of-fit on F^2	0.991	
Final R indices [I>2sigma(I)]	R1 = 0.0438, wR2 = 0.1117	
R indices (all data)	R1 = 0.0641, wR2 = 0.1235	
Absolute structure parameter	-1.6(9)	
Largest diff. peak and hole	0.150 and -0.137 e.Å^{-3}	

Supplementary Table 4. ORTEP Diagram of 8.

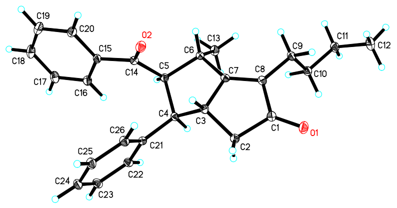

Identification code	**8**	
Empirical formula	C26 H28 O2	
Formula weight	372.48	
Temperature	130 K	
Wavelength	0.71073 Å	
Crystal system	Monoclinic	
Space group	P 1 21/c 1	
Unit cell dimensions	a = 12.7892(10) Å	α= 90°.
	b = 7.8407(6) Å	β= 98.8890(10)°.
	c = 20.1647(16) Å	γ = 90°.
Volume	1997.8(3) Å^3	
Z	4	
Density (calculated)	1.238 Mg/m^3	
Absorption coefficient	0.076 mm^{-1}	
F(000)	800	
Crystal size	0.25 x 0.2 x 0.15 mm^3	
Theta range for data collection	2.044 to 30.504°.	
Index ranges	-18<=h<=18, -10<=k<=11, -28<=l<=26	
Reflections collected	19526	
Independent reflections	6076 [R(int) = 0.0275]	
Completeness to theta = 26.000°	99.9 %	
Absorption correction	Semi-empirical from equivalents	
Max. and min. transmission	0.7461 and 0.6898	
Refinement method	Full-matrix least-squares on F^2	
Data / restraints / parameters	6076 / 0 / 255	
Goodness-of-fit on F^2	1.024	
Final R indices [I>2sigma(I)]	R1 = 0.0448, wR2 = 0.1116	
R indices (all data)	R1 = 0.0597, wR2 = 0.1217	
Extinction coefficient	n/a	
Largest diff. peak and hole	0.396 and -0.243 e.Å^{-3}	

Supplementary Table 5. Optimization of the reaction conditions of Pd-catalyzed allylic alkylation reaction of ketone 1c[a].

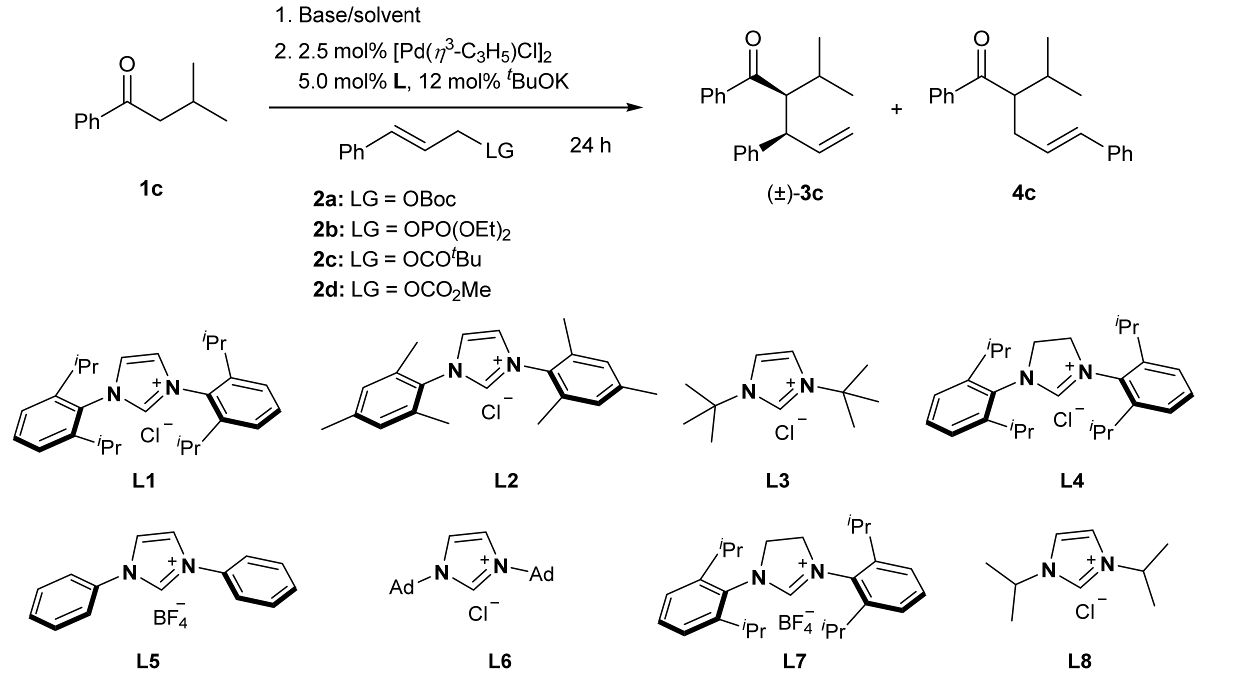

entries	**2**	ligand	base	solvent	T	**3c**/**4c**[b]	*dr*[b]	yield[c]
1[d]	**2a**	**L1**	LiHMDS	toluene	50 °C	95/5	89/11	55
2	**2a**	**L1**	LiHMDS	toluene	50 °C	98/2	85/15	83
3	**2a**	**L1**	LiHMDS	toluene	RT	92/8	92/8	50
4	**2a**	**L4**	LiHMDS	toluene	RT	99/1	92/8	>95
5	**2a**	**L4**	LiHMDS	toluene	50 °C	95/5	88/12	>95
6	**2a**	**L7**	LiHMDS	toluene	50 °C	95/5	88/12	>95
7	**2a**	**L2**	LiHMDS	toluene	50 °C	15/85	--	32
8	**2a**	**L5**	LiHMDS	toluene	50 °C	<5/95	--	28

9	**2a**	**L6**	LiHMDS	toluene	50 °C	82/18	69/31	77
10	**2a**	**L3**	LiHMDS	toluene	50 °C	<5/95	--	33
11	**2a**	**L8**	LiHMDS	toluene	50 °C	<5/95	--	24
12	**2b**	**L4**	LiHMDS	toluene	RT	93/7	89/11	>95
13	**2c**	**L4**	LiHMDS	toluene	RT	97/3	90/10	>95
14	**2d**	**L4**	LiHMDS	toluene	RT	96/4	90/10	>95
15	**2a**	**L4**	NaHMDS	toluene	RT	96/4	91/9	97
16	**2a**	**L4**	KHMDS	toluene	RT	61/39	84/16	74
17	**2a**	**L4**	LiHMDS	THF	RT	98/2	90/10	96
18	**2a**	**L4**	LiHMDS	DME	RT	92/8	89/11	86
19	**2a**	**L4**	LiHMDS	dioxane	RT	94/6	90/10	84
20	**2a**	**L4**	LiHMDS	CH_2Cl_2	RT	NR		

[a]Reaction conditions: **1c**/LiHMDS/**2a**/[Pd(η^3-C_3H_5)Cl]$_2$/ligand = 200/200/100/2.5/5; 0.1 M of ketone **1c**. [b]Determined by ^{1}HNMR, *dr* is the *syn/anti* ratio of **3c**. [c]Isolated yield. [d] **1**/LiHMDS/**2a** = 100/100/100.

Supplementary Table 6. Optimization of the reaction conditions of Pd-catalyzed allylic alkylation reaction of ketone 1f[a].

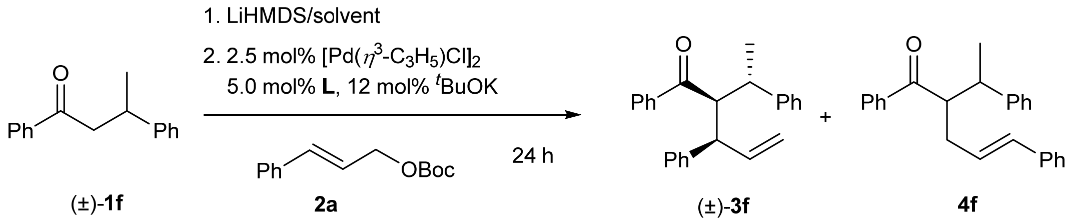

entries	ligand	solvent	T	**3f**/**4f**[b]	*dr*[b]	yield[c]
1	**L1**	toluene	50°C	95/5	85/15	98
2	**L4**	toluene	50°C	95/5	86/14	97
3	**L6**	toluene	50°C	--	--	complex
4	**L4**	THF	50°C	95/5	86/14	95
5	**L4**	toluene	RT	>95/5	86/14	98
6	**L1**	toluene	RT	>95/5	86/14	70

[a]Reaction conditions: **1f**/LiHMDS/**2a**/[Pd(η^3-C_3H_5)Cl]$_2$/ligand = 200/200/100/2.5/5; 0.1 M of ketone **1f**. [b]Determined by ^{1}HNMR, *dr* is the ratio of **3f**/other diastereo isomers. [c]Isolated yield.

Supplementary Table 7. Pd-catalyzed asymmetric allylic alkylation reaction of ketone 1b with chrial NHCs [a].

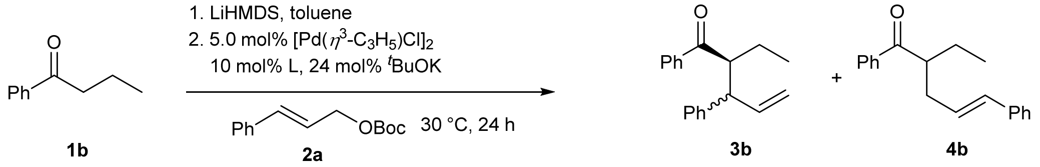

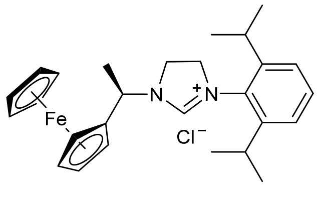

L9

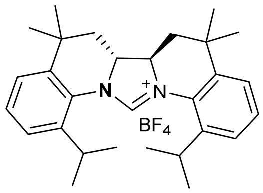

L10

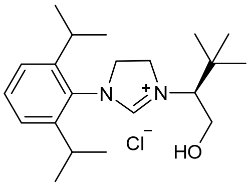

L11

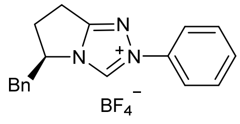

L12

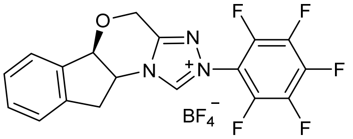

L13

entries	ligand	yield[b]	**3b**/**4b**[c]	*dr*[c]	ee(**3b**, *anti*) [d]	ee(**3b**, *syn*) [d]	ee (**4b**) [d]

1	**L9**	55	60/40	67/33	6	16	8
2	**L10**	52	30/70	77/23	nd	nd	10
3	**L11**	42	11/89	59/41	nd	nd	0
4	**L12**	78	6/94	nd	nd	nd	12
6	**L13**	52	10/90	64/36	nd	nd	4

[a]Reaction conditions: **1b**/LiHMDS/**2a**/[Pd(η^3-C_3H_5)Cl]$_2$/ligand = 200/200/100/5/10; 0.1 M of ketone **1b**. [b]Determined by ^{1}HNMR with mesitylene (23 μL) as an internal standard. [c] Determined by GC. [d]Determined by HPLC. nd was not determined.

Supplementary Table 8. Pd-catalyzed asymmetric allylic alkylation reaction of ketone 1c with chrial NHCs [a].

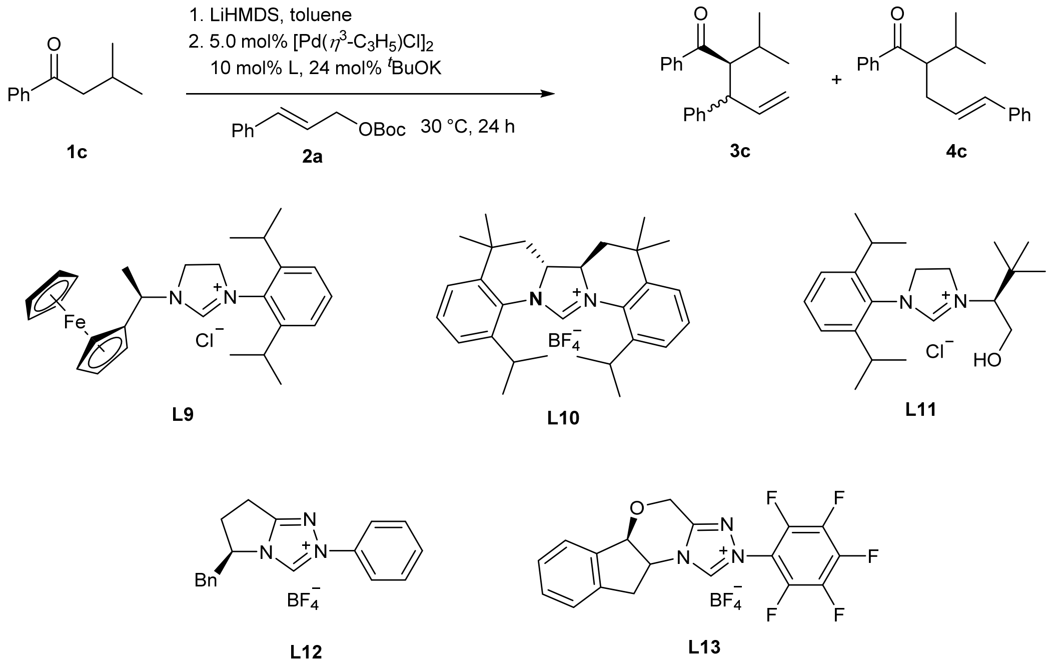

entries	ligand	yield[b]	**3c**/**4c**[c]	*dr*[c]	ee(**3c**, *anti*) [d]	ee(**3c**, *syn*) [d]	ee (**4c**) [d]
1	**L9**	23	29/71	14/86	nd	nd	6
2	**L10**	47	25/75	24/76	nd	nd	8

3	**L11**	38	2/98	nd	nd	nd	2
4	**L12**	30	4/96	nd	nd	nd	1
6	**L13**	59	3/97	nd	nd	nd	10

[a]Reaction conditions: **1c**/LiHMDS/**2a**/[Pd(η^3-C_3H_5)Cl]$_2$/ligand = 200/200/100/5/10; 0.1 M of ketone **1c**. [b]Determined by ^{1}HNMR with mesitylene (23 μL) as an internal standard. [c] Determined by GC. [d]Determined by HPLC. nd was not determined.

Supplementary Methods

Characterization of the products

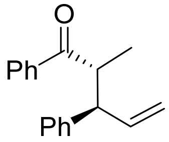

3a[1]

Yield: 95%; B/L: 96/4; *syn/anti*: 20/80; ^{1}H NMR (400 MHz, $CDCl_3$): (taken as a mixture of diastereomers): δ (major diastereomer) 7.72-7.70 (m, 2H), 7.52-7.07 (m, 8H), 6.08-5.97 (m, 1H), 5.08-5.03 (m, 2H), 3.97-3.91 (m, 1H), 3.72-3.68 (m, 1H), 1.20(d, *J* = 6.8Hz, 3H); δ (minor diastereomer) 4.90 (m, 2H), 0.90 (d, *J* = 6.8 Hz, 3H); ^{13}C NMR (101 MHz, $CDCl_3$): δ (major diastereomer) 203.4, 142.8, 139.0, 137.0, 132.7, 128.5, 128.4, 128.0, 127.6, 126.3, 116.6, 53.0, 45.2, 16.5.

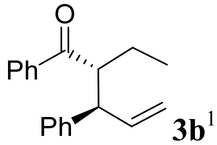

3b[1]

Yield: 98%; B/L: 95/5; *syn/anti*: 30/70;^{1}H NMR (400 MHz, $CDCl_3$): (taken as a mixture of diastereomers): δ (major diastereomer) 7.74 (d, *J* = 7.8 Hz, 2H), 7.56-7.01 (m, 8H), 6.10-5.98 (m, 1H), 5.14 (d, *J* = 17.1 Hz, 1H), 5.12 (d, *J* = 9.9 Hz, 1H), 3.90-3.82 (m, 1H), 3.77-3.71 (m, 1H), 1.87-1.75 (m, 2H), 0.84 (t, *J* = 7.5Hz, 3H); δ (minor diastereomer) 5.92-5.86 (m, 1H), 4.97-4.85 (m, 2H), 0.71 (t, *J* = 7.5 Hz, 3H); ^{13}C NMR (101 MHz, $CDCl_3$): δ (taken as a mixture of diastereomers) 204.2, 203.8, 142.6, 142.0, 139.6, 139.2, 138.9, 138.5, 132.9, 132.6, 128.7, 128.6, 128.4, 128.3, 128.2(2C), 127.9, 127.8, 126.4, 126.3, 116.4, 115.8, 52.7,52.5, 52.1, 51.8, 24.6, 24.1, 11.5, 11.4.

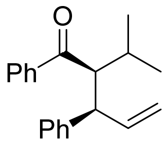

3c

Yield: 95%; B/L: >98/2; *syn/anti*: 92/8; White solid, mp: 97-102°C; ^{1}H NMR (400 MHz, $CDCl_3$): δ (major diastereomer) 7.88 (d, J = 7.2 Hz, 2H), 7.51 (t, J = 7.2 Hz, 1H), 7.41 (t, J = 7.6 Hz, 2H), 7.30-7.22 (m, 4H), 7.20-7.16 (m, 1H), 6.08-5.96 (m, 1H), 4.98 (d, J = 17.2 Hz, 1H), 4.92 (dd, J = 10.4, 1.2 Hz, 1H), 3.94-3.86 (m, 2H),1.90-1.80 (m, 1H), 0.85 (d, J = 6.8 Hz, 3H), 0.83 (d, J = 6.8 Hz, 3H); δ (minor diastereomer) 5.23 (d, J = 17.2 Hz, 1H), 5.09 (dd, J = 10.4, 1.6 Hz, 1H); ^{13}C NMR (101 MHz,$CDCl_3$):(major diastereomer) 203.6, 142.2, 140.0, 139.4, 132.6, 128.6, 128.4, 128.1, 128.0, 126.5, 116.1, 55.4, 50.8, 29.0, 22.0, 18.0; MS (EI) m/z (rel): 278 (M^+, 8), 235 (40), 161(15), 129 (12), 117 (100), 105 (99), 91 (49), 77 (90), 65 (8), 51 (21); IR (film): ν 2957 (w), 2926 (m), 1729 (w), 1660 (m), 1205 (m), 992 (m), 799 (m), 700 (s) cm^{-1}; HRMS (EI) Calcd. for $C_{20}H_{22}O$ (M^+): 278.1671; Found: 278.1668.

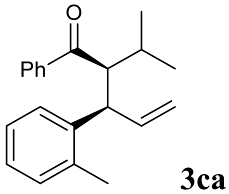

3ca

Yield: 85%; B/L: = 93/7; *syn/anti*: 90/10; ^{1}H NMR (400 MHz, $CDCl_3$): δ (major diastereomer) 7.88 (d, J = 7.2 Hz, 2H), 7.51 (t, J = 7.2 Hz, 1H), 7.41 (t, J = 7.6 Hz, 2H), 7.22-7.08 (m, 4H), 6.08-5.96 (m, 1H), 4.92 (dd, J = 17.2, 10.0 Hz, 2H), 4.20 (t, J = 8.4 Hz, 1H), 3.94-3.86 (m, 1H),2.45 (s, 3H), 1.90-1.80 (m, 1H), 0.85 (d, J = 6.8 Hz, 3H), 0.83 (d, J = 6.8 Hz, 3H); δ (minor diastereomer) 5.18 (d, J = 17.6 Hz, 1H), 5.06 (dd, J = 10.4, 1.6 Hz, 1H); ^{13}C NMR (101 MHz, $CDCl_3$): (major diastereomer) 203.9, 140.1, 139.8, 139.1, 136.0, 132.6, 130.8, 128.4, 128.0, 127.1, 126.2, 126.1, 116.0, 54.1, 46.0, 29.0, 22.0, 19.7, 18.3; MS (EI) m/z (rel): 292 (M^+, 6), 277 (6), 249 (31), 131 (100), 115 (31), 105 (87), 91 (44), 77 (68), 65 (5), 51 (13); IR (film): ν 2961 (w), 2926 (w), 1728 (w), 1668 (m), 1455 (m), 1272 (m), 1207 (m), 992 (m), 760 (m), 697 (s) cm^{-1}; HRMS (EI) Calcd. for $C_{21}H_{24}O$ (M^+): 292.1827; Found: 292.1821.

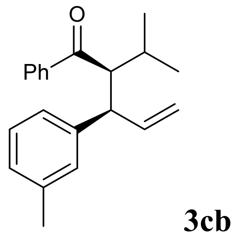

3cb

Yield: 98%; B/L: = 93/7; *syn/anti*: 91/9; ^{1}H NMR (400 MHz, $CDCl_3$): δ (major diastereomer) 7.88 (d, *J* = 7.2 Hz, 2H), 7.51 (t, *J* = 7.2 Hz, 1H), 7.41 (t, *J* = 7.6 Hz, 2H), 7.18 (t, *J* = 8.0 Hz, 1H), 7.08-6.95 (m, 3H), 6.08-5.96 (m, 1H), 4.98 (d, *J* = 16.8Hz, 1H), 4.90 (dd, *J* = 10.0, 1.6 Hz, 1H), 3.94-3.86 (m, 2H), 2.32 (s, 3H), 1.90-1.80 (m, 1H), 0.85 (d, *J* = 6.8 Hz, 3H), 0.83 (d, *J* = 6.8 Hz, 3H); δ (minor diastereomer) 5.20 (d, *J* = 17.6 Hz, 1H), 5.06 (dd, *J* = 10.4, 1.6 Hz, 1H); ^{13}C NMR (101 MHz, $CDCl_3$): (major diastereomer) 203.6, 142.1, 140.0, 139.5, 138.2, 132.6, 128.9, 128.5, 128.4, 128.1, 127.3, 125.1, 116.0, 55.4, 50.8, 29.0, 22.0, 21.5, 18.0; MS (EI) m/z (rel): 292 (M^+, 12), 249 (51), 131 (100), 115 (48), 105 (99), 91 (66), 77 (94), 65 (8), 51 (20); IR (film): *v* 2964 (w), 2925 (w), 1667 (m), 1454 (m), 1272 (m), 1200 (m), 993 (m), 784 (m), 702 (s) cm^{-1}; HRMS (EI) Calcd. for $C_{21}H_{24}O$ (M^+): 292.1827; Found: 292.1825.

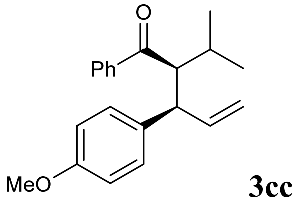

3cc

Yield: 97%; B/L: = 95/5; *syn/anti*: 90/10; ^{1}H NMR (400 MHz,$CDCl_3$): δ (major diastereomer) 7.88 (d, *J* = 7.2 Hz, 2H), 7.51 (t, *J* = 7.2 Hz, 1H), 7.41 (t, *J* = 7.6 Hz, 2H), 7.18 (d, *J* = 8.4 Hz, 2H), 6.84 (d, *J* = 8.8 Hz, 2H), 6.08-5.96 (m, 1H), 4.98 (d, *J* = 17.2Hz, 1H), 4.90 (dd, *J* = 10.0, 1.6 Hz, 1H), 3.94-3.86 (m, 2H), 3.77 (s, 3H), 1.90-1.80 (m, 1H), 0.85 (d, *J* = 6.8 Hz, 3H), 0.83 (d, *J* = 6.8 Hz, 3H); δ (minor diastereomer) 5.20 (d, *J* = 16.8 Hz, 1H), 5.06 (dd, *J* = 10.0, 1.6 Hz, 1H), 3.67 (s, 3H);^{13}C NMR (101 MHz, $CDCl_3$): (major diastereomer) 203.7, 158.2, 140.0, 139.8, 134.2, 132.6, 129.0, 128.5, 128.1, 115.7, 114.0, 55.5, 55.2, 49.9, 29.0, 22.0, 17.9; MS (EI) m/z (rel): 308 (M^+, 1), 265 (41), 147 (100), 105 (27), 91 (24), 77 (29), 65 (2), 51 (5); IR (film): *v*

2960 (w), 1665 (m), 1510 (m), 1261 (m), 996 (m), 921 (m), 825 (m), 699 (s), 655 (m) cm^{-1}; HRMS (EI) Calcd. for $C_{21}H_{24}O_2(M^+)$: 308.1776; Found: 308.1778.

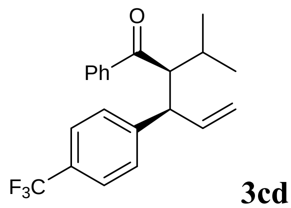

3cd

Yield: 95%; B/L: = 93/7; *syn/anti*: 93/7; ^{1}H NMR (400 MHz, $CDCl_3$): δ (major diastereomer) 7.88 (d, J = 7.2 Hz, 2H), 7.55-7.48 (m, 3H), 7.44-7.36 (m, 4H), 6.08-5.96 (m, 1H), 5.04-4.95 (m, 2H), 4.04-3.96 (m, 1H), 3.93-3.86 (m, 1H), 1.90-1.80 (m, 1H), 0.85 (d, J = 6.8 Hz, 3H), 0.83 (d, J = 6.8 Hz, 3H); δ (minor diastereomer) 5.22 (d, J = 16.8 Hz, 1H), 5.16 (dd, J = 10.0, 1.6 Hz, 1H);^{13}C NMR (101 MHz, $CDCl_3$): (major diastereomer) 202.9, 146.4, 146.3, 139.7, 138.4, 132.8, 128.5, 128.0, 125.6 (q, J = 3.8 Hz), 117.0, 55.2, 50.5, 29.0, 21.8, 18.0; ^{19}F NMR (376 MHz, $CDCl_3$):δ -62.4 (s); MS (EI) m/z (rel): 346 (M^+, 10), 303 (26), 185 (81), 161 (60), 105 (100), 91 (10), 77 (100), 51 (32); IR (film): v 2965 (w), 1670 (m), 1164 (m), 1123 (s), 842(m), 697 (m) cm^{-1}; HRMS (EI) Calcd. for $C_{21}H_{21}OF_3(M^+)$: 346.1545; Found: 346.1541.

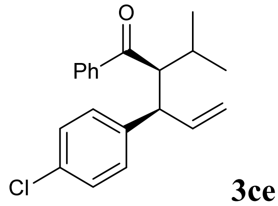

3ce

Yield: 91%; B/L: = 95/5; *syn/anti*: 90/10; ^{1}H NMR (400 MHz, $CDCl_3$): δ (major diastereomer) 7.88 (d, J = 7.2 Hz, 2H), 7.51 (t, J = 7.2 Hz, 1H), 7.41 (t, J = 7.6 Hz, 2H), 7.27-7.24 (m, 2H),7.21-7.18 (m, 2H), 6.08-5.96 (m, 1H), 4.98 (d, J = 16.8Hz, 1H), 4.90 (d, J = 9.6 Hz, 1H), 3.94-3.82 (m, 2H), 1.90-1.80 (m, 1H), 0.85 (d, J = 6.8 Hz, 3H), 0.83 (d, J = 6.8 Hz, 3H); δ (minor diastereomer) 5.20 (d, J = 16.8 Hz, 1H), 5.06 (dd, J = 10.0, 1.6 Hz, 1H); ^{13}C NMR (101 MHz, $CDCl_3$): (major diastereomer) 203.2, 140.7, 138.9, 132.3, 129.5, 128.8, 128.5, 128.1, 116.4, 55.3, 50.1, 29.0, 21.9, 17.9; MS (EI) m/z (rel): 312 (M^+, 6), 314 (2), 269 (49), 151 (99), 115 (95), 105 (100), 89 (12), 77 (99), 69 (6), 51 (33); IR (film): v 2964 (w), 1667 (s), 1488 (m), 1206 (m),

1093 (m), 916 (m), 831(m), 697 (s) cm^{-1}; HRMS (EI) Calcd. for $C_{20}H_{21}OCl(M^+)$: 312.1281; Found: 312.1278.

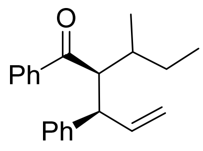

3e

Yield: 98%; B/L: 98/2; *dr*: 62/38; ^{1}H NMR (400 MHz, $CDCl_3$):(mixture of diastereomers) δ (major diastereomer) 7.76 (d, J = 7.6 Hz, 2H), 7.51-7.08 (m, 8H), 6.06-5.96 (m, 1H), 4.98-4.80 (m, 2H), 3.96-3.80 (m, 2H), 1.20-1.15 (m, 1H), 0.90-0.80 (m, 2H), 0.75 (d, J = 6.8 Hz, 3H), 0.60 (t, J = 7.2 Hz, 3H); δ (minor diastereomers) 7.80 (d, J = 7.6 Hz, 2H), 5.92 (m, 1H), 4.98-4.80 (m, 2H), 0.80 (d, J = 6.8 Hz, 3H), 0.65 (t, J = 7.2 Hz, 3H); ^{13}C NMR (101 MHz,$CDCl_3$): (mixture of diastereomers) 203.8, 203.6, 142.4, 142.0, 140.2, 139.8, 139.7, 139.2, 132.6, 132.5, 128.7, 128.6, 128.5,128.4, 128.2(2C), 128.1, 128.0, 126.6, 126.5, 116.1, 116.0, 55.6, 53.6, 50.7, 50.3, 35.8, 35.7, 28.5, 25.2, 17.7, 14.4, 12.0, 11.9; MS (EI) m/z (rel): 292 (M^+, 1), 236 (11), 175 (3), 117 (100), 105 (66), 91 (20), 77 (42), 65 (3), 51 (6); IR (film): v 2963 (m), 2874 (m), 1963 (w), 1674 (m), 1449 (m), 1209 (m), 917 (m), 700 (s) cm^{-1}; HRMS (EI) Calcd. for $C_{21}H_{24}O$ (M^+): 292.1827; Found: 292.1828.

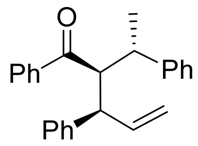

3f

Yield: 98%; B/L: > 95/5; *dr*: 88/12; White solid, mp: 57-60°C; ^{1}H NMR (400 MHz, $CDCl_3$): δ 7.44 (d, J = 7.2 Hz, 2H), 7.35 (t, J = 7.2 Hz, 1H), 7.25-7.10 (m, 12H), 6.18-6.08 (m, 1H), 5.01 (dd, J = 10.0, 1.6 Hz, 1H), 4.88 (d, J =17.6 Hz, 1H), 4.16 (t, J = 7.6 Hz, 1H), 3.70 (t, J = 8.4 Hz, 1H), 3.14-3.06 (m, 1H), 1.21 (d, J = 6.8 Hz, 3H); ^{13}C NMR (101 MHz, $CDCl_3$): 203.4, 144.7, 142.4, 140.0, 137.8, 132.3, 128.7, 128.4, 128.0 (2C), 127.9, 127.6, 126.6, 126.4, 117.0, 57.1, 50.9, 40.6, 17.2; MS (EI) m/z (rel): 340 (M^+, 7), 249 (21), 235 (99), 223 (68), 205 (7), 194 (36), 157 (11), 147 (31), 129 (27), 117 (100), 105 (99), 91 (85), 77 (100), 65 (15), 51 (36); IR (film): v

3059 (w), 2964 (w), 1661 (m), 1449 (m), 1209 (m), 917 (m), 752 (m), 684 (s) cm^{-1}; HRMS (EI) Calcd. for $C_{25}H_{24}O$ (M^+): 340.1827; Found: 340.1823.

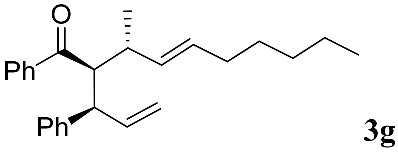

3g

Yield: 98%; B/L: 93/7; *dr*: 77/23; ^1H NMR (400 MHz, $CDCl_3$):(mixture of diastereomers) δ (major diastereomer)7.76 (d, J = 7.2 Hz, 2H), 7.51-7.08 (m, 8H), 6.02-5.95 (m, 1H), 5.35-5.25 (m, 1H), 5.18-5.10 (m, 1H), 5.00-4.90 (m, 2H), 3.95-3.85 (m, 2H), 2.45-2.35 (m, 1H), 1.82-1.73 (m, 2H), 1.30-1.11 (m, 6H), 0.88 (d, J = 6.8 Hz, 3H), 0.77 (t, J = 7.6 Hz, 3H); ^{13}C NMR (101 MHz,$CDCl_3$): (mixture of diastereomers) 203.3, 142.2, 139.7, 138.7, 133.7, 132.5, 130.6, 128.6, 128.3, 128.2, 128.1, 126.5, 116.2, 55.4, 50.7, 37.7, 32.3, 31.3, 28.9, 22.5, 16.2, 14.0; MS (EI) m/z (rel): 360 (M^+, 2), 243 (16), 129 (6), 117 (4), 105 (100), 91 (21), 77 (38), 69 (17), 55 (19); IR (film): ν 2925 (m), 1960 (w), 1963 (w), 1674 (m), 1492 (m), 1205 (m), 916 (m), 700 (s) cm^{-1}; HRMS (EI) Calcd. for $C_{26}H_{32}O$ (M^+): 360.2453; Found: 360.2447.

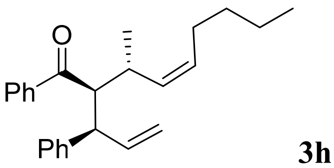

3h

Yield: 56%; B/L: 88/12; *dr*: 86/14; ^1H NMR (400 MHz, $CDCl_3$):(mixture of diastereomers) δ (major diastereomer)7.76 (d, J = 7.2 Hz, 2H), 7.51-7.08 (m, 8H), 6.10-6.00 (m, 1H), 5.30-5.20 (m, 1H), 5.05-4.95 (m, 3H), 3.92-3.84 (m, 2H), 2.80-2.72 (m, 1H), 1.90-1.85 (m, 2H), 1.35-1.20 (m, 4H), 0.90-0.85 (m, 6H);^{13}C NMR (101 MHz,$CDCl_3$): (mixture of diastereomers) 203.3, 142.2, 139.6, 138.5, 133.7, 132.6, 130.0, 128.6, 128.4, 128.2, 128.0, 126.6, 116.4, 55.4, 50.9, 32.9, 31.9, 27.2, 22.4, 16.9, 14.0; MS (EI) m/z (rel): 346 (M^+, 3), 229(12), 171(3), 117(47), 105 (100), 91 (19), 77 (40), 69 (17), 55(18); IR (film): ν 2927 (m), 1960 (w), 1674 (m), 1449 (m), 1204 (m), 917 (m), 697 (s) cm^{-1}; HRMS (EI) Calcd. for $C_{25}H_{30}O$ (M^+): 346.2297; Found: 346.2292.

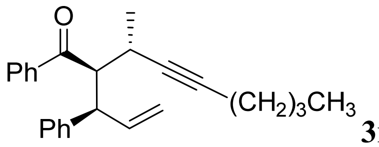

3i

Yield: 98%; B/L: 94/6; *dr*: 93/7; Yellow oil; ^{1}H NMR (400 MHz, $CDCl_3$): (mixture of diastereomers) δ (major diastereomer)7.78 (d, J = 7.2 Hz, 2H), 7.45 (t, J = 7.2 Hz, 1H), 7.38-7.30 (m, 2H), 7.25-7.20 (m, 4H), 7.16-7.10 (m, 1H), 6.17-6.05 (m, 1H), 5.12 (d, J = 16.4 Hz, 1H), 5.05 (dd, J = 10.0, 1.2 Hz, 1H), 4.09 (t, J = 8.0 Hz, 1H), 4.02 (t, J = 7.6 Hz, 1H), 2.80-2.70 (m, 1H), 2.05-2.01 (m, 2H), 1.34-1.25 (m, 4H), 1.01 (d, J = 7.2 Hz, 3H), 0.85 (m, 3H); ^{13}C NMR (101 MHz,$CDCl_3$):δ (major diastereomer) 202.4, 141.7, 139.4, 137.6, 132.6, 128.6, 128.4, 128.3, 128.1, 128.0, 126.6, 83.2, 82.8, 54.9, 50.9, 30.9, 27.2, 21.9, 18.3, 17.6, 13.6; MS (EI) m/z (rel): 344 (M^+, 0.28), 287 (4), 239 (6), 227 (16), 220 (42), 209 (9), 183 (6), 171 (14), 155 (6), 129 (11), 117 (77), 105 (100), 91 (38), 77 (73), 67 (9), 51 (10); IR (film): ν 3028 (w), 2932 (w), 1675 (s), 1449 (m), 1243 (m), 995 (m), 818 (m), 700 (s) cm^{-1}; HRMS (EI) Calcd. for $C_{25}H_{28}O$ (M^+): 344.2140; Found: 344.2146.

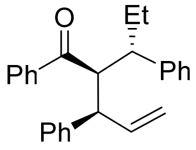

3j

Yield: 98%; B/L: > 95/5; *dr*: 86/14; White solid, mp: 54-56°C; ^{1}H NMR (400 MHz, $CDCl_3$): δ 7.48 (d, J = 8.4 Hz, 2H), 7.38 (t, J = 7.6 Hz, 1H), 7.34-7.26 (m, 2H), 7.24-7.15 (m, 7H), 7.10-7.07 (m, 3H), 6.29-6.21 (m, 1H), 5.08 (dd, J = 10.0,1.6 Hz, 1H), 4.86 (d, J =16.0 Hz, 1H), 4.16 (dd, J = 8.4, 6.4 Hz, 1H), 3.62 (dd, J =8.8, 6.0 Hz, 1H), 2.96-2.90 (m, 1H), 1.81-1.71 (m, 1H), 1.60-1.48 (m, 1H), 0.58 (t, J = 7.2 Hz, 3H); ^{13}C NMR (101 MHz, $CDCl_3$): 203.9, 142.7, 142.3, 139.6, 137.0, 132.4, 128.6, 128.5, 128.4, 128.1, 127.8, 126.5, 126.4, 117.3, 57.2, 50.9, 48.9, 25.3, 12.2; MS (EI) m/z (rel): 354 (M^+, 1.92), 263 (5), 235 (59), 194 (10), 129 (14), 117 (100), 105 (100), 91 (99), 77 (95), 65 (13), 51 (18); IR (film): ν 3058 (w), 2954 (w), 1658 (m), 1206 (m), 919 (m), 759 (m), 687 (s) cm^{-1}; HRMS (EI) Calcd. for $C_{26}H_{26}O$ (M^+): 354.1984; Found: 354.1979.

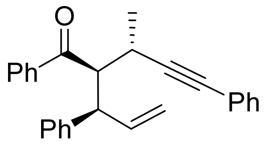

3k

Yield: 98%; B/L: 90/10; *dr*:92/8; Yellow oil; ^{1}H NMR (400 MHz, $CDCl_3$): (mixture of diastereomers) δ 7.85 (d, J = 8.0 Hz, 2H), 7.48 (t, J = 7.2 Hz, 1H), 7.40-7.35 (m, 2H), 7.26-7.20 (m, 7H), 7.18-7.10 (m, 3H), 6.21-6.11 (m, 1H), 5.17 (d, J = 16.8 Hz, 1H), 5.09 (d, J = 10.8 Hz, 1H), 4.20-4.15 (m, 2H), 3.05-2.98 (m, 1H), 1.21 (d, J = 6.8 Hz, 3H); ^{13}C NMR (101 MHz, $CDCl_3$): δ (mixture of diastereomers) 202.0, 141.5, 139.3, 137.5, 132.7, 131.4, 128.7, 128.5, 128.3, 128.1, 128.0, 127.7, 126.8, 126.1, 123.4, 117.1, 92.6, 83.3, 54.4, 50.9, 27.7, 17.2; MS (EI) m/z (rel): 364 (M^+, 1), 259 (13), 247 (49), 220 (11), 203 (6), 181 (4), 165 (4), 141 (9), 128 (35), 115 (51), 105 (99), 91 (61), 77 (100), 65 (6), 51 (20); IR (film): ν 3059 (w), 2977(w), 1673 (m), 1240 (m), 918 (m), 755 (s), 688 (s) cm^{-1}; HRMS (EI) Calcd. for $C_{27}H_{24}O$ (M^+): 364.1827; Found: 364.1823.

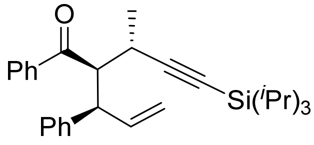

3l

Yield: 99%; B/L: 97/3; *dr*: 96/4;White solid, mp: 49-56°C; ^{1}H NMR (400 MHz, $CDCl_3$): δ 7.68 (d, J = 7.2 Hz, 2H), 7.42 (t, J = 7.2 Hz, 1H), 7.27 (t, J = 8.0 Hz, 2H), 7.20-7.14 (m, 4H), 7.10-7.07 (m, 1H), 6.25-6.15 (m, 1H), 5.20 (dd, J = 16.8, 0.8 Hz,1H), 5.10 (dd, J = 10.0, 1.6 Hz, 1H), 4.19 (dd, J = 9.2, 6.4 Hz, 1H), 3.94 (dd, J = 6.8, 8.4 Hz, 1H), 2.94-2.86 (m, 1H), 1.13 (d, J = 7.2 Hz, 3H), 1.10-1.00 (m, 21H); ^{13}C NMR (101 MHz, $CDCl_3$): δ 202.1, 141.9, 139.1, 136.6, 132.6, 128.6, 128.3, 128.2, 127.9, 126.6, 117.4, 111.3, 83.0, 55.2, 51.2, 28.6, 18.6 (2C), 18.3, 11.2; MS (EI) m/z (rel): 444 (M^+, 2), 401 (20), 359 (11), 118 (10), 117 (100), 115 (18), 105 (11), 91 (8), 77 (8), 59 (1), 45 (1); IR (film): ν 2941 (m), 2864 (m), 2162 (w), 1676 (m), 1206 (m), 882 (m), 675 (s) cm^{-1}; HRMS (EI) Calcd. for $C_{30}H_{40}OSi$ (M^+): 444.2848; Found: 444.2851.

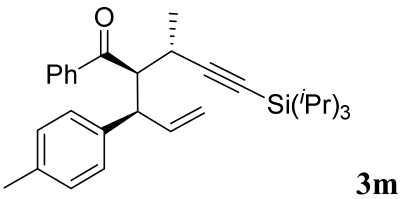

3m

Yield: 98%; B/L: 95/5; *dr*: 95/5; Yellow oil; ^{1}H NMR (400 MHz,$CDCl_3$): δ 7.72 (d, *J* = 7.2 Hz, 2H), 7.43 (t, *J* = 7.2 Hz, 1H), 7.32-7.28 (m, 2H), 7.10-6.90 (m, 4H), 6.20-6.11 (m, 1H), 5.16 (d, *J* = 16.8 Hz, 1H), 5.06 (dd, *J* = 10.4, 1.2 Hz, 1H), 4.12 (t, *J* = 8.0 Hz, 1H), 4.01 (t, *J* = 7.6 Hz, 1H), 2.92-2.85 (m, 1H), 2.25 (s, 3H), 1.12 (d, *J* = 6.8 Hz, 3H), 1.10-1.00 (m, 21H); ^{13}C NMR (101 MHz,$CDCl_3$): 202.2, 139.2, 138.7, 137.2, 136.2, 132.6, 129.3, 128.4, 128.2, 127.8, 117.0, 111.4, 82.8, 54.8, 50.7, 28.5, 20.9, 18.6 (2C), 18.0, 11.2; MS (EI) m/z (rel): 458 (M^+, 0.61), 415 (4), 131 (100), 105 (11), 91(15), 77 (9), 59 (5); IR (film): v 2941 (m), 2864 (m), 2161 (w), 1676 (m), 1243 (m), 993 (m), 918 (s), 674 (s) cm^{-1}; HRMS (EI) Calcd. for $C_{31}H_{42}OSi$ (M^+): 458.3005; Found: 458.3011.

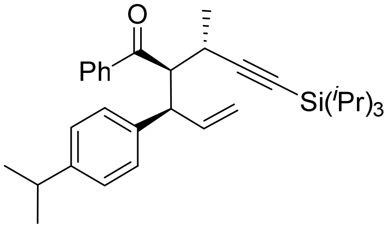

3n

Yield: 98%; B/L: 94/6; *dr*: 95/5; Yellow oil; ^{1}H NMR (400 MHz, $CDCl_3$): δ 7.59 (d, *J* = 7.2 Hz, 2H), 7.38 (t, *J* = 7.2 Hz, 1H), 7.22 (m, 2H), 7.07-6.98 (m, 4H), 6.22-6.12 (m, 1H), 5.22 (dd, *J* = 17.2, 1.2 Hz, 1H), 5.10 (dd, *J* = 10.4, 1.6 Hz, 1H), 4.18 (dd, *J* =9.6, 6.4 Hz, 1H), 3.88 (dd, *J* = 8.8, 6.4 Hz, 1H), 2.95-2.90 (m, 1H), 2.80-2.70(m, 1H), 1.14 (d, *J* = 7.2 Hz, 3H), 1.13 (d, *J* = 6.8 Hz, 3H), 1.05-0.97 (m, 21H); ^{13}C NMR (101 MHz, $CDCl_3$): 202.3, 147.2, 139.2, 139.2, 136.5, 132.5, 128.3, 128.0, 127.7, 126.6, 117.3, 111.5, 82.9, 55.7, 50.8, 33.7, 28.6, 24.0, 23.9 18.7, 18.6, 18.4, 11.2; MS (EI) m/z (rel): 486 (M^+, 1.19), 443 (8), 277 (6), 159 (31), 131 (8), 117 (100), 105 (11), 91 (4), 77 (8), 59 (5); IR (film): v 2941 (m), 2864 (m), 2161 (w), 1675 (m), 1462 (m), 1206 (m), 881 (s), 675 (s) cm^{-1}; HRMS (EI) Calcd. for $C_{33}H_{46}OSi$ (M^+): 486.3318; Found: 486.3310.

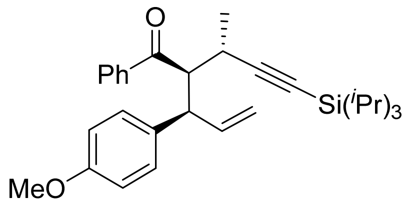

3o

Yield: 99%; B/L: 94/6; *dr*: 96/4; Yellow oil; ^{1}H NMR (400 MHz, $CDCl_3$): δ 7.71 (d, J = 7.2 Hz, 2H), 7.43 (t, J = 7.2 Hz, 1H), 7.32-7.25 (m, 2H), 7.08 (d, J = 8.8 Hz, 2H), 6.73 (d, J = 8.8 Hz, 2H), 6.20-6.10 (m, 1H), 5.16 (d, J = 16.0 Hz, 1H), 5.07 (dd, J = 10.0, 2.0 Hz, 1H), 4.13 (dd, J = 9.2,7.2 Hz, 1H), 3.96 (dd, J = 8.0, 6.8 Hz, 1H), 3.72 (s, 3H), 2.92-2.86 (m, 1H), 1.14 (d, J = 6.8 Hz, 3H), 1.10-1.00 (m, 21H); ^{13}C NMR (101 MHz, $CDCl_3$): 202.3, 158.2, 139.1, 137.1, 134.0, 132.6, 128.8, 128.4, 128.2, 117.0, 114.0, 111.4, 82.9, 55.2, 55.2, 50.3, 28.5, 18.6 (2C), 18.2, 11.2; MS (EI) m/z (rel): 474 (M^+, 0.76), 431 (1), 265 (17), 147 (100), 131 (7), 105 (10), 91 (21), 77 (9), 59 (5); IR (film): *v* 2941 (m), 2864 (m), 2161 (w), 1675 (m), 1511 (s), 1249 (s), 882 (s), 677 (s) cm^{-1}; HRMS (EI) Calcd. for $C_{31}H_{42}O_2Si$ (M^+): 474.2954; Found: 474.2952.

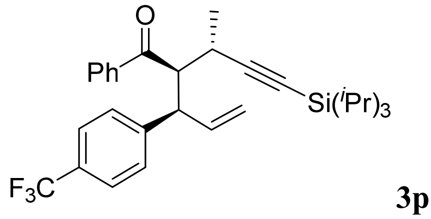

3p

Yield: 95%; B/L: 96/4; *dr*: 97/3; Yellow oil; ^{1}H NMR (400 MHz, $CDCl_3$): δ 7.64 (d, J = 7.2 Hz, 2H), 7.43-7.40 (m, 3H), 7.30-7.20 (m, 4H), 6.25-6.15 (m, 1H), 5.24 (d, J = 16.8 Hz, 1H), 5.17 (dd, J = 10.0, 2.0 Hz, 1H), 4.28 (dd, J = 9.2, 6.2 Hz, 1H), 3.88 (dd, J = 9.2, 6.4 Hz,1H), 2.98-2.90 (m, 1H), 1.12 (d, J = 6.8 Hz, 3H), 1.10-1.00 (m, 21H); ^{13}C NMR (101 MHz, $CDCl_3$): 201.5, 146.2, 138.8, 135.4, 132.9, 128.3, 128.2, 128.1, 125.5 (q, J = 3.8 Hz), 118.4, 110.9, 83.4, 55.2, 51.0, 28.6, 18.6, 18.6, 18.5, 11.2; ^{19}F NMR (376 MHz, $CDCl_3$):δ -62.6 (s); MS (EI) m/z (rel): 512 (M^+, 0.76), 469 (22), 427 (15), 284 (100), 185 (16), 165 (20), 115(15), 105(67), 77 (38), 59 (13); IR (film): *v* 2942 (w), 2865 (w), 2162 (w), 1676 (m), 1462 (w), 1325 (s), 1124(s), 882 (m), 674 (s) cm^{-1}; HRMS (EI) Calcd. for $C_{31}H_{39}OF_3Si$ (M^+): 512.2722; Found: 512.2717.

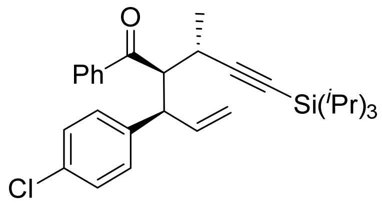

3q

Yield: 98%; B/L: 92/8; *dr*: 95/5; Yellow oil; ^{1}H NMR (400 MHz, $CDCl_3$): δ 7.68 (d, *J* = 7.6 Hz, 2H), 7.45 (t, *J* = 7.6 Hz, 1H), 7.30 (t, *J* = 7.6 Hz, 2H),7.16-7.07 (m, 4H), 6.20-6.10 (m, 1H), 5.20 (d, *J* = 17.2 Hz, 1H), 5.13 (dd, *J* = 10.4, 1.6 Hz, 1H), 4.17 (dd, *J* = 9.2, 6.4 Hz, 1H), 3.90 (dd, *J* = 8.4, 6.4 Hz, 1H), 2.95-2.85 (m, 1H), 1.13 (d, *J* = 6.8 Hz, 3H), 1.10-1.00 (m, 21H); ^{13}C NMR (101 MHz, $CDCl_3$): 201.8, 140.4, 138.9, 136.1, 132.8, 132.3, 129.2, 128.7, 128.3, 117.8, 111.0, 83.2, 55.1, 50.0, 28.6, 18.6 (2C), 18.4, 11.2; MS (EI) m/z (rel): 478 (M^+, 0.81), 435 (16), 393 (13), 242 (9), 151 (100), 131 (13), 115 (45), 105 (19), 95 (5), 77 (22), 59 (12); IR (film): *v* 2941 (m), 2864 (m), 2162 (w), 1675 (m), 1207 (m), 881 (s), 676 (s) cm^{-1}; HRMS (EI) Calcd. for $C_{30}H_{39}OSiCl$ (M^+): 478.2459; Found: 478.2453.

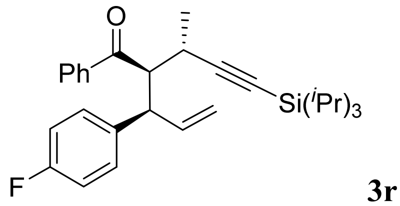

3r

Yield: 94%; B/L: 94/6; *dr*: 95/5; Yellow oil; ^{1}H NMR (400 MHz, $CDCl_3$): δ 7.67 (d, *J* = 7.6 Hz, 2H), 7.43 (t, *J* = 7.6 Hz, 1H), 7.29 (t, *J* = 7.6 Hz, 2H), 7.12-7.08 (m, 2H), 6.89-6.81 (m, 2H), 6.22-6.12 (m, 1H), 5.20 (d, *J* = 17.2 Hz, 1H), 5.12 (dd, *J* = 10.0, 1.2 Hz, 1H), 4.20 (dd, *J* = 9.2, 6.4 Hz, 1H), 3.88 (dd, *J* = 8.8, 6.4 Hz, 1H), 2.95-2.85 (m, 1H), 1.12 (d, *J* = 6.8 Hz, 3H), 1.10-1.00 (m, 21H); ^{13}C NMR (101 MHz, $CDCl_3$): 202.0, 161.5 (d, *J* = 246.1 Hz), 138.9, 137.7 (d, *J* = 3.0 Hz), 136.2, 132.8, 129.3 (d, *J* = 7.7 Hz), 128.3, 117.6, 115.3 (d, *J* = 21.4 Hz), 111.1, 83.1, 55.4, 50.4, 28.7, 18.6 (2C), 18.5, 11.2; ^{19}F NMR (376 MHz, $CDCl_3$):δ -116.2 (m); MS (EI) m/z (rel): 462 (M^+, 0.54), 419 (11), 377 (9), 135 (100), 109 (14), 77 (12), 59 (6); IR (film): *v* 2941 (w), 2864 (w), 2162 (w), 1675 (m), 1508 (m), 1205 (m), 882 (s), 675 (s) cm^{-1}; HRMS (EI) Calcd. for $C_{30}H_{39}OFSi$ (M^+): 462.2754; Found: 462.2761.

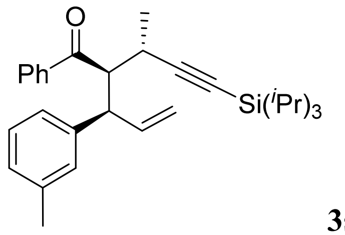

3s

Yield: 98%; B/L: 97/3; *dr*: 97/3; Yellow oil; ^{1}H NMR (400 MHz, $CDCl_3$): δ 7.72 (d, *J*= 7.2 Hz, 2H), 7.43 (t, *J* = 7.2 Hz, 1H), 7.30-7.27 (m, 2H), 7.07-7.05 (m, 1H), 7.00-6.85 (m, 3H), 6.21-6.11 (m, 1H), 5.20 (d, *J* = 17.2 Hz, 1H), 5.10 (dd, *J* = 10.0, 1.6 Hz, 1H), 4.16 (dd, *J* = 8.8, 6.4 Hz, 1H), 3.96 (dd, *J* =8.4, 6.4 Hz, 1H), 2.95-2.85 (m, 1H), 2.21 (s, 3H), 1.14 (d, *J* = 7.2 Hz, 3H), 1.10-1.00 (m, 21H); ^{13}C NMR (101 MHz, $CDCl_3$): 202.2, 141.8, 139.2, 138.1, 136.8, 132.6, 128.7, 128.5, 128.3, 128.1, 127.3, 124.9, 117.3, 111.4, 82.9, 55.2, 51.1, 28.6, 21.3, 18.7, 18.6, 18.3, 11.2; MS (EI) m/z (rel): 458 (M^+, 0.77), 415(8), 373 (8), 131 (100), 105 (13), 91 (17), 77 (11), 59 (6); IR (film): ν 2941 (m), 2864 (m), 2162 (w), 1675 (m), 1598 (w), 1204 (m), 882 (m), 672 (s) cm^{-1}; HRMS (EI) Calcd. for $C_{31}H_{42}OSi$ (M^+): 458.3005; Found: 458.3002.

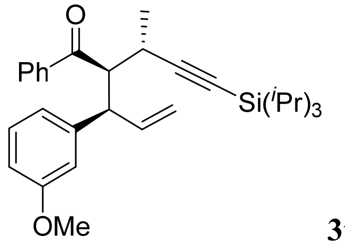

3t

Yield: 95%; B/L: 95/5; *dr*: 95/5; Yellow oil; ^{1}H NMR (400 MHz, $CDCl_3$):δ 7.68 (d, *J* = 7.6 Hz, 2H), 7.43 (t, *J* = 7.6 Hz, 2H), 7.28 (t, *J* = 7.6 Hz, 2H), 7.09 (t, *J* = 7.6Hz, 1H), 6.75 (d, *J* = 7.6 Hz, 2H), 6.68-6.62 (m, 2H), 6.20-6.10 (m, 1H), 5.20 (d, *J* = 16.0Hz, 1H), 5.11 (d, *J* = 10.0Hz, 1H), 4.12-4.05 (m, 1H), 3.94-3.82 (m, 1H), 3.67 (s, 3H), 2.93-2.80 (m, 1H), 1.13 (d, *J* = 6.8Hz, 3H), 1.10-1.00 (m, 21H); ^{13}C NMR (101 MHz, $CDCl_3$): 202.1, 159.7, 143.5, 139.1, 136.5, 132.7, 129.6, 128.3, 128.2, 120.2, 117.4, 113.6, 112.1, 111.3, 82.9, 55.1, 55.0, 51.3, 28.6, 18.6 (2C), 18.3, 11.2; MS (EI) m/z (rel): 474 (M^+, 0.63), 431 (24), 389 (7), 327 (19), 285 (8), 147 (100), 105 (16), 91(31), 77 (15), 59 (8); IR (film): ν 2941 (m), 2891 (m), 2161 (w), 1674 (m), 1598 (m),

1462 (m), 1257 (m), 919 (m), 882 (s), 674 (s) cm^{-1}; HRMS (EI) Calcd. for $C_{31}H_{42}O_2Si$ (M^+): 474.2954; Found: 474.2949.

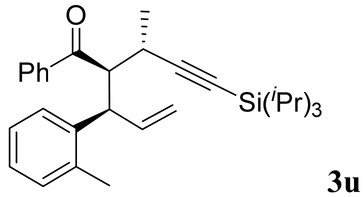

3u

Yield: 96%; B/L: 90/10; *dr*: 93/7; Yellow oil; ^{1}H NMR (400 MHz, $CDCl_3$): δ 7.60 (d, J = 7.2 Hz, 2H), 7.40 (t, J = 7.2 Hz, 1H), 7.30-7.28 (m, 2H), 7.16-7.13 (m, 1H), 7.00-6.95 (m, 2H), 6.90-6.85 (m, 1H), 6.30-6.20 (m, 1H), 5.22 (dd, J = 16.8, 0.8 Hz, 1H), 5.13 (dd, J = 10.0, 2.0 Hz, 1H), 4.43 (dd, J = 9.2, 6.4 Hz, 1H), 3.96 (dd, J = 8.8, 6.0 Hz, 1H), 3.01-2.95 (m, 1H), 2.52 (s, 3H), 1.14 (d, J = 6.8 Hz, 3H), 1.10-1.00 (m, 21H); ^{13}C NMR (101 MHz, $CDCl_3$): 202.4, 139.7, 139.3, 139.5, 135.3, 132.6, 130.7, 128.1, 127.2, 126.4, 126.0, 117.5, 111.2, 82.8, 52.8, 46.7, 28.7, 19.7, 18.7, 18.6, 11.2; MS (EI) m/z (rel): 458 (M^+, 0.75), 415 (6), 373 (7), 131 (100), 105 (12), 91(17), 77 (10), 59 (6); IR (film): *v* 2941 (m), 2864 (m), 2161 (w), 1675 (m), 1462(m), 1205 (m), 994(m), 882 (m), 675 (s) cm^{-1}; HRMS (EI) Calcd. for $C_{31}H_{42}OSi$ (M^+): 458.3005; Found: 458.3010.

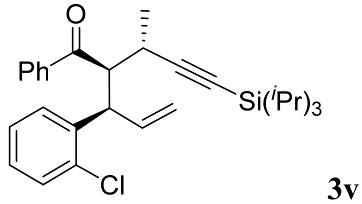

3v

Yield: 99%; B/L: 95/5; *dr*: 94/6; Yellow oil; ^{1}H NMR (400 MHz, $CDCl_3$): δ 7.58 (d, J = 7.2 Hz, 2H), 7.33 (t, J = 7.2 Hz, 1H), 7.26-7.17 (m, 3H), 6.96-6.81 (m, 3H), 6.38 (m, 1H), 5.31 (dd, J = 16.8, 1.6 Hz, 1H), 5.22 (dd, J = 10.0, 1.6 Hz, 1H), 4.71 (dd, J = 9.6, 7.6 Hz, 1H),4.09 (dd, J = 9.6, 6.0 Hz, 1H), 3.10-3.01 (m, 1H), 1.12 (d, J = 6.8 Hz, 3H), 1.11-1.00 (m, 21H); ^{13}C NMR (101 MHz,$CDCl_3$): 202.2, 139.2, 138.9, 134.7, 133.3, 132.7, 129.6, 129.0, 128.1, 127.9, 127.6, 126.7, 118.9, 110.5, 82.9, 51.8, 47.2, 22.8, 19.3, 18.7, 11.3; MS (EI) m/z (rel): 478 (M^+, 1.32), 480 (0.63), 435 (51), 393 (21), 242 (31), 151 (100), 105 (47), 95(12), 77 (45), 59 (21); IR (film): *v*

2942 (m), 2864 (m), 2162 (w), 1675 (m), 1463 (m), 1207 (m), 996 (m), 881 (m), 675 (s) cm^{-1}; HRMS (EI) Calcd. for $C_{30}H_{39}OSiCl$ (M^+): 478.245; Found: 478.2465.

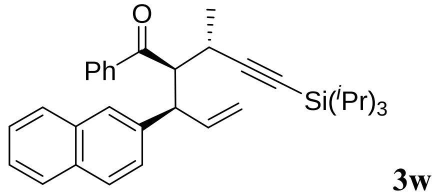

3w

Yield: 90%; B/L: 95/5; *dr*: 98/2; The yield wasdetermined by ^{1}H NMR spectroscopy using mesitylene (23 μL) as internal standard. Yellow oil; The product **3x** was purified by a preparative kaseisorb LC ODS 2000 (10 × 250 mm, 5 um) column (100% acetonitrile, 5.0 mL/min, 240 nm, t_R = 14.1 min) for NMR, IR, and MS analysis; ^{1}H NMR (400 MHz, $CDCl_3$): δ 7.74 (m, 5H), 7.60 (s, 1H), 7.45-7.30 (m, 4H), 7.24-7.20 (m, 2H), 6.28-6.18 (m, 1H), 5.22 (d, J = 16.8 Hz, 1H), 5.13 (dd, J = 10.4, 1.2 Hz, 1H), 4.34 (t, J = 7.6 Hz, 1H), 4.10 (t, J = 7.6 Hz, 1H), 2.93-2.85 (m, 1H), 1.16 (d, J = 6.8 Hz, 3H), 1.10-1.00 (m, 21H); ^{13}C NMR (101 MHz, $CDCl_3$) δ 202.1, 139.2, 139.0, 137.0, 133.4, 133.0, 132.3, 128.3, 128.1, 127.6, 127.5, 126.7, 126.1, 126.0, 125.5, 117.5, 111.3, 83.0, 54.7, 51.2, 28.5, 18.6 (2C), 18.1, 11.2; MS (EI) m/z (rel): 494 (M^+, 2), 451 (7), 285 (8), 168 (20), 167 (100)， 152(14), 131(8), 105 (9), 77(9); IR (film): v 2940 (m), 2863 (m), 2160 (m), 1674 (m), 1250 (m), 881 (m), 674 (s) cm^{-1}; HRMS (EI) Calcd. for $C_{31}H_{42}OSi$ (M^+): 494.3005; Found: 494.2998.

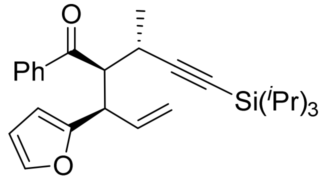

3x

Yield: 98%; B/L: = 95/5; *dr*: 95/5; Yellow oil; ^{1}H NMR (400 MHz, $CDCl_3$): δ 7.75 (d, J = 8.0 Hz, 2H), 7.45 (t, J = 7.2 Hz, 1H), 7.38-7.30 (m, 2H), 7.15-7.12 (m, 1H), 6.09-6.07 (m, 1H), 6.05-5.95 (m, 1H), 5.90 (d, J = 2.0 Hz, 1H), 5.30 (d, J = 16.0Hz, 1H), 5.20 (dd, J = 10.4, 2.0 Hz, 1H), 4.34 (dd, J = 9.6, 5.6 Hz, 1H), 4.05 (dd, J = 9.2 , 5.6Hz, 1H), 2.98-2.90 (m, 1H), 1.12 (d, J = 6.8 Hz, 3H), 1.10-1.00 (m, 21H); ^{13}C NMR (101 MHz, $CDCl_3$): 201.6, 154.6, 141.0, 138.5, 133.6, 132.7, 128.2, 128.1, 118.5, 110.9, 110.0, 106.1, 83.3, 54.5, 44.8, 28.5, 18.8, 18.6 (2C), 11.2; MS

(EI) m/z (rel): 434 (M^+, 0.27), 391 (10), 373 (3), 261 (3), 225 (14), 131 (8), 107 (100), 91 (3), 79 (30), 77 (26), 59 (6); IR (film): *v* 2941 (m), 2865 (m), 2162 (w), 1677 (m), 1462 (m), 1207 (m), 994 (m), 881 (s), 675 (s) cm^{-1}; HRMS (EI) Calcd. for $C_{28}H_{38}O_2Si$ (M^+): 434.2641; Found: 434.2645.

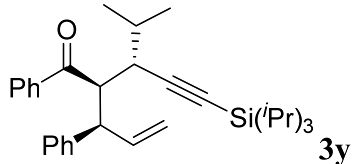

3y

Yield: 99%; B/L:93/7; *dr*: 95/5; White solid, mp: 68-73°C; ^{1}H NMR (400 MHz, $CDCl_3$): δ 7.41 (d, *J* = 7.6 Hz, 2H), 7.35 (t, *J* = 7.2 Hz, 1H), 7.19 (t, *J* = 8.0 Hz, 2H), 7.10-7.05 (m, 4H), 7.04-7.01 (m, 1H), 6.45-6.35 (m, 1H), 5.36 (dd, *J* = 17.2, 2.0 Hz, 1H), 5.28 (dd, *J* = 10.0, 2.0 Hz, 1H), 4.35(dd, *J* = 10.4, 4.0 Hz, 1H), 3.90 (dd, *J* = 11.2, 4.0 Hz, 1H), 3.05-3.01 (m, 1H), 1.55-1.50 (m, 1H), 1.20-1.10 (m, 21H), 1.01 (d, *J* = 6.4 Hz, 3H), 0.81 (d, *J* = 6.4 Hz, 3H); ^{13}C NMR (101 MHz, $CDCl_3$): 202.4, 142.8, 138.6, 135.2, 132.6, 128.4, 128.1, 128.0, 127.8, 126.5, 118.1, 107.4, 85.7, 53.9, 51.5, 42.4, 28.3, 22.4, 18.8 (2C), 16.9, 11.4; MS (EI) m/z (rel): 472 (M^+, 1), 429 (22), 387 (4), 355 (5), 269 (8), 220 (7), 131(10), 117(100), 105 (19), 91(12), 77 (13), 59 (11); IR (film): *v* 2941 (m), 2864 (m), 2165 (w), 1668 (m), 1451 (m), 926 (m), 725 (m), 696 (s), 675 (s) cm^{-1}; HRMS (EI) Calcd. for $C_{32}H_{44}OSi$ (M^+): 472.3161; Found: 472.3165.

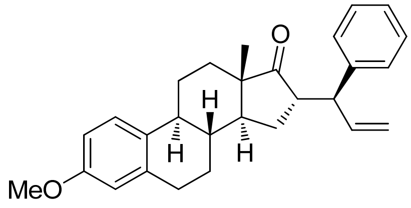

7

Yield: 96%; B/L: 84/16; dr: 91/9; $[\alpha]_D^{28}$= 9.15 (*c*1.0, $CHCl_3$); White solid, mp: 126-129°C; ^{1}H NMR (400 MHz, $CDCl_3$) δ 7.35-7.15 (m, 6H), 6.71 (dd, *J* = 8.8, 2.8 Hz, 1H), 6.63 (d, *J* = 2.8 Hz, 1H), 6.06-5.96 (m, 1H), 5.18-5.10 (m, 2H), 4.10-4.08 (m, 1H), 3.76 (s, 3H), 3.04 (dt, *J* = 10.8, 2.7 Hz, 1H), 2.91-2.88 (m, 2H), 2.42-2.38 (m, 1H), 2.22-2.16 (m, 2H), 2.01-1.85 (m, 3H), 1.60-1.30 (m,5H), 0.95 (s, 3H); ^{13}C NMR (101 MHz, $CDCl_3$): 219.8, 157.6, 142.8, 137.7, 137.2,

132.1, 128.5, 127.9, 126.5, 126.3, 117.9, 113.9, 111.5, 55.2, 49.7, 48.7, 48.5, 48.3, 43.9, 38.4, 31.3, 29.6, 26.5, 25.8, 24.6, 15.2; IR (film): ν 2919 (m), 2858 (m), 1735 (m), 1496 (m), 1278 (s), 1011(s), 799 (s), 698 (m) cm^{-1}; HRMS (EI) Calcd. for $C_{27}H_{24}O$ (M^+): 400.2402; Found: 400.2406.

Synthetic application

1. gram-scale experiment

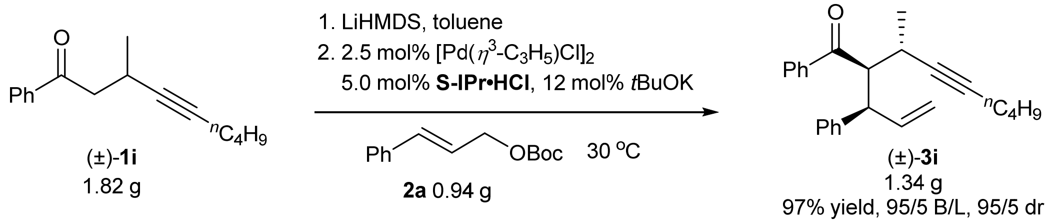

A dry schlenk tube (100 mL) was flame dried and flushed with Argon. Ketone **1i** (1.824g, 8.0 mmol) and toluene (40.0 mL) were added into the dry Schlenk tube. LHMDS(1.0 M in THF, 8.0 mL, 8.0 mmol) were added at 0°C and stirred at room temperature for 30 min. In a separated flask, $[Pd(\eta^3\text{-}C_3H_5)Cl]_2$(38 mg, 0.10 mmol), S-IPr·HCl (88 mg, 0.12 mmol) and toluene (20 mL) were added, followed by addition of *t*-BuOK (1.0 M in THF, 0.5 mL, 0.5 mmol) at 0°C, The resulting mixture was stirred at room temperature for 30 min, then added to the ketone solution. The allylic substrates **2a**(936 mg, 4.0 mmol) and toluene (20 mL) was then added and the mixture was stirred at 30 °C. After 48h the reaction was complete (monitored by GC/MS), the reaction mixture was quenched by H_2O (2 mL). The resulting mixture was extracted with diethyl ether (3 × 10 mL). The combined organic layer was washed with saturated brine (20 mL), dried (Na_2SO_4) and concentrated in vacuo to afford a crude product. The diastereoselectivity of the crude product was then determined by ^1H NMR spectroscopy. After this analysis, the crude reaction mixture was purified by flash column silica gel chromatography (eluting with petroleum ether/toluene 1/1) to yield the product **3i**(1.34 g, yield 97%) and **1i** was recovered (820 mg, yield 90%).**3i**: B/L: 95/5; *dr*: 95/5.

2. Chirality transfer from enantiomerically enriched β-substituted ketones.

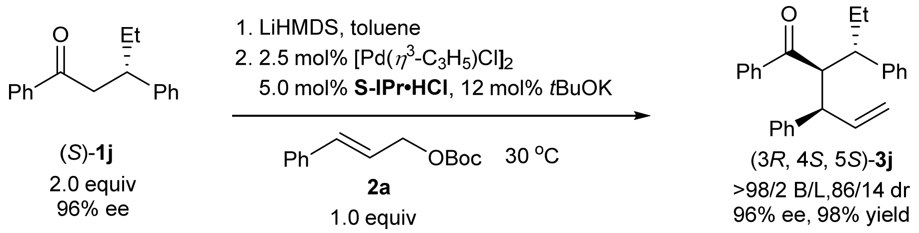

The (*S*)-**1j** was prepared according to the literature procedure[2].

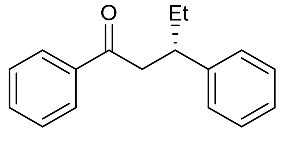

(S)-1j

ee: 96%; $[\alpha]_D^{30}$= 4.0 (*c*1.0, $CHCl_3$); ^{1}H NMR (400 MHz, $CDCl_3$): δ7.92-7.83 (m, 2H), 7.53-7.46 (m, 1H), 7.44-7.35 (m, 2H), 7.31-7.11 (m, 5H), 3.33-3.16 (m, 3H), 1.83-1.55(m, 2H), 0.80 (t, *J* = 7.6 Hz, 3H); HPLC (Chiralcel AD-H, Hexane/*i*-Propanol = 95/5, 1.0 mL/min, 214 nm), t_R (major) = 5.6 min for (*S*), t_R(minor) = 6.5 min for (*R*).

A dry schlenk tube was flame dried and flushed with Argon. Ketone (*S*)-**1j** (95.2 mg, 0.4 mmol) and toluene (2.0 mL) were added into the dry Schlenk tube. LHMDS(1.0 M in THF, 0.4 mL, 0.4mmol) were added at 0°Cand stirred at room temperature for 30 min. In a separated flask, $[Pd(\eta^3-C_3H_5)Cl]_2$ (1.9 mg, 0.005 mmol), S-IPr·HCl (4.4 mg, 0.006 mmol) and toluene (1.0 mL) were added, followed by addition of *t*-BuOK (1.0 M in THF, 25 uL, 0.025 mmol) at 0°C, the resulting mixture was stirred at room temperature for 30 min, then added to the ketone solution. The allylic substrate **2a**(47 mg, 0.2 mmol) and toluene (1.0 mL) was added and the mixture was stirred at 30 °C. After the reaction was complete, the reaction mixture was quenched by H_2O (0. 5 mL).The solution was dried (anhydrous Na_2SO_4) and then filtered through a 0.5 inch plug of silica gel (eluting with EtOAc) to remove the solid. The crude reaction mixture was concentrated under reduced pressure. $CDCl_3$ (0.7-0.8 mL) was added to dissolve the crude reaction mixture, and mesitylene (23 μL) was added as an internal standard. The diastereoselectivity was then determined by ^{1}H NMR spectroscopy. After this analysis, the crude reaction mixture was purified by flash column silica gel chromatography (eluting with petroleum ether/toluene 1/1) to yield the product **3j** (69 mg, yield 98%, B/L > 98/2, *dr* = 86/14). ee: 96%; $[\alpha]_D^{28}$= -33.9 (*c* 0.50,

CHCl$_3$); HPLC (Agilent SDC-AD-H, CO_2/*i*-Propanol = 90/10, 1.3 mL/min, 214 nm), t_R (major) = 8.08 min, t_R(minor) = 9.04 min.

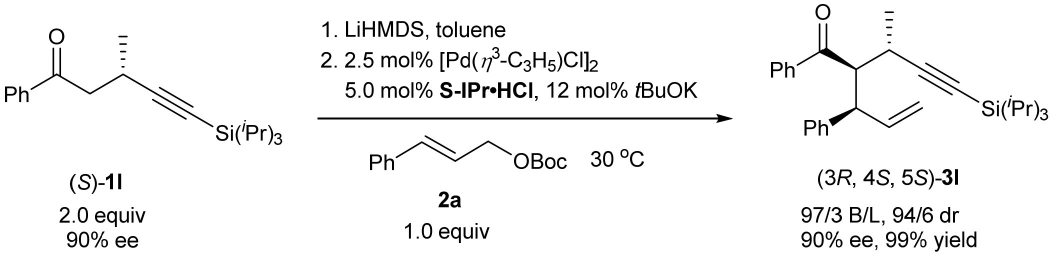

The (*S*)-**1l** was prepared according to the literature procedure[3].

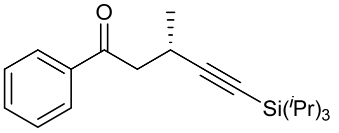

(*S*)-1l

ee: 90% (*S*); $[\alpha]_D^{30}$= -2.2 (*c* 1.0, CHCl$_3$);^{1}H NMR (400 MHz, CDCl$_3$): δ7.96 (d, *J* = 7.2 Hz, 2H), 7.56 (t, *J* = 7.2 Hz,1H), 7.46 (t, *J* = 8.0 Hz, 2H), 3.29-3.19 (m, 2H), 3.05-2.98 (m, 1H), 1.28 (d, *J* = 6.8 Hz, 3H), 1.05-0.80 (m, 21H); HPLC (Agilent SFC-AD-H, CO_2/*i*-Propanol = 99/1, 1.5 mL/min, 230 nm), t_R (major) = 6.87 min, t_R(minor) = 7.54 min for (*R*).{The absolute configuration of **1l** was determined to be *S* by comparing the reported HPLC traces of (*S*)-**1l**[2]. Chiralcel OJ-H Column hexanes, 0.1mL/min, 214 nm, t_R (major)= 42.61 min for (*S*), t_R (minor) = 45.76 min. ($[\alpha]_D^{30}$= -2.2, c 1.0 in CHCl$_3$) }.

A dry schlenk tube was flame dried and flushed with Argon. ketone (***S***)-**1l** (132 mg, 0.4 mmol) was and toluene (2.0 mL) were added into the dry Schlenk tube. LHMDS(1.0 M in THF, 0.4 mL, 0.4mmol) were added at 0°Cand stirred at room temperature for 30 min. In a separated flask, [Pd(η^3-C$_3$H$_5$)Cl]$_2$ (1.9 mg, 0.005 mmol), S-IPr·HCl (4.4 mg, 0.006 mmol) and toluene (1.0 mL) were added, followed by addition of *t*-BuOK (1.0 M in THF, 25 uL, 0.025 mmol) at 0 °C.The resulting mixture was stirred at room temperature for 30 min, then added to the ketone solution. The allylic substrate **2a**(47 mg, 0.2 mmol) and toluene (1.0 mL) was then added and the mixture was stirred at 30 °C.After the reaction was complete, the reaction mixture was quenched by H_2O

(0. 5 mL), the solution was dried (anhydrous Na_2SO_4) and then filtered through a 0.5 inch plug of silica gel (eluting with EtOAc) to remove the solid. The crude reaction mixture was concentrated under reduced pressure. $CDCl_3$ (0.7-0.8 mL) was added to dissolve the crude reaction mixture, and mesitylene (23 μL) was added as an internal standard. The diastereoselectivity was then determined by ^1H NMR spectroscopy. After this analysis, the crude reaction mixture was purified by flash column silica gel chromatography (eluting with petroleum ether/toluene 1/1) to yield the product **3l** (86.9 mg, B/L = 97/3, *dr* = 96/4). ee 90%;$[\alpha]_D^{28}$= 3.7 (*c*1.0, $CHCl_3$);HPLC (Agilent SDC-OJ, CO_2/*i*-Propanol = 95/5, 1.3 mL/min, 214 nm), t_R (major) = 4.58 min, t_R(minor) = 5.80 min.

3. Pauson-Khand reaction of product 3i

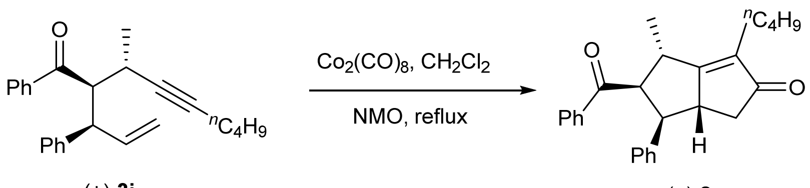

A dry schlenk tube (100 mL) was flame dried and flushed with Argon. Enyne **3i**(69 mg, 0.2 mmol, 1.0 equiv) was added slowly to a stirred solution of $Co_2(CO)_8$ (81 mg, 0.2 mmol, 1.0 equiv) in CH_2Cl_2 (5.0 mL) at 0°C. Stirring was continued at room temperature for 24 h. After the reaction mixture was cooled to 0°C (ice-bath), NMO in CH_2Cl_2 (2.0 mL) was added slowly. The reaction mixture was heated to reflux for 36 h, the reaction was complete (monitored by TLC). The reaction mixture was concentrated under reduced pressure to afford a crude product, which was purified by chromatography (eluting with petroleum ether/EtOAc 10/1) to yield the product **8** (50.2 mg, yield 70%).White solid, mp: 98-102°C; ^1H NMR (400 MHz, $CDCl_3$): δ 7.55 (d, *J* = 7.2 Hz, 2H), 7.36 (t, *J* = 7.6 Hz, 2H), 7.26-7.18 (m, 2H), 7.10-7.01 (m, 4H), 7.01-6.90 (m, 1H), 4.15 (dd, *J* = 9.6, 4.4 Hz, 1H), 3.78-3.68 (m, 2H), 3.21 (dd, *J* = 12.4, 9.2 Hz, 1H), 2.60 (dd, *J* =17.6, 5.2 Hz, 1H), 2.42-2.34 (m, 1H), 2.33-2.22 (m, 1H), 2.01 (dd, *J* = 18.0, 3.6 Hz, 1H), 1.48 (d, *J* = 7.6 Hz, 3H), 1.50-1.30 (m, 4H), 0.93 (t, *J* = 7.2 Hz, 3H);^{13}C NMR (101 MHz, $CDCl_3$): 210.0, 202.1, 182.5, 138.1, 137.7, 137.3, 132.7, 128.2, 128.1, 128.0, 126.9, 60.4, 52.8, 47.7, 40.9, 38.9, 31.3, 22.9, 22.8, 17.9, 13.9; MS (EI) m/z (rel): 372 (M^+, 18), 344 (6), 315 (11), 281 (12),

267(24), 165(7), 128 (6), 115(12), 105 (100), 91(27), 77 (61), 65 (4), 51(7); IR (film): *v* 2963 (w), 2919 (w), 1661 (m), 1092 (m), 799 (s) cm^{-1}; HRMS (EI) Calcd. for $C_{26}H_{28}O_2$ (M^+): 372.2089; Found: 372.2084.

4. Synthesis of cyclopentene 10 via metathesis reaction of desilylated product 3l

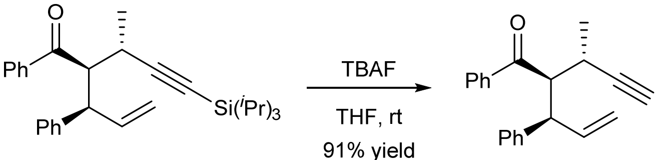

3l
90% ee

9
90% ee

To a solution of **3l** (65.2 mg, 0.15 mmol) in THF (2.0 mL) was added tetrabutylammonium fluoride (TBAF) solution (0.16 mL, 1.0 M in THF) at 0 °C, and the mixture was stirred at room temperature for 2 h. The mixture was quenched with H_2O and extracted with ethyl acetate. The combined organic layer was washed with brine, dried over $MgSO_4$, filtered, and concentrated under reduced pressure. The residue was subjected to a column chromatography on silica gel (hexane/ethyl acetate = 10/1) to give compound **9** (39.2 mg, yield 91%).ee: 90%; $[\alpha]_D^{28}$=39.8 (*c*1.0, $CHCl_3$); Yellow oil; ^{1}H NMR (400 MHz, $CDCl_3$): δ 7.71 (d, *J* = 7.6 Hz, 2H), 7.45 (t, *J* = 7.6 Hz, 1H), 7.18 (m, 4H), 7.10 (m, 1H), 6.20-6.10 (m, 1H), 5.18 (d, *J* = 16.4 Hz, 1H), 5.10 (dd, *J* = 10.4, 1.6 Hz, 1H), 4.12 (t, *J* = 7.2 Hz, 1H), 3.99 (t, *J* = 7.6 Hz, 1H), 2.90-2.80 (m, 1H), 2.12 (d, *J* = 2.4 Hz, 1H), 1.14 (d, *J* = 7.2 Hz, 3H); ^{13}C NMR (101 MHz, $CDCl_3$): 202.0, 142.0, 139.1, 136.8, 133.0, 128.6, 128.4, 128.2, 128.0,126.7, 117.4, 87.0, 71.0, 54.7, 51.0, 27.2, 17.8; MS (EI) m/z (rel): 288 (M^+, 2), 235 (9), 220 (20), 197 (12), 183 (17), 171 (6), 141 (9), 128 (12), 117 (96), 105 (100), 91 (27), 77 (66), 65 (7), 51 (17); IR (film): *v* 3297(m), 2979 (w), 1674 (s), 1493 (m), 1247 (s), 921 (m), 758 (m), 701 (s) cm^{-1}; HRMS (EI) Calcd. for $C_{21}H_{20}O$ (M^+): 288.1514; Found: 288.1508; HPLC (Agilent SDC-OJ, CO_2/*i*-Propanol = 95/5, 1.3 mL/min, 214 nm), t_R (minor) = 6.80min, t_R(major) = 8.69 min.

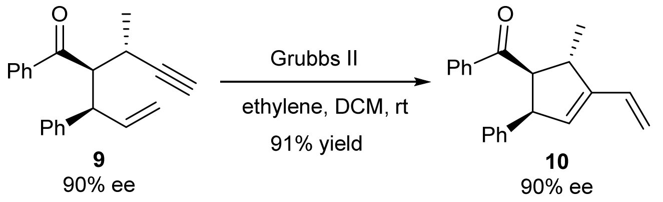

Freshly distilled CH_2Cl_2 was degassed for 1 h. The enyne **9** (7.0 mg, 0.024 mmol) was dissolved in CH_2Cl_2 (4 mL) and ethylene gas was passed through the solution for 30 min. Grubbs**II** catalyst(3.0 mg, 15% mol) was then added, and the solution was degassed again with ethylene for 30 min. The mixture was stirred under an atmosphere of ethylene at room temperature. The reaction was monitored by TLC (ca.2h). The mixture was then concentrated and the residue was purified by neutral alumina (hexane/ethyl acetate = 10/1) to give compound **10** (6.5 mg, yield 91%). ee: 90%; $[\alpha]_D^{30}$= -216.4 (*c*0.5, $CHCl_3$); Yellow solid, mp: 69-75°C; ^{1}H NMR (400 MHz, $CDCl_3$): δ 7.62 (d, J = 7.6 Hz, 2H), 7.44 (t, J = 7.2 Hz, 1H), 7.35-7.29 (m, 2H), 7.01–6.96 (m, 3H), 6.74-6.70 (m, 2H), 6.51 (dd, J = 18.4, 11.2 Hz, 1H), 5.67 (s, 1H), 5.42 (d, J = 18.4 Hz, 1H), 5.23 (d, J = 10.0 Hz, 1H), 4.41 (d, J = 10.8 Hz, 1H), 4.11 (dd, J = 7.2 Hz, J = 9.6 Hz, 1H), 3.95-3.85 (m, 1H), 1.26 (d, J = 7.2 Hz, 3H); ^{13}C NMR (101 MHz, $CDCl_3$): 199.6, 146.9, 139.5, 138.0, 132.4, 132.0, 130.5, 128.5, 128.2, 127.9, 127.8, 126.6, 115.7, 60.9, 53.9, 39.4, 19.7; MS (EI) m/z (rel): 288 (M^+, 22), 273 (30), 183 (28), 168 (24), 141 (13), 105 (100), 91 (26), 77 (60), 51 (12); IR (film): ν 2961 (w), 2926 (w), 1672 (m), 1245 (m), 1023 (m), 692(s) cm^{-1}; HRMS (EI) Calcd. for $C_{21}H_{20}O$ (M^+): 288.1514; Found: 288.1518. HPLC (Agilent SDC-OD-H, CO_2/*i*-Propanol = 90/10, 1.3 mL/min, 214 nm), t_R (minor) = 8.83min, t_R(major) = 10.49 min.

Mechanistic studies.

The ally compound(*S*)-(*Z*)-**5** is prepared according to the literature procedure[4,5].

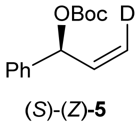

(*S*)-(*Z*)-**5**
ee > 99%, Z/E = 94/6

(*S*)-(*Z*)-**5**, ee = 99.3%, $[\alpha]_D^{23}$= -32.7 (*c*1.0, $CHCl_3$); ^{1}H NMR (400 MHz, $CDCl_3$): δ 7.37-7.26 (m, 5H), 6.04-6.01 (m, 2H), 5.24 (dt, *J* = 9.2, 4.0 Hz, 1H), 1.47 (s, 9H); HPLC: Chiralcel OJ-H (25 cm × 0.46 cm), hexane/2-propanol = 95/5, 0.7mL/min, 214 nm, t_R = 7.58 min (minor), 8.90 min (major).

1. The reaction of ketone 1a with (*S*)-(*Z*)-5 catalyzed by[Pd(η^3-C_3H_5)Cl]$_2$/S-IPr

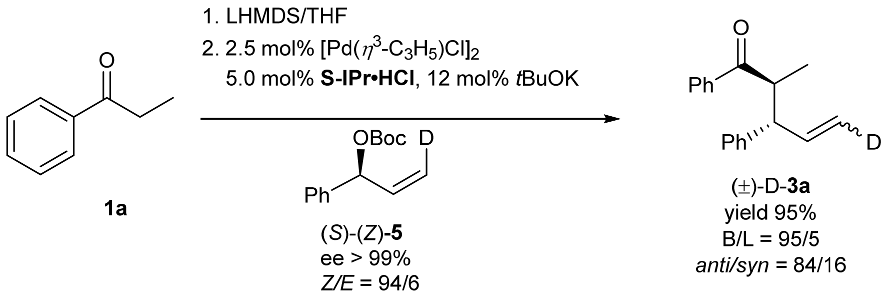

A dry schlenk tube was flame dried and flushed with Argon. Ketone **1a** (54 mg, 0.4 mmol) was and THF (2.0 mL) were added into the dry Schlenk tube. LHMDS(1.0 M in THF, 0.4 mL, 0.4 mmol) were added at 0°C and stirred at room temperature for 30 min. In a separated flushed flask, [Pd(η^3-C_3H_5)Cl]$_2$ (1.9 mg, 0.005 mmol), S-IPr·HCl (4.4 mg, 0.006 mmol) and THF (1.0 mL) were added, followed by addition of *t*-BuOK (1.0 M in THF, 25 uL, 0.025 mmol) at 0°C. The resulting mixture was stirred at room temperature for 30 min.The substrates (*S*)-(*Z*)-**5** (47 mg, 0.2 mmol) and THF (1.0 mL) was then added and the mixture was stirred at 30 °C. After the reaction was complete, the reaction mixture was quenched by H_2O (0.5 mL), the solution was dried (anhydrous Na_2SO_4) and then filtered through a 0.5 inch plug of silica gel (eluting with EtOAc) to remove the solid. The crude reaction mixture was concentrated under reduced pressure. $CDCl_3$ (0.8 mL) was added to dissolve the crude reaction mixture, and mesitylene (23 μL) was added as an internal standard. The diastereoselectivity was then determined by ^{1}H NMR spectroscopy. After this analysis, the crude reaction mixture was purified by flash column silica gel chromatography (eluting with petroleum ether/ethyl acetate 10/1) to afford the product D-**3a**, which was mixture of (*E*)- and (*Z*)-isomer.

Chiral HPLC separation of deutorated products (*R,R*)-(*E*)-**3a and** (*S,S*)-(*Z*)-**3a**

The “racemic” (*R,R*)-(*E*)-**3a** and (*S,S*)-(*Z*)-**3a** products were separated by a preparative OJ-H (25 cm × 0.46 cm)column, hexane/2-propanol =95/5, 8.0 mL/min, 214 nm. The absolute configuration of the product **3a** was determined by comparing the reported[1].

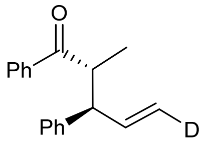

(*R,R*)-(*E*)-**3a**

98% ee, $[\alpha]_D^{26}$= 44.6 (*c* 0.7, $CHCl_3$); ^{1}H NMR (400 MHz, $CDCl_3$):δ 7.80-7.78 (m, 2H), 7.52-7.07 (m, 8H), 6.08-5.98 (m, 1H), 5.12 (d, *J* = 17.2Hz, 1H), 3.97-3.91 (m, 1H), 3.72-3.68 (m, 1H), 1.20 (d, *J* = 6.8Hz, 1H); ^{13}C NMR (101 MHz, $CDCl_3$): 203.4, 142.8, 138.9, 137.0, 132.7, 128.5, 128.4, 128.0, 127.6, 126.3, 116.6 (t, *J* = 23.6 Hz), 53.0, 45.2, 16.5; MS (EI) m/z (rel): 251 (M^+, 2), 236 (19), 118 (61), 105 (100), 92 (10), 77 (42), 51 (8); HRMS (EI) Calcd. for $C_{18}H_{17}DO$ (M^+): 251.1420; Found: 251.1419; HPLC: Chiralcel OJ-H, hexane/2-propanol =90/10, 0/7 mL/min, 214 nm, t_R (major) = 9.10 min, t_R (minor) =10.2 min.

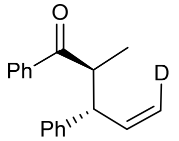

(*S,S*)-(*Z*)-**3a**

98% ee, $[\alpha]_D^{26}$= -37.7 (*c* 0.8, $CHCl_3$); ^{1}H NMR (400 MHz, $CDCl_3$): δ 7.80-7.78 (m, 2H), 7.52-7.07 (m, 8H), 6.08-5.98 (m, 1H), 5.10 (d, *J* = 10.4 Hz, 1H), 3.97-3.91 (m, 1H), 3.72-3.68 (m, 1H), 1.20 (d, *J* = 6.8Hz, 1H); ^{13}C NMR (101 MHz, $CDCl_3$): 203.4, 142.8, 138.9, 137.0, 132.7, 128.5, 128.4, 128.0, 127.6, 126.3, 116.3 (t, *J* = 23.3Hz), 53.0, 45.2, 16.5; MS (EI) m/z (rel): 251 (M^+, 2), 236 (19), 118 (61), 105 (100), 92 (10), 77 (42), 51 (8); HRMS (EI) Calcd. for $C_{18}H_{17}DO$ (M^+): 251.1420; Found: 251.1427;HPLC: Chiralcel OJ-H, hexane/2-propanol =90/10, 0.7 mL/min, 214 nm, t_R (minor) = 9.20 min, t_R (major) = 10.2 min.

The absolute configuration of the product was determined to be (*S,S*) by comparing the reported HPLC traces of (*S,S*)-**3a**[1] {Chiralcel OJ-H Column 95:5 hexanes/2-propanol, 0.7 mL/min, 254 nm, t_R(minor) = 9.9 min, t_R (major)= 11.1 min. 98% ee, ($[\alpha]_D^{25}$= -56.9, c 1.41 in $CHCl_3$) }.

2. The reaction of β-alkenyl-ketone (*S*)-1h with (*S*)-(*Z*)-5 catalyzed by [Pd(η^3-C_3H_5)Cl]$_2$/S-IPr.

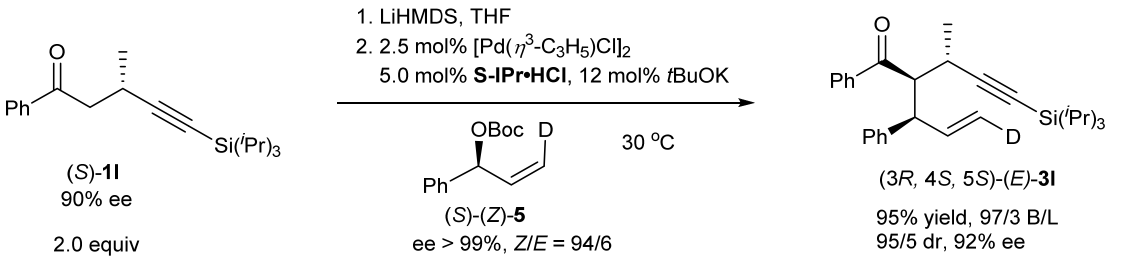

A dry schlenk tube was flame dried and flushed with Argon. Ketone (*S*)-**1l**(131.2 mg, 0.4 mmol) was and THF (2.0 mL) were added into the dry Schlenk tube. LHMDS(1.0 M in THF, 0.4 mL, 0.4mmol) were added at 0°C and stirred at room temperature for 30 min. In a separated flask, [Pd(η^3-C_3H_5)Cl]$_2$ (1.9 mg, 0.005 mmol), S-IPr·HCl (4.4 mg, 0.006 mmol) and THF (1.0 mL) were added, followed by addition of *t*-BuOK (1.0 M in THF, 25 uL, 0.025 mmol) at 0°C.The resulting mixture was stirred at room temperature for 30 min, then added to the ketone solution. the substrates (*S*)-(*Z*)-**5** (47 mg, 0.2 mmol) and THF (1.0 mL) was then added and the mixture was stirred at 30 °C. After the reaction was complete, the reaction mixture was quenched by H_2O (0.5 mL). The solution was dried (anhydrous Na_2SO_4) and then filtered through a 0.5 inch plug of silica gel (eluting with EtOAc) to remove the solid. The crude reaction mixture was concentrated under reduced pressure. $CDCl_3$ (0.7-0.8 mL) was added to dissolve the crude reaction mixture, and mesitylene (23 μL) was added as an internal standard. The diastereoselectivity was then determined by ^{1}H NMR spectroscopy. After this analysis, the crude reaction mixture was purified by flash column silica gel chromatography (eluting with petroleum ether/toluene 1/1) to yield the product (3*R*,4*S*,5*S*)-(*E*)-**3l** (85.1 mg, yield 95%; B/L: 97/3; *dr*: 95/5). ee = 92%, $[\alpha]_D^{28}$= 5.69 (*c* 0.5, $CHCl_3$); Yellow oil; ^{1}H NMR (400 MHz, $CDCl_3$): δ 7.67 (d, *J* = 7.6 Hz, 2H), 7.42 (t, *J* = 7.6 Hz, 1H), 7.30-7.25 (m, 2H), 7.20-7.15 (m, 4H), 7.12-7.07 (m, 1H), 6.17 (dd, *J* = 16.8, 9.2 Hz, 1H), 5.20 (d, *J* = 16.8 Hz, 1H), 4.20 (dd, *J* =9.2, 6.8 Hz, 1H), 3.96 (dd, *J* = 8.0, 6.4 Hz, 1H), 2.95-2.85 (m, 1H), 1.14 (d, *J* = 6.8 Hz, 3H), 1.10-1.00 (m, 21H); ^{13}C NMR (101 MHz, $CDCl_3$): 202.1, 141.9, 139.1, 136.6, 132.6, 128.6, 128.3, 128.1, 127.9, 126.6, 117.4 (t, *J* = 24.0 Hz), 111.3, 83.0, 55.2, 51.2, 28.6, 18.6, 18.6, 18.3, 11.2; MS (EI) m/z (rel): 445 (M^+, 0.58), 402 (10), 360 (9), 131 (6), 118 (100), 105 (12), 92 (6), 77 (12), 51 (2); IR

(film): ν 2940 (m), 2863 (m), 2161 (w), 1671 (m), 1449 (m), 1206 (m), 883 (m), 674 (s) cm^{-1}; HRMS (EI) Calcd. for $C_{30}H_{39}DOSi$ (M^+): 445.2911; Found: 445.2907. HPLC (Agilent SDC-OJ, CO_2/*i*-Propanol = 95/5, 1.3 mL/min, 214 nm), t_R (major) = 4.58 min, t_R(minor) = 5.80 min.

3. **The reaction of 1c with 11 catalyzed by [Pd(η^3-C_3H_5)Cl]$_2$/S-IPr.**

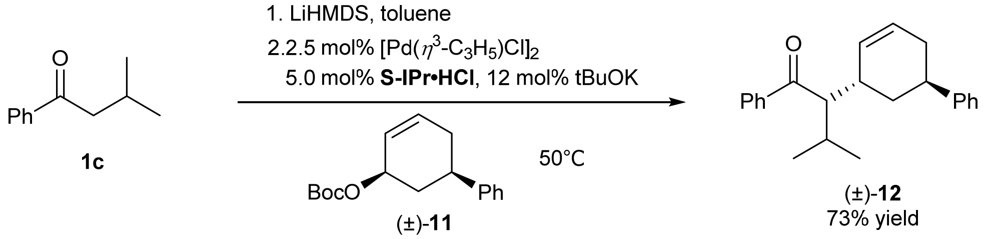

A dry schlenk tube was flame dried and flushed with Argon. Ketone **1c** (65 mg, 0.4 mmol) was and toulene (2.0 mL) were added into the dry Schlenk tube. LHMDS(1.0 M in THF, 0.4 mL, 0.4mmol) were added at 0°C and stirred at room temperature for 30 min. In a separated flask, [Pd(η^3-C_3H_5)Cl]$_2$ (1.9 mg, 0.005 mmol), S-IPr·HCl (4.4 mg, 0.006 mmol) and toulene (1.0 mL) were added, followed by addition of *t*-BuOK (1.0 M in THF, 25 uL, 0.025 mmol) at 0°C.The resulting mixture was stirred at room temperature for 30 min, then added to the ketone solution. the substrates **11** (54 mg, 0.2 mmol) and toulene (1.0 mL) was then added and the mixture was stirred at 30 °C. After the reaction was complete, the reaction mixture was quenched by H_2O (0.5 mL). The solution was exacted with ethyl acetate and dried (anhydrous Na_2SO_4), then concentrated under reduced pressure and purified by flash column silica gel chromatography (eluting with petroleum ether/ethyl acetate 10/1) to yield the product **12** (46.4 mg, yield 73%). ^{1}H NMR (600 MHz, $CDCl_3$): δ 8.01-7.93 (m, 2H), 7.59-7.51 (m, 1H), 7.49-7.43 (m, 2H), 7.31-7.23 (m, 2H), 7.21-7.16 (m, 1H), 7.14-7.08 (m, 2H), 5.83-5.73 (m, 1H), 5.73-5.64 (m, 1H), 3.51 (dd, *J* = 8.4, 6.7 Hz, 1H), 2.90-2.81 (m, 1H), 2.80-2.72 (m, 1H), 2.36-2.28 (m, 1H), 2.23 (dq, *J* = 13.6, 6.8 Hz, 1H), 2.19-2.12 (m, 1H), 1.92-1.86 (m, 2H), 0.91 (d, *J* = 6.7 Hz, 3H), 0.89 (d, *J* = 6.9 Hz, 3H). ^{13}C NMR (101 MHz, $CDCl_3$) δ 204.54, 146.37, 139.82, 132.69, 128.96, 128.54, 128.29, 128.16, 127.31, 126.85, 126.00, 54.85, 36.07, 34.68, 33.32, 32.29, 28.67, 21.33, 18.76; IR: 2959, 1736, 1596, 1579, 1493, 1447 cm^{-1}; HRMS (ESI) Calcd for $C_{23}H_{27}O$ (M+H]$^+$): 319.2062; Found 319.2058.

Computational methods

All calculations were done with the Gaussian 09 program[6]. Gas phase geometries were optimized with the ωB97XD functional[7] of the density functional theory (DFT). The SDD[8] basis set with Stuttgart/Dresden effective core potentials[9,10] (SDDAll) was employed for all atoms. Frequency analyses were performed at the same level of theory, to confirm that each optimized geometry is either an energy minimum (with no imaginary frequencies) or a transition state (with only one imaginary frequency), and to provide gas phase free energy corrections. Solvation free energies in toluene were computed at each optimized geometry with a larger basis set, def2-TZVP[11.12], using the SMD solvation method[13]. To account for the different standard states in the gas-phase (1 atm, 298.15 K) and in solution (1 mol/liter, 298.15 K) when computing the thermal correction in solution, a correction of 1.89 kcal/mol is added to the total energy for each structure[14,15]. The final free energy for each structure, G_{sol}, comprises the SMD energy in solvent, the 1.89 kcal/mol thermal energy correction for the strandard state in solution and the free energy correction of the gas phase. Reported energies in this study are relative free energies ΔG_{sol}. Cartesian coordinates and absolute energies of all calculated structure can be found in a separate file called Supplimental Data 1.

DFT results and discussions

It is proposed (Supplementary **Figure 67**) that ketone **1b** is deprotonated by LiHMDS to generate **lithium enolate**, and **2a** reacts with NHC and Pd(0) to give the **allyl-Pd complex**. Then, lithium enolate and allyl-Pd complex can react to yield the branched product via either an outer-sphere or inner-sphere mechanism.

It should be noted that the structure of lithium enolate generated in the reaction condition is not as simple as what we drew in the above figure. Organolithium compounds usually exist in aggregate forms, such as dimer, trimer, tetramer and so on in solid-state[16]. Solvent and external ligands may change the aggregate form of these compounds. For example, LiHMDS in THF solvent has a dimer structure[17]. In the current reaction conditions, we have no knowledge of the exact structure of the lithium enolate. However, it is reasonable to assume that lithium enolate exists as dimers, possibly with two external amine ligands for each dimer (Supplementary **Figure 68**). It has been reported that under similar conditions, Z-type enolate is formed

dominantly[18,19]. Our experiment also showed that the Z-isomer is indeed the major product. We used the dimer structure of Z-enolate without external amine ligands in our calculations.

In addition to the key structures shown in Figure 6 of the manuscript, we also calculated intermediates and transition stuctures containing η^1-allyl-Pd complex (Supplementary **Figure 69**) and E-enolate (Supplementary **Figure 71**).

The following section presents the analysis and calculations on possible conformations of the transitition state that leads to branched product via an inner-sphere pathway (TS-inner-branched in Figure 6 of the manuscript).

In order to model the seven-membered ring transition state of this C-C bond-forming step, we consulted a conformational study on saturated seven-membered rings[20]. In cycloheptane (Supplementary **Figure 70**), the *chair* and *boat* conformations are not stable (not energy minimun) because of the repulsion between the two eclipsing CH_2 groups (marked by red arrows); instead, they are transition states connecting, respectively, a pair of *twist-chair* and *twist-boat* conformations.

To construct the structure of TS-inner-branched (Figure 6 of the manuscript), one needs to replace two adjacent CH_2 groups in cycloheptane with the –O– and –Pd(NHC)– groups, and also to add necessary substituents. We started with the *chair* and *boat* conformations of cycloheptane. In order to eliminate the repulsion between the two eclipsing CH_2 groups, we replaced one of them with the –O– and an adjacent CH_2 group with the –Pd(NHC)– group. Then we added two phenyls and one ethyl to the seven-membered ring at proper positions to obtain transition state TS-inner-branched for substrate **1b**. Note that if one starts with the *twist-chair* and *twist-boat* conformations, the same structures will be obtained after geometry optimization.

Supplementary **Figure 71** illustrates this substitution process and shows the resulting transition state structures. To show more clearly the difference between the two conformations, in the drawings of both conformations in Supplementary **Figure 71**, we placed the phenylallyl group in the front with the same orientation and the enolate in the back. Since enantioselectivity is not involved in the reaction using achiral NHC ligands, we only considered the *si* face of phenylally being attacked by enolate in our calculations.

A total of four transition state structures can be constructed when both the *E/Z* isomers of enolate are considered. As can be seen in Supplementary **Figure 71**, the difference between the *chair* and *boat* transition states with the same enolate configuration (**TS1** vs **TS3**, both with *E*-enolate) is that, in **TS1**, it is the *re* face of enolate that attacks the phenyl allyl, whereas in **TS3**, the enolate attacks with its *si* face.

We also considered transition state structures generated by substitution of –O– and –Pd(NHC)– for two adjacent CH_2 groups in cycloheptane at different positions. This leads to four more transition states, **TS5** to **TS8**. Their structures and energies are given in the Absolute energies and coordinates section later in this document. Calcultiaons show that these transition states are at least 3.2 kcal/mol higher than **TS2**.

Based on the transition state structures for **1b**, we added a methyl group to the β position of the enolate, thus obtaining the transition state structures for **1c**. These structures and their relative energies are shown in Supplementary **Figure 72**. We also considered the three conformers arising from the rotation of the C(α)-C(β) bond in the enolate.

Among the three *chair* transition states for **1c**, **TS2a-Me** has the lowest energy although it suffers from the 1,3-diaxial strain between one methyl at the β position of enolate and the phenyl group of phenylally. **TS2b-Me** suffers from the repulsion between one methyl at the β position of enolate and the bulky substituent on NHC, which is not obvious in this schematic drawing. **TS2b-Me** is computed to be 1.8 kcal/mol higher than **TS2a-Me**, indicating that the repulsion between the methyl at the β position of enolate and NHC is stronger than the 1,3-diaxial strain. **TS2c-Me** suffers from both, and thus has the highest energy. Among the three *boat* transitions states, **TS4a-Me** is the lowest energy one. **TS4c-Me** is computed to be 4.6 kcal/mol higher than **TS4a-Me** due to the repulsion between one methyl at the β position of enolate and the bulky substituent on NHC, which is also not obvious in this schematic drawing.

Next, based on the transition state structures for **1b**, possible transition state structures for **1k** were constructed by adding a phenyl group to the β position of the enolate (Supplementary **Figure 73**). In each of these transition states, only one conformation, in which the hydrogen at β position is pointing to NHC, were considered. The boat transition state **TS4a-Ph** is predicted to

be the lowest energy transition state; the product generated via **TS4a-Ph** has the same stereo configuration as the one produced in experiments.

Supplementary References

1. Zheng, W.-H., Zheng, B.-H., Zhang, Y. & Hou, X.-L. Highly Regio-, Diastereo-,and Enantioselective Pd-Catalyzed Allylic Alkylation of Acyclic Ketone Enolates with Monosubstituted Allyl Substrates. *J. Am. Chem. Soc.* **129**, 7718-7719 (2007).
2. Endo, K., Ogawa, M. & Shibata, T. Multinuclear catalyst for copper-catalyzed asymmetric conjugate addition of organozinc reagents. *Angew. Chem., Int. Ed.* **49**, 2410-2413 (2010).
3. Nishimura, T., Guo, X. -X., Uchiyama, N., Katoh, T. & Hayashi, T. Steric tuning of silylacetylenes and chiral phosphine ligands for Rhodium-catalyzed asymmetric conjugate alkynylation of enones. *J. Am.Chem. Soc*. **130**, 1576-1577 (2008).
4. Liao, S. P. & Collum, D. B. Lithium diisopropylamide-mediated lithiations of imines: insights into highly structure-dependent rates and selectivities. *J. Am. Chem. Soc.* **125**, 15114-15127 (2003).
5. Chen, J.-P., Peng, Q., Lei, B.-L., Hou, X.-L. & Wu, Y.-D. Chemo- and regioselectivity-tunable Pd-catalyzed allylic alkylation of imines. *J. Am.Chem. Soc.* **133**,14180-14183 (2011).
6. Gaussian 09, Revision D.01, M. J. Frisch, G. W. Trucks, H. B.Schlegel, G. E. Scuseria, M. A. Robb, J. R. Cheeseman, G. Scalmani, V. Barone, B. Mennucci, G. A. Petersson, H. Nakatsuji, M. Caricato, X. Li, H. P. Hratchian, A. F. Izmaylov, J. Bloino, G. Zheng, J. L. Sonnenberg, M. Hada, M. Ehara, K. Toyota, R. Fukuda, J. Hasegawa, M. Ishida, T. Nakajima, Y.Honda, O. Kitao, H. Nakai, T. Vreven, J. A., Jr. Montgomery, J. E. Peralta, F. Ogliaro, M. Bearpark, J. J. Heyd, E. Brothers, K. N. Kudin,V. N. Staroverov, R. Kobayashi, J. Normand, K. Raghavachari, A. Rendell, J. C. Burant, S. S.Iyengar, J. Tomasi, M. Cossi, N. Rega, J. M. Millam, M. Klene, J. E.Knox, J. B.Cross, V. Bakken, C. Adamo, J. Jaramillo, R. Gomperts, R. E. Stratmann, O. Yazyev, A. J.Austin,R. Cammi,C. Pomelli, J. W.Ochterski, R. L. Martin, K.Morokuma, V. G.Zakrzewski, G. A.Voth, P. Salvador, J. J.

Dannenberg, S. Dapprich, A. D. Daniels, Ö. Farkas,J. B.Foresman, J. V. Ortiz, J. Cioslowski, Fox, D. J. Gaussian, Inc., Wallingford CT, 2009.

7. Chai, J.-D. & Head-Gordon, M. Long-range corrected hybrid density functionals with damped atom–atom dispersion corrections. *Phys. Chem. Chem. Phys.* **10**, 6615-6620 (2008).
8. Dunning Jr. T. H., Hay, P. J., in *Modern Theoretical Chemistry*, Ed. Schaefer, H. F. III, Vol. 3 (Plenum, New York, 1977) 1-28.
9. Igel-Mann, G., Stoll, H. & H. Preuss, "Pseudopotentials for main group elements (IIIA through VIIA)," *Mol. Phys.* **65**, 1321-1328 (1988).
10. Andrae, D., Haeussermann, U., Dolg, M., Stoll, H. & Preuss, H. Energy-adjusted ab initio pseudopotentials for the 2nd and 3rd row transition-elements. *Theor. Chem. Acc.*, **77**, 123-141 (1990).
11. Weigend, F. & Ahlrichs, R. Balanced basis sets of split valence, triple zeta valence and quadruple zeta valence quality for H to Rn: Design and assessment of accuracy. *Phys. Chem. Chem. Phys.* **7**, 3297-3305 (2005);
12. Weigend, F. Accurate Coulomb-fitting basis sets for H to Rn. *Phys. Chem. Chem. Phys.* **8**, 1057-1065 (2006).
13. Marenich, A. V., Cramer, C. J. & Truhlar, D. G. Universal Solvation Model Based on Solute Electron Density and on a Continuum Model of the Solvent Defined by the Bulk Dielectric Constant and Atomic Surface Tensions. *J. Phys. Chem. B*, **113**, 6378-6396 (2009).
14. Harvey, J. N. Ab initio transition state theory for polar reactions in solution. *Faraday Discuss.* **145**, 487–505 (2010).
15. 1. General, I. J. A Note on the Standard State's Binding Free Energy. *J. Chem. Theory Comput.* 6, 2520-2524 (2010).

16. 1. Stey, T. & Stalke, D. Lead structures in lithium organic chemistry. *PATAI'S Chemistry of Functional Groups* (John Wiley & Sons, Ltd, 2009).

17. Lucht, B. L. & Collum, D. B. Ethereal Solvation of Lithium Hexamethyldisilazide: Unexpected Relationships of Solvation Number, Solvation Energy, and Aggregation State. *J. Am. Chem. Soc.* **117**, 9863–9874 (1995).

18. Heathcock, C. H. et al. Acyclic Stereoselection. 7. Stereoselective Synthesis of 2-Alkyl-3-hydroxy Carbonyl Compounds by Aldol. *J. Org. Chem.* **45**, 1066–1081 (1980).

19. Xie, L., Isenberger, K. M., Held, G. & Dahl, L. M. Formation: Steric vs Electronic Effects. **62**, 7516–7519 (1997).

20. Entrena, A. Rules for predicting the conformational behavior of saturated seven-membered heterocycles. *Arkivoc* **2005**, 88–108 (2005).
