# Supplementary material for: Palladium/N-heterocyclic carbene catalysed regio and diastereoselective reaction of ketones with allyl reagents via inner-sphere mechanism
Source: Nat Commun. 2016 Jun 10;7:11806. doi: 10.1038/ncomms11806 (PMC4906412; doi:10.1038/ncomms11806)
Supplement: Supplementary Data 1 — Energies and coordinates of calculated structures [file ncomms11806-s2.pdf]

Energies and coordinates of calculated structures

Energies are in hartree; cartesian coordinates are in angstrom. Pictures are generated by CYLview [Legault, C. Y.mCYLview, 1.0b. Université de Sherbrooke. http://www.cylview.org (2009).]. Carbon and hydrogen atoms are in grey, oxygen in red, nitrogen in blue, palladium in turquoise. Some atoms of the bulky substituent on NHC are omitted for clarity.

**lithium enolate**				**^{t}BuOLi**			
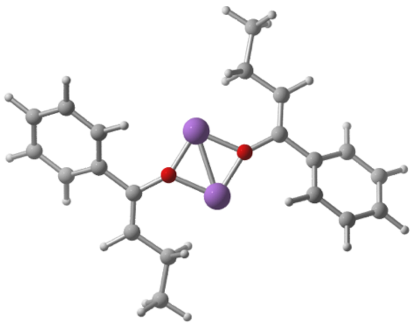				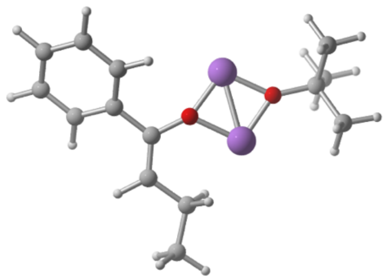			
Gas phase energy= -159.313305075<br>Gas phase free energy correction= 0.322940<br>SMD energy= -941.086062905				Gas phase energy= -124.107569174<br>Gas phase free energy correction= 0.269379<br>SMD energy= -711.247903459			
C	2.5663510000	2.3464830000	-0.1248540000	C	-1.4451320000	2.0087180000	-0.1212910000
C	2.4985070000	1.0051220000	0.0252230000	C	-1.3198400000	0.6631750000	-0.0831700000
O	1.3012720000	0.3909920000	0.1745420000	O	-0.0965060000	0.0859000000	-0.1166550000
C	3.6936090000	0.1160610000	0.0529590000	C	-2.4818890000	-0.2678960000	-0.0263240000
C	3.6708040000	-1.0603170000	0.8178220000	C	-2.3941370000	-1.5296930000	-0.6335550000
C	4.8485960000	0.4120960000	-0.6872360000	C	-3.6696690000	0.0765620000	0.6370650000
C	4.7755640000	-1.9116670000	0.8539940000	C	-3.4691750000	-2.4180230000	-0.5942070000
H	2.7901560000	-1.2872180000	1.4120250000	H	-1.4847380000	-1.7963450000	-1.1632900000
C	5.9539250000	-0.4382120000	-0.6510850000	C	-4.7449090000	-0.8116240000	0.6774620000
H	4.8690400000	1.3002390000	-1.3102220000	H	-3.7403020000	1.0353060000	1.1402330000
C	5.9223800000	-1.6036320000	0.1185570000	C	-4.6496490000	-2.0622110000	0.0622710000
H	4.7454160000	-2.8112960000	1.4604850000	H	-3.3884280000	-3.3850820000	-1.0803210000
H	6.8372400000	-0.1966790000	-1.2330150000	H	-5.6542040000	-0.5321370000	1.1993850000
H	6.7809140000	-2.2662630000	0.1421890000	H	-5.4849460000	-2.7535140000	0.0970350000
H	3.5263470000	2.8494930000	-0.1942170000	H	-2.4258890000	2.4740400000	-0.1524320000
C	1.3185830000	3.1906420000	-0.1584510000	C	-0.2334580000	2.9033620000	-0.1672040000
C	1.5968610000	4.6843840000	-0.3615650000	C	-0.5759370000	4.3974970000	-0.1464480000
H	0.6508730000	2.8481960000	-0.9745180000	H	0.4290980000	2.6852720000	0.6940290000
H	0.7529280000	3.0671220000	0.7874480000	H	0.3570600000	2.6960590000	-1.0833770000
H	0.6709400000	5.2685020000	-0.3695420000	H	0.3258250000	5.0176020000	-0.1803110000
H	2.1133130000	4.8519130000	-1.3125170000	H	-1.1290470000	4.6504520000	0.7642060000
H	2.2354460000	5.0675870000	0.4412520000	H	-1.2024070000	4.6599120000	-1.0055040000
C	-2.5663930000	-2.3464890000	-0.1247390000	O	2.5891180000	-0.5011570000	0.0630090000
C	-2.4984680000	-1.0051040000	0.0250780000	Li	1.5270390000	0.8928360000	-0.2327880000
O	-1.3011980000	-0.3909570000	0.1739570000	Li	1.0022900000	-1.2605180000	0.2045630000
C	-3.6935680000	-0.1160400000	0.0529200000	C	3.9857190000	-0.7700500000	0.1010680000
C	-3.6708160000	1.0602100000	0.8179810000	C	4.4434010000	-0.8441160000	1.5678600000
C	-4.8484740000	-0.4119370000	-0.6874440000	C	4.2571960000	-2.1084840000	-0.6072140000
C	-4.7755800000	1.9115450000	0.8542250000	C	4.7266050000	0.3665710000	-0.6254110000
H	-2.7902190000	1.2870280000	1.4123000000	H	4.2219750000	0.1025060000	2.0727300000
C	-5.9538120000	0.4383650000	-0.6512340000	H	3.8988720000	-1.6417420000	2.0859430000
H	-4.8688340000	-1.2999710000	-1.3105920000	H	5.5182140000	-1.0441950000	1.6523240000
C	-5.9223350000	1.6036360000	0.1186310000	H	3.8976210000	-2.0580590000	-1.6408150000
H	-4.7454900000	2.8110560000	1.4608920000	H	5.3247160000	-2.3577320000	-0.6180450000

H	-6.8370730000	0.1969470000	-1.2332930000	H	3.7222090000	-2.9162100000	-0.0923890000
H	-6.7808680000	2.2662680000	0.1423030000	H	5.8117360000	0.2121110000	-0.6352030000
H	-3.5264370000	-2.8494590000	-0.1937290000	H	4.3756920000	0.4326120000	-1.6619480000
C	-1.3186720000	-3.1907050000	-0.1585220000	H	4.5232960000	1.3215190000	-0.1238080000
C	-1.5970820000	-4.6844720000	-0.3613290000				
H	-0.6511840000	-2.8484240000	-0.9748430000				
H	-0.7527220000	-3.0670640000	0.7871720000				
H	-0.6711880000	-5.2686280000	-0.3695120000				
H	-2.1138490000	-4.8521070000	-1.3120910000				
H	-2.2354230000	-5.0675320000	0.4417500000				
Li	-0.3610980000	1.1127270000	0.1484760000				
Li	0.3611620000	-1.1126600000	0.1470910000				

**allyl-Pd complex**	**allyl-Pd complex-2**
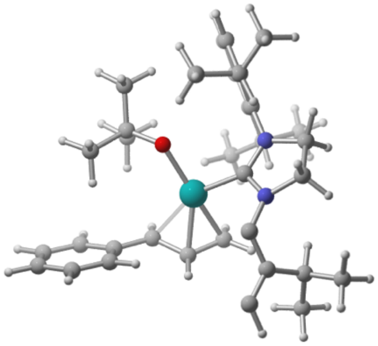	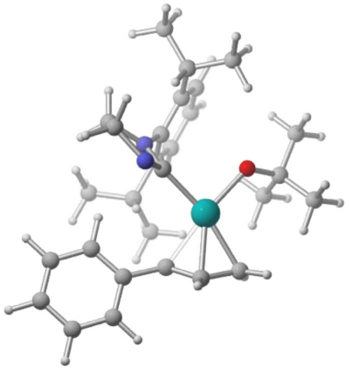
Gas phase energy= -424.354529979<br>Gas phase free energy correction= 0.816094<br>SMD energy= -1870.81779572	Gas phase energy= -424.344276892<br>Gas phase free energy correction= 0.819843<br>SMD energy= -1870.80621391

C	2.2097870000	-1.3095970000	-1.7952630000	C	0.3806730000	0.4993770000	-3.5232480000
H	1.7206470000	-1.8542430000	-2.6034430000	H	-0.2052630000	1.3702940000	-3.8087640000
C	2.1800210000	0.1023910000	-1.8540370000	C	1.5496130000	0.6663860000	-2.7397280000
C	1.0406050000	0.7492740000	-2.3928470000	C	1.5242730000	1.6056100000	-1.6862280000
H	2.9100740000	0.6897360000	-1.3027330000	H	2.3615310000	-0.0551180000	-2.7904100000
H	0.4991830000	0.2932320000	-3.2206920000	H	0.8198150000	2.4350960000	-1.8033470000
Pd	0.4078250000	-0.4102760000	-0.7143090000	Pd	-0.1080930000	0.1490350000	-1.4089440000
C	3.2595940000	-2.0524320000	-1.0709300000	C	0.7338750000	-1.0021430000	2.6885980000
C	3.5882220000	-3.3592560000	-1.4635220000	C	-0.1463010000	0.2286820000	2.9381530000
C	3.9183350000	-1.5042550000	0.0443840000	H	0.2656920000	-1.9362320000	3.0163670000
C	4.5402550000	-4.0996180000	-0.7622460000	H	-0.9440670000	0.0518380000	3.6593630000
H	3.0807400000	-3.8009730000	-2.3165950000	C	-0.0094270000	-0.1408410000	0.6154530000
C	4.8726390000	-2.2419820000	0.7402360000	N	0.8631760000	-0.9931260000	1.2128570000
H	3.6505920000	-0.5124190000	0.3976930000	N	-0.6936820000	0.5019210000	1.5913280000
C	5.1871260000	-3.5450800000	0.3433650000	C	-1.9602040000	1.1676030000	1.4286300000
H	4.7714670000	-5.1123520000	-1.0753900000	C	-2.0204440000	2.4949820000	0.9686870000
H	5.3583640000	-1.8065720000	1.6074490000	C	-3.1225490000	0.4496580000	1.7718840000
H	5.9205320000	-4.1230890000	0.8953440000	C	-3.2757190000	3.1030490000	0.8650380000
H	0.9740520000	1.8287980000	-2.3458190000	C	-4.3563960000	1.0977980000	1.6534050000
C	-1.4566010000	3.1824290000	0.7792180000	C	-4.4358790000	2.4120540000	1.2039020000
C	-2.6987960000	2.4854270000	0.2065520000	H	-3.3492390000	4.1231010000	0.5044350000
H	-1.4734450000	3.2442520000	1.8731710000	H	-5.2665780000	0.5627580000	1.9064780000
H	-3.5368110000	2.4497090000	0.9043280000	C	1.5775490000	-2.0374180000	0.5224370000
C	-0.8448280000	1.0666230000	-0.0763110000	C	0.8441220000	-3.0593590000	-0.1266750000
N	-0.3739830000	2.2723790000	0.3428030000	C	2.9870480000	-2.0563730000	0.5546580000
N	-2.1865070000	1.1274520000	-0.0728760000	C	1.5621470000	-4.0519640000	-0.8039470000
C	-3.0742120000	0.0111800000	-0.2158780000	C	3.6560840000	-3.0847070000	-0.1204210000
C	-3.3480590000	-0.5184030000	-1.4852630000	C	2.9530960000	-4.0646240000	-0.8107910000
C	-3.6486220000	-0.5104730000	0.9580320000	H	1.0216600000	-4.8355520000	-1.3219020000
C	-4.2281450000	-1.6048460000	-1.5631000000	H	4.7403600000	-3.1166910000	-0.1050290000
C	-4.5304530000	-1.5862620000	0.8325860000	C	-0.6719080000	-3.1870490000	-0.0304030000
C	-4.8162150000	-2.1324020000	-0.4172290000	H	-1.1187910000	-2.2002590000	0.1120250000

H	-4.4550990000	-2.0413150000	-2.5308930000	C	3.8067880000	-1.0472270000	1.3450300000
H	-4.9765990000	-2.0202780000	1.7217430000	H	3.1567120000	-0.2018780000	1.5925170000
C	1.0072860000	2.5030440000	0.6627240000	C	-3.0844230000	-0.9846010000	2.2890980000
C	1.5954670000	1.7745940000	1.7218150000	H	-2.0485050000	-1.3382270000	2.2601050000
C	1.7467230000	3.4348320000	-0.0942270000	C	-0.7561720000	3.2573180000	0.6129440000
C	2.9590120000	1.9701720000	1.9706300000	H	-0.0156630000	2.5137820000	0.3183490000
C	3.1045790000	3.6023520000	0.2011770000	H	-5.4019990000	2.8960800000	1.1098760000
C	3.7102840000	2.8668140000	1.2154770000	H	3.4865310000	-4.8477290000	-1.3393060000
H	3.4373510000	1.4160600000	2.7703770000	C	-0.9299430000	4.2012930000	-0.5849560000
H	3.6958750000	4.3089270000	-0.3716500000	H	0.0468570000	4.5957490000	-0.8884490000
C	0.7894860000	0.8524710000	2.6306380000	H	-1.5659430000	5.0607890000	-0.3445320000
H	-0.1072160000	0.5217760000	2.1020610000	H	-1.3737460000	3.6736920000	-1.4364230000
C	1.1024010000	4.2881530000	-1.1796530000	C	-0.2047840000	4.0116680000	1.8366850000
H	0.1510680000	3.8138300000	-1.4525840000	H	-0.0313830000	3.3285090000	2.6758940000
C	-3.2987090000	0.0354940000	2.3373630000	H	-0.9135550000	4.7786030000	2.1704180000
H	-2.6718080000	0.9240550000	2.2069520000	H	0.7451620000	4.4993830000	1.5896960000
C	-2.7419170000	0.0635030000	-2.7541390000	C	4.9869510000	-0.4868940000	0.5374320000
H	-2.0026510000	0.8164600000	-2.4608010000	H	5.7354560000	-1.2587210000	0.3266760000
H	-5.4935340000	-2.9760730000	-0.4959130000	H	5.4824490000	0.3094120000	1.1007130000
H	4.7664920000	2.9992400000	1.4238800000	H	4.6467110000	-0.0656430000	-0.4120130000
C	-2.0021070000	-1.0055240000	-3.5756240000	C	4.3228020000	-1.6795070000	2.6552170000
H	-1.5166760000	-0.5463900000	-4.4448780000	H	3.5137460000	-2.1230190000	3.2453570000
H	-2.6900550000	-1.7733050000	-3.9478350000	H	4.8313630000	-0.9273190000	3.2690150000
H	-1.2369960000	-1.4998200000	-2.9680030000	H	5.0403730000	-2.4783200000	2.4342810000
C	-3.8197630000	0.7666930000	-3.6013170000	C	-1.0270850000	-4.0858890000	1.1723870000
H	-4.3273220000	1.5476460000	-3.0244580000	H	-0.6713510000	-3.6610740000	2.1178570000
H	-4.5791500000	0.0521910000	-3.9396250000	H	-0.5712900000	-5.0771220000	1.0596190000
H	-3.3696980000	1.2280720000	-4.4877180000	H	-2.1117060000	-4.2143250000	1.2417550000
C	1.9580790000	4.3947190000	-2.4557780000	C	-1.3159720000	-3.7328010000	-1.3124260000
H	2.8533760000	5.0025580000	-2.2849280000	H	-2.4010920000	-3.6118140000	-1.2447320000
H	1.3827220000	4.8784060000	-3.2524960000	H	-1.1016450000	-4.7977980000	-1.4672170000
H	2.2844910000	3.4141240000	-2.8145760000	H	-0.9823260000	-3.1603560000	-2.1809380000
C	0.8077790000	5.7048950000	-0.6413490000	C	-3.5751870000	-1.0544160000	3.7489700000
H	0.2275840000	5.6787750000	0.2865400000	H	-3.0348560000	-0.3540760000	4.3960560000
H	0.2551440000	6.2931260000	-1.3824460000	H	-3.4426570000	-2.0667750000	4.1466740000
H	1.7468780000	6.2267380000	-0.4238030000	H	-4.6407020000	-0.8059090000	3.8132590000
C	0.3505790000	1.6235940000	3.8921890000	C	-3.8828350000	-1.9465000000	1.3972220000
H	-0.2217430000	2.5232080000	3.6381630000	H	-3.4294610000	-1.9924620000	0.4045050000
H	1.2230190000	1.9371640000	4.4781030000	H	-4.9249820000	-1.6271520000	1.2863500000
H	-0.2750530000	0.9840810000	4.5243460000	H	-3.8871030000	-2.9483750000	1.8408550000
C	1.5380670000	-0.4326670000	3.0080440000	O	-1.9880550000	-0.6926590000	-1.4055180000
H	0.8558320000	-1.1017320000	3.5411350000	H	0.4385680000	1.0914570000	3.2760720000
H	2.3978720000	-0.2367150000	3.6606750000	H	1.7092500000	-0.9168320000	3.1661590000
H	1.8680160000	-0.9640650000	2.1125210000	C	-3.0002290000	-0.3227690000	-2.3653570000
C	-4.5514430000	0.4622060000	3.1223760000	C	-3.0985330000	1.2078870000	-2.5002340000
H	-5.1574380000	1.1781040000	2.5549350000	C	-2.7307900000	-0.9780910000	-3.7304890000
H	-4.2615790000	0.9275050000	4.0710600000	C	-4.3410130000	-0.8464050000	-1.8229760000
H	-5.1853230000	-0.4002690000	3.3570510000	H	-3.2740640000	1.6469610000	-1.5128620000
C	-2.4446870000	-0.9704080000	3.1255650000	H	-2.1673550000	1.6222290000	-2.9000370000
H	-1.5594050000	-1.2404730000	2.5374820000	H	-3.9184570000	1.5000540000	-3.1686620000
H	-3.0101430000	-1.8860690000	3.3347210000	H	-2.6534700000	-2.0628490000	-3.6053270000
H	-2.1390280000	-0.5341230000	4.0845250000	H	-3.5382250000	-0.7634220000	-4.4417720000
O	-0.3582750000	-1.7253330000	0.6688320000	H	-1.7936670000	-0.6141410000	-4.1555790000
H	-3.0427460000	2.9428570000	-0.7280730000	H	-5.1586770000	-0.6257910000	-2.5204000000
H	-1.3155200000	4.1841920000	0.3745040000	H	-4.2896720000	-1.9303780000	-1.6792360000
C	-0.2783480000	-3.1636550000	0.5780740000	H	-4.5618740000	-0.3731260000	-0.8621410000
C	-0.3938310000	-3.6367330000	-0.8823760000	C	2.7153300000	1.9899180000	-0.8756420000
C	1.0321400000	-3.6599170000	1.2098240000	C	3.9088660000	2.3302780000	-1.5348260000
C	-1.4710360000	-3.7378790000	1.3633810000	C	2.6504390000	2.1701280000	0.5135480000
H	-1.3121530000	-3.2287500000	-1.3205330000	C	4.9948030000	2.8517600000	-0.8298580000
H	0.4623500000	-3.2920450000	-1.4686220000	H	3.9700550000	2.2097790000	-2.6122330000
H	-0.4311580000	-4.7314890000	-0.9452030000	C	3.7315000000	2.6993880000	1.2185200000
H	1.0599070000	-3.3755840000	2.2669170000	H	1.7470780000	1.8934130000	1.0444580000
H	1.1267130000	-4.7510230000	1.1389120000	C	4.9060740000	3.0485900000	0.5490310000
H	1.8917690000	-3.2064290000	0.7133260000	H	5.9046910000	3.1131880000	-1.3594810000
H	-1.5146650000	-4.8300850000	1.2663510000	H	3.6554710000	2.8382740000	2.2922760000

H	-1.3810570000	-3.4918490000	2.4250490000	H	5.7441430000	3.4669530000	1.0960650000
H	-2.4025150000	-3.3075600000	0.9831030000	H	0.3385290000	-0.3319160000	-4.2187870000

**TS-outer-linear**	**TS-outer-branched**
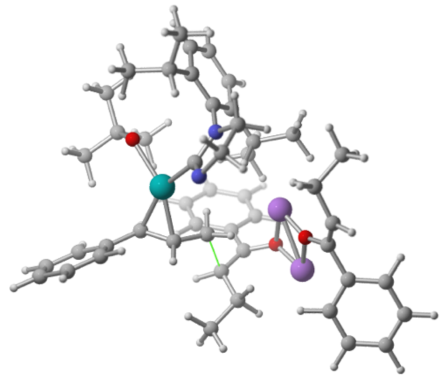	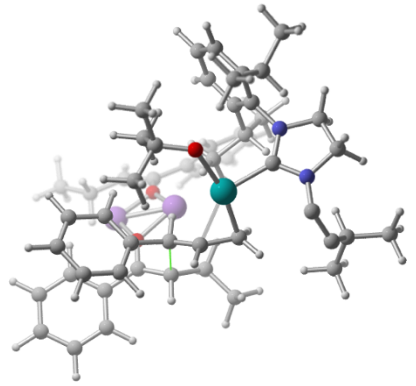
Gas phase energy= -583.654428037<br>Gas phase free energy correction= 1.174035<br>SMD energy= -2811.88184567	Gas phase energy= -583.632566904<br>Gas phase free energy correction= 1.169720<br>SMD energy= -2811.86564820

C	-1.2176440000	-1.0434640000	2.4898050000	C	1.1634660000	-1.8373130000	0.4880110000
H	-1.2465810000	-2.1352200000	2.4639220000	H	1.4299250000	-0.8529990000	0.0832190000
C	-0.0002260000	-0.3898680000	2.0463150000	C	-0.2121540000	-1.9022810000	0.9977950000
C	0.9489910000	-1.0880800000	1.2207720000	C	-0.8883670000	-0.8589570000	1.7561800000
H	0.3364310000	0.5137220000	2.5502280000	H	-0.6052100000	-2.9118240000	1.1196930000
H	0.5197970000	-1.8903090000	0.6272780000	H	-0.3446650000	0.0284170000	2.0851270000
Pd	-1.4988600000	-0.1797500000	0.5496290000	Pd	-1.7124570000	-0.6631250000	-0.0937880000
C	-1.9846040000	-0.4581490000	3.6142580000	C	1.5283500000	-2.9106410000	-0.5122320000
C	-2.6951770000	-1.2798440000	4.5064370000	C	2.1478860000	-2.5724580000	-1.7201010000
C	-2.0356860000	0.9334560000	3.8218180000	C	1.3033750000	-4.2638570000	-0.2318560000
C	-3.4339200000	-0.7356840000	5.5575390000	C	2.5154080000	-3.5563870000	-2.6376370000
H	-2.6737450000	-2.3575920000	4.3644370000	H	2.3208630000	-1.5277430000	-1.9554460000
C	-2.7665720000	1.4768420000	4.8755200000	C	1.6814440000	-5.2515520000	-1.1401420000
H	-1.5300280000	1.5970160000	3.1277100000	H	0.8255600000	-4.5490430000	0.7003310000
C	-3.4729260000	0.6470760000	5.7512580000	C	2.2866160000	-4.9014750000	-2.3492530000
H	-3.9821280000	-1.3930400000	6.2254370000	H	2.9678880000	-3.2702210000	-3.5817400000
H	-2.7948490000	2.5550670000	5.0026900000	H	1.4956880000	-6.2950060000	-0.9092430000
H	-4.0490260000	1.0714370000	6.5673240000	H	2.5672090000	-5.6695560000	-3.0617980000
H	1.6570520000	-0.4502840000	0.6844400000	H	-1.6077070000	-1.1943870000	2.5048210000
C	-0.5950290000	3.1505440000	-2.0861600000	C	-4.7882980000	2.1123950000	1.2067970000
C	-0.6013990000	2.0393430000	-3.1443830000	C	-3.9626880000	3.0302450000	0.2975570000
H	-1.4316160000	3.8505160000	-2.1964610000	H	-5.7711630000	1.8727800000	0.7822480000
H	-1.2899890000	2.2372130000	-3.9683900000	H	-4.5648730000	3.5593580000	-0.4441780000
C	-1.0680130000	1.0765320000	-1.0224420000	C	-3.0027940000	0.8612260000	0.2654900000
N	-0.7457090000	2.3830700000	-0.8333490000	N	-3.9489250000	0.8967060000	1.2453840000
N	-1.0414500000	0.8623380000	-2.3633950000	N	-3.0502850000	2.0690240000	-0.3516640000
C	-1.6276960000	-0.2585180000	-3.0342440000	C	-2.3856190000	2.3925670000	-1.5749290000
C	-0.9072600000	-1.4538580000	-3.1829690000	C	-1.0404810000	2.7909230000	-1.5577940000
C	-2.9227510000	-0.1020380000	-3.5630310000	C	-3.1258400000	2.3097290000	-2.7680250000
C	-1.4985810000	-2.4984320000	-3.9027880000	C	-0.4268350000	3.1031000000	-2.7757310000
C	-3.4763750000	-1.1684420000	-4.2758970000	C	-2.4758940000	2.6247860000	-3.9643100000
C	-2.7714930000	-2.3558150000	-4.4487850000	C	-1.1381320000	3.0162520000	-3.9703680000
H	-0.9707950000	-3.4370170000	-4.0353000000	H	0.6161390000	3.4092350000	-2.7935520000
H	-4.4793940000	-1.0756780000	-4.6804610000	H	-3.0162430000	2.5463940000	-4.9027670000
C	-0.8490810000	3.0617870000	0.4244380000	C	-4.3327970000	-0.2372340000	2.0285330000
C	-2.1263630000	3.3517300000	0.9480390000	C	-4.9646810000	-1.3297480000	1.3992040000
C	0.3306200000	3.4540860000	1.0840350000	C	-4.0798010000	-0.2213110000	3.4139700000
C	-2.1918790000	4.0713800000	2.1472490000	C	-5.3386680000	-2.4155760000	2.2002110000
C	0.2158820000	4.1655710000	2.2833440000	C	-4.4818120000	-1.3238270000	4.1753490000
C	-1.0360670000	4.4766560000	2.8091410000	C	-5.1036290000	-2.4150520000	3.5729990000
H	-3.1620530000	4.3016840000	2.5749020000	H	-5.8208150000	-3.2723230000	1.7411640000
H	1.1118400000	4.4783910000	2.8108370000	H	-4.3010910000	-1.3328250000	5.2463600000

C	-3.4149200000	2.9279670000	0.2545160000	C	-5.2792500000	-1.3461320000	-0.0923050000
H	-3.1677390000	2.2212900000	-0.5414760000	H	-4.7078300000	-0.5551100000	-0.5833830000
C	1.7043420000	3.1348140000	0.5122140000	C	-3.4009620000	0.9639510000	4.0893390000
H	1.5665520000	2.4114050000	-0.2999450000	H	-3.0275830000	1.6224590000	3.2964420000
C	-3.7354180000	1.1705640000	-3.3568530000	C	-4.5908100000	1.8917140000	-2.7692350000
H	-3.1143620000	1.8905940000	-2.8146440000	H	-4.8997670000	1.7536030000	-1.7279270000
C	0.4929000000	-1.6159220000	-2.6007510000	C	-0.2688450000	2.9013170000	-0.2510880000
H	0.5883360000	-0.8459310000	-1.8176180000	H	-0.9396690000	2.5836430000	0.5537280000
H	-3.2196210000	-3.1779860000	-4.9964220000	H	-0.6467250000	3.2462510000	-4.9099420000
H	-1.1106620000	5.0273040000	3.7410890000	H	-5.4051840000	-3.2669170000	4.1740610000
C	0.6844400000	-2.9977020000	-1.9552170000	C	0.9326940000	1.9389630000	-0.2184270000
H	1.6720010000	-3.1174060000	-1.4901950000	H	1.4199090000	2.0360520000	0.7669230000
H	0.6186570000	-3.7996730000	-2.6969260000	H	1.6594070000	2.1711240000	-1.0090270000
H	-0.0733530000	-3.1832490000	-1.1884260000	H	0.5635340000	0.9163450000	-0.3530440000
C	1.5570400000	-1.3599220000	-3.6874530000	C	0.1596360000	4.3510980000	0.0269440000
H	1.4176010000	-0.3780680000	-4.1473640000	H	-0.7079500000	5.0190660000	0.0242010000
H	1.4818700000	-2.1185340000	-4.4745180000	H	0.8604470000	4.7113280000	-0.7360870000
H	2.5871460000	-1.3971220000	-3.2978070000	H	0.6495990000	4.4240740000	1.0052090000
C	2.6287350000	2.4802600000	1.5503910000	C	-2.1884380000	0.5482530000	4.9417050000
H	2.8906050000	3.1753340000	2.3562730000	H	-2.4879510000	-0.0905860000	5.7803620000
H	3.5568630000	2.1658420000	1.0612310000	H	-1.7025450000	1.4376070000	5.3599040000
H	2.1499680000	1.6066490000	2.0019350000	H	-1.4547200000	0.0029640000	4.3422140000
C	2.3738990000	4.3960380000	-0.0658430000	C	-4.4084150000	1.7518690000	4.9510480000
H	1.7441000000	4.8768620000	-0.8219390000	H	-5.2898140000	2.0484520000	4.3724350000
H	3.3352240000	4.1374850000	-0.5242150000	H	-3.9434750000	2.6546870000	5.3634780000
H	2.5563130000	5.1319840000	0.7263800000	H	-4.7576400000	1.1373810000	5.7893830000
C	-4.1073950000	4.1468970000	-0.3851990000	C	-6.7769340000	-1.0687450000	-0.3277250000
H	-3.4415760000	4.6671710000	-1.0839660000	H	-7.0838310000	-0.1178120000	0.1237130000
H	-4.4184140000	4.8675310000	0.3807680000	H	-7.3973890000	-1.8621110000	0.1070300000
H	-5.0008320000	3.8266980000	-0.9325730000	H	-6.9830430000	-1.0227510000	-1.4029420000
C	-4.3676270000	2.1767790000	1.1982620000	C	-4.8416410000	-2.6514610000	-0.7738230000
H	-5.2416620000	1.8351390000	0.6346920000	H	-4.9666030000	-2.5536710000	-1.8563140000
H	-4.7152460000	2.8128310000	2.0217380000	H	-5.4303400000	-3.5121990000	-0.4310390000
H	-3.8833240000	1.2877930000	1.6098400000	H	-3.7791240000	-2.8356460000	-0.5943410000
C	-4.1354450000	1.8147120000	-4.6965750000	C	-5.4866280000	2.9842790000	-3.3810480000
H	-3.2618960000	2.0129430000	-5.3294000000	H	-5.3468910000	3.9498100000	-2.8800470000
H	-4.6545730000	2.7626440000	-4.5165430000	H	-6.5409330000	2.6980820000	-3.2951270000
H	-4.8146640000	1.1655010000	-5.2607140000	H	-5.2661530000	3.1251280000	-4.4454440000
C	-4.9558810000	0.9093410000	-2.4593900000	C	-4.7959910000	0.5309140000	-3.4519430000
H	-4.6178670000	0.4645980000	-1.5137020000	H	-4.1177750000	-0.2047150000	-2.9969450000
H	-5.6596590000	0.2178330000	-2.9387940000	H	-4.5763910000	0.5909460000	-4.5252730000
H	-5.4853100000	1.8512200000	-2.2679960000	H	-5.8403410000	0.2121350000	-3.3397800000
O	-3.3746020000	-0.7046160000	-0.2576550000	O	-2.3448910000	-0.8992920000	-2.1171500000
H	0.3915380000	1.8574120000	-3.5654230000	H	-3.3827270000	3.7693780000	0.8648080000
H	0.3352970000	3.7221060000	-2.0818590000	H	-4.9335430000	2.5247930000	2.2065600000
C	-3.9499770000	-1.9947620000	-0.0081560000	C	-1.4861600000	-1.4745720000	-3.1038700000
C	-2.9105420000	-3.1111220000	-0.2475810000	C	-0.1736900000	-0.6680920000	-3.1685740000
C	-4.4946690000	-2.0741790000	1.4274140000	C	-1.2169200000	-2.9545330000	-2.7706340000
C	-5.1117940000	-2.1870170000	-1.0024200000	C	-2.1778690000	-1.3885690000	-4.4788340000
H	-2.5225300000	-3.0258110000	-1.2688310000	H	-0.4067080000	0.3662660000	-3.4405990000
H	-2.0739400000	-2.9978870000	0.4527110000	H	0.2863270000	-0.6583590000	-2.1751580000
H	-3.3467740000	-4.1106660000	-0.1144600000	H	0.5426770000	-1.0836080000	-3.8912130000
H	-5.2129430000	-1.2635140000	1.5890180000	H	-2.1648710000	-3.5040110000	-2.7981910000
H	-4.9928360000	-3.0332990000	1.6232730000	H	-0.5133380000	-3.4221600000	-3.4725040000
H	-3.6814420000	-1.9454230000	2.1438080000	H	-0.8080030000	-3.0347740000	-1.7592080000
H	-5.5814420000	-3.1718100000	-0.8812450000	H	-1.5683080000	-1.8558900000	-5.2636730000
H	-5.8725800000	-1.4167390000	-0.8400830000	H	-3.1470210000	-1.8973710000	-4.4378930000
H	-4.7384160000	-2.0943550000	-2.0270240000	H	-2.3488170000	-0.3400440000	-4.7438760000
C	4.2850550000	1.9574710000	-2.0604570000	C	6.9243280000	2.5846890000	-0.4730460000
C	4.6966800000	1.0019520000	-1.2061910000	C	5.6799320000	2.7225410000	0.0377420000
O	3.9727560000	-0.0928250000	-0.8438540000	O	4.8193700000	1.6697150000	0.0661810000
C	6.0406920000	1.0628150000	-0.5484600000	C	5.1912000000	4.0107080000	0.6031550000
C	6.1692100000	0.7949480000	0.8276320000	C	3.8639100000	4.4199570000	0.4097170000
C	7.2029390000	1.3286100000	-1.2862490000	C	6.0508760000	4.8377880000	1.3432050000
C	7.4220410000	0.7912920000	1.4457740000	C	3.4170430000	5.6400200000	0.9183520000
H	5.2752980000	0.6793780000	1.4393880000	H	3.1913130000	3.7933800000	-0.1671720000

C	8.4543710000	1.3271900000	-0.6681000000	C	5.6006970000	6.0533570000	1.8587730000
H	7.1178100000	1.5190710000	-2.3508250000	H	7.0700990000	4.5149170000	1.5287820000
C	8.5709320000	1.0521310000	0.6965970000	C	4.2826170000	6.4604560000	1.6449440000
H	7.4980400000	0.6055910000	2.5120920000	H	2.3950180000	5.9560780000	0.7423200000
H	9.3430010000	1.5323630000	-1.2549300000	H	6.2758420000	6.6776280000	2.4344010000
H	9.5451990000	1.0477090000	1.1721190000	H	3.9307900000	7.4060980000	2.0430460000
H	4.9528060000	2.7965100000	-2.2476880000	H	7.5863740000	3.4435980000	-0.5283620000
C	2.9838160000	1.9474380000	-2.8078140000	C	7.4372560000	1.2747570000	-1.0136170000
C	3.1913850000	1.7738360000	-4.3226960000	C	8.8179050000	1.3905160000	-1.6707770000
H	2.3363660000	1.1495590000	-2.4201560000	H	7.5181410000	0.5203930000	-0.2010740000
H	2.4502870000	2.8922160000	-2.6275630000	H	6.7323940000	0.8719520000	-1.7700270000
H	2.2426340000	1.8082780000	-4.8713500000	H	9.1634110000	0.4267350000	-2.0578350000
H	3.6764520000	0.8153120000	-4.5364430000	H	9.5560800000	1.7524290000	-0.9477750000
H	3.8335660000	2.5690050000	-4.7173940000	H	8.7828960000	2.1013640000	-2.5022710000
C	2.2582720000	-2.0744120000	2.2359170000	C	2.3689080000	-1.9136620000	1.7743020000
C	2.6037580000	-2.9857640000	1.2151360000	C	3.6125440000	-1.9887250000	1.0499450000
O	3.4565390000	-2.5941400000	0.2871000000	O	4.0254690000	-0.8982670000	0.4760760000
C	1.8885080000	-4.2553610000	0.9949200000	C	4.3538150000	-3.2364950000	0.8160730000
C	2.4381870000	-5.2061160000	0.1186010000	C	5.1241170000	-3.3842480000	-0.3493240000
C	0.6352620000	-4.5077480000	1.5800060000	C	4.3107510000	-4.2907730000	1.7437830000
C	1.7530880000	-6.3851780000	-0.1607800000	C	5.8231240000	-4.5626510000	-0.5899450000
H	3.3993950000	-5.0029440000	-0.3385030000	H	5.1136650000	-2.5993850000	-1.0979100000
C	-0.0569870000	-5.6791400000	1.2813870000	C	5.0224670000	-5.4641270000	1.5077460000
H	0.1787680000	-3.7794640000	2.2404030000	H	3.7344520000	-4.1883420000	2.6559550000
C	0.5000670000	-6.6202110000	0.4132980000	C	5.7752120000	-5.6044660000	0.3397710000
H	2.1878130000	-7.1167080000	-0.8329870000	H	6.3864760000	-4.6791370000	-1.5087950000
H	-1.0362630000	-5.8466470000	1.7141560000	H	4.9874010000	-6.2698650000	2.2319120000
H	-0.0414700000	-7.5300680000	0.1809430000	H	6.3157480000	-6.5248280000	0.1510170000
H	1.6619700000	-2.4701010000	3.0547440000	H	2.1160780000	-2.8297190000	2.3083280000
C	3.2968850000	-1.0452040000	2.6658840000	C	2.2410160000	-0.6623920000	2.6627830000
C	2.9883290000	-0.4228420000	4.0308800000	C	1.5698990000	-0.9636790000	4.0055540000
H	4.2918520000	-1.5240200000	2.7145250000	H	3.2400280000	-0.2397660000	2.8511650000
H	3.3602770000	-0.2396090000	1.9193200000	H	1.6573730000	0.1125650000	2.1449600000
H	3.7148580000	0.3575370000	4.2794250000	H	1.4043080000	-0.0446430000	4.5771260000
H	3.0182690000	-1.1829570000	4.8182450000	H	2.1957080000	-1.6323320000	4.6073280000
H	1.9909480000	0.0262900000	4.0394050000	H	0.6009650000	-1.4443150000	3.8459220000
Li	2.7218280000	-1.3108300000	-1.0508390000	Li	5.4068750000	-0.0050880000	-0.2466510000
Li	4.6906250000	-1.2489020000	0.3654670000	Li	3.3065420000	0.8894970000	0.5794170000

TS-outer-linear-2

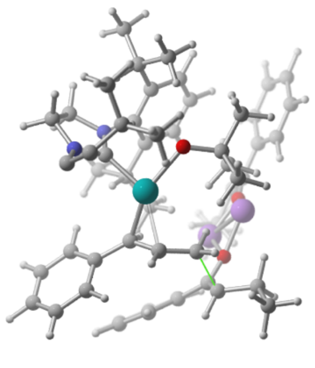

Gas phase energy= -583.645766498
Gas phase free energy correction= 1.174114
SMD energy= -2811.86963039

TS-outer-branched-2

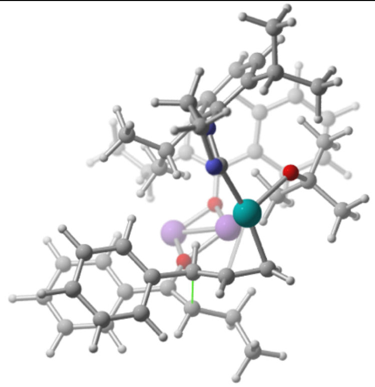

Gas phase energy= -583.638427798
Gas phase free energy correction= 1.169966
SMD energy= -2811.86429685

C	0.2285990000	1.6731940000	2.6466320000	C	0.9234430000	0.5059570000	-2.8417600000
H	1.0217570000	1.0945920000	2.1880360000	H	-0.0371800000	0.2316150000	-3.2643720000
C	-0.9918420000	1.8209160000	1.9125790000	C	1.0650950000	1.6726370000	-2.0015550000
C	-1.0921400000	1.8903820000	0.4672530000	C	-0.0322960000	2.2809350000	-1.2416910000
H	-1.8650140000	2.0950150000	2.5027450000	H	1.9542900000	2.2912170000	-2.1198920000
H	-0.1230680000	1.9142270000	-0.0421580000	H	-0.6815410000	1.5389670000	-0.7706680000

Pd	-1.2152740000	-0.1512500000	0.8814810000	Pd	1.3397880000	-0.2993380000	-0.9883730000
C	-3.4798650000	-2.0363220000	-2.3191680000	C	3.9581520000	-1.0047090000	2.4231920000
C	-2.0757720000	-2.0509570000	-2.9269370000	C	2.6456440000	-1.3906620000	3.1091840000
H	-3.8379030000	-3.0360960000	-2.0474120000	H	4.6054870000	-1.8672240000	2.2227570000
H	-1.7782540000	-3.0284730000	-3.3079080000	H	2.6716900000	-2.3792450000	3.5690000000
C	-1.9593770000	-1.0774960000	-0.7744100000	C	2.1589810000	-0.6926660000	0.8903160000
N	-3.2739740000	-1.2276440000	-1.0949910000	N	3.4707020000	-0.4340860000	1.1520560000
N	-1.2410310000	-1.6515780000	-1.7760960000	N	1.6789660000	-1.3482180000	1.9864420000
C	0.1245190000	-2.0794030000	-1.6755010000	C	0.5003110000	-2.1660700000	2.0389370000
C	1.1729910000	-1.1754460000	-1.9174600000	C	-0.7718950000	-1.5833670000	2.1726620000
C	0.3664690000	-3.4307600000	-1.3635680000	C	0.6684630000	-3.5618560000	1.9844370000
C	2.4879200000	-1.6469360000	-1.8400100000	C	-1.8814370000	-2.4280760000	2.2908940000
C	1.6954740000	-3.8595700000	-1.2908360000	C	-0.4613580000	-4.3713980000	2.1407960000
C	2.7473050000	-2.9794600000	-1.5255160000	C	-1.7247020000	-3.8126200000	2.2954230000
H	3.3214790000	-0.9751610000	-2.0194020000	H	-2.8791890000	-2.0085300000	2.3688920000
H	1.9109140000	-4.8950280000	-1.0446550000	H	-0.3534950000	-5.4508460000	2.0966990000
C	-4.3763550000	-0.9646600000	-0.2105700000	C	4.4081410000	0.0392840000	0.1777980000
C	-4.4992880000	-1.7037870000	0.9886650000	C	4.8081230000	-0.8156150000	-0.8719620000
C	-5.3549750000	-0.0262560000	-0.5965120000	C	4.9473820000	1.3283350000	0.3294360000
C	-5.5932100000	-1.4277250000	1.8178840000	C	5.7541520000	-0.3310790000	-1.7823640000
C	-6.4416830000	0.1959460000	0.2571320000	C	5.8980220000	1.7673370000	-0.5988360000
C	-6.5537110000	-0.4857730000	1.4634280000	C	6.2950910000	0.9460750000	-1.6504750000
H	-5.7004080000	-1.9705640000	2.7500810000	H	6.0753710000	-0.9649600000	-2.6024510000
H	-7.2034360000	0.9157170000	-0.0238490000	H	6.3275930000	2.7599270000	-0.4989700000
C	-3.5619240000	-2.8469140000	1.3612030000	C	4.2913490000	-2.2434370000	-1.0089990000
H	-2.5915290000	-2.7051180000	0.8800130000	H	3.3985510000	-2.3559420000	-0.3889540000
C	-5.2951240000	0.7200530000	-1.9219290000	C	4.5212460000	2.2388980000	1.4706060000
H	-4.2801180000	0.6117190000	-2.3231120000	H	3.6987240000	1.7434650000	1.9984830000
C	-0.7598860000	-4.4352140000	-1.1488420000	C	2.0164230000	-4.2134020000	1.6994510000
H	-1.7135070000	-3.9004030000	-1.1999430000	H	2.7739770000	-3.4264530000	1.6318720000
C	0.8741300000	0.2664760000	-2.2968080000	C	-0.9227670000	-0.0698050000	2.1826210000
H	-0.1286130000	0.4910940000	-1.9244300000	H	-0.2250340000	0.3162800000	1.4279950000
H	3.7710450000	-3.3292630000	-1.4594270000	H	-2.5934950000	-4.4557260000	2.3887630000
H	-7.3943490000	-0.2928800000	2.1220160000	H	7.0296630000	1.2990730000	-2.3671820000
C	1.8340680000	1.2662780000	-1.6362580000	C	-2.3342860000	0.3930180000	1.8043800000
H	1.5000920000	2.2952860000	-1.8180820000	H	-2.3357590000	1.4765990000	1.6444950000
H	2.8492960000	1.1498820000	-2.0404730000	H	-3.0576800000	0.1708600000	2.5984210000
H	1.8524900000	1.0885990000	-0.5540010000	H	-2.6941960000	-0.0950790000	0.8935130000
C	0.8725210000	0.4467990000	-3.8257570000	C	-0.5363270000	0.5212040000	3.5520070000
H	0.1567580000	-0.2344590000	-4.2973020000	H	0.5073970000	0.3092190000	3.7947790000
H	1.8641890000	0.2322860000	-4.2421680000	H	-1.1681320000	0.0961800000	4.3409390000
H	0.6000760000	1.4747830000	-4.0941310000	H	-0.6696260000	1.6102360000	3.5594310000
C	-5.5558980000	2.2230560000	-1.7491950000	C	3.9739670000	3.5737880000	0.9462320000
H	-6.6010010000	2.4222370000	-1.4851090000	H	4.7509620000	4.1565090000	0.4371590000
H	-5.3439870000	2.7543100000	-2.6824160000	H	3.5847490000	4.1801270000	1.7710510000
H	-4.9176750000	2.6384460000	-0.9676810000	H	3.1584840000	3.3978970000	0.2402380000
C	-6.3072370000	0.1320280000	-2.9284970000	C	5.6752550000	2.4665390000	2.4649010000
H	-6.1890650000	-0.9487460000	-3.0576430000	H	6.0659250000	1.5189190000	2.8523390000
H	-6.1991850000	0.6136640000	-3.9071380000	H	5.3377770000	3.0746180000	3.3120380000
H	-7.3308370000	0.3062880000	-2.5762030000	H	6.5063190000	2.9931900000	1.9807720000
C	-4.1684510000	-4.1804530000	0.8783800000	C	5.3545880000	-3.2430130000	-0.5118020000
H	-4.3085570000	-4.1912320000	-0.2088020000	H	5.6354640000	-3.0472700000	0.5294970000
H	-5.1471450000	-4.3523440000	1.3434020000	H	6.2645880000	-3.1813560000	-1.1211160000
H	-3.5095360000	-5.0122180000	1.1474630000	H	4.9679230000	-4.2658810000	-0.5775840000
C	-3.2521370000	-2.9096590000	2.8635390000	C	3.8475860000	-2.5823750000	-2.4402400000
H	-2.4533670000	-3.6381590000	3.0290270000	H	3.3671000000	-3.5655740000	-2.4465920000
H	-4.1240280000	-3.2104730000	3.4587260000	H	4.6935300000	-2.6062220000	-3.1389760000
H	-2.8825760000	-1.9432470000	3.2160920000	H	3.1046130000	-1.8602040000	-2.7889790000
C	-0.7518660000	-5.5086920000	-2.2552290000	C	2.4388250000	-5.1838360000	2.8168880000
H	-0.7850100000	-5.0637620000	-3.2565720000	H	2.4471730000	-4.6960010000	3.7988150000
H	-1.6138090000	-6.1761340000	-2.1436080000	H	3.4422140000	-5.5763390000	2.6170610000
H	0.1562540000	-6.1197900000	-2.1964960000	H	1.7540090000	-6.0375710000	2.8763750000
C	-0.7142010000	-5.0761140000	0.2444590000	C	2.0023770000	-4.9016310000	0.3235710000
H	-0.8209790000	-4.2939610000	1.0018300000	H	1.7397830000	-4.1621210000	-0.4436070000
H	0.2306650000	-5.6057060000	0.4135270000	H	1.2645320000	-5.7128890000	0.2960870000
H	-1.5256870000	-5.8060690000	0.3456020000	H	2.9877060000	-5.3345660000	0.1111150000

O	-0.5058180000	-2.0616860000	1.6562190000	O	0.7483210000	-2.3520920000	-1.1707260000
H	-1.9692910000	-1.3160630000	-3.7334260000	H	2.3562790000	-0.6655780000	3.8780240000
H	-4.2125790000	-1.5735000000	-2.9777340000	H	4.5315750000	-0.2663840000	2.9876970000
C	0.6161480000	-2.1321530000	2.5324480000	C	-0.3909520000	-2.7087200000	-1.9493530000
C	1.8401430000	-1.4269840000	1.8791980000	C	-1.5521160000	-1.7060590000	-1.6997150000
C	0.2617340000	-1.5219310000	3.9044880000	C	-0.0371030000	-2.7610950000	-3.4487780000
C	1.0182400000	-3.6043370000	2.7589100000	C	-0.8824090000	-4.0989060000	-1.5026050000
H	2.2271230000	-2.0319160000	1.0539590000	H	-1.8402790000	-1.7714510000	-0.6419440000
H	1.4974910000	-0.4805210000	1.4427570000	H	-1.1516240000	-0.7043130000	-1.9139500000
H	2.6484130000	-1.2737340000	2.6192560000	H	-2.4248720000	-1.9362990000	-2.3279740000
H	-0.4457190000	-2.1842760000	4.4143100000	H	0.7563930000	-3.5019070000	-3.5922980000
H	1.1396080000	-1.3877720000	4.5524130000	H	-0.9003120000	-3.0481990000	-4.0654400000
H	-0.2384670000	-0.5630130000	3.7624660000	H	0.3434720000	-1.7966950000	-3.7922680000
H	1.8325840000	-3.6830110000	3.4924340000	H	-1.8019410000	-4.3870420000	-2.0276280000
H	0.1580710000	-4.1677180000	3.1341860000	H	-0.1123260000	-4.8485750000	-1.7077130000
H	1.3495280000	-4.0574010000	1.8206080000	H	-1.0795440000	-4.0894610000	-0.4275590000
C	-2.0203600000	2.8194800000	-0.2341880000	C	0.3768570000	3.3892310000	-0.3081430000
C	-1.9506150000	2.9265500000	-1.6365520000	C	0.0913690000	3.3293850000	1.0580320000
C	-2.8434760000	3.7359610000	0.4434800000	C	1.0021850000	4.5401350000	-0.8100760000
C	-2.6211740000	3.9357730000	-2.3241660000	C	0.3938020000	4.3992800000	1.9019550000
H	-1.3316660000	2.2230210000	-2.1863450000	H	-0.3712180000	2.4379630000	1.4617060000
C	-3.5206360000	4.7468120000	-0.2442830000	C	1.3041320000	5.6128460000	0.0277440000
H	-2.9273310000	3.6854160000	1.5239870000	H	1.2470450000	4.5974430000	-1.8672980000
C	-3.4007640000	4.8661810000	-1.6302160000	C	0.9919820000	5.5500130000	1.3877510000
H	-2.5291040000	4.0024910000	-3.4039420000	H	0.1595860000	4.3310810000	2.9592550000
H	-4.1379670000	5.4469980000	0.3097080000	H	1.7861960000	6.4953170000	-0.3798220000
H	-3.9190870000	5.6576020000	-2.1616210000	H	1.2224200000	6.3851780000	2.0405910000
H	0.0762930000	1.3956130000	3.6850490000	H	1.7354190000	0.3206630000	-3.5472580000
C	6.1011690000	0.6389530000	-1.5135110000	C	-5.8297410000	-0.9293730000	1.1277410000
C	5.4784240000	-0.0915130000	-0.5585380000	C	-5.1580530000	-1.2268810000	-0.0081080000
O	4.5673820000	0.4904000000	0.2560170000	O	-4.6180880000	-0.2347640000	-0.7631810000
C	5.7283300000	-1.5457210000	-0.3395980000	C	-4.9638330000	-2.6129710000	-0.5110640000
C	5.3426490000	-2.1448000000	0.8663380000	C	-4.9490160000	-2.8547830000	-1.8922330000
C	6.3085480000	-2.3575440000	-1.3285610000	C	-4.7765800000	-3.6906410000	0.3652240000
C	5.5240560000	-3.5101380000	1.0846690000	C	-4.7657670000	-4.1457120000	-2.3852500000
H	4.8892220000	-1.5356220000	1.6384670000	H	-5.0885950000	-2.0218530000	-2.5732800000
C	6.5013430000	-3.7210880000	-1.1098310000	C	-4.5865020000	-4.9815710000	-0.1277810000
H	6.5839490000	-1.9305000000	-2.2865780000	H	-4.7550390000	-3.5103840000	1.4351850000
C	6.1060860000	-4.3055220000	0.0965540000	C	-4.5821950000	-5.2139530000	-1.5039390000
H	5.2040530000	-3.9523620000	2.0221120000	H	-4.7583940000	-4.3187210000	-3.4563810000
H	6.9452360000	-4.3318540000	-1.8888110000	H	-4.4252670000	-5.8030130000	0.5623920000
H	6.2452590000	-5.3684150000	0.2608100000	H	-4.4236940000	-6.2157040000	-1.8879290000
H	6.8603000000	0.1910630000	-2.1468290000	H	-6.3048240000	-1.7156420000	1.7070360000
C	5.8351450000	2.1120510000	-1.6964180000	C	-6.0080100000	0.4944640000	1.5877500000
C	6.8159260000	2.7859760000	-2.6635460000	C	-6.7363050000	0.6156660000	2.9309380000
H	4.8115940000	2.2883290000	-2.0861620000	H	-5.0212850000	0.9880170000	1.6930090000
H	5.9013770000	2.6266390000	-0.7177500000	H	-6.5792430000	1.0729680000	0.8312350000
H	6.6046770000	3.8547900000	-2.7719680000	H	-6.8594630000	1.6616300000	3.2303080000
H	6.7549270000	2.3248140000	-3.6549590000	H	-6.1756570000	0.0981370000	3.7164730000
H	7.8430390000	2.6751880000	-2.3013390000	H	-7.7296690000	0.1595350000	2.8698500000
C	1.3768820000	3.1442590000	3.0194170000	C	-1.3786730000	3.1115240000	-2.2738980000
C	2.0923590000	3.2440320000	1.8055040000	C	-2.4517750000	3.1329310000	-1.3314810000
O	3.0047490000	2.3485430000	1.5174340000	O	-3.1655810000	2.0514390000	-1.1855080000
C	1.7769190000	4.2174090000	0.7413560000	C	-2.7045610000	4.2466320000	-0.3949140000
C	2.7719800000	4.5744540000	-0.1899880000	C	-3.4113570000	4.0082440000	0.7979430000
C	0.4864820000	4.7410400000	0.5909640000	C	-2.2393260000	5.5469200000	-0.6492930000
C	2.4682110000	5.4011960000	-1.2682070000	C	-3.6313460000	5.0291940000	1.7163920000
H	3.8056270000	4.2718220000	-0.0254700000	H	-3.7355190000	3.0020240000	1.0438020000
C	0.1774530000	5.5464750000	-0.5047970000	C	-2.4570540000	6.5688850000	0.2712480000
H	-0.2940540000	4.4722750000	1.2912240000	H	-1.7034520000	5.7633030000	-1.5644090000
C	1.1620130000	5.8721740000	-1.4384460000	C	-3.1496630000	6.3144520000	1.4559230000
H	3.2453760000	5.6842080000	-1.9700150000	H	-4.1592470000	4.8216590000	2.6406240000
H	-0.8405080000	5.8948150000	-0.6366840000	H	-2.0790630000	7.5635250000	0.0660740000
H	0.9162410000	6.4963190000	-2.2899440000	H	-3.3097890000	7.1102280000	2.1742140000
H	0.7228870000	3.9845980000	3.2481540000	H	-0.9322890000	4.0834020000	-2.4749390000
C	2.1499160000	2.6061890000	4.2272040000	C	-1.6640910000	2.3311390000	-3.5581430000

C	1.4279130000	2.8769790000	5.5506640000	C	-0.7247330000	2.7266750000	-4.6993140000
H	2.3153590000	1.5237690000	4.1143840000	H	-1.6021290000	1.2521650000	-3.3819630000
H	3.1482400000	3.0616740000	4.2558650000	H	-2.7030210000	2.5235090000	-3.8618580000
H	1.9933410000	2.4681790000	6.3937340000	H	-0.9224960000	2.1258970000	-5.5927020000
H	0.4310730000	2.4222150000	5.5684090000	H	0.3204380000	2.5761840000	-4.4169940000
H	1.3067040000	3.9533940000	5.7139930000	H	-0.8664420000	3.7817750000	-4.9626790000
Li	3.8079800000	2.0748630000	-0.1310600000	Li	-4.7245600000	1.5013290000	-0.4267700000
Li	3.4314700000	0.5168410000	1.6123860000	Li	-2.9947150000	0.1425710000	-1.4542440000

**P-linear**	**P-branched**
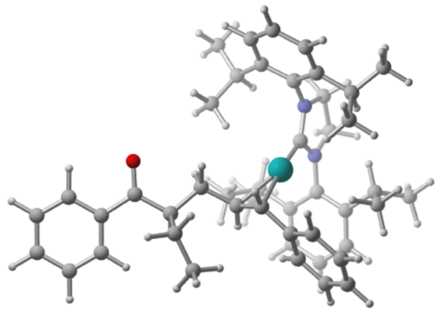	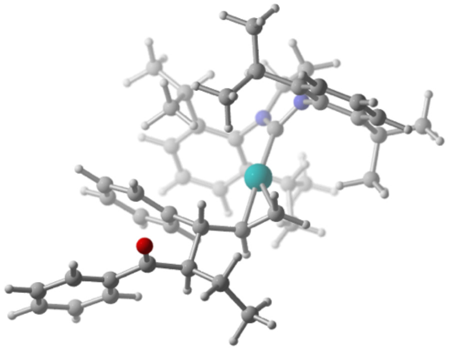
Gas phase energy= -459.582373061	Gas phase energy= -459.578045666
Gas phase free energy correction= 0.865819	Gas phase free energy correction= 0.866005
SMD energy= -2100.68731150	SMD energy= -2100.67986638

Pd	-0.6054740000	-0.0937510000	-0.5620920000	Pd	0.6727000000	-0.4335530000	-0.5337820000
C	-3.6044590000	0.8896470000	2.4379220000	C	3.9675340000	2.1548650000	0.7378080000
C	-3.6676360000	2.1857120000	1.6029750000	C	4.4834320000	0.7842300000	1.2099370000
H	-4.5441180000	0.3282100000	2.4235500000	H	4.5465370000	2.5566230000	-0.1027630000
H	-4.6293900000	2.3196150000	1.0965130000	H	5.4754830000	0.5418130000	0.8216970000
C	-1.9827360000	0.7724840000	0.6995910000	C	2.3581550000	0.4975800000	0.1910870000
N	-2.5444930000	0.1271950000	1.7506880000	N	2.5984200000	1.8269580000	0.2919140000
N	-2.5978050000	1.9729330000	0.6060890000	N	3.4643730000	-0.1311270000	0.6570990000
C	-2.3278520000	2.9391150000	-0.4148450000	C	3.5729320000	-1.5518430000	0.7906330000
C	-1.4105410000	3.9717290000	-0.1465660000	C	2.9790690000	-2.1861880000	1.8956040000
C	-2.9766900000	2.8177670000	-1.6559660000	C	4.2536270000	-2.2706510000	-0.2093760000
C	-1.1628370000	4.9081840000	-1.1550850000	C	3.0895770000	-3.5787070000	1.9895690000
C	-2.6987570000	3.7747570000	-2.6388750000	C	4.3400490000	-3.6600080000	-0.0774140000
C	-1.8045410000	4.8127220000	-2.3890670000	C	3.7644490000	-4.3084750000	1.0147420000
H	-0.4504710000	5.7084000000	-0.9849400000	H	2.6349800000	-4.0964060000	2.8288590000
H	-3.1748320000	3.7001470000	-3.6116500000	H	4.8511950000	-4.2440960000	-0.8354910000
C	-2.2024700000	-1.2127930000	2.1199620000	C	1.6646830000	2.8390430000	-0.0941360000
C	-2.8796460000	-2.2798060000	1.5032500000	C	1.5630390000	3.2083430000	-1.4459110000
C	-1.1731760000	-1.4206090000	3.0549490000	C	0.8641550000	3.4239420000	0.9064160000
C	-2.4983610000	-3.5827550000	1.8398420000	C	0.6340190000	4.1995350000	-1.7867310000
C	-0.8174100000	-2.7393340000	3.3582860000	C	-0.0457300000	4.4128400000	0.5241920000
C	-1.4740870000	-3.8114210000	2.7556140000	C	-0.1570700000	4.7995700000	-0.8111080000
H	-2.9932820000	-4.4249130000	1.3660130000	H	0.5285670000	4.5010940000	-2.8246270000
H	-0.0174690000	-2.9311680000	4.0668650000	H	-0.6870850000	4.8729760000	1.2678010000
C	-3.9793620000	-2.0446330000	0.4773370000	C	2.4073580000	2.5459080000	-2.5256300000
H	-4.1175050000	-0.9619180000	0.3769850000	H	3.0888110000	1.8410730000	-2.0366050000
C	-0.4240400000	-0.2515740000	3.6798190000	C	0.9403440000	2.9273170000	2.3441500000
H	-0.9822800000	0.6630930000	3.4488380000	H	1.9352450000	2.4915480000	2.4952040000
C	-3.9042560000	1.6490110000	-1.9610730000	C	4.8068770000	-1.5607960000	-1.4382030000
H	-4.0989650000	1.1182330000	-1.0220010000	H	4.9597930000	-0.5075590000	-1.1738770000
C	-0.6391310000	4.0058100000	1.1654220000	C	2.2076240000	-1.4112180000	2.9557610000
H	-1.2103970000	3.4303520000	1.9039880000	H	2.2485080000	-0.3470570000	2.6972450000
H	-1.1844990000	-4.8285290000	2.9993020000	H	-0.8706420000	5.5675820000	-1.0919480000
C	0.7155280000	3.2915810000	0.9893150000	C	0.7218290000	-1.8126450000	2.9634540000
H	1.2494690000	3.2355600000	1.9451240000	H	0.1700890000	-1.2194190000	3.7010540000
H	1.3494880000	3.8264330000	0.2727340000	H	0.5970780000	-2.8721840000	3.2163360000

H	0.5624680000	2.2745430000	0.6117050000	H	0.2772320000	-1.6344420000	1.9774590000
C	-0.4557870000	5.4245640000	1.7261130000	C	2.8398030000	-1.5790090000	4.3494760000
H	-1.4147660000	5.9460480000	1.8161710000	H	3.8948260000	-1.2833650000	4.3447670000
H	0.2032550000	6.0279370000	1.0923630000	H	2.7836140000	-2.6197810000	4.6885220000
H	0.0055990000	5.3746970000	2.7183900000	H	2.3093090000	-0.9600920000	5.0817390000
C	0.9685360000	-0.0959800000	3.0411990000	C	-0.0879880000	1.8025190000	2.5698990000
H	1.5779660000	-0.9914160000	3.2155500000	H	-1.1064200000	2.1861970000	2.4533990000
H	1.4933170000	0.7656880000	3.4692680000	H	0.0139110000	1.3874830000	3.5797870000
H	0.8692110000	0.0545620000	1.9614930000	H	0.0425980000	0.9895860000	1.8461130000
C	-0.3303790000	-0.3692420000	5.2104750000	C	0.7657020000	4.0460000000	3.3830270000
H	-1.3205840000	-0.4979340000	5.6604940000	H	1.4632880000	4.8715260000	3.2056050000
H	0.1255410000	0.5343030000	5.6299530000	H	0.9471310000	3.6498440000	4.3880270000
H	0.2905420000	-1.2207200000	5.5110590000	H	-0.2528670000	4.4494200000	3.3728150000
C	-5.3178210000	-2.6459430000	0.9440690000	C	3.2619200000	3.5733710000	-3.2893420000
H	-5.6142230000	-2.2463240000	1.9203040000	H	3.8858590000	4.1579360000	-2.6046150000
H	-5.2510550000	-3.7362390000	1.0337940000	H	2.6340210000	4.2727120000	-3.8531090000
H	-6.1088330000	-2.4175810000	0.2210390000	H	3.9167540000	3.0630050000	-4.0043880000
C	-3.5801840000	-2.5769410000	-0.9095190000	C	1.5281650000	1.7249060000	-3.4860020000
H	-4.3652030000	-2.3527300000	-1.6409770000	H	2.1511680000	1.2109360000	-4.2268030000
H	-3.4295110000	-3.6618660000	-0.8945480000	H	0.8208320000	2.3690570000	-4.0222650000
H	-2.6460580000	-2.1172320000	-1.2489350000	H	0.9620940000	0.9686390000	-2.9282000000
C	-5.2607870000	2.1097980000	-2.5215630000	C	6.1601400000	-2.1228650000	-1.9024250000
H	-5.7415380000	2.8301930000	-1.8510420000	H	6.8829680000	-2.1566300000	-1.0804420000
H	-5.9296460000	1.2505630000	-2.6432110000	H	6.5715960000	-1.4929700000	-2.6986320000
H	-5.1496460000	2.5837440000	-3.5031050000	H	6.0582670000	-3.1351290000	-2.3091850000
C	-3.2164330000	0.6508510000	-2.9111600000	C	3.7726300000	-1.5913070000	-2.5811420000
H	-2.2726550000	0.2887070000	-2.4784900000	H	2.8276320000	-1.1364890000	-2.2614510000
H	-2.9885690000	1.1244370000	-3.8735390000	H	3.5685800000	-2.6259640000	-2.8828100000
H	-3.8622150000	-0.2148210000	-3.0952140000	H	4.1500380000	-1.0463180000	-3.4545710000
H	-3.4657740000	3.0844770000	2.1931140000	H	4.5094300000	0.6960220000	2.3031090000
H	-3.3305720000	1.0694360000	3.4826060000	H	3.9486700000	2.9052210000	1.5317170000
C	2.4854320000	0.2898600000	-0.5661820000	C	-3.4760820000	2.4808820000	1.0368440000
H	2.3350670000	0.9692100000	-1.4118100000	C	-2.8549110000	2.3590290000	-0.2072960000
C	1.4548490000	-0.8140300000	-0.5808550000	C	-2.4120010000	1.1131680000	-0.6545670000
C	0.7993460000	-1.2275110000	-1.7445740000	C	-2.5812490000	-0.0323810000	0.1350480000
H	1.5314250000	-1.5074530000	0.2583570000	C	-3.1626760000	0.1107290000	1.4025190000
H	1.0009180000	-0.6803730000	-2.6675990000	C	-3.6194490000	1.3521760000	1.8454240000
C	3.9301480000	-0.2555500000	-0.5968900000	H	-3.8337490000	3.4470670000	1.3777900000
H	4.0578230000	-0.9172490000	0.2726470000	H	-2.7044830000	3.2342060000	-0.8298140000
C	4.2391430000	-1.0815780000	-1.8814160000	H	-1.9229130000	1.0395640000	-1.6201630000
H	5.2682660000	-0.8920650000	-2.2131100000	H	-3.2830260000	-0.7640710000	2.0350380000
H	3.5868920000	-0.7171870000	-2.6844830000	H	-4.0872080000	1.4364420000	2.8206140000
C	4.0484830000	-2.5921640000	-1.7045410000	C	-2.2344130000	-1.4251680000	-0.3836000000
H	4.2480830000	-3.1190690000	-2.6432980000	H	-1.9917000000	-2.0587870000	0.4813620000
H	4.7355710000	-2.9903620000	-0.9475360000	C	-1.0524940000	-1.4459950000	-1.3361660000
H	3.0275050000	-2.8353540000	-1.3973200000	C	-0.0011210000	-2.3573690000	-1.2146470000
C	4.9288670000	0.8831400000	-0.4649770000	H	-1.2362520000	-1.0015930000	-2.3169850000
O	4.5943560000	2.0564650000	-0.7005420000	H	0.6152720000	-2.6186420000	-2.0714270000
C	6.3427400000	0.5813770000	-0.0728650000	H	0.0361820000	-3.0634250000	-0.3870000000
C	6.7803280000	-0.7102590000	0.2560550000	C	-3.4956190000	-2.0783870000	-1.0687350000
C	7.2607730000	1.6418490000	-0.0390130000	H	-3.7644670000	-1.4432270000	-1.9208230000
C	8.1104000000	-0.9358740000	0.6104850000	C	-3.2045840000	-3.5086440000	-1.5955470000
H	6.0920670000	-1.5471520000	0.2361060000	H	-4.0643100000	-4.1532380000	-1.3827400000
C	8.5882770000	1.4168940000	0.3158520000	H	-2.3670780000	-3.9357500000	-1.0327920000
H	6.9107020000	2.6347850000	-0.2947540000	C	-2.9032990000	-3.5537870000	-3.0973660000
C	9.0156280000	0.1264410000	0.6406210000	H	-2.7271020000	-4.5845050000	-3.4234330000
H	8.4388110000	-1.9385470000	0.8613060000	H	-3.7463890000	-3.1589750000	-3.6770210000
H	9.2900570000	2.2432890000	0.3383360000	H	-2.0138170000	-2.9679280000	-3.3473930000
H	10.0500710000	-0.0502260000	0.9141900000	C	-4.6395620000	-2.1161160000	-0.0678950000
H	-1.5965010000	5.5443780000	-3.1626160000	O	-4.6207440000	-2.9797900000	0.8301720000
C	0.1556480000	-2.5496350000	-1.9335180000	C	-5.7657950000	-1.1382860000	-0.1268440000
C	-0.3603870000	-2.8828700000	-3.1979330000	C	-5.7812140000	-0.0346670000	-0.9917760000
C	0.0152710000	-3.4892830000	-0.8941910000	C	-6.8317250000	-1.3167270000	0.7693640000
C	-0.9890620000	-4.1081100000	-3.4207760000	C	-6.8373150000	0.8752880000	-0.9541310000
H	-0.2716770000	-2.1646220000	-4.0087160000	H	-4.9609230000	0.1453850000	-1.6747030000
C	-0.6054370000	-4.7151670000	-1.1201700000	C	-7.8905010000	-0.4144010000	0.7996440000

H	0.3633850000	-3.2510560000	0.1054160000	H	-6.8053480000	-2.1674040000	1.4395490000
C	-1.1110780000	-5.0348540000	-2.3835990000	C	-7.8933310000	0.6866870000	-0.0615640000
H	-1.3825280000	-4.3388140000	-4.4055530000	H	-6.8299860000	1.7337920000	-1.6165070000
H	-0.7040550000	-5.4203220000	-0.3008220000	H	-8.7084240000	-0.5628650000	1.4962790000
H	-1.5960310000	-5.9902110000	-2.5551990000	H	-8.7132410000	1.3962110000	-0.0340800000
H	2.3700410000	0.9001040000	0.3364450000	H	3.8381720000	-5.3874120000	1.1020290000

IM1	IM2
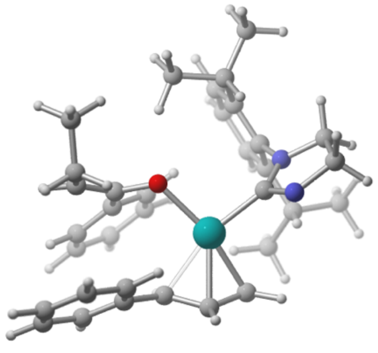	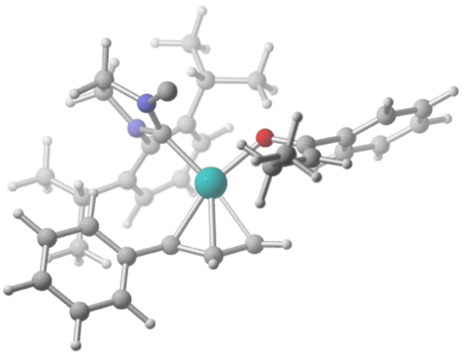
Gas phase energy= -459.571943541<br>Gas phase free energy correction= 0.871378<br>SMD energy= -2100.66593936	Gas phase energy= -459.565913387<br>Gas phase free energy correction= 0.873628<br>SMD energy= -2100.65813069

C	3.4202840000	-0.6588630000	-1.7850530000	C	2.8383160000	2.0483440000	-1.6394500000
C	2.6089810000	0.0941910000	-1.0104280000	C	2.9495190000	0.7670200000	-1.2172830000
C	1.8008950000	-1.1040420000	2.0171070000	C	0.4281570000	-0.1350930000	-3.2034190000
H	2.2929760000	-0.1918940000	2.3575300000	H	0.5502380000	-1.2148540000	-3.2658600000
C	0.5218240000	-1.4086540000	2.5249330000	C	-0.8322870000	0.4361410000	-3.0075610000
C	-0.3817970000	-0.3451640000	2.7976470000	C	-1.8053930000	-0.2285900000	-2.1957630000
H	0.1522680000	-2.4296200000	2.4601420000	H	-0.9779090000	1.4917740000	-3.2242610000
H	0.0083010000	0.5879310000	3.2012800000	H	-1.8393950000	-1.3210340000	-2.2434110000
Pd	0.1780080000	-0.2103350000	0.7421960000	Pd	0.0169480000	-0.0274790000	-1.0436280000
C	2.6816850000	-2.1404930000	1.4350000000	C	-1.5495630000	0.2709390000	2.9910310000
C	4.0548110000	-2.1349270000	1.7174230000	C	-1.6609160000	-1.2461930000	2.7822680000
C	2.1721640000	-3.1268060000	0.5749440000	H	-1.0599740000	0.5522160000	3.9235200000
C	4.8992050000	-3.0953410000	1.1610710000	H	-0.9938820000	-1.8137610000	3.4416980000
H	4.4577410000	-1.3669940000	2.3708930000	C	-0.6628070000	-0.2605140000	0.8730790000
C	3.0148780000	-4.0901380000	0.0250260000	N	-0.7354100000	0.6884820000	1.8275920000
H	1.1217790000	-3.1041980000	0.2959090000	N	-1.2264430000	-1.3969610000	1.3758410000
C	4.3814340000	-4.0773920000	0.3139640000	C	-1.0151230000	-2.7013980000	0.7998630000
H	5.9609430000	-3.0754410000	1.3846760000	C	-2.0710310000	-3.3739940000	0.1537160000
H	2.6098860000	-4.8339470000	-0.6531480000	C	0.2583610000	-3.2947500000	0.9363620000
H	5.0393130000	-4.8194630000	-0.1260820000	C	-1.7901330000	-4.5875610000	-0.4843290000
H	-1.3964060000	-0.5730100000	3.0993220000	C	0.4958270000	-4.5063340000	0.2775690000
C	-3.9402740000	0.2402210000	-0.5228580000	C	-0.5098110000	-5.1344150000	-0.4513380000
C	-3.4962620000	1.7100740000	-0.6398960000	H	-2.5852470000	-5.1154540000	-1.0010950000
H	-4.3052890000	-0.1735450000	-1.4678190000	H	1.4742730000	-4.9694940000	0.3532410000
H	-3.6060120000	2.1121840000	-1.6510440000	C	-0.1061780000	1.9841460000	1.8288170000
C	-1.6364330000	0.4052320000	0.0242370000	C	1.2468440000	2.0859880000	2.2261570000
N	-2.6926180000	-0.4379770000	-0.1156230000	C	-0.8664590000	3.1178850000	1.4965210000
N	-2.0652270000	1.6459500000	-0.2729310000	C	1.7865150000	3.3690580000	2.3607350000
C	-1.2564000000	2.8264310000	-0.3657980000	C	-0.2806810000	4.3806810000	1.6448590000
C	-1.0224850000	3.5987060000	0.7868770000	C	1.0280200000	4.5067840000	2.0931740000
C	-0.7635880000	3.2043630000	-1.6293730000	H	2.8226170000	3.4818500000	2.6562360000
C	-0.3118340000	4.7950080000	0.6437030000	H	-0.8512870000	5.2683330000	1.3892760000
C	-0.0483290000	4.4035100000	-1.7224210000	C	2.1181750000	0.8550270000	2.4437590000
C	0.1685440000	5.1970190000	-0.6002040000	H	1.8121930000	0.1247750000	1.6911000000
H	-0.1229930000	5.4143200000	1.5147510000	C	-2.2573790000	3.0023170000	0.9008720000
H	0.3531070000	4.7114870000	-2.6821990000	H	-2.5282870000	1.9444480000	0.8915240000
C	-2.5524610000	-1.8672270000	-0.1553220000	C	1.3192070000	-2.7354350000	1.8754440000
C	-2.1108830000	-2.4517860000	-1.3572530000	H	0.9873740000	-1.7541620000	2.2242980000

C	-2.8443690000	-2.6426810000	0.9835660000
C	-1.8475530000	-3.8265680000	-1.3642060000
C	-2.5763370000	-4.0145680000	0.9303830000
C	-2.0564660000	-4.5974400000	-0.2246610000
H	-1.4790370000	-4.2943490000	-2.2715350000
H	-2.7766600000	-4.6357730000	1.7969410000
C	-1.9834900000	-1.6493320000	-2.6447770000
H	-2.2504170000	-0.6099770000	-2.4257200000
C	-3.5197410000	-2.0341230000	2.2028530000
H	-3.2981200000	-0.9600480000	2.1997200000
C	-0.9718280000	2.3557850000	-2.8753740000
H	-1.5013120000	1.4430160000	-2.5809260000
C	-1.5234690000	3.1664170000	2.1567920000
H	-1.8118150000	2.1122230000	2.0872130000
H	0.7244220000	6.1236500000	-0.6919300000
H	-1.8374570000	-5.6596790000	-0.2413840000
C	-0.4218680000	3.2720220000	3.2254370000
H	-0.7555760000	2.8055010000	4.1591630000
H	-0.1781620000	4.3166260000	3.4502750000
H	0.4962520000	2.7763440000	2.8924680000
C	-2.7675780000	3.9760160000	2.5697360000
H	-3.5667130000	3.8855240000	1.8259430000
H	-2.5222870000	5.0400060000	2.6675930000
H	-3.1524460000	3.6247500000	3.5338570000
C	-3.0361530000	-2.6287590000	3.5365770000
H	-3.4210550000	-3.6444630000	3.6809510000
H	-3.4003730000	-2.0216940000	4.3724160000
H	-1.9449610000	-2.6731260000	3.5964450000
C	-5.0508800000	-2.2019370000	2.0903690000
H	-5.4375830000	-1.7829790000	1.1559550000
H	-5.5546360000	-1.7072300000	2.9284540000
H	-5.3164000000	-3.2653260000	2.1102040000
C	-2.9858670000	-2.1689180000	-3.6944640000
H	-4.0100530000	-2.1752750000	-3.3036980000
H	-2.7378620000	-3.1907990000	-4.0028790000
H	-2.9574760000	-1.5338630000	-4.5867030000
C	-0.5512050000	-1.6271620000	-3.1908460000
H	-0.5219990000	-1.0708110000	-4.1349190000
H	-0.1844310000	-2.6406480000	-3.3947290000
H	0.1288980000	-1.1389260000	-2.4825870000
C	-1.8359520000	3.0970030000	-3.9136290000
H	-2.7891320000	3.4298570000	-3.4862580000
H	-2.0480360000	2.4412430000	-4.7652580000
H	-1.3154000000	3.9830800000	-4.2947330000
C	0.3701160000	1.9072560000	-3.4789300000
H	0.9170560000	1.2934550000	-2.7561140000
H	0.9867450000	2.7659180000	-3.7691050000
H	0.1898850000	1.3076190000	-4.3787990000
O	1.2463380000	-0.0276150000	-1.0406000000
C	3.1652580000	1.1338270000	-0.0888910000
C	2.3999860000	2.2589140000	0.2542710000
C	4.4343850000	0.9905500000	0.4966790000
C	2.8867700000	3.2088620000	1.1536100000
H	1.4224070000	2.3836820000	-0.1949110000
C	4.9218470000	1.9391320000	1.3961590000
H	5.0270040000	0.1122520000	0.2660510000
C	4.1477760000	3.0525900000	1.7334950000
H	2.2782500000	4.0740230000	1.3959870000
H	5.9024920000	1.8036050000	1.8422340000
H	4.5230760000	3.7880770000	2.4380480000
H	4.4847990000	-0.4350880000	-1.7939030000
C	2.9534780000	-1.7732960000	-2.6766400000
C	2.7106510000	-1.3267640000	-4.1297270000
H	3.7020310000	-2.5777570000	-2.6676200000
H	2.0315590000	-2.1987560000	-2.2658600000
H	2.3638130000	-2.1604460000	-4.7527860000
C	-3.5083770000	-2.8870860000	0.2429550000
H	-3.4842990000	-1.8533120000	0.5924510000
H	-0.3061430000	-6.0686490000	-0.9635570000
H	1.4724560000	5.4899290000	2.2045530000
C	-4.2605460000	-2.9147630000	-1.0960790000
H	-5.2391000000	-2.4361850000	-0.9847370000
H	-4.4275860000	-3.9419950000	-1.4397530000
H	-3.7166380000	-2.3777040000	-1.8773630000
C	-4.2705420000	-3.7149210000	1.2999070000
H	-3.7573140000	-3.6973740000	2.2676290000
H	-4.3509480000	-4.7619830000	0.9858590000
H	-5.2837720000	-3.3201730000	1.4355910000
C	-2.2410590000	3.4728260000	-0.5649880000
H	-1.9880270000	4.5376100000	-0.6335560000
H	-3.2222930000	3.3208200000	-1.0273280000
H	-1.4964720000	2.9092240000	-1.1371520000
C	-3.3234310000	3.7513830000	1.7159870000
H	-3.3446390000	3.4063350000	2.7558420000
H	-4.3129170000	3.5878550000	1.2738940000
H	-3.1356530000	4.8311760000	1.7245760000
C	1.9493500000	0.2540760000	3.8513230000
H	0.9258850000	-0.0868180000	4.0376570000
H	2.2106100000	0.9877310000	4.6233180000
H	2.6113970000	-0.6123850000	3.9658230000
C	3.6047920000	1.1221530000	2.1616930000
H	4.1352290000	0.1671650000	2.0922740000
H	4.0794780000	1.7116520000	2.9563760000
H	3.7337720000	1.6387990000	1.2061680000
C	1.4332940000	-3.6468270000	3.1156850000
H	0.4610960000	-3.7935450000	3.6005260000
H	2.1219090000	-3.2043750000	3.8441930000
H	1.8215570000	-4.6338770000	2.8392640000
C	2.6849400000	-2.5270990000	1.2098930000
H	2.6139210000	-1.7651250000	0.4289190000
H	3.0792800000	-3.4561670000	0.7819450000
H	3.4094030000	-2.1773160000	1.9551640000
O	2.0065720000	0.0861940000	-0.5131970000
C	4.1822360000	-0.0324840000	-1.4915540000
C	4.0685400000	-1.3887520000	-1.8310070000
C	5.4622040000	0.5336300000	-1.4069470000
C	5.2026450000	-2.1542100000	-2.0990370000
H	3.0802380000	-1.8336250000	-1.8764010000
C	6.5993010000	-0.2318610000	-1.6735200000
H	5.5627340000	1.5737240000	-1.1129930000
C	6.4741000000	-1.5782630000	-2.0222210000
H	5.0956710000	-3.2011260000	-2.3657880000
H	7.5830400000	0.2204740000	-1.5964760000
H	7.3580160000	-2.1740820000	-2.2262560000
H	3.6543090000	2.4666920000	-2.2270470000
C	1.6526710000	2.9416580000	-1.3941860000
C	2.0451430000	4.4212690000	-1.2924220000
H	0.9063190000	2.8318160000	-2.2004710000
H	1.1420870000	2.6335610000	-0.4747360000
H	1.1680770000	5.0492670000	-1.1004990000
H	2.5201390000	4.7674130000	-2.2185790000
H	2.7554580000	4.5711060000	-0.4719140000
H	-2.6783390000	-1.6201490000	2.9117300000
H	-2.5245180000	0.7710720000	2.9494260000
C	-3.1270030000	0.4300730000	-1.9618850000
C	-3.7951740000	1.0572010000	-3.0264360000
C	-3.7477850000	0.4260200000	-0.7077930000
C	-5.0389930000	1.6625850000	-2.8375690000
H	-3.3402950000	1.0595110000	-4.0125310000
C	-4.9959620000	1.0163850000	-0.5156530000
H	-3.2268180000	-0.0239930000	0.1272190000
C	-5.6472930000	1.6420000000	-1.5810800000

H	3.6296550000	-0.9270030000	-4.5739470000	H	-5.5344720000	2.1440910000	-3.6739290000
H	1.9543560000	-0.5361660000	-4.1582040000	H	-5.4527400000	1.0023840000	0.4689720000
H	-4.0285900000	2.3689800000	0.0509280000	H	-6.6154350000	2.1083790000	-1.4341450000
H	-4.7133200000	0.0989930000	0.2364610000	H	1.2410140000	0.4610830000	-3.6028830000
**IM1-eta1**				**IM2-eta1**			
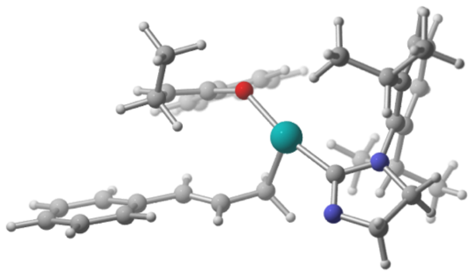				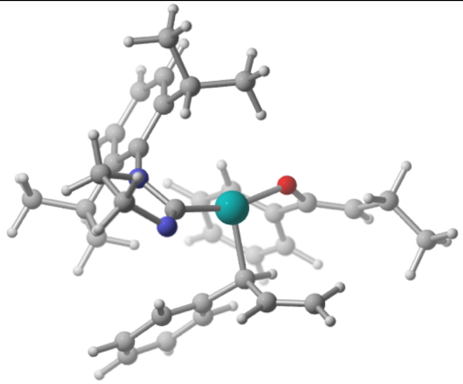			
Gas phase energy=		-459.557806479		Gas phase energy=		-459.548605304	
Gas phase free energy correction=		0.865423		Gas phase free energy correction=		0.869216	
SMD energy=		-2100.65073592		SMD energy=		-2100.63777372	
C	2.8261930000	2.1428670000	-1.7849400000	C	-2.8646410000	-3.8823060000	0.0960950000
C	1.6730810000	2.5337560000	-1.1955150000	C	-2.7065100000	-2.5437840000	0.1968740000
C	3.1150310000	0.5946160000	0.8661650000	C	1.0506190000	-3.1864560000	-1.8012460000
H	3.0450720000	1.6427910000	1.1493720000	H	0.2093510000	-3.7973810000	-1.4817530000
C	2.0080160000	-0.1722960000	0.9514730000	C	0.9418350000	-1.8539500000	-1.9003000000
C	0.6818870000	0.3485090000	1.3144650000	C	-0.3099170000	-1.1103440000	-1.6007120000
H	2.0685740000	-1.2341670000	0.7252740000	H	1.8046890000	-1.2717220000	-2.2152810000
H	0.7061110000	1.3938720000	1.6413090000	H	-1.1607970000	-1.7999820000	-1.5997960000
Pd	-0.4346480000	0.3973110000	-0.4158360000	Pd	-0.3018940000	-0.6152120000	0.4014230000
C	4.4448160000	0.1393310000	0.4352240000	C	3.0886660000	2.0132570000	1.0648940000
C	5.5559720000	0.9738590000	0.6470920000	C	1.8797640000	2.8433330000	1.5232720000
C	4.6533460000	-1.0926700000	-0.2114580000	H	3.6768480000	1.6192780000	1.9014630000
C	6.8336020000	0.5890650000	0.2421950000	H	1.9093020000	3.0943930000	2.5865210000
H	5.4079930000	1.9340340000	1.1339280000	C	1.1098080000	0.8269180000	0.5364390000
C	5.9308230000	-1.4788490000	-0.6127050000	N	2.4539850000	0.8827480000	0.3436660000
H	3.8079060000	-1.7391450000	-0.4239100000	N	0.7609300000	1.9277000000	1.2455380000
C	7.0281080000	-0.6424380000	-0.3873170000	C	-0.5417680000	2.1218650000	1.8188720000
H	7.6761400000	1.2506840000	0.4155730000	C	-1.4935530000	2.9002500000	1.1374780000
H	6.0707500000	-2.4311640000	-1.1146400000	C	-0.8211820000	1.5197210000	3.0628600000
H	8.0203850000	-0.9433450000	-0.7068260000	C	-2.7520110000	3.0597290000	1.7266790000
H	0.1393650000	-0.2681080000	2.0340570000	C	-2.1005090000	1.6878090000	3.6029980000
C	-2.6342790000	-3.0626470000	0.9282990000	C	-3.0585190000	2.4500950000	2.9408590000
C	-3.7207260000	-1.9729550000	0.9293830000	H	-3.5051750000	3.6580020000	1.2250530000
H	-2.7804700000	-3.8061330000	0.1360910000	H	-2.3492140000	1.2158510000	4.5480460000
H	-4.5840790000	-2.2243510000	0.3086770000	C	3.2810200000	-0.1691430000	-0.1795540000
C	-1.6616620000	-1.0125560000	0.2666080000	C	3.4026600000	-1.3834270000	0.5291890000
N	-1.4059390000	-2.2859470000	0.6523330000	C	3.9984050000	0.0596690000	-1.3737730000
N	-2.9951250000	-0.8203450000	0.3670370000	C	4.2252830000	-2.3794490000	-0.0087680000
C	-3.6580070000	0.4086630000	0.0453300000	C	4.8122520000	-0.9654300000	-1.8666260000
C	-3.7567680000	1.4162750000	1.0197510000	C	4.9185970000	-2.1805530000	-1.1972970000
C	-4.1802230000	0.5657230000	-1.2526270000	H	4.3228730000	-3.3257690000	0.5124170000
C	-4.4293320000	2.5946060000	0.6768080000	H	5.3647480000	-0.8129320000	-2.7879000000
C	-4.8373270000	1.7620030000	-1.5554460000	C	2.7221480000	-1.6465800000	1.8666110000
C	-4.9678170000	2.7645830000	-0.5958030000	H	2.0073740000	-0.8422760000	2.0550570000
H	-4.5198230000	3.3911600000	1.4086320000	C	3.9485790000	1.3839760000	-2.1271920000
H	-5.2415910000	1.9199150000	-2.5496250000	H	3.1285670000	1.9811860000	-1.7104620000
C	-0.1338390000	-2.9370320000	0.5420400000	C	0.2190710000	0.7120390000	3.8278070000
C	0.3745780000	-3.2439430000	-0.7360450000	H	1.1453750000	0.7081580000	3.2421700000
C	0.5705980000	-3.2628450000	1.7183120000	C	-1.1705450000	3.5753900000	-0.1848490000
C	1.6269500000	-3.8652240000	-0.8123110000	H	-0.2550240000	3.1200650000	-0.5795760000

C	1.8076930000	-3.9034390000	1.5927800000	H	-4.0465030000	2.5691780000	3.3716770000
C	2.3374800000	-4.1945870000	0.3386810000	H	5.5480510000	-2.9678820000	-1.5974730000
H	2.0489230000	-4.0964100000	-1.7851870000	C	-2.2674950000	3.3528210000	-1.2346710000
H	2.3724160000	-4.1568700000	2.4836420000	H	-1.9650150000	3.7939880000	-2.1886810000
C	-0.3900310000	-2.9558240000	-2.0226140000	H	-3.2152640000	3.8176260000	-0.9385650000
H	-1.3158410000	-2.4289720000	-1.7673550000	H	-2.4405210000	2.2853150000	-1.3979690000
C	0.0253850000	-2.9385350000	3.1024030000	C	-0.9108020000	5.0799280000	0.0307160000
H	-0.8238020000	-2.2560000000	2.9719210000	H	-0.1236930000	5.2514930000	0.7739050000
C	-3.9599840000	-0.4982310000	-2.3198290000	H	-1.8190400000	5.5801140000	0.3873120000
H	-3.7131030000	-1.4386760000	-1.8117690000	H	-0.6092860000	5.5531730000	-0.9102580000
C	-3.1257950000	1.2692140000	2.3959860000	C	3.6735200000	1.1964570000	-3.6309420000
H	-2.5801400000	0.3189260000	2.4178220000	H	4.5261910000	0.7280690000	-4.1344200000
H	-5.4794690000	3.6874220000	-0.8463550000	H	3.5064970000	2.1695410000	-4.1056990000
H	3.3071520000	-4.6733720000	0.2582870000	H	2.7908600000	0.5762620000	-3.8086240000
C	-2.0979200000	2.3857290000	2.6530030000	C	5.2629350000	2.1685720000	-1.9313590000
H	-1.5613150000	2.2002010000	3.5899150000	H	5.5018120000	2.3093500000	-0.8720330000
H	-2.5845280000	3.3646110000	2.7347320000	H	5.1957950000	3.1533080000	-2.4071050000
H	-1.3666970000	2.4371390000	1.8409780000	H	6.1005740000	1.6284070000	-2.3872340000
C	-4.1946550000	1.2255140000	3.5033320000	C	3.7507920000	-1.6210780000	3.0131910000
H	-4.9130710000	0.4158160000	3.3329890000	H	4.2954500000	-0.6702550000	3.0388410000
H	-4.7548320000	2.1667850000	3.5442810000	H	4.4863600000	-2.4259860000	2.9005180000
H	-3.7240940000	1.0718320000	4.4806800000	H	3.2452400000	-1.7560550000	3.9762690000
C	1.0616420000	-2.2230390000	3.9890090000	C	1.9180020000	-2.9576050000	1.8655710000
H	1.8792950000	-2.8965760000	4.2685230000	H	1.3716460000	-3.0624730000	2.8083470000
H	0.5872170000	-1.8804020000	4.9151060000	H	2.5706010000	-3.8319620000	1.7600230000
H	1.4984420000	-1.3572290000	3.4829580000	H	1.1874170000	-2.9670610000	1.0508930000
C	-0.4788510000	-4.2156330000	3.8046820000	C	0.5443800000	1.3698500000	5.1820120000
H	-1.2130120000	-4.7545370000	3.1962370000	H	0.8683630000	2.4081320000	5.0507290000
H	-0.9400650000	-3.9689160000	4.7673460000	H	1.3440120000	0.8182460000	5.6887740000
H	0.3552820000	-4.9010590000	3.9945550000	H	-0.3316450000	1.3724060000	5.8401320000
C	-0.7884290000	-4.2679770000	-2.7255680000	C	-0.2164180000	-0.7519920000	4.0147540000
H	-1.3775770000	-4.9115140000	-2.0627760000	H	-0.4677790000	-1.2376770000	3.0643630000
H	0.0968840000	-4.8312580000	-3.0415700000	H	-1.1047500000	-0.8195460000	4.6529260000
H	-1.3853990000	-4.0506330000	-3.6183750000	H	0.5863480000	-1.3246330000	4.4933790000
C	0.4060190000	-2.0394350000	-2.9666300000	O	-1.6612660000	-2.0095580000	0.8801840000
H	-0.1728290000	-1.8279110000	-3.8720100000	C	-3.6699720000	-1.5693920000	-0.4032690000
H	1.3510630000	-2.5009820000	-3.2745240000	C	-3.7702270000	-0.2729600000	0.1242660000
H	0.6383780000	-1.0852410000	-2.4820820000	C	-4.4549410000	-1.8976410000	-1.5212770000
C	-5.2081160000	-0.7571420000	-3.1791320000	C	-4.6344280000	0.6629080000	-0.4435230000
H	-6.0772830000	-0.9928770000	-2.5563250000	H	-3.1644140000	-0.0038990000	0.9826570000
H	-5.0280400000	-1.6002020000	-3.8548960000	C	-5.3213840000	-0.9619300000	-2.0874360000
H	-5.4597210000	0.1096250000	-3.7998280000	H	-4.3637240000	-2.8825940000	-1.9677130000
C	-2.7524630000	-0.1233740000	-3.2008200000	C	-5.4123390000	0.3259970000	-1.5530590000
H	-1.8389150000	-0.0030920000	-2.6045230000	H	-4.6945420000	1.6592260000	-0.0176320000
H	-2.9289370000	0.8244250000	-3.7210020000	H	-5.9144160000	-1.2342680000	-2.9550070000
H	-2.5644540000	-0.9013990000	-3.9493780000	H	-6.0795240000	1.0574820000	-1.9974970000
O	0.5244430000	1.8469720000	-1.3881820000	H	-3.7676400000	-4.2795200000	-0.3644300000
C	1.5651110000	3.6978790000	-0.2650760000	C	-1.8776410000	-4.8817560000	0.6303930000
C	0.2922550000	4.1787040000	0.0794890000	H	-0.9371200000	-4.3651250000	0.8537120000
C	2.6873680000	4.2990950000	0.3300530000	C	-1.6352410000	-6.0437530000	-0.3459870000
C	0.1445180000	5.2304250000	0.9839570000	H	-1.2431840000	-5.6750540000	-1.3010820000
H	-0.5734980000	3.7085030000	-0.3720320000	H	-0.9174200000	-6.7641210000	0.0623080000
C	2.5399810000	5.3544390000	1.2304770000	H	-2.5680730000	-6.5802650000	-0.5574700000
H	3.6826270000	3.9310990000	0.1010930000	H	1.7615390000	3.7683190000	0.9496300000
C	1.2671980000	5.8251480000	1.5639640000	H	3.7551770000	2.5720140000	0.4079840000
H	-0.8501270000	5.5859740000	1.2354250000	H	-2.2328320000	-5.2922760000	1.5884960000
H	3.4198120000	5.8035060000	1.6805430000	C	-0.6105160000	0.0668500000	-2.4786070000
H	1.1536390000	6.6403860000	2.2712670000	C	-1.8354380000	0.1257090000	-3.1611470000
H	3.7226330000	2.7461430000	-1.6714900000	C	0.3211130000	1.0914770000	-2.7020640000
C	2.9266730000	0.8886080000	-2.6094580000	C	-2.1005360000	1.1524130000	-4.0690190000
C	2.2401320000	0.9877790000	-3.9834840000	H	-2.5817460000	-0.6408930000	-2.9810230000
H	3.9832140000	0.6251860000	-2.7408630000	C	0.0623510000	2.1151090000	-3.6108780000
H	2.4669380000	0.0620940000	-2.0473020000	H	1.2520850000	1.0823010000	-2.1479100000
H	2.3280620000	0.0432020000	-4.5348350000	C	-1.1483270000	2.1441420000	-4.3095300000
H	2.6899840000	1.7815610000	-4.5899490000	H	-3.0538230000	1.1732410000	-4.5863820000
H	1.1789620000	1.2190700000	-3.8488510000	H	0.8065590000	2.8888370000	-3.7763180000

H	-4.0769910000	-1.7326770000	1.9376110000	H	-1.3500440000	2.9363730000	-5.0231540000
H	-2.5571970000	-3.5851050000	1.8826470000	H	1.9837210000	-3.6951740000	-2.0189920000
**TS-inner-branched (or TS2)**				**TS-inner-linear**			
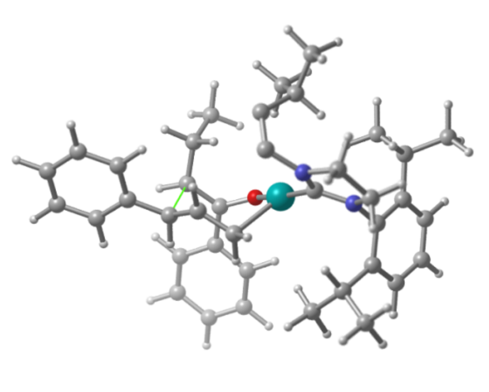				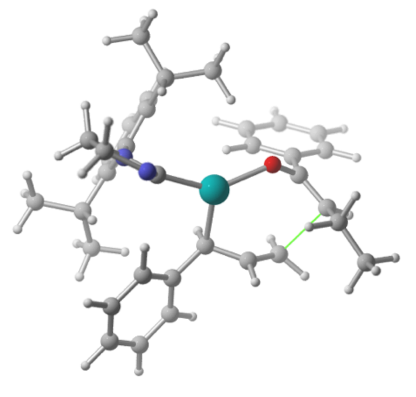			
Gas phase energy=	-459.539803013			Gas phase energy=	-459.523872303		
Gas phase free energy correction=	0.869345			Gas phase free energy correction=	0.868289		
SMD energy=	-2100.63186908			SMD energy=	-2100.61314516		
C	3.003507	1.178005	-1.446355	C	-2.9085020000	-2.8182690000	-1.9607880000
C	1.987788	2.134230	-1.252318	C	-2.9895890000	-2.0315630000	-0.8164490000
C	3.195684	0.478187	0.501530	C	-2.1663210000	-1.0898290000	-3.1510250000
H	3.263117	1.479711	0.924458	H	-2.9581020000	-0.4000680000	-2.8731070000
C	2.053566	-0.264947	0.871096	C	-0.8437380000	-0.7729810000	-2.8624280000
C	0.855215	0.302560	1.347197	C	-0.3966200000	0.3728100000	-2.1052450000
H	2.075261	-1.344365	0.748484	H	-0.0630260000	-1.3710500000	-3.3311470000
H	0.891173	1.348168	1.663324	H	-1.2025860000	1.0756160000	-1.8460340000
Pd	-0.443214	0.472884	-0.400432	Pd	-0.2433180000	-0.5987930000	-0.2438770000
C	4.525052	-0.189071	0.397138	C	2.9839740000	0.7810870000	2.2625180000
C	5.686064	0.566824	0.629243	C	2.0096420000	1.9668060000	2.3850490000
C	4.670374	-1.545769	0.063064	H	3.1353080000	0.2494730000	3.2056690000
C	6.951954	-0.011770	0.537861	H	1.5893730000	2.0706200000	3.3907930000
H	5.587553	1.618101	0.888138	C	1.1310480000	0.4139570000	0.8155710000
C	5.936251	-2.124347	-0.028046	N	2.3043030000	-0.0965380000	1.2843610000
H	3.792351	-2.151952	-0.134860	N	0.9415890000	1.6103670000	1.4298580000
C	7.083267	-1.362528	0.208717	C	-0.3047830000	2.3256320000	1.4321980000
H	7.834964	0.590019	0.726852	C	-0.4730040000	3.4388300000	0.5885740000
H	6.028227	-3.174482	-0.286774	C	-1.3207450000	1.9056500000	2.3156280000
H	8.066100	-1.815977	0.135542	C	-1.7024200000	4.1075290000	0.6128650000
H	0.223620	-0.320654	1.975178	C	-2.5342830000	2.6020320000	2.3045120000
C	-2.933354	-2.764355	1.035236	C	-2.7278520000	3.6887500000	1.4562840000
C	-3.885676	-1.572985	1.220676	H	-1.8588620000	4.9620480000	-0.0369860000
H	-3.255548	-3.440852	0.233923	H	-3.3352220000	2.2872970000	2.9663380000
H	-4.852926	-1.712967	0.731908	C	2.7860490000	-1.4302600000	1.0445540000
C	-1.838931	-0.785115	0.311738	C	2.0933440000	-2.5321120000	1.6029470000
N	-1.673419	-2.097213	0.645788	C	3.9750730000	-1.6100700000	0.3122400000
N	-3.131038	-0.479341	0.581391	C	2.6326050000	-3.8088760000	1.4196930000
C	-3.726795	0.803677	0.362969	C	4.4849130000	-2.9072650000	0.1733360000
C	-3.592969	1.806436	1.337729	C	3.8226210000	-3.9982420000	0.7214400000
C	-4.439839	1.013942	-0.832746	H	2.1176370000	-4.6691070000	1.8308970000
C	-4.215838	3.038000	1.103615	H	5.4072510000	-3.0628870000	-0.3772050000
C	-5.038601	2.261570	-1.031578	C	0.8152760000	-2.3661700000	2.4193630000
C	-4.933082	3.263558	-0.068010	H	0.2955980000	-1.4742110000	2.0537730000
H	-4.129095	3.830466	1.840706	C	4.7280910000	-0.4554960000	-0.3363040000
H	-5.582580	2.458752	-1.949338	H	4.1160980000	0.4481490000	-0.2388300000
C	-0.496738	-2.854739	0.338034	C	-1.1276200000	0.7633610000	3.3049900000
C	-0.195459	-3.134999	-1.010513	H	-0.1380590000	0.3275140000	3.1362580000
C	0.326225	-3.307488	1.388589	C	0.6542210000	3.9424140000	-0.2965930000
C	0.983761	-3.834868	-1.290365	H	1.3733370000	3.1255950000	-0.4096320000
C	1.482874	-4.024647	1.062360	H	-3.6762390000	4.2160830000	1.4584630000

C	1.818201	-4.273801	-0.266022	H	4.2278840000	-4.9971730000	0.6008660000
H	1.245064	-4.048164	-2.321981	C	0.1750820000	4.3372920000	-1.7020100000
H	2.137457	-4.375670	1.853265	H	1.0342370000	4.4617230000	-2.3691510000
C	-1.132253	-2.764368	-2.154675	H	-0.3794550000	5.2831970000	-1.6875560000
H	-1.949572	-2.158537	-1.750835	H	-0.4705240000	3.5669590000	-2.1312120000
C	-0.025454	-3.060534	2.849481	C	1.3738750000	5.1327950000	0.3706040000
H	-0.805853	-2.289448	2.873311	H	1.7436010000	4.8780550000	1.3696240000
C	-4.491902	-0.059384	-1.911570	H	0.6883290000	5.9819360000	0.4779520000
H	-4.245857	-1.018735	-1.440622	H	2.2237480000	5.4543150000	-0.2418180000
C	-2.776597	1.592040	2.603287	C	4.9439630000	-0.7020900000	-1.8405440000
H	-2.307043	0.604509	2.536344	H	5.5973440000	-1.5643680000	-2.0127630000
H	-5.404121	4.226088	-0.236547	H	5.4167910000	0.1694700000	-2.3055430000
H	2.727219	-4.816980	-0.502339	H	3.9924000000	-0.8832560000	-2.3486250000
C	-1.641083	2.625466	2.709451	C	6.0738580000	-0.1998260000	0.3700440000
H	-0.982002	2.382211	3.550222	H	5.9396580000	-0.0294660000	1.4437790000
H	-2.034727	3.635743	2.871074	H	6.5721900000	0.6757250000	-0.0610010000
H	-1.043902	2.631494	1.792137	H	6.7427480000	-1.0602470000	0.2529330000
C	-3.669302	1.610198	3.857615	C	1.1410970000	-2.1349280000	3.9069270000
H	-4.461265	0.855974	3.790904	H	1.7821760000	-1.2580670000	4.0486460000
H	-4.147754	2.588263	3.984852	H	1.6592260000	-3.0047390000	4.3275010000
H	-3.073179	1.407225	4.754313	H	0.2178590000	-1.9762310000	4.4758690000
C	1.166460	-2.545129	3.676303	C	-0.1762180000	-3.5256910000	2.2476620000
H	1.935531	-3.317103	3.792139	H	-1.1247640000	-3.2657540000	2.7272560000
H	0.828888	-2.266788	4.680798	H	0.1880880000	-4.4512610000	2.7085630000
H	1.633578	-1.670854	3.214580	H	-0.3871420000	-3.7044800000	1.1896780000
C	-0.589593	-4.345731	3.490889	C	-1.1507520000	1.2911240000	4.7532140000
H	-1.434780	-4.749341	2.923527	H	-0.4132570000	2.0882960000	4.9002950000
H	-0.920544	-4.150532	4.517036	H	-0.9273200000	0.4789630000	5.4541770000
H	0.183422	-5.122242	3.525396	H	-2.1365580000	1.6959390000	5.0086250000
C	-1.754313	-4.035353	-2.766485	C	-2.1598880000	-0.3602320000	3.1130190000
H	-2.262734	-4.635243	-2.003436	H	-2.0906170000	-0.8089220000	2.1171920000
H	-0.986908	-4.664582	-3.231795	H	-3.1831400000	0.0100940000	3.2449130000
H	-2.483908	-3.764797	-3.538064	H	-1.9948160000	-1.1482230000	3.8574820000
C	-0.440753	-1.914300	-3.231138	O	-1.9239410000	-1.8520230000	-0.0881260000
H	-1.142598	-1.686439	-4.040652	C	-4.1848320000	-1.1899440000	-0.5248150000
H	0.419247	-2.433063	-3.669598	C	-4.0130350000	0.0912600000	0.0230380000
H	-0.090513	-0.967233	-2.807948	C	-5.4853670000	-1.6428140000	-0.7927050000
C	-5.882492	-0.204050	-2.550482	C	-5.1163970000	0.9017540000	0.2859800000
H	-6.655554	-0.359034	-1.790439	H	-3.0105000000	0.4475770000	0.2370930000
H	-5.892482	-1.059974	-3.234131	C	-6.5894540000	-0.8331330000	-0.5230170000
H	-6.153225	0.682499	-3.134401	H	-5.6328960000	-2.6409270000	-1.1933200000
C	-3.414483	0.209384	-2.979602	C	-6.4082940000	0.4431810000	0.0151710000
H	-2.418938	0.271920	-2.525011	H	-4.9636540000	1.8930180000	0.6998790000
H	-3.608135	1.157911	-3.493713	H	-7.5902370000	-1.2008190000	-0.7251750000
H	-3.405801	-0.592862	-3.726601	H	-7.2667250000	1.0730560000	0.2246760000
O	0.737487	1.797726	-1.403311	H	-3.8109730000	-2.9792480000	-2.5477590000
C	2.253231	3.459825	-0.631771	C	-1.7914910000	-3.8207220000	-2.1195980000
C	1.167902	4.184133	-0.110566	H	-0.8350730000	-3.3269500000	-1.9032930000
C	3.544307	4.000752	-0.504720	C	-1.7485120000	-4.4834110000	-3.5003490000
C	1.367300	5.406862	0.526950	H	-1.5365660000	-3.7527280000	-4.2891950000
H	0.173955	3.766131	-0.219403	H	-0.9662330000	-5.2483210000	-3.5416960000
C	3.741977	5.226087	0.132111	H	-2.7036550000	-4.9667180000	-3.7379920000
H	4.401142	3.476286	-0.914179	H	2.4705670000	2.9168310000	2.1051540000
C	2.655523	5.932809	0.653363	H	3.9625500000	1.0904560000	1.8865210000
H	0.516652	5.949442	0.926381	H	-1.8997030000	-4.5955070000	-1.3459240000
H	4.744643	5.632080	0.218390	C	0.7779330000	1.1096380000	-2.7212420000
H	2.811662	6.884995	1.149239	C	0.5609530000	1.6981000000	-3.9825720000
H	4.024569	1.547665	-1.507331	C	2.0347950000	1.2689420000	-2.1371580000
C	2.720533	-0.057176	-2.271294	C	1.5534570000	2.4446820000	-4.6137080000
C	2.397721	0.267568	-3.737692	H	-0.4047050000	1.5660080000	-4.4614880000
H	3.591991	-0.720693	-2.227602	C	3.0310970000	2.0274340000	-2.7594930000
H	1.880558	-0.604979	-1.825012	H	2.2303160000	0.7885020000	-1.1898790000
H	2.221052	-0.651322	-4.308567	C	2.7957260000	2.6231610000	-3.9971090000
H	3.224355	0.807990	-4.212642	H	1.3583610000	2.8909800000	-5.5834390000
H	1.499145	0.889461	-3.799565	H	3.9958350000	2.1431960000	-2.2747150000
H	-4.063120	-1.337053	2.276857	H	3.5696720000	3.2090830000	-4.4819160000

H -2.804078 -3.346749 1.948455	H -2.3784950000 -1.7069340000 -4.0162310000

**TS1**	**TS3**
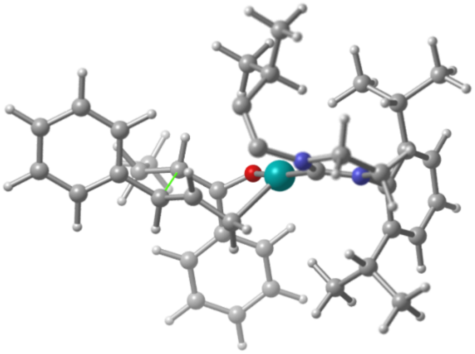	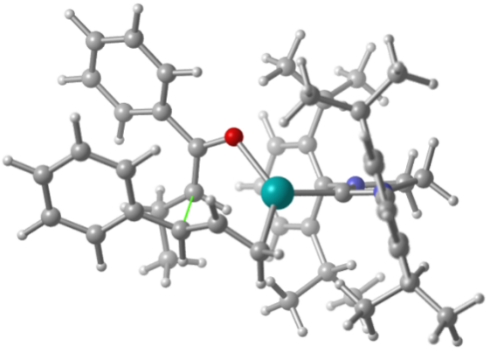
Gas phase energy= -459.529216242<br>Gas phase free energy correction= 0.866850<br>SMD energy= -2100.62027722	Gas phase energy= -459.533926723<br>Gas phase free energy correction= 0.870864<br>SMD energy= -2100.62448381
C 2.982461 1.629145 -1.263027	C -2.327892 0.854122 -0.814631
C 1.834150 2.380200 -0.935965	C -2.451183 0.184860 0.416570
C 3.191997 0.422252 0.435940	C -1.896686 -1.043051 -2.128761
H 3.421678 1.294957 1.048077	H -1.746546 -0.327345 -2.931042
C 2.014864 -0.288543 0.762159	C -0.750041 -1.767731 -1.706127
C 0.853547 0.262116 1.340612	C 0.605131 -1.467172 -2.114867
H 1.997755 -1.350804 0.531925	H -0.921414 -2.717973 -1.205152
H 0.908356 1.287363 1.715425	H 0.755934 -0.769312 -2.940226
Pd -0.450129 0.435846 -0.389313	Pd 0.385458 -0.517558 -0.281759
C 4.380288 -0.387748 0.046141	C -3.256265 -1.600922 -2.024749
C 5.639741 -0.132505 0.606769	C -4.243783 -1.209294 -2.945058
C 4.263313 -1.420973 -0.898565	C -3.611246 -2.503370 -1.005558
C 6.751610 -0.894886 0.243532	C -5.547635 -1.696943 -2.845908
H 5.744063 0.664224 1.338059	H -3.982740 -0.521520 -3.744727
C 5.372814 -2.182748 -1.261363	C -4.914956 -2.978606 -0.900625
H 3.297226 -1.617627 -1.354720	H -2.874559 -2.797163 -0.266102
C 6.623194 -1.923466 -0.692068	C -5.889387 -2.578333 -1.819289
H 7.717448 -0.686699 0.692316	H -6.295483 -1.383890 -3.567044
H 5.265292 -2.973305 -1.997492	H -5.176408 -3.647507 -0.088093
H 7.487789 -2.513281 -0.978220	H -6.905152 -2.948519 -1.731754
H 0.231309 -0.388970 1.947426	H 1.329952 -2.280414 -2.103125
C -2.871598 -2.961357 0.816878	C 3.381645 2.149122 1.277173
C -3.923363 -1.841043 0.779666	C 4.117863 0.805166 1.415728
H -3.003863 -3.692905 0.010348	H 3.268259 2.672586 2.228586
H -4.770271 -2.066725 0.126570	H 4.157685 0.455122 2.454903
C -1.805858 -0.930313 0.205357	C 2.041473 0.431231 0.337179
N -1.610370 -2.217264 0.607580	N 2.069358 1.722219 0.757521
N -3.141874 -0.710922 0.247868	N 3.261643 -0.094216 0.617834
C -3.767489 0.520436 -0.126517	C 3.623663 -1.451460 0.347365
C -3.862259 1.563857 0.809562	C 3.173878 -2.471335 1.202354
C -4.265069 0.644295 -1.437735	C 4.408872 -1.718266 -0.791546
C -4.498228 2.745898 0.412443	C 3.511634 -3.792177 0.881602
C -4.890907 1.842591 -1.794256	C 4.721844 -3.050820 -1.075538
C -5.012435 2.882956 -0.874159	C 4.272148 -4.079918 -0.247630
H -4.581258 3.570078 1.114418	H 3.171756 -4.601465 1.520621
H -5.275601 1.972434 -2.800575	H 5.316184 -3.293990 -1.949476

C	-0.356015	-2.908757	0.549871
C	0.198776	-3.235031	-0.704677
C	0.290121	-3.258583	1.753428
C	1.428536	-3.903501	-0.728229
C	1.512225	-3.935559	1.679872
C	2.083358	-4.249173	0.450038
H	1.882314	-4.152313	-1.682668
H	2.033325	-4.201272	2.593469
C	-0.485758	-2.900199	-2.023683
H	-1.393297	-2.327538	-1.808360
C	-0.307640	-2.926502	3.114131
H	-1.111392	-2.197382	2.950625
C	-4.049348	-0.459336	-2.464896
H	-3.796788	-1.378556	-1.922888
C	-3.257775	1.452839	2.201332
H	-2.810532	0.456914	2.296471
H	-5.499055	3.807489	-1.166106
H	3.043564	-4.750983	0.409326
C	-2.122926	2.477439	2.385143
H	-1.637293	2.339942	3.357844
H	-2.504356	3.504443	2.339815
H	-1.366901	2.358403	1.601745
C	-4.327920	1.596882	3.298484
H	-5.121216	0.850748	3.178520
H	-4.792656	2.589113	3.271144
H	-3.876276	1.465809	4.288095
C	0.716270	-2.286411	4.070366
H	1.471640	-3.013129	4.389450
H	0.209626	-1.923044	4.971228
H	1.235286	-1.445951	3.600722
C	-0.914903	-4.185949	3.765980
H	-1.640451	-4.677132	3.109267
H	-1.415283	-3.930172	4.706608
H	-0.127287	-4.915497	3.987961
C	-0.911455	-4.181993	-2.764499
H	-1.559774	-4.804889	-2.138233
H	-0.039950	-4.783576	-3.047677
H	-1.456213	-3.926167	-3.680370
C	0.405098	-2.014726	-2.911784
H	-0.132202	-1.727081	-3.821961
H	1.317791	-2.542190	-3.214386
H	0.690046	-1.099230	-2.380538
C	-5.302291	-0.751843	-3.306110
H	-6.166038	-0.971240	-2.669536
H	-5.123168	-1.615725	-3.955597
H	-5.562712	0.093850	-3.951987
C	-2.843237	-0.117129	-3.360934
H	-1.940856	0.048791	-2.759888
H	-3.034955	0.796944	-3.934690
H	-2.643910	-0.932498	-4.066439
O	0.673580	1.872386	-1.270826
C	1.781086	3.577298	-0.053949
C	0.535070	4.205825	0.130747
C	2.885492	4.075247	0.658565
C	0.400413	5.292626	0.989596
H	-0.320720	3.813211	-0.404587
C	2.748736	5.163760	1.522208
H	3.859317	3.616587	0.552105
C	1.507769	5.778231	1.691481
H	-0.570849	5.760127	1.115230
H	3.615061	5.528617	2.064451
H	1.403538	6.623217	2.363958
H	2.723913	0.818316	-1.943535
C	4.380020	2.186966	-1.497922
C	4.395640	3.402278	-2.438712
H	4.888743	2.439740	-0.559196
C	0.975367	2.629515	0.589775
C	0.809029	3.268754	-0.652259
C	0.086846	2.837838	1.661481
C	-0.279364	4.133337	-0.808652
C	-0.998224	3.696526	1.455694
C	-1.181004	4.338549	0.232934
H	-0.428055	4.642600	-1.756427
H	-1.709769	3.861531	2.258006
C	2.351853	-2.177452	2.450117
H	2.156521	-1.100248	2.483955
C	4.854110	-0.584343	-1.709417
H	4.960227	0.317888	-1.094914
C	0.317927	2.194830	3.021577
H	1.085739	1.422862	2.892547
C	1.763018	3.022578	-1.812955
H	2.529192	2.316606	-1.475408
H	4.518086	-5.109642	-0.484947
H	-2.028670	5.000932	0.091685
C	3.135047	-2.552738	3.722688
H	4.100967	-2.036877	3.760738
H	3.328375	-3.631009	3.763225
H	2.561137	-2.279954	4.615390
C	0.982359	-2.877263	2.412499
H	1.092251	-3.968460	2.410628
H	0.420804	-2.578603	1.520723
H	0.391689	-2.595704	3.291131
C	0.851524	3.245235	4.017880
H	1.092925	2.773537	4.976946
H	0.095935	4.018471	4.200603
H	1.751111	3.743549	3.639052
C	-0.932090	1.497862	3.581713
H	-1.278658	0.725389	2.890609
H	-1.747669	2.208835	3.758193
H	-0.693789	1.028515	4.543442
C	3.773041	-0.278745	-2.763216
H	3.607456	-1.150331	-3.407321
H	4.081823	0.562731	-3.395050
H	2.823002	-0.029219	-2.282239
C	6.212972	-0.843181	-2.378512
H	6.148292	-1.641038	-3.126731
H	6.975502	-1.123335	-1.644299
H	6.549124	0.060897	-2.897531
C	1.036355	2.364388	-2.998079
H	1.752765	2.089479	-3.780346
H	0.298318	3.045161	-3.438250
H	0.518402	1.458208	-2.668528
C	2.476565	4.316666	-2.243390
H	3.007228	4.773420	-1.400935
H	1.762328	5.053349	-2.628836
H	3.202147	4.105171	-3.036842
H	-1.304910	1.174184	-1.018562
C	-3.376242	1.660418	-1.542857
C	-2.836419	2.297699	-2.829056
H	-3.742278	2.471286	-0.894358
H	-4.250290	1.041339	-1.788147
H	-3.603585	2.912107	-3.311982
H	-1.975034	2.935490	-2.607052
H	-2.511922	1.543156	-3.553843
C	-3.726198	-0.142910	1.127518
C	-3.664288	-1.130692	2.127485
C	-4.974196	0.433945	0.846575
C	-4.807714	-1.547978	2.800876
H	-2.697363	-1.565564	2.347089
C	-6.122543	0.014589	1.521489
H	-5.073132	1.212035	0.106275
C	-6.047807	-0.981136	2.494270

H	4.986090	1.386417	-1.935794	H	-4.733810	-2.317340	3.562717
H	5.423791	3.720191	-2.644819	H	-7.077653	0.470962	1.283155
H	3.856316	4.250053	-2.003447	H	-6.943461	-1.308109	3.012117
H	3.915558	3.156726	-3.391864	O	-1.381946	-0.340566	0.956474
H	-4.311333	-1.592829	1.774796	H	3.868291	2.824981	0.562681
H	-2.858085	-3.495910	1.767562	H	5.136446	0.829955	1.022752

TS2 (or TS-inner-branched)	TS4
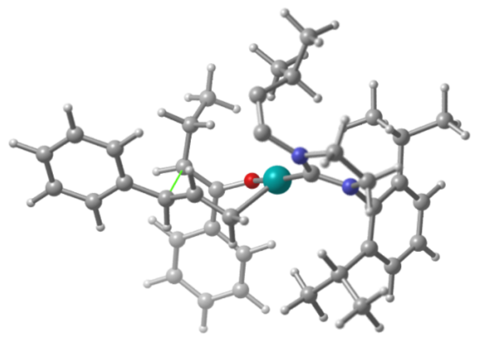	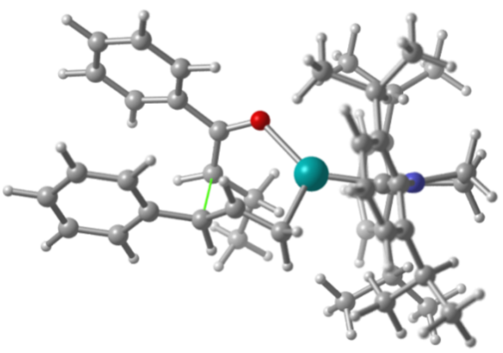
Gas phase energy= -459.539803013	Gas phase energy= -459.537279738
Gas phase free energy correction= 0.869345	Gas phase free energy correction= 0.868156
SMD energy= -2100.63186908	SMD energy= -2100.62852628

C	3.003507	1.178005	-1.446355	C	-2.702043	1.555400	-0.387758
C	1.987788	2.134230	-1.252318	C	-2.690233	0.748114	0.755797
C	3.195684	0.478187	0.501530	C	-2.480071	-0.205234	-1.828722
H	3.263117	1.479711	0.924458	H	-2.121881	0.522174	-2.555343
C	2.053566	-0.264947	0.871096	C	-1.528494	-1.142780	-1.377017
C	0.855215	0.302560	1.347197	C	-0.144979	-1.149873	-1.742844
H	2.075261	-1.344365	0.748484	H	-1.881237	-1.988404	-0.791438
H	0.891173	1.348168	1.663324	H	0.159718	-0.521594	-2.581273
Pd	-0.443214	0.472884	-0.400432	Pd	0.196979	-0.164272	0.055594
C	4.525052	-0.189071	0.397138	C	-3.925237	-0.515010	-1.941460
C	5.686064	0.566824	0.629243	C	-4.744535	0.327909	-2.713065
C	4.670374	-1.545769	0.063064	C	-4.523168	-1.610927	-1.298304
C	6.951954	-0.011770	0.537861	C	-6.112743	0.092711	-2.830602
H	5.587553	1.618101	0.888138	H	-4.298227	1.183072	-3.214985
C	5.936251	-2.124347	-0.028046	C	-5.893098	-1.843063	-1.409791
H	3.792351	-2.151952	-0.134860	H	-3.926272	-2.277349	-0.687486
C	7.083267	-1.362528	0.208717	C	-6.695479	-0.994308	-2.173917
H	7.834964	0.590019	0.726852	H	-6.724502	0.756316	-3.433217
H	6.028227	-3.174482	-0.286774	H	-6.335135	-2.684183	-0.886905
H	8.066100	-1.815977	0.135542	H	-7.761509	-1.176906	-2.257974
H	0.223620	-0.320654	1.975178	H	0.379568	-2.104707	-1.708864
C	-2.933354	-2.764355	1.035236	C	4.270873	0.965143	0.812966
C	-3.885676	-1.572985	1.220676	C	4.373445	-0.563176	0.954832
H	-3.255548	-3.440852	0.233923	H	4.537955	1.497991	1.728593
H	-4.852926	-1.712967	0.731908	H	4.422957	-0.881781	2.003527
C	-1.838931	-0.785115	0.311738	C	2.186003	-0.011152	0.227178
N	-1.673419	-2.097213	0.645788	N	2.838714	1.144819	0.510961
N	-3.131038	-0.479341	0.581391	N	3.098382	-1.011064	0.359471
C	-3.726795	0.803677	0.362969	C	2.807648	-2.391514	0.119202
C	-3.592969	1.806436	1.337729	C	2.090338	-3.130435	1.075513
C	-4.439839	1.013942	-0.832746	C	3.240087	-2.959055	-1.097345
C	-4.215838	3.038000	1.103615	C	1.786457	-4.465586	0.779909
C	-5.038601	2.261570	-1.031578	C	2.914501	-4.294827	-1.350694
C	-4.933082	3.263558	-0.068010	C	2.188134	-5.040177	-0.421514
H	-4.129095	3.830466	1.840706	H	1.227355	-5.057857	1.497530
H	-5.582580	2.458752	-1.949338	H	3.224968	-4.762452	-2.278186

C	-0.496738	-2.854739	0.338034
C	-0.195459	-3.134999	-1.010513
C	0.326225	-3.307488	1.388589
C	0.983761	-3.834868	-1.290365
C	1.482874	-4.024647	1.062360
C	1.818201	-4.273801	-0.266022
H	1.245064	-4.048164	-2.321981
H	2.137457	-4.375670	1.853265
C	-1.132253	-2.764368	-2.154675
H	-1.949572	-2.158537	-1.750835
C	-0.025454	-3.060534	2.849481
H	-0.805853	-2.289448	2.873311
C	-4.491902	-0.059384	-1.911570
H	-4.245857	-1.018735	-1.440622
C	-2.776597	1.592040	2.603287
H	-2.307043	0.604509	2.536344
H	-5.404121	4.226088	-0.236547
H	2.727219	-4.816980	-0.502339
C	-1.641083	2.625466	2.709451
H	-0.982002	2.382211	3.550222
H	-2.034727	3.635743	2.871074
H	-1.043902	2.631494	1.792137
C	-3.669302	1.610198	3.857615
H	-4.461265	0.855974	3.790904
H	-4.147754	2.588263	3.984852
H	-3.073179	1.407225	4.754313
C	1.166460	-2.545129	3.676303
H	1.935531	-3.317103	3.792139
H	0.828888	-2.266788	4.680798
H	1.633578	-1.670854	3.214580
C	-0.589593	-4.345731	3.490889
H	-1.434780	-4.749341	2.923527
H	-0.920544	-4.150532	4.517036
H	0.183422	-5.122242	3.525396
C	-1.754313	-4.035353	-2.766485
H	-2.262734	-4.635243	-2.003436
H	-0.986908	-4.664582	-3.231795
H	-2.483908	-3.764797	-3.538064
C	-0.440753	-1.914300	-3.231138
H	-1.142598	-1.686439	-4.040652
H	0.419247	-2.433063	-3.669598
H	-0.090513	-0.967233	-2.807948
C	-5.882492	-0.204050	-2.550482
H	-6.655554	-0.359034	-1.790439
H	-5.892482	-1.059974	-3.234131
H	-6.153225	0.682499	-3.134401
C	-3.414483	0.209384	-2.979602
H	-2.418938	0.271920	-2.525011
H	-3.608135	1.157911	-3.493713
H	-3.405801	-0.592862	-3.726601
O	0.737487	1.797726	-1.403311
C	2.253231	3.459825	-0.631771
C	1.167902	4.184133	-0.110566
C	3.544307	4.000752	-0.504720
C	1.367300	5.406862	0.526950
H	0.173955	3.766131	-0.219403
C	3.741977	5.226087	0.132111
H	4.401142	3.476286	-0.914179
C	2.655523	5.932809	0.653363
H	0.516652	5.949442	0.926381
H	4.744643	5.632080	0.218390
H	2.811662	6.884995	1.149239
H	4.024569	1.547665	-1.507331
C	2.720533	-0.057176	-2.271294
C	2.397721	0.267568	-3.737692
H	3.591991	-0.720693	-2.227602
C	2.234317	2.440426	0.452607
C	2.210027	3.126129	-0.775457
C	1.677488	2.988273	1.622210
C	1.615301	4.391306	-0.813684
C	1.095379	4.258132	1.541055
C	1.063113	4.954040	0.334963
H	1.576616	4.937889	-1.751342
H	0.653024	4.700792	2.427865
C	1.661339	-2.532498	2.408253
H	1.894568	-1.462610	2.389760
C	4.006114	-2.122013	-2.118935
H	4.620381	-1.402611	-1.564208
C	1.698414	2.238918	2.945807
H	2.108668	1.240329	2.756216
C	2.784218	2.511723	-2.043944
H	3.237566	1.550893	-1.776786
H	1.940352	-6.074319	-0.636169
H	0.599502	5.933591	0.287603
C	2.451516	-3.169065	3.567923
H	3.531420	-3.054205	3.421637
H	2.236130	-4.240921	3.649037
H	2.178226	-2.696075	4.517722
C	0.144666	-2.653291	2.635930
H	-0.164985	-3.701337	2.727169
H	-0.408020	-2.190903	1.810989
H	-0.138956	-2.134588	3.557952
C	2.620624	2.944902	3.958637
H	2.678328	2.368251	4.888489
H	2.238029	3.942546	4.204013
H	3.634769	3.064565	3.560326
C	0.283199	2.046624	3.517404
H	-0.364093	1.526844	2.803671
H	-0.178526	3.009201	3.768453
H	0.329289	1.452511	4.437465
C	3.046834	-1.300510	-3.001012
H	2.392577	-1.962739	-3.579531
H	3.615031	-0.678894	-3.703187
H	2.416388	-0.647334	-2.392095
C	4.960438	-2.953758	-2.990148
H	4.411362	-3.596237	-3.687495
H	5.612506	-3.587531	-2.380169
H	5.589910	-2.288232	-3.590380
C	1.671965	2.217267	-3.065043
H	2.085556	1.716204	-3.947610
H	1.183170	3.141269	-3.395493
H	0.910956	1.568137	-2.621683
C	3.886613	3.393852	-2.656298
H	4.680002	3.595121	-1.928417
H	3.485618	4.357054	-2.991831
H	4.331812	2.896837	-3.525481
H	-3.666238	1.887725	-0.762281
C	-1.520276	2.408214	-0.777295
C	-1.829374	3.356526	-1.938181
H	-0.658931	1.772437	-1.036742
H	-1.175119	2.984810	0.093555
H	-0.952266	3.962970	-2.186426
H	-2.120486	2.803535	-2.840255
H	-2.651359	4.036142	-1.684276
C	-3.947496	0.179381	1.317999
C	-3.872664	-1.008957	2.059800
C	-5.205786	0.756512	1.091829
C	-5.029456	-1.622084	2.537488
H	-2.894471	-1.438825	2.241969
C	-6.362706	0.144303	1.570502
H	-5.287874	1.684577	0.537499
C	-6.279986	-1.050552	2.288887

H	1.880558	-0.604979	-1.825012	H	-4.957133	-2.546852	3.101096
H	2.221052	-0.651322	-4.308567	H	-7.329334	0.598181	1.379618
H	3.224355	0.807990	-4.212642	H	-7.181834	-1.527841	2.658107
H	1.499145	0.889461	-3.799565	O	-1.570876	0.294035	1.239564
H	-4.063120	-1.337053	2.276857	H	4.883540	1.356350	-0.008849
H	-2.804078	-3.346749	1.948455	H	5.229503	-0.988838	0.427696

**TS5**	**TS7**
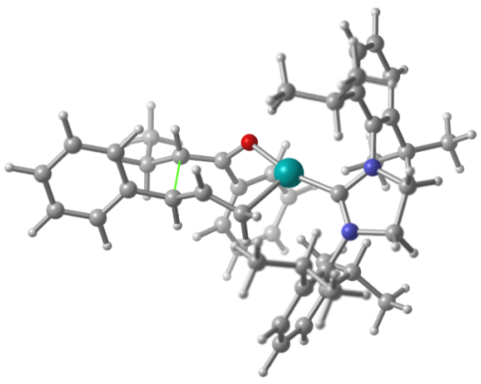	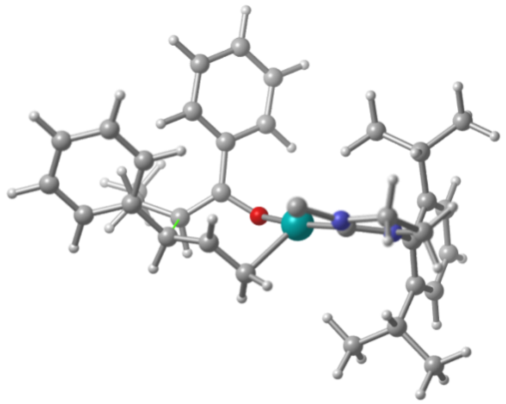
Gas phase energy= -459.529130298	Gas phase energy= -459.525555033
Gas phase free energy correction= 0.865495	Gas phase free energy correction= 0.869088
SMD energy= -2100.62294772	SMD energy= -2100.61694595

C	-3.538268	-1.416249	-0.749392	C	2.786873	-1.455579	-2.257830
C	-2.310795	-1.868730	-0.243362	C	1.883875	-2.029718	-1.335212
C	-3.440967	0.641717	-0.311132	C	2.866803	0.601433	-1.891889
H	-3.523378	0.514672	0.768658	H	2.987373	0.706800	-2.968457
C	-2.232495	1.209864	-0.763039	C	1.676584	1.162056	-1.347045
C	-1.061300	1.345219	0.012725	C	0.403058	1.137895	-1.924868
H	-2.198174	1.582333	-1.785760	H	1.757105	1.622711	-0.365555
H	-1.150648	1.235771	1.096368	H	0.295288	0.750339	-2.938966
Pd	0.244486	-0.320254	-0.458809	Pd	-0.552591	-0.441063	-0.708938
C	-4.720425	1.051619	-0.957795	C	4.124366	0.840772	-1.130913
C	-5.832183	1.368127	-0.162891	C	5.319743	1.147538	-1.798954
C	-4.855100	1.122876	-2.353937	C	4.147677	0.764869	0.271962
C	-7.042326	1.751851	-0.742778	C	6.500557	1.374922	-1.090870
H	-5.743958	1.310740	0.918463	H	5.319867	1.213068	-2.883426
C	-6.063507	1.506395	-2.933684	C	5.325768	0.993758	0.981441
H	-4.013543	0.860971	-2.988790	H	3.238130	0.513181	0.807407
C	-7.163814	1.823175	-2.131527	C	6.509420	1.298648	0.303783
H	-7.889774	1.994188	-0.109735	H	7.413408	1.612230	-1.627433
H	-6.150408	1.552468	-4.014386	H	5.321023	0.924631	2.064983
H	-8.104126	2.118028	-2.585107	H	7.427895	1.472702	0.854425
H	-0.382432	2.138146	-0.286939	H	-0.303693	1.918567	-1.665151
C	3.836842	1.834978	0.769338	C	-2.881391	2.270885	1.779388
C	4.318837	0.997710	-0.427352	C	-3.982105	1.348727	1.230039
H	4.258937	1.492585	1.721885	H	-2.697349	2.117916	2.849523
H	5.154229	0.336781	-0.180935	H	-4.545666	0.836625	2.014526
C	1.997002	0.601161	-0.086751	C	-1.892831	0.704243	0.289941
N	2.377172	1.595571	0.759665	N	-1.694109	1.845297	1.006352
N	3.115894	0.213719	-0.750802	N	-3.201222	0.381598	0.438428
C	3.135274	-0.837415	-1.721933	C	-0.415298	2.438624	1.264846
C	2.836992	-0.541171	-3.063530	C	0.454978	1.819093	2.184099
C	3.437820	-2.144398	-1.293668	C	-0.067389	3.638949	0.612936
C	2.859575	-1.589883	-3.989137	C	1.697921	2.417221	2.422229
C	3.443337	-3.163999	-2.250521	C	1.183685	4.202750	0.885748
C	3.160588	-2.888752	-3.587240	C	2.063813	3.594484	1.776558
H	2.624628	-1.391641	-5.030379	H	2.391359	1.950736	3.115378
H	3.657275	-4.184517	-1.949388	H	1.480112	5.118109	0.384646

C	1.546385	2.173066	1.772959	C	-3.822478	-0.756819	-0.166748
C	1.057514	3.483033	1.598221	C	-4.340574	-0.643709	-1.468035
C	1.249305	1.418380	2.923492	C	-3.887396	-1.958080	0.563606
C	0.248886	4.026207	2.601187	C	-4.946062	-1.770295	-2.035217
C	0.425930	1.997317	3.896690	C	-4.497760	-3.061305	-0.041455
C	-0.071403	3.287741	3.738334	C	-5.025907	-2.967243	-1.328130
H	-0.147696	5.029866	2.486783	H	-5.345230	-1.714747	-3.043293
H	0.172873	1.431220	4.787805	H	-4.552385	-4.006184	0.489180
C	3.686006	-2.457902	0.175980	C	-3.244312	-2.078481	1.939204
H	3.857056	-1.507923	0.696072	H	-3.099139	-1.064338	2.331130
C	2.452906	0.863606	-3.503399	C	-4.195871	0.639822	-2.272361
H	2.525346	1.519600	-2.628498	H	-3.784197	1.407595	-1.607266
C	1.802970	0.017316	3.145143	C	0.090659	0.537024	2.921009
H	2.376098	-0.270894	2.257674	H	-0.880251	0.190601	2.551117
C	1.399287	4.304838	0.362064	C	-1.019674	4.333608	-0.351323
H	1.816566	3.616453	-0.383439	H	-1.798212	3.609392	-0.622144
H	-0.710624	3.719204	4.501250	C	-0.327567	4.789310	-1.649212
H	3.164001	-3.692222	-4.315917	H	0.359443	5.622335	-1.462807
C	0.989232	0.907792	-3.979808	H	-1.076391	5.137214	-2.369234
H	0.703121	1.935871	-4.230381	H	0.244882	3.978394	-2.108271
H	0.317962	0.539154	-3.195495	C	-1.695331	5.542653	0.328883
H	0.846205	0.287495	-4.872426	H	-2.436305	5.994877	-0.339852
C	3.411685	1.405737	-4.578499	H	-0.947335	6.305725	0.574059
H	4.450921	1.377001	-4.232719	H	-2.195555	5.261486	1.261222
H	3.157782	2.442683	-4.825179	C	-5.547485	1.152167	-2.799067
H	3.348099	0.818028	-5.501367	H	-6.266561	1.287407	-1.983840
C	2.465601	5.369145	0.693151	H	-5.984585	0.453719	-3.521588
H	2.065968	6.099898	1.406197	H	-5.414549	2.113919	-3.306807
H	2.766028	5.907566	-0.212719	C	-3.183797	0.444031	-3.416739
H	3.358707	4.924485	1.144330	H	-3.012399	1.390803	-3.941754
C	0.165848	4.978620	-0.266791	H	-3.553542	-0.288627	-4.143848
H	0.441198	5.448605	-1.217262	H	-2.227813	0.081948	-3.022652
H	-0.235154	5.763860	0.383870	C	-1.849626	-2.722293	1.821511
H	-0.635181	4.258632	-0.457906	H	-1.196832	-2.129517	1.166984
C	0.679401	-1.017472	3.306113	H	-1.923436	-3.730717	1.397390
H	0.055400	-0.804285	4.181374	H	-1.368338	-2.794573	2.803611
H	1.100059	-2.020116	3.433027	C	-4.122463	-2.839565	2.945859
H	0.035186	-1.033534	2.420696	H	-5.125734	-2.404849	3.006786
C	2.762745	-0.008558	4.350005	H	-3.667640	-2.799807	3.941674
H	2.233070	0.221395	5.281682	H	-4.225738	-3.896202	2.675644
H	3.568567	0.724794	4.233023	C	-0.048111	0.788721	4.433975
H	3.211158	-1.002926	4.455090	H	-0.788056	1.569966	4.641157
C	2.436979	-3.100212	0.809096	H	0.905802	1.106120	4.870694
H	2.605880	-3.301429	1.873464	H	-0.362214	-0.129841	4.942260
H	2.194413	-4.047618	0.313001	C	1.103424	-0.580823	2.629881
H	1.567309	-2.437704	0.720528	H	0.795426	-1.519033	3.102779
C	4.932490	-3.332765	0.390442	H	2.102343	-0.332284	3.007324
H	4.799701	-4.334270	-0.033406	H	1.176487	-0.759449	1.552253
H	5.126246	-3.453057	1.461966	H	3.038846	4.032225	1.958863
H	5.817307	-2.883951	-0.073276	H	-5.491421	-3.834269	-1.784443
O	-1.235720	-1.657720	-0.958415	H	2.265679	-1.290007	-3.200404
C	-2.116001	-2.471919	1.105939	C	2.229334	-2.718893	-0.063201
C	-1.177966	-3.510036	1.234466	C	1.288137	-3.619404	0.467889
C	-2.794534	-2.034189	2.252154	C	3.417775	-2.499283	0.649715
C	-0.943399	-4.109470	2.468655	C	1.524959	-4.274144	1.672883
H	-0.636540	-3.830269	0.351522	H	0.366735	-3.782188	-0.078395
C	-2.553369	-2.630453	3.491905	C	3.651741	-3.150788	1.861845
H	-3.498678	-1.212694	2.187416	H	4.149210	-1.790909	0.285535
C	-1.633388	-3.673363	3.603953	C	2.708988	-4.040116	2.379185
H	-0.219371	-4.914027	2.547360	H	0.785169	-4.965883	2.063146
H	-3.080774	-2.274497	4.370999	H	4.571955	-2.955646	2.403015
H	-1.451151	-4.139063	4.566763	H	2.893054	-4.545238	3.321652
H	-3.490794	-1.237892	-1.822156	C	4.224434	-1.902535	-2.458907
C	-4.887705	-1.914452	-0.259934	C	4.319175	-3.375019	-2.890742
C	-5.060405	-3.429067	-0.464401	H	4.671128	-1.268661	-3.235808
H	-5.671996	-1.386832	-0.812531	H	4.838185	-1.742312	-1.568858

H -5.051295 -1.671287 0.796012<br>H -6.066022 -3.751164 -0.171544<br>H -4.909110 -3.694925 -1.516454<br>H -4.333530 -3.990635 0.132391<br>H 4.054934 2.898403 0.657758<br>H 4.609516 1.614695 -1.285891	H 5.358384 -3.656045 -3.094951<br>H 3.731041 -3.555212 -3.797463<br>H 3.934631 -4.034033 -2.104951<br>O 0.607749 -1.882012 -1.593750<br>H -3.093209 3.328390 1.614323<br>H -4.693605 1.876911 0.584424
**TS6**	**TS8**
Optimization leads to TS2.	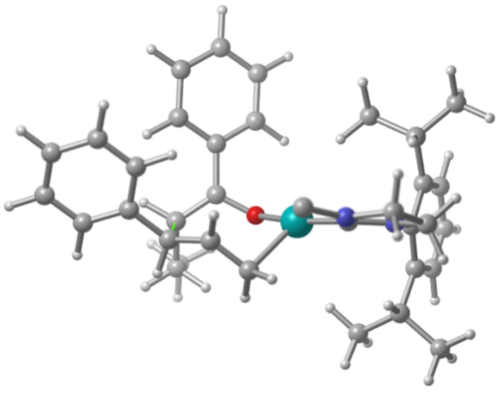
	Gas phase energy= -459.530193342<br>Gas phase free energy correction= 0.869264<br>SMD energy= -2100.62263465
	C 2.823580 -1.788182 -2.008590<br>C 1.846080 -2.216346 -1.085916<br>C 2.993130 0.264163 -1.765912<br>H 3.077939 0.313169 -2.848910<br>C 1.863358 0.943716 -1.233279<br>C 0.600340 1.012163 -1.832473<br>H 1.968703 1.422591 -0.262992<br>H 0.483390 0.633635 -2.849388<br>Pd -0.506092 -0.481138 -0.646363<br>C 4.303701 0.416838 -1.072283<br>C 5.494415 0.354531 -1.814643<br>C 4.397015 0.593940 0.318381<br>C 6.737601 0.474258 -1.193277<br>H 5.439544 0.210692 -2.890675<br>C 5.639316 0.716929 0.940249<br>H 3.495505 0.614238 0.921391<br>C 6.816094 0.658368 0.189252<br>H 7.644424 0.426196 -1.787340<br>H 5.688471 0.850899 2.016310<br>H 7.781547 0.752754 0.675036<br>H -0.040297 1.852576 -1.594505<br>C -2.792830 2.549358 1.498968<br>C -3.903842 1.617678 0.994069<br>H -2.647450 2.483672 2.584223<br>H -4.540729 1.231802 1.794058<br>C -1.805390 0.803020 0.226293<br>N -1.596376 2.018116 0.808327<br>N -3.125142 0.530027 0.376770<br>C -0.315245 2.617455 1.044843<br>C 0.526286 2.080170 2.039251<br>C 0.057266 3.757162 0.302153<br>C 1.763435 2.697338 2.262350<br>C 1.302569 4.339342 0.558942<br>C 2.153062 3.812604 1.527291<br>H 2.431975 2.295017 3.017293<br>H 1.615408 5.208560 -0.010136

	C	-3.751401	-0.682771	-0.052755
	C	-4.203289	-0.799787	-1.377367
	C	-3.889037	-1.727852	0.881024
	C	-4.823552	-1.996005	-1.756254
	C	-4.511516	-2.906841	0.460730
	C	-4.979481	-3.037964	-0.846119
	H	-5.174561	-2.118241	-2.776333
	H	-4.622970	-3.734450	1.153597
	C	-3.304503	-1.607975	2.282719
	H	-3.143247	-0.542901	2.488811
	C	-3.985955	0.308243	-2.396840
	H	-3.506369	1.148563	-1.882121
	C	0.144681	0.863093	2.870350
	H	-0.804515	0.470174	2.492798
	C	-0.866181	4.374291	-0.740503
	H	-1.661447	3.647218	-0.947584
	C	-0.149612	4.676082	-2.069574
	H	0.580810	5.484691	-1.954393
	H	-0.878816	4.996936	-2.821580
	H	0.379238	3.798633	-2.451972
	C	-1.513659	5.663846	-0.193936
	H	-2.243409	6.060625	-0.908583
	H	-0.748871	6.431497	-0.027413
	H	-2.020551	5.493567	0.761459
	C	-5.315435	0.812529	-2.985694
	H	-5.992297	1.159798	-2.197350
	H	-5.828239	0.020896	-3.543930
	H	-5.133506	1.643166	-3.676682
	C	-3.019592	-0.155081	-3.502668
	H	-2.804163	0.669743	-4.191651
	H	-3.451368	-0.979002	-4.083080
	H	-2.076151	-0.501458	-3.065270
	C	-1.924948	-2.290876	2.342675
	H	-1.240621	-1.858722	1.601629
	H	-2.015440	-3.363609	2.133970
	H	-1.473387	-2.168511	3.334166
	C	-4.242162	-2.152007	3.372479
	H	-5.231009	-1.685057	3.314694
	H	-3.820662	-1.950311	4.363297
	H	-4.373525	-3.236333	3.287811
	C	-0.060533	1.239859	4.349141
	H	-0.822495	2.019547	4.460183
	H	0.868933	1.613504	4.794178
	H	-0.378509	0.360648	4.920983
	C	1.184185	-0.257839	2.717602
	H	0.859650	-1.163525	3.239278
	H	2.156961	0.032122	3.131968
	H	1.319908	-0.513126	1.661913
	H	3.121259	4.267793	1.703750
	H	-5.457065	-3.960664	-1.157997
	H	3.851206	-2.069949	-1.783149
	C	2.176404	-2.736148	0.266113
	C	1.180383	-3.427565	0.976569
	C	3.435783	-2.570470	0.863015
	C	1.430124	-3.927352	2.250912
	H	0.211251	-3.558759	0.509782
	C	3.679687	-3.056830	2.148252
	H	4.224174	-2.041628	0.340857
	C	2.680278	-3.736157	2.847670
	H	0.648921	-4.462393	2.781783
	H	4.653824	-2.904047	2.601260
	H	2.873951	-4.117681	3.844809
	C	2.475696	-1.871989	-3.486958
	C	2.350063	-3.324462	-3.970321
	H	1.531278	-1.348406	-3.670784
	H	3.254221	-1.362259	-4.070399

H	2.156153	-3.363027	-5.048162
H	1.524276	-3.824810	-3.455276
H	3.270090	-3.885388	-3.769581
O	0.584951	-2.068883	-1.390110
H	-2.963033	3.594392	1.236389
H	-4.545282	2.092497	0.241897

**TS2a-Me**				**TS4a-Me**			
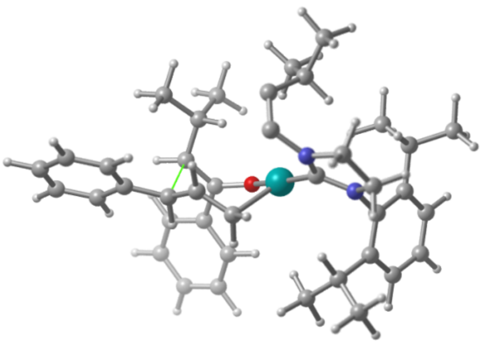				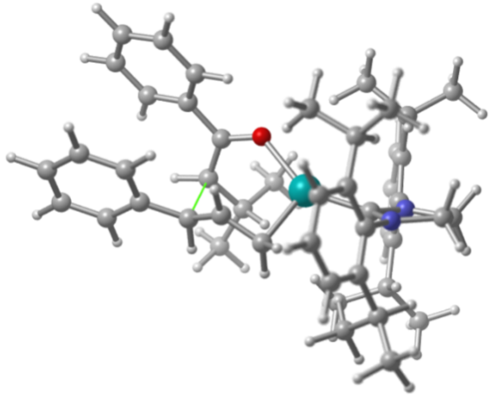			
Gas phase energy=	-466.414582932			Gas phase energy=	-466.417272253		
Gas phase free energy correction=	0.897058			Gas phase free energy correction=	0.895080		
SMD energy=	-2139.94608978			SMD energy=	-2139.94723377		
C	2.946574	0.842850	-1.378103	C	2.689579	-1.585619	-0.882171
C	2.116724	1.965280	-1.166847	C	2.658876	-1.145799	0.453879
C	3.071768	0.243562	0.631086	C	2.638928	0.401403	-1.774258
H	3.078612	1.261526	1.018446	H	2.304439	-0.065861	-2.697783
C	1.910383	-0.495424	0.936678	C	1.692603	1.247457	-1.154097
C	0.726781	0.083243	1.436168	C	0.317070	1.310266	-1.484436
H	1.893813	-1.560713	0.719412	H	2.037939	1.930292	-0.381657
H	0.810609	1.086737	1.860987	H	-0.005258	0.822695	-2.406295
Pd	-0.499258	0.543788	-0.299082	Pd	-0.241894	0.019540	0.116715
C	4.433304	-0.373822	0.698880	C	4.095830	0.702439	-1.751672
C	5.563065	0.449944	0.560630	C	4.936828	0.071592	-2.684128
C	4.638885	-1.739702	0.949634	C	4.680362	1.569421	-0.815219
C	6.852529	-0.074936	0.637810	C	6.314019	0.287722	-2.674911
H	5.422610	1.515690	0.398779	H	4.502331	-0.605698	-3.415617
C	5.928158	-2.267367	1.021484	C	6.057823	1.783088	-0.801649
H	3.788985	-2.397452	1.099425	H	4.066689	2.063903	-0.071856
C	7.041872	-1.440425	0.860908	C	6.882592	1.143144	-1.728127
H	7.708506	0.583142	0.529348	H	6.943053	-0.212251	-3.404429
H	6.063613	-3.327175	1.212016	H	6.486824	2.441310	-0.053832
H	8.043412	-1.852895	0.921744	H	7.954795	1.307892	-1.712625
H	0.052837	-0.565291	1.989314	H	-0.213214	2.235956	-1.270862
C	-3.260133	-2.550574	0.933233	C	-4.465772	-0.344780	0.807409
C	-4.099858	-1.296196	1.215611	C	-4.275167	1.165635	1.019477
H	-3.651779	-3.138373	0.093415	H	-4.818023	-0.861617	1.703411
H	-5.086164	-1.322038	0.746157	H	-4.273499	1.442041	2.081201
C	-2.008591	-0.629726	0.310498	C	-2.229815	0.238759	0.245042
N	-1.951784	-1.970653	0.566220	N	-3.097456	-0.780759	0.468540
N	-3.267109	-0.233749	0.621351	N	-2.933007	1.387543	0.446217
C	-3.770186	1.099053	0.481638	C	-2.388282	2.700068	0.283894
C	-3.548531	2.039253	1.501363	C	-1.556685	3.240339	1.279744
C	-4.499167	1.416928	-0.680615	C	-2.689128	3.399748	-0.902811
C	-4.096820	3.318270	1.347394	C	-0.993680	4.502277	1.050191
C	-5.022946	2.707526	-0.798515	C	-2.108100	4.657630	-1.089075
C	-4.829207	3.649314	0.210906	C	-1.259945	5.200306	-0.123604
H	-3.940469	4.064731	2.120169	H	-0.337554	4.939057	1.796617

H	-5.578609	2.985380	-1.687946
C	-0.851297	-2.804794	0.181893
C	-0.604147	-3.017668	-1.190130
C	-0.048303	-3.398012	1.176376
C	0.516852	-3.773728	-1.548769
C	1.047933	-4.168365	0.769995
C	1.343315	-4.336342	-0.580150
H	0.740540	-3.932474	-2.598770
H	1.684706	-4.629904	1.517981
C	-1.552034	-2.522938	-2.276794
H	-2.304221	-1.875671	-1.814771
C	-0.363545	-3.255784	2.659730
H	-1.078912	-2.430904	2.767979
C	-4.644776	0.408455	-1.812026
H	-4.457600	-0.589556	-1.397575
C	-2.717210	1.709775	2.731986
H	-2.313877	0.699222	2.605313
H	-5.243510	4.646278	0.105148
H	2.211350	-4.914343	-0.878377
C	-1.514997	2.662940	2.853001
H	-0.851728	2.339432	3.662979
H	-1.837495	3.687916	3.070364
H	-0.944074	2.672687	1.918806
C	-3.574612	1.723794	4.010629
H	-4.408004	1.016396	3.936039
H	-3.995759	2.719789	4.190357
H	-2.967238	1.451598	4.881084
C	0.872659	-2.918158	3.512650
H	1.576583	-3.757436	3.544720
H	0.567173	-2.710359	4.544047
H	1.404908	-2.043530	3.128705
C	-1.026466	-4.545162	3.189555
H	-1.906963	-4.823482	2.600783
H	-1.331294	-4.419429	4.234495
H	-0.319900	-5.381842	3.139683
C	-2.292460	-3.715904	-2.914159
H	-2.811725	-4.312710	-2.156010
H	-1.593441	-4.376331	-3.440227
H	-3.031547	-3.357992	-3.639926
C	-0.841094	-1.682304	-3.346097
H	-1.552111	-1.373306	-4.119766
H	-0.037376	-2.243439	-3.836109
H	-0.408943	-0.780794	-2.901669
C	-6.051832	0.393063	-2.430398
H	-6.822030	0.251343	-1.664848
H	-6.131798	-0.422783	-3.157060
H	-6.269363	1.325468	-2.962773
C	-3.568053	0.662414	-2.884291
H	-2.564604	0.635097	-2.444266
H	-3.707240	1.647341	-3.344900
H	-3.624481	-0.097832	-3.671966
O	0.820437	1.850255	-1.181346
C	2.668119	3.258253	-0.678872
C	1.847161	4.094559	0.094000
C	3.985311	3.665346	-0.947374
C	2.337601	5.294989	0.605487
H	0.824563	3.785096	0.277139
C	4.473719	4.868648	-0.438418
H	4.624924	3.053292	-1.575009
C	3.653712	5.684901	0.344884
H	1.693294	5.927199	1.207616
H	5.491738	5.172890	-0.658709
H	4.035990	6.618889	0.743003
H	4.010902	1.035897	-1.495637
C	2.425068	-0.317057	-2.211406
C	2.131015	0.185273	-3.640305
H	-2.307259	5.218409	-1.995282
C	-2.788441	-2.171088	0.331552
C	-2.952195	-2.784031	-0.924437
C	-2.367763	-2.892408	1.463005
C	-2.713839	-4.158536	-1.023336
C	-2.137817	-4.265440	1.319717
C	-2.318266	-4.895309	0.091345
H	-2.828470	-4.656180	-1.981499
H	-1.807923	-4.844960	2.176427
C	-1.275646	2.510126	2.585767
H	-1.716367	1.510008	2.515499
C	-3.578683	2.771518	-1.972667
H	-4.311753	2.133620	-1.464633
C	-2.153236	-2.217501	2.809569
H	-2.355361	-1.147844	2.682353
C	-3.324083	-1.976041	-2.159614
H	-3.609001	-0.969526	-1.832487
H	-0.807453	6.172263	-0.288620
H	-2.134756	-5.960264	-0.002715
C	-1.943410	3.240762	3.766890
H	-3.020626	3.357342	3.603980
H	-1.514457	4.240704	3.901348
H	-1.791114	2.680815	4.696307
C	0.230612	2.317636	2.831655
H	0.741880	3.278934	2.961607
H	0.692748	1.782102	1.995190
H	0.390144	1.726086	3.739886
C	-3.132398	-2.761152	3.866857
H	-3.001801	-2.228998	4.815693
H	-2.957337	-3.826957	4.053605
H	-4.173316	-2.644147	3.544509
C	-0.693104	-2.350698	3.278267
H	0.002711	-1.934222	2.542486
H	-0.427331	-3.400952	3.448330
H	-0.551592	-1.814365	4.223629
C	-2.763222	1.854723	-2.904237
H	-1.984692	2.425832	-3.422993
H	-3.417822	1.401022	-3.657838
H	-2.278413	1.052969	-2.340258
C	-4.368641	3.807477	-2.787601
H	-3.712395	4.395652	-3.438416
H	-4.915002	4.498217	-2.137050
H	-5.091694	3.296992	-3.432672
C	-2.100179	-1.820303	-3.080781
H	-2.350343	-1.206381	-3.953666
H	-1.751857	-2.797452	-3.436450
H	-1.278164	-1.339383	-2.540883
C	-4.522984	-2.575202	-2.913778
H	-5.385725	-2.696902	-2.250007
H	-4.283081	-3.556341	-3.338535
H	-4.813537	-1.918847	-3.741698
H	3.656090	-1.875443	-1.286953
C	1.496375	-2.268674	-1.525786
C	1.823778	-2.744577	-2.948904
H	0.668643	-1.543892	-1.579526
H	0.939357	-3.194612	-3.413299
H	2.160991	-1.927800	-3.596890
H	2.616086	-3.503530	-2.925523
C	3.909255	-0.817795	1.191617
C	3.848344	0.122798	2.230737
C	5.151779	-1.375904	0.856315
C	5.006508	0.522470	2.894763
H	2.880971	0.535185	2.493161
C	6.309525	-0.978043	1.521841
H	5.221411	-2.117637	0.068736
C	6.242812	-0.021683	2.537558

H 1.481799 -0.662418 -1.765836	H 4.946240 1.259798 3.688922
H 1.764062 -0.632613 -4.271089	H 7.264835 -1.409806 1.243226
H 3.044329 0.582115 -4.100780	H 7.146156 0.291246 3.050869
H 1.376476 0.977495 -3.627074	O 1.538974 -0.785683 1.000566
C 3.389646 -1.508443 -2.261192	C 0.989033 -3.450622 -0.678908
H 3.470040 -2.009181 -1.294892	H 1.766875 -4.218014 -0.580773
H 3.032682 -2.245932 -2.989618	H 0.699648 -3.116453 0.321037
H 4.396752 -1.193046 -2.561888	H 0.110814 -3.905971 -1.149377
H -4.232031 -1.110418 2.288318	H -5.151152 -0.575510 -0.017495
H -3.172215 -3.205259 1.801363	H -5.028055 1.771006 0.510445
**TS2b-Me**	**TS4b-Me**
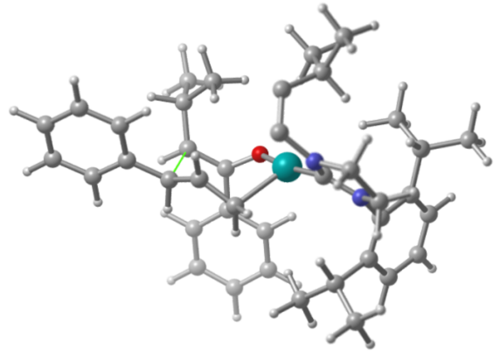	Optimization leads to TS4a-Me.
Gas phase energy= -466.411246510<br>Gas phase free energy correction= 0.896751<br>SMD energy= -2139.94285643	
C 3.089745 1.633840 -1.286003	
C 1.808099 2.216402 -1.236852	
C 3.145520 0.757726 0.606698	
H 3.086541 1.740505 1.073873	
C 2.054818 -0.098667 0.884639	
C 0.781285 0.344183 1.290642	
H 2.193516 -1.172082 0.779854	
H 0.687102 1.394614 1.574488	
Pd -0.432905 0.281944 -0.523464	
C 4.531080 0.208947 0.593125	
C 5.603276 1.053426 0.922691	
C 4.816214 -1.122141 0.244803	
C 6.917790 0.586225 0.913675	
H 5.398531 2.086883 1.191245	
C 6.129714 -1.590088 0.237609	
H 4.011778 -1.793644 -0.037662	
C 7.187431 -0.740335 0.571831	
H 7.729790 1.256663 1.175694	
H 6.329046 -2.621530 -0.034943	
H 8.208220 -1.107624 0.563236	
H 0.194576 -0.326817 1.912068	
C -2.859064 -2.873595 1.170280	
C -3.835634 -1.689678 1.279815	
H -3.181192 -3.618701 0.432584	
H -4.797030 -1.877377 0.795076	
C -1.813821 -0.929315 0.294267	
N -1.621786 -2.214723 0.704941	
N -3.101420 -0.620208 0.578059	
C -3.702068 0.652891 0.316816	
C -3.591117 1.677906 1.272217	
C -4.386119 0.836779 -0.899105	
C -4.200410 2.906492 0.992817	

C	-4.970859	2.083346	-1.144089
C	-4.883252	3.108375	-0.203936
H	-4.131762	3.714585	1.714590
H	-5.490692	2.259965	-2.080017
C	-0.423686	-2.953218	0.432784
C	-0.146219	-3.333542	-0.895294
C	0.453131	-3.266554	1.490918
C	1.068210	-3.977113	-1.157051
C	1.648502	-3.926631	1.184760
C	1.963385	-4.262464	-0.129999
H	1.314432	-4.258628	-2.175933
H	2.347272	-4.167971	1.978799
C	-1.138982	-3.116233	-2.031261
H	-1.991622	-2.549510	-1.641781
C	0.108628	-2.947245	2.939754
H	-0.700455	-2.205789	2.929977
C	-4.430623	-0.262931	-1.950396
H	-4.158111	-1.204607	-1.458891
C	-2.826137	1.481530	2.572322
H	-2.305046	0.519531	2.511139
H	-5.343958	4.069076	-0.407847
H	2.902763	-4.757921	-0.351368
C	-1.753393	2.566783	2.763400
H	-1.119575	2.327372	3.624363
H	-2.204250	3.549629	2.943417
H	-1.118098	2.643209	1.876751
C	-3.784299	1.431933	3.777578
H	-4.540265	0.648156	3.655630
H	-4.309756	2.387016	3.894549
H	-3.228644	1.236403	4.701487
C	1.284973	-2.345756	3.728882
H	2.076951	-3.085726	3.890158
H	0.939577	-2.017994	4.715630
H	1.724076	-1.486203	3.215257
C	-0.403839	-4.215352	3.655415
H	-1.240168	-4.677898	3.120771
H	-0.730490	-3.977179	4.673902
H	0.396486	-4.961641	3.720821
C	-1.674055	-4.469427	-2.540009
H	-2.115283	-5.053388	-1.724656
H	-0.870955	-5.067806	-2.985304
H	-2.440597	-4.309981	-3.306695
C	-0.541519	-2.289162	-3.178640
H	-1.281047	-2.158315	-3.976098
H	0.341449	-2.772174	-3.611939
H	-0.243458	-1.299650	-2.818961
C	-5.829304	-0.447927	-2.561520
H	-6.585418	-0.605032	-1.785036
H	-5.833876	-1.315878	-3.229972
H	-6.128542	0.423125	-3.154895
C	-3.377062	0.001895	-3.042165
H	-2.375463	0.088722	-2.605241
H	-3.594531	0.936873	-3.571525
H	-3.367510	-0.814181	-3.773585
O	0.759636	1.544341	-1.619187
C	1.558274	3.503865	-0.523653
C	0.227612	3.892619	-0.287895
C	2.586912	4.337245	-0.049611
C	-0.065569	5.065843	0.402831
H	-0.569324	3.253291	-0.649826
C	2.292412	5.511210	0.645075
H	3.626571	4.085943	-0.229350
C	0.965788	5.880361	0.876767
H	-1.101684	5.338733	0.575611
H	3.101140	6.140955	1.001443
H	0.739629	6.793628	1.416951

H	3.914247	2.311176	-1.065843
C	3.510473	0.603380	-2.327375
C	2.568492	-0.589302	-2.539331
H	4.478418	0.208244	-1.991438
H	3.077281	-1.360662	-3.131165
H	1.670831	-0.273245	-3.073516
H	2.244512	-1.036899	-1.595743
H	-4.024038	-1.396376	2.319136
H	-2.700964	-3.378291	2.124504
C	3.758049	1.322835	-3.670682
H	4.485800	2.134672	-3.560530
H	4.143448	0.617362	-4.416543
H	2.822085	1.748553	-4.050161

**TS2c-Me**	**TS4c-Me**
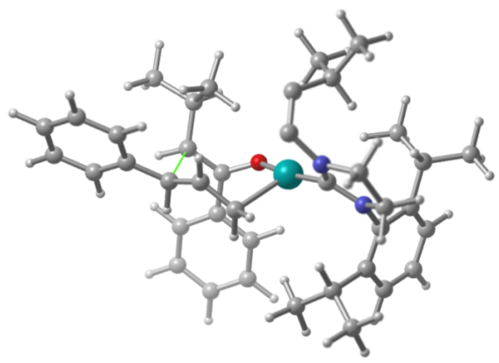	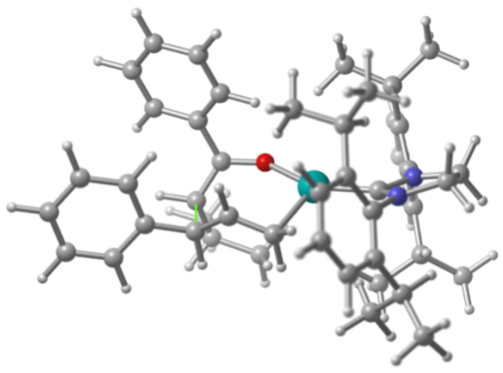
Gas phase energy= -466.409914631<br>Gas phase free energy correction= 0.897083<br>SMD energy= -2139.94121615	Gas phase energy= -466.407528073<br>Gas phase free energy correction= 0.894563<br>SMD energy= -2139.93931902

C	3.035935	1.374067	-1.443761	C	3.171293	-1.460315	-1.313034
C	1.835589	2.110983	-1.366389	C	2.553230	-1.598245	-0.054771
C	3.134370	0.719884	0.531185	C	2.950637	0.613981	-1.575705
H	3.078942	1.746304	0.892701	H	2.658803	0.456631	-2.613480
C	2.033128	-0.090886	0.893988	C	1.968282	1.257594	-0.780406
C	0.775952	0.407746	1.276070	C	0.608427	1.397890	-1.113286
H	2.136449	-1.170974	0.838411	H	2.291197	1.697488	0.161528
H	0.707353	1.472727	1.507947	H	0.314900	1.185763	-2.143371
Pd	-0.488684	0.333887	-0.530819	Pd	-0.313728	-0.276371	-0.074710
C	4.531660	0.188719	0.588882	C	4.386349	0.988929	-1.418752
C	5.599013	1.102903	0.577784	C	5.217206	1.002662	-2.551267
C	4.838704	-1.179016	0.666554	C	4.954232	1.299382	-0.171918
C	6.923782	0.671086	0.603942	C	6.572765	1.315675	-2.445052
H	5.382237	2.167764	0.546610	H	4.791609	0.766196	-3.523129
C	6.164407	-1.613825	0.691496	C	6.309888	1.607968	-0.065786
H	4.043997	-1.915533	0.712137	H	4.346285	1.274685	0.726516
C	7.214183	-0.694175	0.651622	C	7.126679	1.618288	-1.199460
H	7.728342	1.398843	0.588725	H	7.195104	1.322971	-3.334171
H	6.376695	-2.676688	0.745953	H	6.730300	1.830155	0.909478
H	8.243534	-1.036167	0.667648	H	8.181523	1.856481	-1.111827
H	0.165531	-0.220649	1.918710	H	0.076475	2.235172	-0.671157
C	-2.961470	-2.757819	1.200605	C	-4.470787	-0.109537	0.892852
C	-3.902733	-1.548988	1.336161	C	-4.160944	1.382935	1.095914
H	-3.315309	-3.486863	0.461375	H	-4.805356	-0.605210	1.807905
H	-4.879508	-1.708945	0.872710	H	-4.128197	1.665431	2.155029
C	-1.879175	-0.836798	0.319771	C	-2.219401	0.312977	0.235110
N	-1.713816	-2.128306	0.721334	N	-3.161908	-0.637883	0.470749
N	-3.155176	-0.497393	0.621868	N	-2.811026	1.505771	0.507375
C	-3.740236	0.780718	0.351509	C	-2.179552	2.782424	0.393523

C	-3.590255	1.822246	1.282940	C	-1.352282	3.243442	1.431229
C	-4.454925	0.950007	-0.849251	C	-2.407680	3.536313	-0.774751
C	-4.197214	3.051088	0.999194	C	-0.730752	4.488380	1.271914
C	-5.035545	2.197235	-1.099848	C	-1.765924	4.772413	-0.894228
C	-4.913463	3.237477	-0.180215	C	-0.931610	5.242784	0.119853
H	-4.100476	3.871377	1.703791	H	-0.075922	4.864267	2.051951
H	-5.578926	2.362973	-2.024224	H	-1.907891	5.372213	-1.786257
C	-0.546455	-2.903794	0.420541	C	-2.960709	-2.037755	0.248095
C	-0.309057	-3.292839	-0.913617	C	-3.207272	-2.564022	-1.033491
C	0.334073	-3.257072	1.462570	C	-2.517697	-2.843543	1.312359
C	0.860821	-4.007203	-1.192578	C	-3.014769	-3.934949	-1.230128
C	1.488610	-3.977910	1.136701	C	-2.345338	-4.211148	1.074745
C	1.758116	-4.337813	-0.181564	C	-2.593044	-4.753085	-0.183844
H	1.075235	-4.302591	-2.214514	H	-3.182841	-4.365981	-2.212337
H	2.186157	-4.256770	1.920031	H	-1.996801	-4.854546	1.876541
C	-1.301616	-3.012983	-2.037325	C	-1.107764	2.423422	2.689047
H	-2.090897	-2.361324	-1.648728	H	-1.619859	1.462644	2.569094
C	0.031509	-2.921040	2.917131	C	-3.277155	2.982041	-1.897642
H	-0.752140	-2.152938	2.920993	H	-4.026736	2.322293	-1.443643
C	-4.532653	-0.166850	-1.880981	C	-2.195284	-2.256771	2.678548
H	-4.281681	-1.107113	-1.375319	H	-2.322552	-1.170383	2.609370
C	-2.784104	1.643767	2.560402	C	-3.615390	-1.671656	-2.197306
H	-2.273713	0.676176	2.499937	H	-3.790377	-0.663303	-1.804542
H	-5.372438	4.198254	-0.387633	H	-0.434608	6.200226	0.007169
H	2.664914	-4.883425	-0.419869	H	-2.444245	-5.814028	-0.353984
C	-1.696091	2.723066	2.693636	C	-1.690337	3.122477	3.931043
H	-1.033835	2.493817	3.535637	H	-2.761577	3.317411	3.809621
H	-2.132757	3.712810	2.871052	H	-1.193392	4.082725	4.111744
H	-1.091205	2.775520	1.783843	H	-1.550705	2.497870	4.820459
C	-3.700888	1.625244	3.798099	C	0.386688	2.110062	2.870806
H	-4.464698	0.843907	3.716326	H	0.968382	3.018502	3.066549
H	-4.216499	2.585821	3.913999	H	0.784741	1.629071	1.972116
H	-3.115760	1.443467	4.706510	H	0.530734	1.428137	3.716909
C	1.243545	-2.356182	3.678702	C	-3.161361	-2.780747	3.756733
H	2.023632	-3.115016	3.807545	H	-2.950314	-2.306140	4.721657
H	0.934530	-2.034098	4.679314	H	-3.054495	-3.864224	3.884473
H	1.685988	-1.498716	3.164683	H	-4.204804	-2.575873	3.491757
C	-0.506809	-4.168739	3.649435	C	-0.728710	-2.521261	3.064948
H	-1.366507	-4.608076	3.132714	H	-0.048756	-2.207054	2.266729
H	-0.807256	-3.915195	4.672275	H	-0.554335	-3.587332	3.252613
H	0.271132	-4.939138	3.705091	H	-0.473174	-1.975320	3.980521
C	-1.967410	-4.325897	-2.495912	C	-2.439799	2.112161	-2.854725
H	-2.447451	-4.840920	-1.656167	H	-1.643588	2.706978	-3.317451
H	-1.229021	-5.009806	-2.930044	H	-3.073574	1.705130	-3.651248
H	-2.728211	-4.120363	-3.257358	H	-1.977418	1.276394	-2.321152
C	-0.661715	-2.275773	-3.224747	C	-4.034670	4.072286	-2.671102
H	-1.410843	-2.105258	-4.005678	H	-3.356215	4.686979	-3.272929
H	0.157387	-2.853795	-3.667069	H	-4.587827	4.733299	-1.995745
H	-0.263788	-1.304208	-2.914712	H	-4.748633	3.609436	-3.360771
C	-5.937415	-0.329085	-2.484175	C	-2.470264	-1.562702	-3.219882
H	-6.694922	-0.450759	-1.702701	H	-2.746081	-0.882126	-4.033467
H	-5.965410	-1.210913	-3.133579	H	-2.237745	-2.541325	-3.655770
H	-6.216705	0.535831	-3.095915	H	-1.564783	-1.181114	-2.737156
C	-3.477150	0.053171	-2.981162	C	-4.919370	-2.147694	-2.860963
H	-2.470812	0.117637	-2.550681	H	-5.728957	-2.230813	-2.127733
H	-3.671248	0.986955	-3.521466	H	-4.791714	-3.127744	-3.334378
H	-3.494759	-0.772621	-3.701652	H	-5.227987	-1.440676	-3.639126
O	0.709916	1.537617	-1.676903	H	4.259039	-1.457600	-1.285286
C	1.761437	3.450069	-0.720620	C	2.659254	-2.092592	-2.603326
C	0.501628	3.954310	-0.353532	C	3.340115	-1.358841	1.185935
C	2.899692	4.222349	-0.430492	C	2.710883	-0.724877	2.268275
C	0.384731	5.182117	0.293995	C	4.696024	-1.702890	1.299559
H	-0.378604	3.364595	-0.583528	C	3.426965	-0.409433	3.422040
C	2.781818	5.451037	0.219490	H	1.660052	-0.476360	2.183579
H	3.884956	3.877480	-0.725717	C	5.410053	-1.397369	2.457098
C	1.524585	5.934878	0.587927	H	5.195652	-2.211619	0.482303

H	-0.597736	5.549509	0.572319	C	4.780792	-0.740883	3.518542
H	3.672467	6.032378	0.434549	H	2.929608	0.095095	4.244402
H	1.434170	6.889940	1.094400	H	6.458220	-1.667215	2.530706
H	3.952630	1.950798	-1.345887	H	5.339741	-0.496542	4.415819
C	3.153205	0.238144	-2.468004	O	1.265701	-1.641419	0.058219
C	2.595644	-1.131696	-2.050655	C	3.057278	-3.583662	-2.616390
H	2.546972	0.561137	-3.327572	H	4.133632	-3.711171	-2.455580
H	2.522995	-1.780018	-2.932202	H	2.526094	-4.118511	-1.820614
H	1.600776	-1.045201	-1.608620	H	2.796807	-4.046027	-3.575772
H	3.249452	-1.630535	-1.330587	H	-5.219784	-0.285797	0.111440
H	-4.059615	-1.254322	2.380396	H	-4.868187	2.043389	0.588517
H	-2.802120	-3.275381	2.147649	H	3.210355	-1.601441	-3.421838
C	4.610631	0.092796	-2.941333	C	1.160181	-1.949522	-2.900421
H	4.681637	-0.661418	-3.733350	H	0.836370	-0.905498	-2.869490
H	4.996374	1.038589	-3.339037	H	0.944420	-2.342945	-3.901463
H	5.261112	-0.221063	-2.116699	H	0.559670	-2.497834	-2.170784

**TS2a-Ph**	**TS4a-Ph**
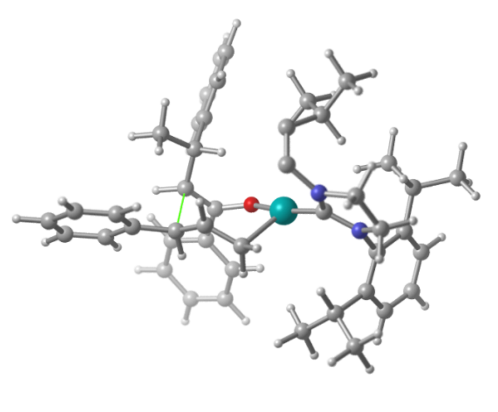	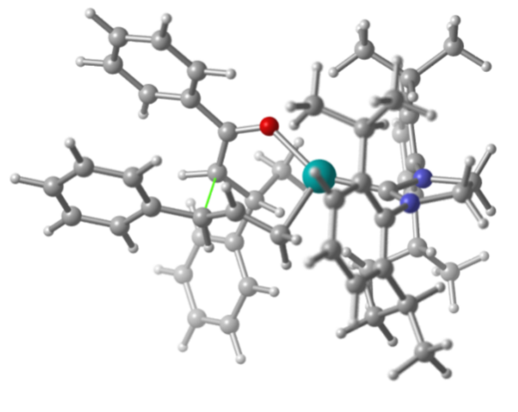
Gas phase energy= -495.959478103	Gas phase energy= -495.971169104
Gas phase free energy correction= 0.945066	Gas phase free energy correction= 0.944111
SMD energy= -2331.68111117	SMD energy= -2331.6901434

C	3.024910	-0.630254	0.514988	C	-2.778342	0.890808	0.440035
C	2.149692	-1.729312	0.637245	C	-2.406625	-0.119854	1.344052
C	2.674583	-0.274355	-1.532694	C	-2.605450	-0.283417	-1.361202
H	2.550167	-1.328421	-1.778951	H	-2.516188	0.647129	-1.917416
C	1.497224	0.491692	-1.646851	C	-1.451064	-1.100073	-1.360695
C	0.220194	-0.069962	-1.856642	C	-0.162037	-0.685332	-1.768385
H	1.554298	1.569664	-1.510373	H	-1.551275	-2.134960	-1.041180
H	0.190121	-1.089651	-2.247697	H	-0.080702	0.261500	-2.305389
Pd	-0.635326	-0.455991	0.101334	Pd	0.532212	-0.288185	0.220787
C	4.002539	0.237452	-1.996541	C	-3.978016	-0.857019	-1.402307
C	5.108356	-0.629324	-1.995531	C	-5.043581	-0.025271	-1.788758
C	4.182081	1.536312	-2.496880	C	-4.265436	-2.183171	-1.045881
C	6.358372	-0.203449	-2.442600	C	-6.354000	-0.499919	-1.807690
H	4.979527	-1.650730	-1.646646	H	-4.836419	1.006708	-2.063244
C	5.433233	1.966144	-2.939314	C	-5.576610	-2.657526	-1.061788
H	3.337534	2.215150	-2.552837	H	-3.471068	-2.848597	-0.728849
C	6.529453	1.101796	-2.909079	C	-6.627229	-1.819941	-1.439934
H	7.196979	-0.891952	-2.431599	H	-7.161096	0.160042	-2.109097
H	5.549688	2.976062	-3.319082	H	-5.776533	-3.681580	-0.765091
H	7.500887	1.436960	-3.256738	H	-7.646763	-2.191113	-1.448662
H	-0.529078	0.583069	-2.296891	H	0.527737	-1.448029	-2.123036
C	-3.727177	2.383280	-0.871533	C	4.721101	0.445085	0.769154
C	-4.498261	1.071311	-1.084979	C	4.791503	-0.861622	-0.039532
H	-4.081917	2.941646	0.003839	H	5.129615	0.344667	1.777943
H	-5.437786	1.026897	-0.528848	H	5.036189	-1.729684	0.584810

C	-2.295462	0.555570	-0.374019
N	-2.354189	1.895673	-0.629234
N	-3.542936	0.068982	-0.574825
C	-3.943577	-1.289354	-0.365254
C	-3.753477	-2.240452	-1.381832
C	-4.542747	-1.622632	0.864576
C	-4.207350	-3.545240	-1.154860
C	-4.972630	-2.939413	1.054307
C	-4.813904	-3.891813	0.049344
H	-4.076630	-4.299460	-1.924583
H	-5.426690	-3.227923	1.996616
C	-1.269213	2.786710	-0.338679
C	-0.905464	2.997108	1.007529
C	-0.585364	3.416592	-1.397408
C	0.211041	3.798334	1.269920
C	0.519779	4.217545	-1.086145
C	0.928222	4.390817	0.233851
H	0.516633	3.967552	2.297568
H	1.068752	4.707957	-1.883662
C	-1.723099	2.439481	2.166893
H	-2.435730	1.710857	1.769054
C	-1.059928	3.296882	-2.840261
H	-1.743529	2.440353	-2.893126
C	-4.650342	-0.601272	1.988337
H	-4.516541	0.395378	1.551388
C	-3.065109	-1.889288	-2.691765
H	-2.643663	-0.883511	-2.589907
H	-5.155019	-4.908940	0.210133
H	1.798731	4.998270	0.456795
C	-1.893757	-2.843849	-2.981359
H	-1.327075	-2.495576	-3.852005
H	-2.246277	-3.858949	-3.197365
H	-1.216464	-2.891779	-2.122591
C	-4.067665	-1.871248	-3.860931
H	-4.880690	-1.160450	-3.675788
H	-4.515610	-2.861294	-4.006121
H	-3.564789	-1.586253	-4.791826
C	0.075057	3.046753	-3.848267
H	0.757415	3.902359	-3.904795
H	-0.346094	2.899425	-4.848875
H	0.659721	2.159173	-3.591087
C	-1.843431	4.565889	-3.240408
H	-2.647199	4.787460	-2.530371
H	-2.280925	4.450396	-4.238379
H	-1.173981	5.433777	-3.259669
C	-2.535793	3.568342	2.832343
H	-3.173934	4.083221	2.105295
H	-1.870128	4.314754	3.281384
H	-3.172918	3.160027	3.625077
C	-0.863611	1.701977	3.202731
H	-1.503589	1.236231	3.959348
H	-0.186045	2.384424	3.725679
H	-0.262267	0.915134	2.735011
C	-6.020461	-0.617762	2.685247
H	-6.834549	-0.497053	1.962568
H	-6.080032	0.198234	3.413671
H	-6.186046	-1.554185	3.229027
C	-3.506178	-0.809236	2.998586
H	-2.530522	-0.750381	2.502545
H	-3.584807	-1.793657	3.474189
H	-3.544621	-0.044440	3.782895
O	0.894866	-1.553738	0.917379
C	2.562424	-3.101613	0.233580
C	1.576037	-4.004193	-0.193931
C	3.900926	-3.526466	0.261021
C	1.921400	-5.290248	-0.605435
C	2.529329	-0.139114	0.049672
N	3.268417	0.693810	0.826237
N	3.410111	-0.987928	-0.545755
C	3.030876	-2.047186	-1.428713
C	2.490085	-3.232432	-0.900600
C	3.189679	-1.847819	-2.814762
C	2.079359	-4.223519	-1.801161
C	2.769869	-2.864796	-3.677309
C	2.212525	-4.040411	-3.174098
H	1.647932	-5.144705	-1.422077
H	2.868772	-2.740304	-4.749613
C	2.723583	1.729576	1.650968
C	2.563734	3.018142	1.109283
C	2.374253	1.429397	2.980167
C	2.048333	4.020664	1.936727
C	1.862743	2.462341	3.773722
C	1.703920	3.746104	3.258700
H	1.900748	5.021019	1.542445
H	1.579468	2.257573	4.801484
C	2.351246	-3.464201	0.597915
H	2.646111	-2.542318	1.110555
C	3.746407	-0.532094	-3.350220
H	4.476387	-0.158482	-2.622005
C	2.538512	0.030950	3.557621
H	2.870915	-0.628392	2.747636
C	2.868243	3.300335	-0.355371
H	3.479176	2.473994	-0.738234
H	1.881195	-4.814668	-3.857649
H	1.299842	4.533279	3.886172
C	3.293987	-4.589366	1.065489
H	4.336039	-4.366554	0.810309
H	3.030840	-5.543961	0.595315
H	3.222902	-4.717174	2.151369
C	0.895871	-3.752666	1.005129
H	0.527813	-4.681508	0.553176
H	0.240983	-2.928812	0.699454
H	0.823324	-3.854692	2.093699
C	3.620378	0.011558	4.654374
H	3.772277	-1.009426	5.022122
H	3.324127	0.635336	5.505728
H	4.577448	0.391385	4.278904
C	1.205418	-0.532023	4.081723
H	0.431761	-0.516970	3.307284
H	0.842113	0.045906	4.939925
H	1.343197	-1.567533	4.414247
C	2.636767	0.532010	-3.456778
H	1.856427	0.202477	-4.152531
H	3.050794	1.477731	-3.825634
H	2.172489	0.713181	-2.483386
C	4.479099	-0.685077	-4.692208
H	3.782670	-0.909177	-5.507865
H	5.229476	-1.481467	-4.652474
H	4.985271	0.252274	-4.946347
C	1.560740	3.306318	-1.167459
H	1.764448	3.469236	-2.232243
H	0.885178	4.097793	-0.822105
H	1.047138	2.346122	-1.050797
C	3.662300	4.600195	-0.561593
H	4.579251	4.605203	0.037213
H	3.072562	5.481577	-0.286530
H	3.937136	4.707436	-1.616669
H	-3.838768	1.100548	0.338062
C	-1.851774	2.056013	0.129953
H	-0.928109	1.646518	-0.306287
C	-3.409252	-1.074405	1.888483
C	-2.983658	-2.364981	2.237701

H	0.542517	-3.676793	-0.188855	C	-4.767943	-0.750792	2.017906
C	4.244991	-4.815326	-0.146920	C	-3.900130	-3.319470	2.674726
H	4.676677	-2.858616	0.622209	H	-1.929195	-2.599842	2.148569
C	3.257244	-5.699816	-0.586833	C	-5.683462	-1.704194	2.458784
H	1.148575	-5.974960	-0.938721	H	-5.117435	0.244748	1.768176
H	5.282245	-5.132186	-0.113001	C	-5.254707	-2.993953	2.781190
H	3.525699	-6.701711	-0.904621	H	-3.559502	-4.317672	2.930783
H	4.082479	-0.855111	0.399465	H	-6.732968	-1.443745	2.544056
C	2.740172	0.642767	1.293460	H	-5.969847	-3.736828	3.118833
H	1.690562	0.922093	1.114274	O	-1.156125	-0.404641	1.528380
C	3.629567	1.816411	0.836881	C	-1.427726	2.814713	1.405535
H	3.324181	2.184712	-0.143435	H	-2.298693	3.223246	1.930504
H	3.559976	2.653229	1.537856	H	-0.893688	2.140003	2.081265
H	4.680898	1.512673	0.775400	H	-0.758918	3.642833	1.149457
H	-4.711952	0.877763	-2.142735	H	5.229505	1.280083	0.272913
H	-3.765236	3.041789	-1.740417	H	5.510542	-0.819974	-0.860613
C	2.898893	0.440990	2.808950	C	-2.473250	2.994570	-0.891853
C	2.522849	1.487212	3.666142	C	-1.863066	3.211978	-2.133866
C	3.423059	-0.720458	3.387800	C	-3.677487	3.660103	-0.616588
C	2.651838	1.377908	5.049050	C	-2.436980	4.067188	-3.078062
H	2.113163	2.397213	3.237242	H	-0.931792	2.702366	-2.363875
C	3.559756	-0.834966	4.774646	C	-4.254939	4.514202	-1.556747
H	3.715622	-1.555329	2.761574	H	-4.166234	3.508616	0.341958
C	3.174715	0.210728	5.612261	C	-3.636346	4.720779	-2.793098
H	2.343043	2.199887	5.687541	H	-1.948938	4.218321	-4.035352
H	3.965789	-1.748333	5.197218	H	-5.187371	5.019166	-1.326153
H	3.278772	0.118548	6.688318	H	-4.085243	5.384293	-3.524696

TS2b-Ph

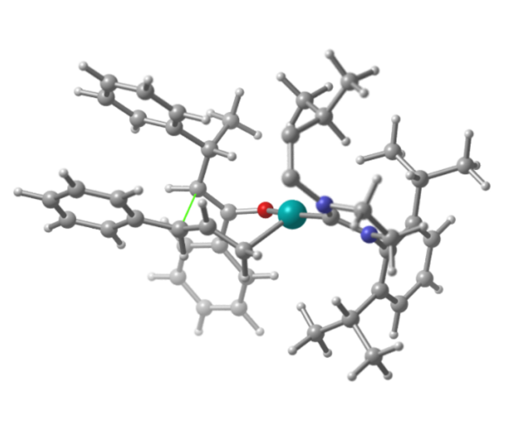

Gas phase energy= -495.970372296
Gas phase free energy correction= 0.945457
SMD energy= -2331.68980855

C	2.547613	1.494591	-1.046823
C	1.465003	2.400979	-1.008949
C	2.593653	1.001337	0.971446
H	2.353180	2.008480	1.310312
C	1.585052	0.043349	1.227632
C	0.267653	0.382809	1.588505
H	1.809021	-1.012662	1.086908
H	0.099156	1.400236	1.949796
Pd	-0.865830	0.493253	-0.270318
C	4.043260	0.688193	1.171761
C	4.971538	1.742308	1.141516
C	4.520139	-0.608143	1.412318
C	6.335013	1.506486	1.310737
H	4.613697	2.757402	0.985970
C	5.883456	-0.845981	1.578192

TS4b-Ph

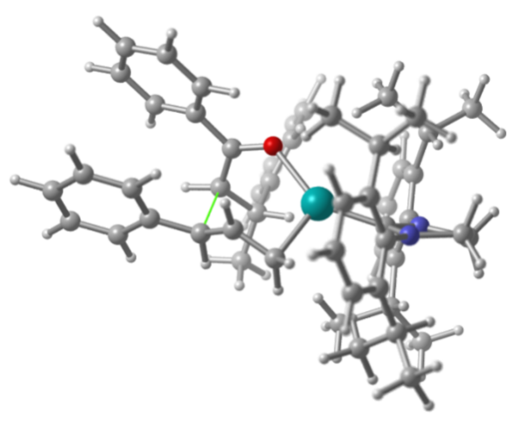

Gas phase energy= -495.966434487
Gas phase free energy correction= 0.944433
SMD energy= -2331.68522243

C	-2.790826	0.500811	-0.734096
C	-2.618085	-0.045893	0.550946
C	-2.172998	-1.373092	-1.756545
H	-1.952809	-0.792828	-2.646799
C	-1.054937	-1.985288	-1.145167
C	0.296341	-1.726059	-1.487855
H	-1.230755	-2.746373	-0.388836
H	0.488616	-1.177964	-2.411300
Pd	0.478126	-0.362522	0.121491
C	-3.509308	-2.023765	-1.797578
C	-4.468905	-1.547762	-2.707724
C	-3.866688	-3.089498	-0.957345
C	-5.745360	-2.103803	-2.766430
H	-4.207613	-0.724626	-3.368632
C	-5.144836	-3.643564	-1.011054

H	3.831133	-1.442985	1.454199	H	-3.156272	-3.479020	-0.238271
C	6.798912	0.206417	1.522505	C	-6.091522	-3.153506	-1.912095
H	7.033918	2.336371	1.282074	H	-6.469422	-1.718771	-3.477253
H	6.231032	-1.860429	1.741729	H	-5.403808	-4.451616	-0.335502
H	7.859555	0.017257	1.650584	H	-7.086037	-3.585150	-1.950242
H	-0.313592	-0.367063	2.114974	H	1.031526	-2.502356	-1.281343
C	-3.405739	-2.756159	1.090149	C	4.423289	1.303639	0.562609
C	-4.384915	-1.573879	1.080239	C	4.700220	-0.171076	0.895218
H	-3.635326	-3.501763	0.318685	H	4.665599	1.982447	1.383651
H	-5.289232	-1.767146	0.497555	H	4.805713	-0.343256	1.973818
C	-2.258442	-0.818658	0.332232	C	2.443870	0.042387	0.194792
N	-2.116534	-2.100955	0.775759	N	2.969804	1.287607	0.316357
N	-3.575679	-0.516196	0.449968	N	3.470938	-0.827028	0.405586
C	-4.166203	0.727266	0.057625	C	3.342926	-2.250061	0.334650
C	-4.187652	1.803772	0.960071	C	2.733044	-2.950739	1.389468
C	-4.726386	0.822734	-1.230399	C	3.816827	-2.901771	-0.822695
C	-4.806406	2.991392	0.553273	C	2.569108	-4.334838	1.248924
C	-5.327007	2.029783	-1.600567	C	3.634276	-4.284398	-0.919683
C	-5.372492	3.103998	-0.713704	C	3.006781	-4.993862	0.104558
H	-4.836275	3.839036	1.231126	H	2.091647	-4.899360	2.043795
H	-5.755708	2.137490	-2.591615	H	3.976678	-4.815538	-1.800674
C	-0.938334	-2.903540	0.624841	C	2.218720	2.491578	0.135805
C	-0.575212	-3.352503	-0.662220	C	2.031095	2.990297	-1.165133
C	-0.214663	-3.290955	1.771581	C	1.675479	3.128483	1.265546
C	0.537430	-4.193442	-0.778343	C	1.246925	4.138410	-1.322998
C	0.896540	-4.125640	1.604701	C	0.916589	4.284790	1.064150
C	1.269302	-4.576528	0.342041	C	0.691157	4.778676	-0.218078
H	0.838102	-4.549847	-1.758729	H	1.062638	4.530782	-2.318767
H	1.470803	-4.431286	2.473328	H	0.460953	4.782847	1.913487
C	-1.358729	-2.981103	-1.915546	C	2.276000	-2.259789	2.666924
H	-2.184335	-2.324009	-1.624394	H	2.398009	-1.179934	2.531397
C	-0.635704	-2.876932	3.175539	C	4.467168	-2.109851	-1.954180
H	-1.386197	-2.083045	3.076598	H	4.983160	-1.252171	-1.506931
C	-4.620361	-0.328201	-2.221428	C	1.870175	2.567145	2.667274
H	-4.365314	-1.233689	-1.658113	H	2.541970	1.704210	2.590840
C	-3.541519	1.711821	2.333847	C	2.637819	2.296721	-2.376825
H	-3.036046	0.742322	2.403619	H	3.252743	1.464925	-2.015525
H	-5.842671	4.033830	-1.015412	H	2.862972	-6.064700	0.008160
H	2.130323	-5.227121	0.230902	H	0.057169	5.647654	-0.355367
C	-2.466000	2.797738	2.512456	C	3.155439	-2.688116	3.857837
H	-1.921254	2.643830	3.450584	H	4.214818	-2.488869	3.661326
H	-2.911217	3.799035	2.542980	H	3.047150	-3.760276	4.058908
H	-1.750422	2.764623	1.684957	H	2.861695	-2.143243	4.761939
C	-4.594641	1.781346	3.455197	C	0.786673	-2.509089	2.959960
H	-5.347543	0.993389	3.341940	H	0.589754	-3.569394	3.158921
H	-5.115755	2.745720	3.444466	H	0.167579	-2.187820	2.115211
H	-4.117761	1.666066	4.434943	H	0.475460	-1.936561	3.840533
C	0.533009	-2.320392	4.009536	C	2.532250	3.596177	3.601432
H	1.256783	-3.105744	4.255158	H	2.732581	3.145526	4.579906
H	0.155349	-1.916271	4.955266	H	1.880500	4.462616	3.760910
H	1.064931	-1.521985	3.484644	H	3.478394	3.959911	3.185696
C	-1.286101	-4.063672	3.917472	C	0.544205	2.053830	3.256430
H	-2.115443	-4.497429	3.349193	H	0.097770	1.282070	2.620535
H	-1.664870	-3.743082	4.894334	H	-0.180091	2.870995	3.359255
H	-0.549472	-4.858444	4.083225	H	0.711803	1.624751	4.251168
C	-1.972197	-4.228851	-2.578084	C	3.407610	-1.537288	-2.914195
H	-2.594015	-4.789400	-1.871202	H	2.828821	-2.345258	-3.376329
H	-1.195446	-4.905785	-2.951334	H	3.889156	-0.958736	-3.711401
H	-2.596660	-3.933572	-3.428854	H	2.713093	-0.881351	-2.382809
C	-0.489355	-2.191752	-2.908183	C	5.521055	-2.916683	-2.729088
H	-1.060129	-1.957935	-3.813355	H	5.061256	-3.706268	-3.333598
H	0.400467	-2.758691	-3.206154	H	6.247675	-3.380479	-2.053924
H	-0.162406	-1.247126	-2.461445	H	6.061788	-2.256477	-3.415521
C	-5.940832	-0.603319	-2.959512	C	1.546022	1.691191	-3.273079
H	-6.765897	-0.748847	-2.254089	H	1.994146	1.123288	-4.096564
H	-5.846623	-1.506603	-3.572222	H	0.912160	2.474245	-3.704324

H	-6.208772	0.220625	-3.629926	H	0.909686	1.019413	-2.688634
C	-3.469514	-0.071582	-3.212732	C	3.555337	3.241486	-3.172946
H	-2.520429	0.070825	-2.682854	H	4.339588	3.660359	-2.533196
H	-3.663969	0.830736	-3.804085	H	2.990374	4.076576	-3.602275
H	-3.359818	-0.917770	-3.901255	H	4.032851	2.701471	-3.998399
O	0.246042	1.976254	-1.159136	H	-3.802689	0.527088	-1.131895
C	1.635968	3.807798	-0.558099	C	-1.909499	1.641285	-1.241103
C	0.529042	4.478854	-0.014479	C	-1.946175	1.786134	-2.767797
C	2.863611	4.485564	-0.638012	H	-0.867039	1.441563	-0.947018
C	0.651593	5.783771	0.459466	H	-1.424029	2.696079	-3.079163
H	-0.420841	3.958185	0.020833	H	-1.442703	0.950231	-3.258560
C	2.984009	5.792875	-0.167001	H	-2.975405	1.831854	-3.144860
H	3.724850	4.003077	-1.088130	C	-3.719571	-0.772677	1.235902
C	1.880391	6.444701	0.389045	C	-3.389531	-1.757671	2.178689
H	-0.212109	6.285137	0.883598	C	-5.071659	-0.532185	0.950971
H	3.937406	6.305589	-0.240790	C	-4.386446	-2.510849	2.795412
H	1.977045	7.460097	0.758514	H	-2.342360	-1.919733	2.406482
H	3.544703	1.925169	-1.074153	C	-6.068698	-1.283990	1.569478
C	2.373918	0.196432	-1.817971	H	-5.349688	0.241993	0.245038
C	2.060571	0.476424	-3.305989	C	-5.729824	-2.281103	2.486912
H	1.502942	-0.318780	-1.390956	H	-4.117365	-3.276475	3.516025
H	1.921197	-0.467392	-3.844971	H	-7.110270	-1.094045	1.333671
H	2.885649	1.019579	-3.780937	H	-6.507545	-2.867870	2.964728
H	1.146987	1.071586	-3.393920	O	-1.439261	-0.119869	1.090122
H	-4.684006	-1.265767	2.089225	H	4.954669	1.641926	-0.335938
H	-3.360942	-3.261625	2.055749	H	5.587205	-0.567567	0.397625
C	3.557169	-0.747579	-1.707868	C	-2.330925	2.938471	-0.529446
C	3.338252	-2.084523	-1.361442	C	-2.146102	3.052020	0.857853
C	4.866157	-0.334352	-1.989295	C	-2.900809	4.022723	-1.208564
C	4.397291	-2.990892	-1.288057	C	-2.514564	4.208171	1.540175
H	2.327991	-2.409052	-1.133145	H	-1.697106	2.226883	1.397164
C	5.928550	-1.233721	-1.909482	C	-3.264674	5.189279	-0.526500
H	5.062115	0.700098	-2.256532	H	-3.068068	3.973578	-2.278552
C	5.698792	-2.567203	-1.559579	C	-3.072644	5.288634	0.850582
H	4.204961	-4.024075	-1.013850	H	-2.360047	4.268413	2.613343
H	6.938566	-0.892737	-2.111112	H	-3.705105	6.015948	-1.075376
H	6.527085	-3.265657	-1.497739	H	-3.358925	6.190934	1.381298
